# Catalytic, Kinetic, and Mechanistic Insights into the Fixation of CO_2_ with Epoxides Catalyzed by Phenol‐Functionalized Phosphonium Salts

**DOI:** 10.1002/cssc.202002267

**Published:** 2020-11-13

**Authors:** Yuya Hu, Zhihong Wei, Anna Frey, Christoph Kubis, Chang‐Yue Ren, Anke Spannenberg, Haijun Jiao, Thomas Werner

**Affiliations:** ^1^ Leibniz Institute for Catalysis e. V. Albert-Einstein-Straße 29a 18059 Rostock Germany; ^2^ Institute of Molecular Science Key Laboratory of Materials for Energy Conversion and Storage of Shanxi Province Shanxi University Taiyuan 030006 P. R. China

**Keywords:** CO_2_ fixation, cyclic carbonates, homogeneous catalysis, mechanism, organocatalysts

## Abstract

A series of hydroxy‐functionalized phosphonium salts were studied as bifunctional catalysts for the conversion of CO_2_ with epoxides under mild and solvent‐free conditions. The reaction in the presence of a phenol‐based phosphonium iodide proceeded via a first order rection kinetic with respect to the substrate. Notably, in contrast to the aliphatic analogue, the phenol‐based catalyst showed no product inhibition. The temperature dependence of the reaction rate was investigated, and the activation energy for the model reaction was determined from an Arrhenius‐plot (*E*_a_=39.6 kJ mol^−1^). The substrate scope was also evaluated. Under the optimized reaction conditions, 20 terminal epoxides were converted at room temperature to the corresponding cyclic carbonates, which were isolated in yields up to 99 %. The reaction is easily scalable and was performed on a scale up to 50 g substrate. Moreover, this method was applied in the synthesis of the antitussive agent dropropizine starting from epichlorohydrin and phenylpiperazine. Furthermore, DFT calculations were performed to rationalize the mechanism and the high efficiency of the phenol‐based phosphonium iodide catalyst. The calculation confirmed the activation of the epoxide via hydrogen bonding for the iodide salt, which facilitates the ring‐opening step. Notably, the effective Gibbs energy barrier regarding this step is 97 kJ mol^−1^ for the bromide and 72 kJ mol^−1^ for the iodide salt, which explains the difference in activity.

## Graphical abstract

**CO_2_ fixation**: Phenol‐functionalized phosphonium salts are efficient catalysts for the synthesis of cyclic carbonates under mild and solvent‐free conditions. Their superior activity is elucidated by kinetic investigations and DFT calculations.

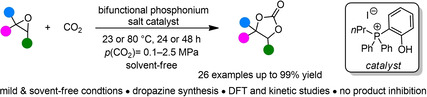

## Introduction

**Scheme 1:**
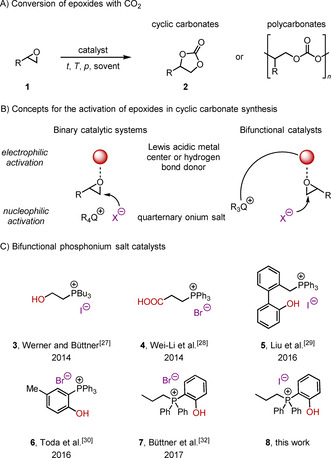
Synthesis of cyclic and polycarbonates as well as concepts and selected catalysts for the conversion of epoxides with CO_2_.

The use of CO_2_ as a C_1_ building block is receiving increasing attention from the scientific and industrial community due to economic and ecological considerations.^[1]^ It is progressively regarded as an attractive, inexpensive, and abundant renewable feedstock rather than waste.^[2]^ Furthermore, in a future CO_2_ based circular economy it can be used as a platform to produce bulk chemicals and energy carriers through its transformation to fuels.^[3]^ Nevertheless, due to its thermodynamic stability, the efficient activation and chemical fixation is still challenging.^[4]^ This can be partially overcome by converting CO_2_ with high‐energy starting materials such as epoxides or hydrogen in the presence of a catalyst. Thus, it is crucial to develop new catalytic processes that allow the efficient transformation of CO_2_ into valuable products. Promising transformations even on an industrial scale are the fixation of CO_2_ into organic carbonates or polycarbonates (Scheme 1, A).[^1b^, ^5^] These processes can be of significant advantage regarding economic and ecological considerations, for example, by saving fossil resources or lowering the carbon footprint of a process.^[6]^

The synthesis of five‐membered cyclic carbonates **2** from CO_2_ and epoxides **1** is of particular interest. It is an atom‐economic reaction and an excellent example of green chemistry.^[7]^ Moreover, cyclic carbonates are important materials that are used in various applications, for example, as intermediates in organic synthesis,^[8]^ indirect CO_2_ reductions to methanol,^[9]^ green solvents,^[10]^ electrolyte in lithium ion batteries,^[11]^ polymer building blocks,^[12]^ and even as additives in drugs and cosmetics.^[13]^

Metal‐based systems including Al‐salen^[14]^ and triphenolat^[15]^ complexes have been reported for the synthesis of cyclic carbonates.^[16]^ In these binary catalytic systems, the Lewis‐acidic metal complexes are often combined with an external nucleophile, typically a halide ion from an onium salt, to activate the epoxide (Scheme 1, B). The nucleophilic counter anion of the salt acts as a temporary relay, ring‐opening the epoxide, which is activated by the Lewis‐acidic metal center, and subsequently serving as a leaving group upon ring closure after CO_2_ insertion. Catalytic systems that operate under near‐ambient conditions (low CO_2_ pressure and room temperature) are of interest in terms of advances towards sustainability.^[17]^ In the past decade, the use of organocatalysts has attracted increasing attention in this research area.^[18]^ A significant benefit of this catalyst class is undoubtedly the metal‐free carbon‐based scaffold, which is associated with a high potential for structural modification, catalyst tuning, and immobilization.^[19]^ In analogy to the metal‐based systems, the combination of hydrogen bond donors and quaternary ammonium salts to activate the epoxide proved to be a promising concept in the field of organocatalyzed cyclic carbonate synthesis (Scheme 1, B).^[20]^ Systems based on different hydrogen bond donor functionalities, such as alcohols,^[21]^ carboxylic acids,^[22]^ silanol,^[23]^ phenols,^[24]^ and (thio‐)ureas,^[25]^ were used in combination with external nucleophiles to catalyze the addition of CO_2_ to epoxides to yield the corresponding cyclic carbonates. Even though great advances have been made it is still challenging to realize this reaction efficiently under low CO_2_ pressures (1 atm) and reaction temperatures below 100 °C.^[17]^

It is generally recognized that bifunctional catalysts consisting of an electrophilic group and an internal nucleophile significantly accelerate the process through synergistic effects.[^18^, ^26^] However, it was not until recently that bifunctional phosphonium salts emerged as potent class of organocatalysts (Scheme 1, C). In 2014 our group reported the use of bifunctional phosphonium salt catalyst **3** bearing an alcohol moiety as hydrogen bond donor. This catalyst allowed for the synthesis of cyclic carbonates at 90 °C and 1.0 MPa CO_2_ pressure, yielding the desired products in up to 99 % yield under solvent‐free conditions.^[27]^ More recently, we reported a mechanistic study on the use of bifunctional phosphonium salt **3**.^[26a]^ This study clearly showed that the epoxide is activated by hydrogen bonding and that the order of reactivity in regard to the anion increased in the order Cl^−^<Br^−^<I^−^. Significantly, the analysis of the kinetic data revealed that partial product inhibition hampered the overall efficiency of this catalyst. Also in 2014, Wei‐Li et al. introduced carboxylic acid derivative **4** as a catalyst for the synthesis of cyclic carbonates at elevated temperatures and pressure (130 °C and 2.5 MPa).^[28]^ In 2016, Liu et al. showed that biphenyl‐derived phosphonium salt **5** enables the conversion of epoxides with CO_2_ at 60 °C and low CO_2_ pressure (1.0 atm).^[29]^ In the same year, Toda et al. studied bifunctional tetraarylphosphonium salt **6**, which allowed for the synthesis of cyclic carbonate at atmospheric CO_2_ pressure but high reaction temperatures of 120 °C in chlorobenzene as the solvent.^[30]^ Very recently, they carefully investigated the impact of the electronic properties of the substituents on the catalyst efficiency.^[31]^ The introduction of electron‐donating groups significantly enhanced the catalytic activity, which allowed to reduce the reaction temperature to 60 °C. In 2017 our group reported the use of bifunctional phenolic phosphonium bromide **7** for the preparation of oleochemical carbonates at 80 °C and 2.5 MPa CO_2_ pressure.^[32]^ Based on our previous results we envisioned that the corresponding iodide **8** should show superior efficiency as catalyst in the synthesis of cyclic carbonates under mild and solvent free‐conditions. Herein we report the synthesis and application of this catalyst as well as thorough kinetic investigations and DFT calculations to rationalize its superior performance and the reaction mechanism.

## Results and Discussion

### Catalyst screening

We chose the conversion of 1,2‐butylene oxide (**1 a**) with CO_2_ to produce 1,2‐butylene carbonate (**2 a**) as a benchmark system to evaluate and compare to previously reported catalysts under uniform reaction conditions (Table 1). Our first‐generation bifunctional phosphonium salt **3** (2 mol %) gave the desired product **2 a** in 25 % yield after 24 h at 23 °C and 1.0 MPa CO_2_ pressure (Table 1, entry 1). In the presence of catalysts **5**, **6**, and **7** significantly lower conversions and yields ≤10 % were achieved (entries 2–4). In contrast, phosphonium iodide **8** gave **2 a** in 65 % yield (entry 5).

**Table 1:** Comparison of bifunctional phosphonium salts in the cycloaddition of CO_2_ with epoxides.

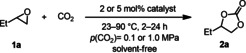						
Entry	Catalyst	*Loading* [mol %]	*T* [°C]	*t* [h]	Yield^[a]^ [%]	TON
1	**3**	2	23	24	25	13
2	**5**	2	23	24	2	1
3	**6**	2	23	24	2	1
4	**7**	2	23	24	10	5
5	**8**	2	23	24	65	33
6	**7**	2	45	6 (24)	60 (>99)	30
7	**8**	2	45	6 (24)	85 (>99)	43
8	**7**	2	90	2	89	45
9	**8**	2	90	2	96	48
10	**8**	5	23	24	>99	50
11^[b]^	**8**	5	23	24	71	36

Reaction conditions: 1.0 equiv. **1 a** (1.00 g, 13.9 mmol), 2–5 mol % catalyst, 2–24 h, *p*(CO_2_)=1.0 MPa, solvent‐free. [a] Yields were determined by ^1^H NMR spectroscopy using mesitylene as internal standard. [b] *p*(CO_2_)=0.1 MPa. TON: turnover number.

This is remarkable since catalyst **7** was identified to be more efficient compared to **8** at higher temperature (100 °C) and CO_2_ pressure (5.0 MPa) in the synthesis of oleochemical carbonates.^[32]^ With increasing reaction temperature the obtained yield for **2 a** equalizes (entries 6–9). Notably, in the presence of 5 mol % **8** an excellent yield >99 % was achieved at 23 °C (entry 10). Even at a lower CO_2_ pressure of 0.1 MPa **2 a** was obtained in 71 % yield (entry 11). The lower yield might be due to a lower CO_2_ concentration in solution at this pressure, which can have a strong impact on the reaction rate.^[33]^

### Kinetic investigations

The efficiency of bifunctional phosphonium salts is closely related to the ability of activating the epoxide by hydrogen bonding.^[20]^ Recently, we reported a detailed mechanistic study on alkyl‐bridged bifunctional phosphonium salts as catalysts in cyclic carbonate synthesis.^[26a]^ In this study the activation of epoxide **1 a** by hydrogen bonding to catalyst **3** was proven by IR spectroscopy. Moreover, kinetic and IR studies revealed an interaction of **3** with the product **2 a** leading to significant product inhibition. In the present case attempts failed to investigate the interaction of phenolic catalyst **8** with the epoxide or the carbonate by transmission IR spectroscopy due to an overlap of the OH‐stretching vibration from the phenol moiety with bands of the C−H stretching modes. Unfortunately, also measurements in the attenuated total reflection (ATR) mode did not provide unambiguous results in this regard.^[34]^

**Figure 1:**
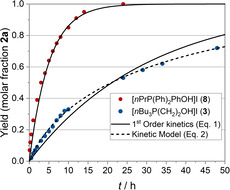
Comparison of a first‐order kinetic fit and the kinetic model represented in Equation (2) for the conversion of epoxide **1 a** with CO_2_ in the presence of catalyst **3** and **8**. The observed selectivity was >99 %. Reaction conditions: 1,2‐butylene oxide (**1 a**, 460 mmol), 2 mol % catalyst **3** or **8**, *p*(CO_2_)=1.0 MPa, 45 °C, 48 h.

However, to further investigate the difference in the activity of catalysts **3** and **8**, we performed kinetic measurements of the model reaction with 1,2‐butylene oxide (**1 a**) as the substrate at 23 and 45 °C. These experiments have been conducted at a CO_2_ pressure of 1.0 MPa with a substrate content of 460 mmol and 2 mol % of catalyst. Product formation was monitored by the sampling of the liquid reaction mixture at distinct time intervals followed by ^1^H NMR spectroscopic analyses. The selectivity of **2 a** was consistently >99 %. At 23 °C with **8** as a catalyst, an induction period was observed, which was attributed to the low solubility of **8** in the pure epoxide at room temperature.^[34]^ For the catalyst system **3**, no induction period has been identified, but at higher conversions the catalytic performance is significantly lower compared to the phenolic catalyst **8**. In the next step, we performed these model reactions at 45 °C. Now, for the catalyst **8**, the yield versus time data can be described by a first‐order kinetic model [Eq. (1)], in which *Y* is the yield and *k* ^obs^ is the observable rate constant, as shown in Figure 1.

(1) $$\frac{dY}{dt}=k^{obs}\left(1-Y\right)$$

(2) $$\frac{dY}{dt}=\frac{k^{obs}\left(1-Y\right)}{1+K_{\mathrm{inh}}{\left[S\right]}^{0}Y}$$

In contrast, for the reaction in the presence of **3** a kinetic model in which product inhibition was considered needs to be used [Eq. (2)]. This equation is based on a Michaelis‐Menten model including a reversible product inhibition, which is valid for the case of a first order with respect to the substrate. The equilibrium constant *K*_inh_ of the product inhibition with the aliphatic catalyst **3** was calculated as 0.260 L mol^−1^ for the given initial substrate concentration [S]^0^. The fact that the yield versus time data for **8** was best described with the first‐order model indicates that product inhibition is not relevant for this system. The obtained observable rate constants *k* ^obs^ were 0.197 h^−1^ (**8**) >0.0605 h^−1^ (**3**), reflecting the catalytic activity.^[34]^ From these observations it can be concluded that both the absence of a significant product inhibition and an intrinsically higher activity for the catalytic system with catalyst **8** cause its better performance.

**Figure 2:**
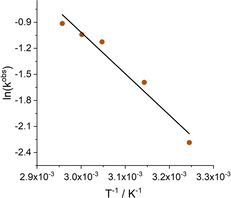
Arrhenius‐plot for the estimation of the activation energy *E*_a_ of the conversion of epoxide **1 a** with CO_2_ in the presence of catalyst **8** over the temperature range of 35–65 °C (*T*=308.15–338.15 K). Reaction conditions: 1,2‐butylene oxide (**1 a**, 460 mmol), 2 mol % catalyst **8**, *p*(CO_2_)=1.0 MPa. *y*=−476.5*x*+13.277, *R* ^2^=0.9638.

We were further interested in the temperature dependence of the reaction rate for the catalytic system with the phenolic catalyst **8**.^[34]^ Therefore, additional experiments have been performed so that a temperature range of 35–90 °C was covered. Yield versus time data was analyzed using a first‐order kinetic model (first‐order with respect to the substrate concentration) providing observable rate constants (*k* ^obs^). An acceptable Arrhenius behavior was found between 35–65 °C (*T*=308.15–338.15 K). Interestingly, at higher temperatures the values of the rate constant decreased, which might be attributed to a change in the solubility of CO_2_ at higher temperatures.^[33b]^ From an Arrhenius‐plot a value for the activation energy was calculated with *E*_a_=39.6 kJ mol^−1^, which is in agreement with previous reports (Figure 2).^[35]^ A respective Eyring‐plot allowed for the calculation of the enthalpy of activation, which gave a value of Δ*H* ^≠^=36.9 kJ mol^−1^.

### Substrate scope

**Figure 3:**
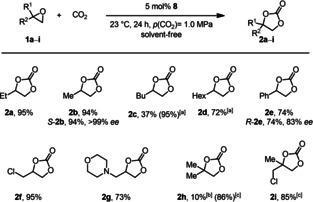
Substrate scope for the conversion of terminal epoxides **1** into the corresponding carbonates **2**. Reaction conditions: 1.0 equiv. **1** (1.00 g), 5 mol % **8**, 23 °C, 24 h, *p*(CO_2_)=1.0 MPa, solvent‐free. Isolated yields are given. [a] *n*‐Butanol was employed as a solvent. [b] Yields were determined by ^1^H NMR spectroscopy using mesitylene as internal standard. [c] 80 °C, *p*(CO_2_)=2.5 MPa.

Due to the observed superior efficiency of catalyst **8** we evaluated the substrate scope regarding the conversion of terminal epoxides **1** to the respective cyclic carbonates **2** at room temperature (Figure 3). Carbonates **2 a**–**d** bearing aliphatic substituents were synthesized in good to excellent yields. Particularly, propylene carbonate (**2 b**) has attracted much interest due to its applications as an electrolyte in lithium‐ion batteries^[11]^ and is regarded as one of the most sustainable alternative solvents in organic chemistry.^[10a]^ Notably, the conversion of enantiomerically pure (*S*)‐propylene oxide (*S*‐**1 b**) led to the corresponding cyclic carbonates *S*‐**2 b** in 94 % yield and >99 % enantiomeric excess (*ee*). Due to the lower polarity of epoxides **1 c** and **1 d** the solubility of the catalyst was reduced, and the addition of a solvent (*n*BuOH) was required to achieve 95 and 72 % yield for **2 c** and **2 d**, respectively. The phenyl‐substituted product **2 e** was isolated in a yield of 74 %. In this case acetophenone was found as a by‐product that was derived from a Meinwald rearrangement.^[36]^ This indicates that the conversion of styrene oxide (**1 e**) proceeds at least partially via a cationic intermediate. This assumption is supported by the partial racemization, which was observed when (*R*)‐styrene oxide (*R*‐**1 e**) was converted. In this case, carbonate *R*‐**2 e** was isolated in 74 % yield and 83 % *ee*. Furthermore, epichlorohydrin (**1 f**) was converted to the respective carbonate **2 f** in an excellent yield of 95 % while morpholine derivative **2 g** was obtained in 73 % yield. After having established a general protocol for the conversion of monosubstituted epoxides under mild conditions, the catalyst system was applied in converting disubstituted terminal epoxides. Products **2 h** and **2 i** were obtained in yields of 86 and 85 %, respectively, even though elevated reaction temperature (80 °C) and higher CO_2_ pressure (2.5 MPa) were required.

Cyclic carbonates are frequently used as protecting groups for 1,2‐diols.^[37]^ The successful preparation of morpholine derivative **2 g** prompted us to evaluate the synthesis of dropropizine *rac‐* **10**, which is an antitussive agent typically employed as a racemic mixture in a number of commercial cough suppressant.^[38]^ In general, it is prepared from the epoxides **1 j** under acidic conditions or by direct amination of solketal, a 1,2‐hydroxy‐protected derivative of glycerol, via ruthenium‐catalyzed hydrogen borrowing reaction and subsequent ketal hydrolysis.^[39]^

**Scheme 2:**
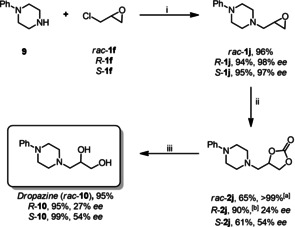
Synthesis of dropropizine via cyclic carbonate **2 j**. Reaction conditions: i) 23 °C, 1 h, H_2_O then NaOH/H_2_O, 75 °C, 15 min. ii) 5 mol % **8**, 23 °C, 48 h, *p*(CO_2_)=1.0 MPa. iii) NaOH/H_2_O, 3 h, 23 °C. [a] 45 °C. [b] 45 °C, 24 h.

The intermediate epoxide *rac*‐**1 j** as well as enantiomerically pure *R*‐ and *S*‐**1 j** were obtained in yields up to 96 % from phenylpiperazine (**9**) and the respective epichlorohydrin (**1 f**) (Scheme 2). The subsequent conversion under the standard reaction conditions for terminal epoxides gave the respective carbonate *rac*‐**2 j** in 65 % after 48 h. At 45 °C full conversion was achieved, and **2 j** was isolated in >99 % yield. The subsequent cleavage of the carbonate protecting group under basic conditions gave dropropizine *rac*‐**10** in 95 % yield. Notably, the formation and subsequent cleavage of the carbonate to obtain dropropizine in a sequential one‐pot reaction could also be achieved, leading to *rac*‐**10** in 61 % yield. We envisioned that also enantiomerically pure dropropizine should be accessible via this route. Notably, this protocol circumvents the acidic hydrolysis of the epoxide to the 1,2‐diol, which might lead to racemization. The precursors *R*‐**1 j** and *S*‐**1 j** were readily accessible from (*R*)‐ and (*S*)‐epichlorohydrin (*R*‐ and *S*‐**1 f**) and amine **9** in excellent yields and enantioselectivities of 94 % (98 % *ee*) and 95 % (97 % *ee*), respectively. The conversion of *R*‐**1 j** with CO_2_ at 45 °C led to carbonate *R*‐**2 j** in excellent 90 % yield. However, under these conditions partial racemization was observed, and *R*‐**2 j** was obtained in 24 % *ee*. Thus, *S*‐**2 h** was converted at lower temperature (23 °C), which led to a significant improvement to 54 % *ee* but a lower yield of 61 %. The deprotection of carbonates *R*‐**2 j** and *S*‐**2 j** occurred stereoselectively as expected to yield enantiomerically enriched *R*‐**10** and *S*‐**10** in 95 and 99 % yield, respectively.

**Figure 4:**
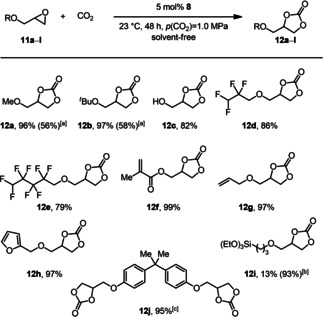
Substrate scope for the conversion of glycidol and its derivatives **11** into the corresponding carbonates **12**. Reaction conditions: 1.0 equiv. **11** (1.00 g), 5 mol % **8**, 23 °C, 48 h, *p*(CO_2_)=1.0 MPa, solvent‐free. Isolated yields are given. [a] 24 h. [b] 2 mol % **8**, 90 °C, 4 h. [c] 10 mol % **8**, 45 °C, *n*‐butanol as solvent.

As the major by‐product in the manufacturing of biodiesel, glycerol is widely available.^[40]^ Thus, the use of glycerol as the feedstock for the preparation of value‐added products is an attractive goal. Moreover, the use of this biobased material can lead to a significant reduction in the carbon footprint, for example, in the synthesis of carbonates, compared to their production from fossil resources.^[6a]^ Glycidol (**11 c**) and its derivatives can be obtained from glycerol.^[41]^ The respective carbonates show unique properties and find a range of applications, for example, as synthetic building blocks, monomers, and solvents.[^10b^, ^42^] Hence, we were particularly interested in the preparation of cyclic carbonates **12 a**–**j** from epoxides **11 a**–**j** in the presence of catalyst **8** (Figure 4). Under the standard conditions the conversion of glycidyl ethers **11 a** and **11 b** did not lead to full conversion, and **12 a** and **12 b** were isolated in 56 and 58 % yield, respectively. Hence, we adjusted the reaction time to 48 h, which led to full conversion and excellent isolated yields of 96 and 97 %. The conversion of glycidol (**11 c**), which is susceptible to polymerization,^[43]^ gave glycerol carbonate **12 c** in 82 %. In addition, the highly fluorinated carbonates **12 d** and **12 e**, which can be used as electrolytes in lithium batteries,^[44]^ were prepared in yields of 86 and 79 % respectively. Furthermore, the unsaturated carbonates **12 f** and **12 g**, which are potential building blocks for homo‐ and copolymers with cyclic carbonate units in the backbone, were isolated in distinguished yields up to 99 %.^[45]^ Epoxide **11 h**, containing a furfuryl moiety, can also be synthesized from renewables,^[46]^ and the respective carbonate was isolated in 97 % yield. Silyl‐functionalized carbonates are often used as precursors for the synthesis of non‐isocyanate polyhydroxy‐urethane hybrid materials.^[47]^ They also find applications in industry due to their potential utilization as electrolytes^[48]^ and in surface modification. Thus, we converted **11 i** with CO_2_ in the presence of catalyst **8** and were able to obtain **12 i** in 93 % under slightly modified conditions. Additionally, bisphenol diglycidyl ether (**11 j**) was converted into the respective carbonate **12 j** in 95 % yield. This biscarbonate is a frequently used monomer for the synthesis of non‐isocyanate polyurethanes (NIPUs).^[49]^

**Figure 5:**
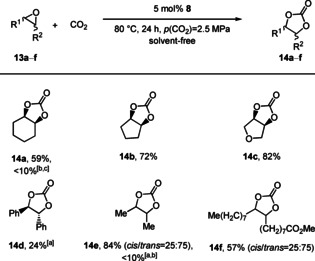
Substrate scope for the conversion of internal epoxides **13** into the corresponding carbonates **14**. Reaction conditions: 1.0 equiv. **13** (1.00 g), 5 mol % **8**, 80 °C, 24 h, *p*(CO_2_)=2.5 MPa, solvent‐free. Isolated yields are given. [a] *n*‐Butanol was employed as a solvent. [b] 23 °C, *p*(CO_2_)=1.0 MPa. [c] Yields were determined by ^1^H NMR spectroscopy using mesitylene as the internal standard.

The synthesis of internal carbonates derived from epoxides and CO_2_ is rather challenging and is often marginally studied in the evaluation of the substrate scope. Considering the high efficiency of catalyst **8** in the conversion of terminal epoxides under mild conditions we were interested in its performance regarding the conversion of internal epoxides **13** (Figure 5). Initially, the standard protocol [5 mol %, 23 °C, 24 h, *p*(CO_2_)=1.0 MPa] was tested for the conversion of cyclohexene oxide (**13 a**). Under these conditions <10 % of the desired carbonate **14 a** was obtained. Hence, the reaction temperature and CO_2_ pressure were increased to 80 °C and 2.5 MPa, respectively. This led to significant improvement of the yield, and **14 a** was obtained in 59 %. Moreover, the conversion of epoxides **13 b** and **13 c** led to the corresponding cyclic carbonates **14 b** and **14 c** in 72 and 82 % yield, respectively. Notably, the conversion of *cis*‐stilbene oxide (**13 d**) gave the *trans*‐stilbene carbonate *trans‐* **14 d** in a selectivity of 99 %. This indicates that the reaction proceeds via a cationic intermediate (S_N_1‐pathway), which is stabilized by the phenyl substituent.^[34]^ This pathway leads to the thermodynamically more stable *trans*‐product. Moreover, 1,2‐diphenyl‐ethan‐1‐one was observed as a by‐product, which comes from the Meinwald rearrangement.^[36]^ In contrast, the conversion of *cis*‐**13 e** and *cis*‐methyl oleate (**13 f**) gave the respective carbonates in yields up to 84 % with a *cis*/*trans‐*selectivity of 25 : 75. This can be attributed to the less pronounced stabilization of the cationic intermediate by hyperconjugation in the S_N_1‐pathway.

### DFT calculations

**Figure 6:**
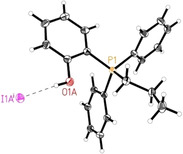
Molecular structure of catalyst **8** in the solid state. Displacement ellipsoids correspond to 30 % probability. Lower occupancy sites are omitted for clarity. The intermolecular hydrogen bond is shown as dashed line.^[51]^

On the basis of our findings and previous reports^[50]^ we propose a three‐step mechanism. The initial step is the ring‐opening of epoxide **1 a**. Subsequently, the positively charged carbon atom of CO_2_ couples with the negatively charged oxygen atom to form a linear carbonate; alternatively, this step can also be considered as a nucleophilic attack of the negatively charged oxygen atom to the lowest unoccupied molecular orbital (LUMO) of CO_2_. Finally, an intramolecular nucleophilic substitution leads to the formation of the desired cyclic carbonate and liberates the catalyst. Since the nucleophilic counterion has been found to have an important effect on the activity of the catalyst, both bromide salt **7** and iodide salt **8** were considered. On the basis of this proposal, we calculated the full Gibbs free‐energy surface for the cycloaddition of CO_2_ to epoxide **1 a** catalyzed by phenol‐derived phosphonium halides **7** and **8**, affording the five‐membered cyclic carbonate **2 a**. All computational details are given in the Supporting Information. Here we present the results for the more active catalyst **8**, while those for the less active bromide salt **7** can be found in the Supporting Information. The optimized structure **I** shows a hydrogen bonding (2.297 Å) interaction between the phenolic OH and the I^−^ counter ion. Notably, this interaction can also be seen in the crystal structure of catalyst **8** (Figure 6, O1A−H1A⋅⋅⋅I1A^i^: O1A−H1A=0.89(5) Å, H1A⋅⋅⋅I1A^i^=2.48(5) Å, O1A⋅⋅⋅I1A^i^=3.365(3) Å, O1A−H1A⋅⋅⋅I1A^i^=172(4)°, symmetry code: (i) −1/2+*x*,*y*,1/2−*z)*.

**Scheme 3:**
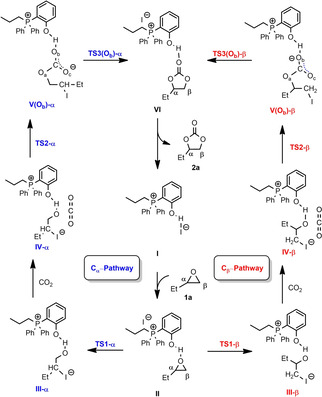
Intermediates of the calculated catalytic cycle for ring‐opening at the methylene (C_β_, right) and methine carbon (C_α_, left) at epoxide **1 a**.

For catalyst **8**, two possible pathways for the ring‐opening at the methylene (C_β_, Scheme 3, right) and methine carbon (C_α_, Scheme 3, left) in the epoxide function were evaluated.

**Figure 7:**
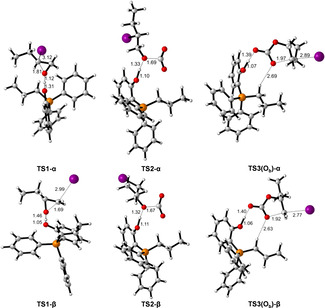
Optimized structures for transition states of **TS1‐α** to **TS3‐α** as well as **TS1‐β** to **TS3‐β**.

**Figure 8:**
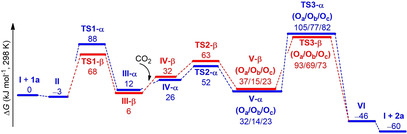
Calculated Gibbs free‐energy profile for the addition of CO_2_ to epoxide **1 a** in the presence of catalyst **8** for the reaction at the methylene (C_β_) and methine carbon (C_α_).

The structure of the intermediates in the catalytic cycle are shown in Scheme 3 while for clarity the optimized structures of transition states **TS1‐α** to **TS3‐α** as well as **TS1**‐**β** to **TS3‐β** are shown separately in Figure 7. The corresponding Gibbs free‐energy profile for the opening at C_α_ and C_β_ is depicted in Figure 8. The initial step is the epoxide coordination to the phenol‐derived phosphonium salt to form intermediate **II** via hydrogen bonding. The coordination is exergonic by 3 kJ mol^−1^. This energy difference indicates dynamic equilibrium in favor of **II** (77 %). The next step is the nucleophilic attack of I^−^ to the carbon of the epoxide with C−O bond cleavage and the formation of a C−I bond.

Notably, the ring‐opening at C_β_ via transition state of **TS1‐β** has an energy barrier of 68 kJ mol^−1^, which is lower than that at C_α_ via transition state of **TS1‐α** by 20 kJ mol^−1^. This can be attributed to the steric interaction as estimated on the basis of the distortion energy of epoxide. Namely, the geometrical strain energy Δ*E*_strain_ of **1 a** in **TS1‐β** is 30.6 kJ mol^−1^ lower than that in **TS1‐α**. The proton of the phenol group is transferred to the oxygen of iodo butanolate forming an ylide and iodo‐butanol. The formation of **III** from **II** is endergonic by 15 and 9 kJ mol^−1^ for 2‐iodobutan‐1‐ol (**III‐α**) and 1‐iodobutan‐2‐ol (**III‐β**), respectively. The co‐adsorption of CO_2_ from **III‐α** and **III‐β** to form **IV‐α** and **IV‐β** is also endergonic by 14 and 26 kJ mol^−1^, respectively. The energy barrier of C−O coupling via transition state of **TS2‐α** and **TS2‐β** is 40 and 57 kJ mol^−1^ from **III‐α** and **III‐β**, respectively. The formation of 2‐iodobutyl carbonate anion **V‐α** and 1‐iodobutan‐2‐yl carbonate anion **V‐β** in which O_b_ is interacting with the OH of phenol is endergonic by 2 and 9 kJ mol^−1^ from **III‐α** and **III‐β**, respectively.

**Figure 9:**
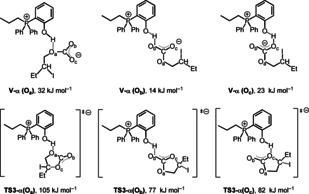
The three possibilities of the stabilized intermediates **Vα** (O_a_, O_b_, and O_c_) and the transition states **TS3‐α** (O_a_, O_b_, and O_c_) in the C_α_ pathway. The structures of O_b_ hydrogen bonding interaction have the lowest energy.

For the last step (the ring‐closure reaction), three possible pathways were calculated considering that three oxygen (O_a_/O_b_/O_c_) in carbonate may interact with the OH group of phenol (Figure 9). For the α route, the interaction in **V‐α** between 2‐iodobutyl carbonate through O_a_ and O_c_ with OH of phenol is less favorable than that through O_b_ by 21 and 8 kJ mol^−1^, respectively. This indicates the strong stabilization of the carboxylate anion through the hydrogen bonding between the OH‐group of the phenol and O_b_ and O_c_ of the carboxylate moiety, respectively.

For the last step through the α route, the ring closure through the O_a_, O_b_, and O_c_ routes from the most stable **V‐α** (O_b_) via **TS3‐α** to the carbonate–catalyst adduct **VI** has an energy barrier of 91, 63, and 68 kJ mol^−1^, respectively. Notably, the formation of adduct **VI** is exergonic by 60 kJ mol^−1^. For the β route, the interaction in **V‐β** between 2‐iodobutyl carbonate through O_b_ and O_c_ with OH of phenol is more favorable than that through O_a_ by 22 and 14 kJ mol^−1^, respectively. The ring closure through the O_a_, O_b_, and O_c_ route from **V‐β** (O_b_) via **TS3‐β** to form the adduct **VI** has an energy barrier of 78, 54, and 58 kJ mol^−1^, respectively. The formation of cyclic carbonate from **V‐β** (O_b_) is exergonic by 61 kJ mol^−1^. Notably, the release of the cyclic carbonate **2 a** from the catalyst‐carbonate adduct **VI** is exergonic by 14 kJ mol^−1^. This confirms the experimental finding that in the presence of catalyst **8** no product inhibition takes place. Overall, the Gibbs free‐energy profile shows that intermediate **II** is the resting state of the catalytic cycle. Interestingly, the ring‐opening and ring‐closing steps have very similar effective Gibbs energy barriers of 71 and 72 kJ mol^−1^, respectively, indicating that both steps can be rate‐determining.

For catalyst **7**, the Gibbs free‐energy profile (Figure S22) shows that the interaction of epoxide with phenol‐derived phosphonium to form intermediate **II_Br_** through the H‐bond is endergonic by 27 kJ mol^−1^. The large energy difference indicates dynamic equilibrium in favor of **I_Br_** and **1 a** (>99.99 %) rather than **II_Br_**. For the ring‐opening step, the β route is also more favorable than the α route. The energy barrier of ring‐opening via **TS1‐β_Br_** is 97 kJ mol^−1^, which is 5 kJ mol^−1^ lower than that via **TS1‐α_Br_**. It is worth noting that the formation of **III‐β_Br_** is highly exergonic by 22 kJ mol^−1^, while the formation of **III‐β_I_** is slightly endergonic by 6 kJ mol^−1^. This indicates that the reaction with both the Br^−^ catalyst **7** and the I^−^ catalyst **8** can compensate the increase of energy caused by ring‐opening, and the formation of 1‐bromobutan‐2‐ol is more thermodynamically preferred. The following C−O coupling via **TS2‐β_Br_** has an energy barrier of 51 kJ mol^−1^. For the last step of ring‐closing, the effective energy barrier via **TS3‐β_Br_(O_b_)** from **III‐β_Br_** is 86 kJ mol^−1^. In contrast to catalyst **8**, the rate‐determining step for the reaction in the presence of catalyst **7** is clearly the ring‐opening. The effective barrier regarding this step for the bromide salt **7** is 97 kJ mol^−1^, which is higher than that for iodide salt **8** (72 kJ mol^−1^) by 25 kJ mol^−1^. This can reasonably explain the superior catalytic activity of **8** compared to catalyst **7**.

## Conclusions

Bifunctional phenolic phosphonium salt catalysts showed superior efficiency in converting epoxides and CO_2_ into value‐added cyclic carbonates under mild and solvent‐free conditions. The phenol‐based phosphonium iodide **8** proved to be the most active catalyst. Notably, this catalyst showed high activity even at room temperature. Kinetic investigations revealed that the superior activity in comparison with the previously reported aliphatic catalyst **3** originates from a higher intrinsic activity as well as the absence of any product inhibition. In the model reaction the equilibrium constant *K*_inh_ of the product inhibition with the aliphatic catalyst was calculated as 0.260 L mol^−1^. Moreover, the observable rate constants *k* ^obs^ were determined as 0.197 h^−1^ (**8**) >0.0605 h^−1^ (**3**), reflecting the catalytic activity. An apparent activation energy with a value of *E*_a_=39.6 kJ mol^−1^ was estimated for the benchmark reaction with catalyst **8**. The substrate scope was evaluated, and 20 terminal and 6 demanding internal epoxides were converted. Excellent results were achieved at room temperature for terminal epoxides while the conversion of internal epoxides required elevated reaction temperatures. Notably, catalyst **8** showed high functional group tolerance, and the desired cyclic carbonates were isolated in yields up to 99 % even on multi‐gram scale (up to 50 g substrate). Furthermore, this method was applied in the three‐step synthesis of antitussive agent dropropizine starting from epichlorohydrin and phenylpiperazine. On the basis of the experimental findings the full Gibbs free‐energy surfaces for the use of phenol‐derived phosphonium halides **7** and **8** as catalysts in the model reaction were calculated. The calculation confirmed the activation of the epoxide via hydrogen bonding for the iodide salt, which facilitates the ring‐opening step. Notably, the effective barrier regarding this step is 97 kJ mol^−1^ for the bromide and 72 kJ mol^−1^ for the iodide salt, which clearly explains the difference in activity. The DFT calculations also confirmed the experimental finding that no product inhibition occurs in the presence of the phenol‐based phosphonium iodide. Interestingly, for catalyst **8** the ring‐opening and ring‐closing steps have similar effective Gibbs energy barriers of 71 and 72 kJ mol^−1^, respectively, indicating that both steps can be rate‐determining. In contrast, for catalyst **7** the rate‐determining step is the epoxide opening with an effective Gibbs energy barrier of 97 kJ mol^−1^.

## Experimental Section

### Synthesis of catalyst 8

2‐(Diphenylphosphanyl)phenol:^[52]^ Under argon a mixture of 2‐iodophenol (660 mg, 3.00 mmol, 1.00 equiv.), Pd(OAc)_2_ (6.7 mg, 0.029 mmol, 0.03 equiv.), and NaOAc (271 mg, 3.30 mmol, 1.10 equiv.) was dissolved in anhydrous dimethylacetamide (9.00 mL). After addition of diphenylphosphine (559 mg, 3.00 mmol, 1.00 equiv.) the reaction mixture was heated to 110 °C and stirred for 17 h. Subsequently, the reaction mixture was cooled to 23 °C and filtered over celite using CH_2_Cl_2_ as eluent. After removal of all volatiles under vacuum the crude product was purified by column chromatography (SiO_2_, CH_2_Cl_2_) to yield the title compound (710 mg, 2.55 mmol, 83 %) as a colorless solid. ^1^H NMR (300 MHz, CDCl_3,_ 25 °C): *δ*=6.26–6.28 (br s, 1H), 6.88–7.03 (m, 3H), 7.29–7.39 (m, 11H) ppm. ^31^P NMR (122 MHz, CDCl_3,_ 25 °C): *δ*=−28.62 ppm.

(2‐Hydroxyphenyl)diphenyl(propyl)phosphonium iodide (**8**):^[32]^ In a pressure tube 1‐iodopropane (2.17 g, 12.8 mmol) was added to 2‐(diphenylphosphanyl)phenol (510 mg, 2.55 mmol). The tube was flushed with argon and sealed. Subsequently, the reaction mixture was stirred at 102 °C for 24 h. The crude product was filtered off and washed with Et_2_O (4×50 mL) to yield **8** (1.06 g, 2.37 mmol, 93 %) as a white solid. ^1^H NMR (300 MHz, CDCl_3_): *δ*=1.14 (td, *J*=7.3, 1.9 Hz, 3H), 1.68–1.81(m, 2H), 3.08–3.18 (m, 2H), 6.84–6.92 (m, 1H), 6.94–7.01 (m, 1H), 7.53–7.69 (m, 9H), 7.75–7.82 (m, 2H), 7.96–8.01 (m, 1H), 10.78 (br s, 1H) ppm. ^31^P NMR (122 MHz, CDCl_3_) *δ*=23.49 ppm.

### Representative examples for the synthesis of cyclic carbonates

4‐Methyl‐1,3‐dioxalan‐2‐one (**2 a**):^[53]^ A 45 cm^3^ stainless‐steel autoclave was charged with catalyst **8** (306 mg, 0.683 mmol, 5 mol %) and 1,2‐epoxybutane (**1 a**, 1.00 g, 13.9 mmol). The autoclave was purged with CO_2_. Subsequently, the reaction mixture was stirred at 23 °C for 24 while *p*(CO_2_) was kept constant at 1.00 MPa. CO_2_ was released slowly, and the reaction mixture was filtered over SiO_2_ with EtOAc as eluent. After the removal of all volatiles under vacuum **2 a** (1.53 g, 13.1 mmol, 95 %) was obtained as a light‐yellow oil. ^1^H NMR (300 MHz, CDCl_3_) *δ*=1.03 (t, *J*=7.4 Hz, 3H), 1.66–1.93 (m, 2H), 4.09 (dd, *J*=8.4, 7.0 Hz, 1H), 4.53 (dd, *J*=8.4, 7.9 Hz, 1H), 4.61–4.74 (m, 1H) ppm.

4‐(Methoxymethyl)‐1,3‐dioxalan‐2‐one (**12 a**):^[53]^ A 45 cm^3^ stainless‐steel autoclave was charged with 2‐(methoxymethyl)oxirane (**11 a**, 1.00 g, 11.4 mmol) and catalyst **8** (254 mg, 0.567 mmol, 5 mol %). The autoclave was purged with CO_2_. Subsequently, the reaction mixture was stirred at 23 °C for 48 h while *p*(CO_2_) was kept constant at 1.00 MPa. CO_2_ was released slowly, and the reaction mixture was filtered over SiO_2_ with EtOAc as eluent. After the removal of all volatiles under vacuum **12 a** (1.44 g, 10.9 mmol, 96 %) was obtained as a yellow liquid. ^1^H NMR (300 MHz, CDCl_3_) *δ*=3.43 (s, 3H), 3.58 (dd, *J*=10.9, 3.8 Hz, 1H), 3.63 (dd, *J*=3.9 Hz, 1H), 4.39 (dd, *J*=8.4, 6.1 Hz, 1H), 4.50 (t, *J*=8.3 Hz, 1H), 4.75–4.87 (m, 1H) ppm.

*cis*‐Tetrahydrofuro[3,4‐d][1,3]dioxol‐2‐one (**14 c**):^[53]^ A 45 cm^3^ stainless‐steel autoclave was charged with 6‐dioxabicyclo[3.1.0]hexane (**13 c**, 1.00 g, 11.6 mmol) and catalyst **8** (261 mg, 0.582 mmol, 5 mol %). The autoclave was purged with CO_2_. Subsequently the mixture was stirred at 80 °C for 24 h, while *p*(CO_2_) was kept constant at 2.50 MPa. The reactor was cooled with an ice bath below 20 °C and CO_2_ was released slowly. The reaction mixture was filtered over SiO_2_ with EtOAc as eluent. After the removal of all volatiles under vacuum the desired products **14 c** (1.25 g, 9.61 mmol, 82 %) was obtained as a colorless liquid.

## Conflict of interest

The authors declare no conflict of interest.

## Supporting information

As a service to our authors and readers, this journal provides supporting information supplied by the authors. Such materials are peer reviewed and may be re‐organized for online delivery, but are not copy‐edited or typeset. Technical support issues arising from supporting information (other than missing files) should be addressed to the authors.

Supplementary
