# Supplementary material for: Catalytic, Kinetic, and Mechanistic Insights into the Fixation of CO2 with Epoxides Catalyzed by Phenol‐Functionalized Phosphonium Salts
Source: ChemSusChem. 2020 Nov 13;14(1):363–72. doi: 10.1002/cssc.202002267 (PMC7839512; doi:10.1002/cssc.202002267)
Supplement: Supplementary file 1 — Supplementary [file CSSC-14-363-s001.pdf]

ChemSusChem

Supporting Information

Catalytic, Kinetic, and Mechanistic Insights into the Fixation of CO_2 with Epoxides Catalyzed by Phenol-Functionalized Phosphonium Salts

Yuya Hu, Zhihong Wei, Anna Frey, Christoph Kubis, Chang-Yue Ren, Anke Spannenberg, Haijun Jiao, and Thomas Werner*© 2020 The Authors. ChemSusChem published by Wiley-VCH GmbH. This is an open access article under the terms of the Creative Commons Attribution License, which permits use, distribution and reproduction in any medium, provided the original work is properly cited.

Contents

1. General Considerations 1

2. Synthesis of phosphonium salt catalysts 1

3. General procedure for the catalyst screening and parameter optimization 4

4. Synthesis of cyclic carbonates 5

5. Synthesis of dropropizine..15

6. Putative S_N1 and S_N2 reaction pathways 21

7. Infrared spectroscopic investigations...22

8. Control experiments with catalyst **3** and **8** at room temperature 30

9 Kinetic investigations 32

9.1 Kinetic evaluation of yield vs. time data for catalysts **3** and **8**.................................... 32

9.1.1 Kinetic evaluation of the yield vs. time data for the [*n*$Bu_3P(CH_2)_2OH$]I (**3**) catalyst...... 35

9.1.2 Kinetic evaluation of the yield vs. time data for the [*n*$PrP(Ph)_2PhOH$]I (**8**) catalyst...... 36

9.2 The yield vs. time data of 1,2-butylene carbonate formation at various temperatures. 38

9.3 Calculation of the activation energy from an Arrhenius-plot...................................... 39

9.4 Calculation of the enthalpy and entropy of activation.. 40

10 DFT calculation 42

10.1 Catalytic cycle in both the C_α and C_β pathways 43

10.2 Possibilities of the intermediates V and the transition states TS3 in the C_α pathway... 44

10.3 Possibilities of the intermediates V and the transition states TS3 in the C_β pathway... 45

10.4 Optimized structures for the transition states 46

10.5 Gibbs free energy for the α and β routes with catalyst 7 50

10.6 Computational details.. 50

11 Crystallographic Data..76

12. NMR-spectra of catalysts and synthesized carbonates 77

13 HPLC-spectra of chiral carbonates...109

14 References...122

1. General Considerations

All chemicals were purchased from commercial sources (Sigma Aldrich, TCI, Alfa Aesar or abcr) with purities ≥95% and used without further purification. Deuterated solvents were ordered from *Deutero GmbH* and stored over molecular sieves. NMR spectra were received using *Bruker* 300 Fourier, *Bruker* AV 300 and *Bruker* AV 400 spectrometers. Chemical shifts are reported in ppm relative to the deuterated solvent. Coupling constants are expressed in Hertz (Hz). The following abbreviations are used: s= singlet, d= doublet, t= triplet and m= multiplet. NMR yields were determined by using mesitylene as internal standard. Gas chromatography was performed on *Agilent 7890A GC System*, mass spectra were measured on downstream *5975C inert XL MSD* mass detector also from *Agilent*. Elementary analysis was performed on a TruSpec CHMS Micro from *Leco*. IR spectra were recorded on a Nicolet iS10 MIR FT-IR-spectrometer from *Thermo Fisher Scientific*. Thin layer chromatography was performed on *Merck* TLC-plates with fluorescence indication (silica type 60, F_{254}), spots were visualized using UV-light or potassium permanganate. Flash chromatography was performed using silica with a grain size of 40–63 µm from *Macherey-Nagel*. For chiral HPLC-analysis a device Agilent 1100 Series was used.

2. Synthesis of phosphonium salt catalysts

Tri-*n*-butyl(2-hydroxyethyl)phosphonium iodide (3)[1]

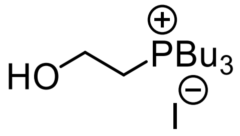

In a pressure tube, tri-*n*-butylphosphine (4.46 g, 22.0 mmol) and 2-iodoethanol (3.73 g, 22.0 mmol) were dissolved in toluene (22 mL). The tube was sealed, and the mixture stirred at 60 °C for 24 h under argon atmosphere. Then the reaction mixture was cooled to room temperature and all volatiles were removed in vacuo. The desired product **3** (8.15 g, 21.78 mmol, 99 %) was obtained as a colorless solid. 1H-NMR (300 MHz, $CDCl_3$) δ = 0.97 (t, 9H), 1.49–1.62 (m, 12H), 2.34–2.40 (m, 6H), 2.67–2.72 (m, 2H), 3.80 (br. s, 1H, OH) 4.03–4.11 (m, 2H) ppm. ^{31}P NMR (122 MHz, $CDCl_3$, 25 °C): δ= 33.29 ppm.

(2-Carboxyethyl)triphenylphosphonium bromide (4)[2]

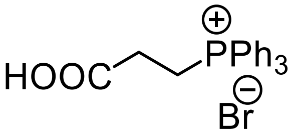

In a pressure tube, Ph_3P (262 mg, 1.00 mmol) was added to a solution of 3-bromopropionic acid (153 mg, 1.00 mmol) in toluene (4.00 mL). The tube was flushed with argon and sealed. Subsequently, the reaction mixture was stirred at 110 °C for 24 h. After cooling to room temperature, the resulting crude solid was filtered off, and the solid was washed with EtOAc (6×50 mL), then Et_2O (3×20 mL) to yield **5** (369 mg, 0.890 mmol, 89 %) as a colorless solid. 1H-NMR (300 MHz, $CDCl_3$) δ= 2.96–3.05 (m, 2H), 3.69–3.79 (m, 2H), 7.65–7.81 (m, 15H), 10.19 (br. s, 1H, OH) ppm. ^{31}P NMR (122 MHz, $CDCl_3$, 25 °C): δ= 24.68 ppm.

(2-Hydroxy-5-methylphenyl)triphenylphosphonium bromide (6)[3]

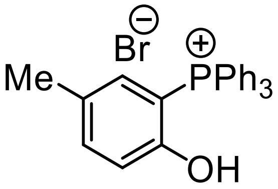

Under argon a mixture of Ph_3P (1.13 g, 4.29 mmol), 2-bromo-4-methylphenol (535 mg, 2.86 mmol), and Pd_2dba_3 (27.5 mg, 300 µmol) was dissolved in ethylene glycol (0.95 mL). The reaction mixture was heated to 140 °C and stirred for 20 h. After cooling to room temperature, the reaction mixture was quenched with H_2O (10 mL) and extracted with CH_2Cl_2 (2×20 mL). The combined organic layers were washed with H_2O (3×20 mL) and dried over Na_2SO_4. After removal of all volatiles in vacuo the crude mixture was purified by flash column chromatography (SiO_2, CH_2Cl_2/MeOH= 10:1). The obtained oil was triturated with CH_2Cl_2/Et_2O (1/20) to give the product as a colorless solid (1.29 g, 2.37 mmol, 83 %). ^1H-NMR (300 MHz, $CDCl_3$) δ= 2.13 (s, 3H), 6.48–6.53 (dd, J= 14.7, 1.6 Hz, 2H), 7.28 (br. s, 1H, OH), 7.52–7.80 (m, 16H) ppm. ^{31}P NMR (122 MHz, $CDCl_3$, 25 °C): δ= 22.29 ppm.

2-(Diphenylphosphanyl)phenol[4]

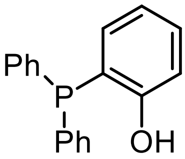

Under argon a mixture of 2-iodophenol (660 mg, 3.00 mmol, 1.00 equiv), $Pd(OAc)_2$ (6.7 mg, 0.029 mmol, 0.03 equiv), NaOAc (271 mg, 3.30 mmol, 1.10 equiv) was dissolved in anhydrous DMA (9.00 mL). After addition of diphenylphosphine (559 mg, 3.00 mmol, 1.00 equiv) the reaction mixture was heated to 110 °C and stirred for 17 h. Subsequently, the reaction mixture was cooled to 23 °C and filtered over celite using CH_2Cl_2 as eluent. After removal of all volatiles in vacuo the crude product was purified by column chromatography (SiO_2, CH_2Cl_2) to yield the title compound (710 mg, 2.55 mmol, 83%) as a colorless solid. ^{1}H NMR (300 MHz, $CDCl_3$, 25 °C): δ = 6.26–6.28 (br s, 1H), 6.88–7.03 (m, 3H), 7.29–7.39 (m, 11H) ppm. ^{31}P NMR (122 MHz, $CDCl_3$, 25 °C): δ = –28.62 ppm.

(2-Hydroxyphenyl)diphenyl(propyl)phosphonium bromide (7)[5]

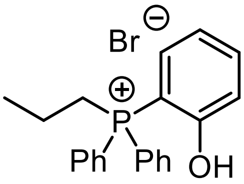

In a pressure tube 2-(diphenylphosphanyl)phenol (500 mg, 1.80 mmol) was added to 1-bromopropane (1.08 g, 9.00 mmol). The tube was flushed with argon and sealed. Subsequently, the reaction mixture was stirred at 70 °C for 24 h. The crude product was filtered off and washed with Et_2O (4×50 mL) to yield **7** (670 mg, 1.67 mmol, 93%) as a colourless solid. ^{1}H NMR (300 MHz, $CDCl_3$, 25 °C) δ = 1.11 (t, *J* = 7.3 Hz, 3H), 1.55–1.85 (m, 2H), 3.12 (m, 1H), 6.7–7.04 (m, 2H), 7.48–7.67 (m, 10H), 7.71–7.82 (m, 2H), 8.01–8.14 (m, 1H), 11.11 (s, 1H) ppm. ^{31}P NMR (122 MHz, $CDCl_3$, 25 °C): δ = 23.45 ppm.

(2-Hydroxyphenyl)diphenyl(propyl)phosphonium iodide (8)[5]

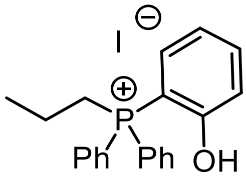

In a pressure tube 1-iodopropane (2.17 g, 12.8 mmol) was added to 2-(diphenylphosphanyl)phenol (510 mg, 2.55 mmol). The tube was flushed with argon and sealed. Subsequently, the reaction mixture was stirred at 102 °C for 24 h. The crude product was filtered off and washed with Et_2O (4×50 mL) to yield **8** (1.06 g, 2.37 mmol, 93%) as a colourless solid. ^1H NMR (300 MHz, $CDCl_3$): δ= 1.14 (td, J= 7.3, 1.9 Hz, 3H), 1.68–1.81(m, 2H), 3.08–3.18 (m, 2H), 6.84–6.92 (m, 1H), 6.94–7.01 (m, 1H), 7.53–7.69 (m, 9H), 7.75–7.82 (m, 2H), 7.96–8.01 (m, 1H), 10.78 (br s, 1H) ppm. ^{31}P NMR (122 MHz, $CDCl_3$) δ= 23.49 ppm.

3. General procedure for the catalyst screening and parameter optimization

A 45 cm^3 stainless-steel autoclave was charged with the catalyst (2 or 5 mol%). Subsequently, 1,2-butylene oxide (**1a**, 1.00 g, 13.9 mmol, 1.00 equiv) was added. The autoclave was purged with CO_2 and was kept at 23 °C or heated to 90 °C for 2–48 h, while $p(CO_2)$ was kept constant at 1.00 MPa (for reaction with 1 atm CO_2: a 50 cm^3 Schlenk tube was charged with the catalyst and 1,2-butylene oxide and purged with CO_2. Subsequently, CO_2 was slowly bubbled through the reaction mixture for 24 h). Afterwards the reactor was cooled with an ice bath below 20 °C and CO_2 was released slowly. The conversion of the epoxide **1a** and yield of the carbonate **2a** were determined by ^1H NMR spectroscopy from the reaction mixture using mesitylene as internal standard.

4. Synthesis of cyclic carbonates

General procedure for the coupling reaction of CO_2 and terminal epoxides and glycidyl ether derivatives (GP1)

A 45 cm^3 stainless-steel autoclave was charged with (2-hydroxyphenyl)-diphenyl(propyl)phosphonium iodide (**8**, 5 mol%) and epoxide **1** or **11** (1.00 equiv). The autoclave was purged with CO_2. The reaction mixture was stirred at 23 °C for 24 (for substrates **1**) or 48 h (for substrates **11**), while *p*(CO_2) was kept constant at 1.00 MPa. Afterwards the reactor was cooled with an ice bath below 20 °C and CO_2 was released slowly. The reaction mixture was filtered over silica (SiO_2) with EtOAc as eluent. After the removal of all volatiles in vacuo the desired products **2** or **12** were obtained without further purification.

4-Ethyl-1,3-dioxalan-2-one (2a)[6]

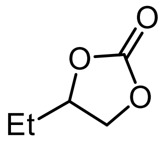

According to **GP1**, 1,2-epoxybutane (**1a**, 1.00 g, 13.9 mmol), **8** (306 mg, 0.683 mmol) and CO_2 were converted to yield **2a** (1.53 g, 13.1 mmol, 95%) as a light yellow oil.

^1H NMR (300 MHz, $CDCl_3$) δ= 1.03 (t, *J*= 7.4 Hz, 3H), 1.66–1.93 (m, 2H), 4.09 (dd, *J*= 8.4, 7.0 Hz, 1H), 4.53 (dd, *J*= 8.4, 7.9 Hz, 1H), 4.61–4.74 (m, 1H) ppm.

4-Methyl-1,3-dioxalan-2-one (2b)[6]

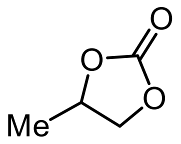

According to **GP1**, 1,2-epoxypropane (**1b**, 1.00 g, 17.2 mmol), **8** (385 mg, 0.859 mmol) and CO_2 were converted to yield **2b** (1.65 g, 16.2 mmol, 94%) as a light yellow liquid.

^1H NMR (300 MHz, $CDCl_3$) δ= 1.49 (t, *J*= 6.3 Hz, 3H), 4.03 (dd, *J*= 8.4, 7.2 Hz, 1H), 4.55 (dd, *J*= 8.4, 7.7 Hz, 1H), 4.74–4.98 (m, 1H) ppm.

(*S*)-4-Methyl-1,3-dioxalan-2-one (*S*-2b)[6]

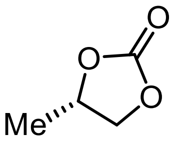

According to **GP1**, (*S*)-1,2-epoxypropane (*S*-**1b**, 1.00 g, 17.2 mmol), **8** (380 mg, 0.848 mmol) and CO_2 were converted to yield *S*-**2b** (1.65 g, 16.2 mmol, 94%, >99% *ee*) as a light yellow liquid.

The enantiomeric excess was determined on a Hydrodex, β-TBDAc column (50 m), 190 °C, isotherm, heptane/EtOH= 80:20 v/v, *v*= 1.0 mL/min, t_r (major)= 10.291 min, t_r (minor)= 11.928 min, >99% *ee*.

4-Butyl-1,3-dioxolan-2-one (2c)[6]

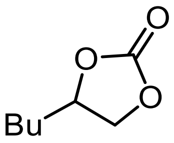

According to **GP1**, 2-butyloxirane (**1c**, 1.02g, 10.2 mmol), **8** (224 mg, 0.499 mmol) were dissolved in *n*-butanol (1.00 mL) and converted with CO_2 to yield **2c** (1.40 g, 9.69 mmol, 95%) as a yellow oil.

^{1}H NMR (300 MHz, $CDCl_3$) δ= 0.89 (t, 3H), 1.28–1.34 (m, 4H), 1.61–1.86 (m, 2H), 4.07 (dd, *J*= 8.4, 7.2 Hz, 1H), 4.53 (dd, *J*= 8.3, 7.8 Hz, 1H), 4.65–4.76 (m, 1H) ppm.

4-Hexyl-1,3-dioxolan-2-one (2d) [6]

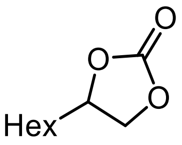

According to **GP1**, 2-hexyloxirane (**1d**, 1.31g, 7.03 mmol), **8** (158 mg, 0.352 mmol) and CO_2 were converted in *n*-butanol (1.00 mL). After purification with column chromatography (SiO_2, cHex : EtOAc= 5:1) **2d** (936 mg, 5.02 mmol, 72%) was isolated as a yellow liquid.

^{1}H NMR (300 MHz, $CDCl_3$) δ= 0.84–0.95 (m, 3H), 1.23–1.52 (m, 8H), 1.60–1.88 (m, 2H), 4.07 (dd, J= 8.4, 7.2 Hz, 1H), 4.52 (dd, J= 8.3, 7.8 Hz, 1H), 4.62–4.79 (m, 1H) ppm.

4-Phenyl-1,3-dioxalan-2-one (2e) [6]

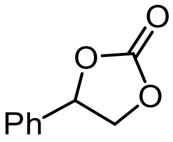

According to **GP1**, 2-phenyloxirane (**1e**,1.00 g, 8.33 mmol), **8** (311 mg, 0.694 mmol) and CO_2 were converted. After purification *via* column chromatography (SiO_2, *c*Hex:EtOAc= 5:1) the product **2e** (1.01 g, 6.16 mmol, 74%) was obtained as a yellow solid.

^{1}H NMR (300 MHz, $CDCl_3$) δ= 4.24–4.46 (m, 1H), 4.81 (dd, J= 8.6, 8.2 Hz, 1H), 5.56–5.82 (m, 1H), 7.34–7.41 (m, 2H), 7.41–7.51 (m, 3H) ppm.

(*R*)-4-Phenyl-1,3-dioxalan-2-one (*R*-2e) [6]

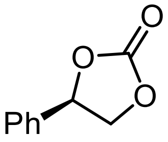

According to **GP1**, 2-phenyloxirane (*R*-**1e**,1.00 g, 8.33 mmol), **8** (311 mg, 0.694 mmol) and CO_2 were converted. After purification *via* column chromatography (SiO_2, *c*Hex:EtOAc= 5:1) the product *R*-**2e** (1.01 g, 6.15 mmol, 74%, 73% *ee*) was obtained as a colorless solid.

The enantiomeric excess was determined on a Hydrodex, β-TBDAc (50 m), 190 °C, isotherm, heptane/EtOH= 80:20 v/v, v= 1.0 mL/min, t_r (minor)= 15.216 min, t_r (major)= 15.615 min, 83% *ee*.

4-(Chloromethyl)-1,3-dioxalan-2-one (2f)

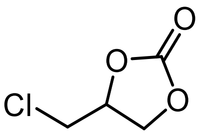

According to **GP1**, epichlorohydrin (**1f**, 1.00 g, 10.9 mmol), **8** (244 mg, 0.544 mmol) and CO_2 were converted to yield **2f** (1.41 g, 10.4 mmol, 95%) as a light yellow liquid.

^{1}H NMR (300 MHz, $CDCl_3$) δ= 3.63–3.87 (m, 2H), 4.42 (dd, *J*= 8.9, 5.8 Hz, 1H), 4.60 (dd, *J*= 8.9, 8.2 Hz, 1H), 4.84–5.08 (m, 1H) ppm.

4-(Morpholinomethyl)-1,3-dioxolan-2-one (2g)[7]

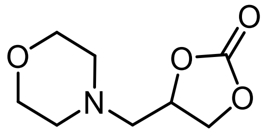

According to **GP1**, 4-(oxiran-2-ylmethyl)morpholine (**1g**, 1.00 g, 6.98 mmol), **8** (157 mg, 0.350 mmol) and CO_2 were converted. After purification *via* column chromatography (SiO_2, *c*Hex:EtOAc= 5:1) the product **2g** (940 mg, 5.02 mmol, 73%) was obtained as a yellow liquid.

^{1}H NMR (300 MHz, $CDCl_3$) δ= 2.56 (q, *J*= 4.1 Hz, 4H), 2.69 (dd, *J*= 5.4, 1.0 Hz, 2H), 3.69 (t, *J*= 4.6 Hz, 4H), 4.24 (dd, *J*= 8.5, 7.0 Hz, 1H), 4.48–4.58 (m, 1H), 4.77–4.91 (m, 1H) ppm.

4,4-Dimethyl-1,3-dioxalan-2-one (2h) [6]

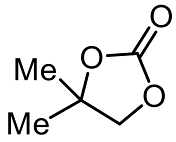

According to **GP1**, 2,2-dimethyloxiran (**1h**) (1.00 g, 13.9 mmol), **8** (311 mg, 0.694 mmol) and CO_2 (*p*= 2.5 MPa) were converted at 80 °C. The product **2h** (1.19 g, 10.2 mmol, 86%) was isolated as a yellow liquid.

^{1}H NMR (400 MHz, $CDCl_3$) δ= 1.53 (s, 6H), 4.15 (s, 2H) ppm.

4-(Chloromethyl)-4-methyl-1,3-dioxolan-2-one (2i) [6]

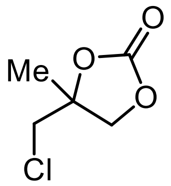

According to **GP1**, 2-(chloromethyl)-2-methyloxirane (**1i**, 1.00 g, 9.39 mmol), **8** (210 mg, 0.468 mmol) were converted at 80 °C and $p(CO_2)$= 2.5 MPa to yield **2i** (1.15 g, 8.00 mmol, 85%) as a yellow liquid.

^{1}H NMR (400 MHz, $CDCl_3$) δ= 1.64 (s, 3H), 3.60 (d, *J*= 11.9 Hz, 1H), 3.73 (d, *J*= 11.9 Hz, 1H), 4.16 (d, *J*= 8.7 Hz, 1H), 4.52 (d, *J*= 8.8 Hz, 1H) ppm.

4-(Methoxymethyl)-1,3-dioxalan-2-one (12a)[6]

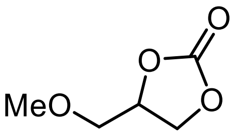

According to **GP1**, 2-(methoxymethyl)oxirane (**11a**, 1.00 g, 11.4 mmol), **8** (254 mg, 0.567 mmol) and CO_2 were converted to yield **12a** (1.44 g, 10.9 mmol, 96%) as a yellow liquid.

^{1}H NMR (300 MHz, $CDCl_3$) δ= 3.43 (s, 3H), 3.58 (dd, *J*= 10.9, 3.8 Hz, 1H), 3.63 (dd, *J*= 3.9 Hz, 1H), 4.39 (dd, *J*= 8.4, 6.1 Hz, 1H), 4.50 (t, *J*= 8.3 Hz, 1H), 4.75–4.87 (m, 1H) ppm.

4-(*tert*-Butoxymethyl)-1,2-dioxalan-2-one (12b)[6]

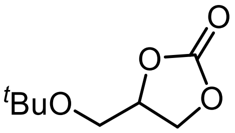

According to **GP1**, 2-(tert-butoxymethyl)oxirane (**11b**, 1.00 g, 7.68 mmol), **8** (172 mg, 0.384 mmol) and CO_2 were converted to yield **12b** (1.30 g, 7.47 mmol, 97%) as a colorless liquid.

^{1}H NMR (300 MHz, $CDCl_3$) δ= 1.20 (s, 9H), 3.53 (dd, *J*= 10.3, 3.6 Hz, 1H), 3.62 (dd, *J*= 10.3, 4.6 Hz, 1H), 4.39 (dd, *J*= 8.3, 5.9 Hz, 1H), 4.48 (t, *J*= 8.2 Hz, 1H), 4.77 (m, 1H) ppm.

4-(Hydroxymethyl)-1,3-dioxalan-2-one (12c)[6]

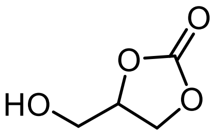

According to **GP1**, oxiran-2-ylmethanol (**11c**, 1.00 g, 13.5 mmol), **8** (297 mg, 0.663 mmol) and CO_2 were converted to yield **12c** (1.30 g, 11.0 mmol, 82%) as a colorless oil.

^{1}H NMR (300 MHz, $CDCl_3$) δ= 2.35 (s, 1H), 3.72 (dd, *J*= 12.8, 3.5 Hz, 1H), 4.00 (dd, *J*= 12.8, 3.0 Hz, 1H), 4.41–4.59 (m, 2H), 4.77–4.89 (m, 1H) ppm.

4-((2,2,3,3-Tetraflouropropoxy)methyl-1,3-dioxalan-2-one (12d) [6]

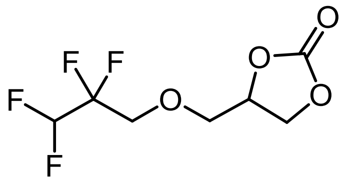

According to **GP1**, 2-((2,2,3,3-tetrafluoropropoxy)methyl)oxirane (**11d**, 1.00 g, 5.32 mmol), **8** (119 mg, 0.265 mmol) and CO_2 were converted to yield **12d** (1.04 g, 4.48 mmol, 86%) as a colorless liquid.

^{1}H NMR (300 MHz, $CDCl_3$) δ= 3.76–4.04 (m, 4H), 4.39 (dd, *J*= 8.5, 6.0 Hz, 1H), 4.54 (t, *J*= 8.5 Hz, 1H), 4.81–4.90 (m, 1H), 5.88 (tt, *J*= 53.1, 4.4 Hz, 1H) ppm.

4-(((2,2,3,3,4,4,5,5-Octafluoropentyl)oxy)methyl)-1,3-dioxalan-2-one (12e) [6]

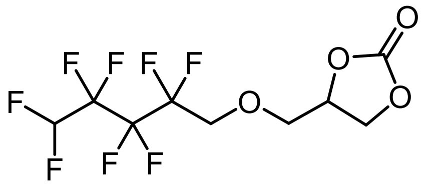

According to **GP1**, 4-(((2,2,3,3,4,4,5,5-octafluoropentyl)oxy)methyloxirane (**11e**, 1.00 g, 3.47 mmol) **8** (77.8 mg, 0.174 mmol) and CO_2 were converted to yield **12e** (910 mg, 2.74 mmol, 79%) as a colorless liquid.

^{1}H NMR (300 MHz, $CDCl_3$) δ= 3.82–3.94 (m, 2H), 3.95–4.23 (m, 2H), 4.41 (dd, *J*= 8.5, 6.1 Hz, 1H), 4.54 (t, *J*= 8.5 Hz, 1H), 4.76–4.90 (m, 1H), 6.05 (tt, *J*= 51.9, 5.4 Hz, 1H) ppm.

2-(Oxo-1,3-dioxalan-4-yl)methyl methacrylate (12f) [6]

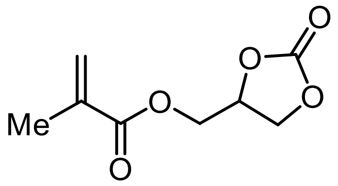

According to **GP1**, oxiran-2-ylmethyl methacrylate (**11f**, 1.00 g, 7.03 mmol), **8** (157 mg, 0.350mmol) and CO_2 were converted to yield **12f** (1.27 g, 6.99 mmol, 99%) as a colorless liquid.

^{1}H NMR (300 MHz, $CDCl_3$) δ= 1.95–1.96 (d, 3H), 4.31–4.37 (d, *J*= 20.1 Hz, 2H), 4.45 dd, *J*= 12.6 Hz, 1H), 4.56–4.62 (t, *J*= 8.6 Hz, 1H), 4.95–5.02 (m, 1H), 5.65–5.67 (d, *J*= 4.5 Hz, 1H), 6.16 (d, *J*= 2.7 Hz, 1H) ppm.

4-((Allyloxy)methyl)-1,3-dioxolan-2-one (12g)[6]

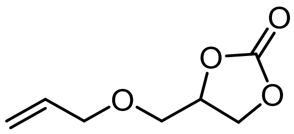

According to **GP1**, 2-((allyloxy)methyl)oxirane (**11g**, 1.00 g, 6.49 mmol), **8** (145 mg, 0.323 mmol) and CO_2 were converted to yield **12g** (1.35 g, 8.54 mmol, 97%) as a light yellow liquid.

^{1}H NMR (300 MHz, $CDCl_3$) δ= 3.62 (dd, *J*= 11.0, 3.8 Hz, 1H), 3.70 (dd, *J*= 11.0, 4.0 Hz, 1H), 4.00–4.13 (m, 2H), 4.40 (dd, *J*= 8.4, 6.1 Hz, 1H), 4.46–4.55 (m, 1H), 4.77–4.89 (m, 1H), 5.18–5.35 (m, 2H), 5.80–5.95 (m, 1H) ppm.

4-((Furan-2-ylmethoxy)methyl)-1,3-dioxolan-2-one (12h)[6]

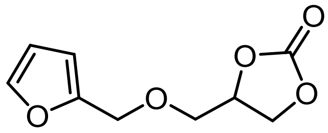

According to **GP1**, 2-((oxiran-2-ylmethoxy)methyl)furan (**11h**, 1.00 g, 6.49 mmol), **8** (145 mg, 0.323 mmol) and CO_2 were converted to yield **12h** (1.25 g, 6.29 mmol, 97%) as a yellow liquid.

^{1}H NMR (300 MHz, $CDCl_3$) δ= 3.64 (dd, *J*= 10.9, 3.9 Hz, 1H), 3.71 (dd, *J*= 10.9, 4.3 Hz, 1H), 4.34 (dd, *J*= 8.4, 6.2 Hz, 1H), 4.43–4.61 (m, 3H), 4.79 (m, 1H), 6.32–6.41 (m, 2H), 7.40–7.47 (m, 1H) ppm.

4-((3-(Triethoxysilyl)propoxy)methyl)-1,3-dioxolan-2-one (12i) [8]

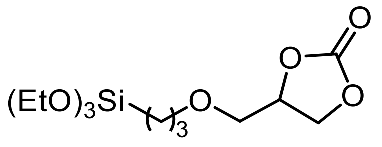

According to **GP1**, (3-glycidoxypropyl)triethoxysilane (**11i**, 1.00 g, 3.59 mmol), **8** (32 mg, 71.3 µmol) and CO_2 were converted at 90 °C for 4 h to yield **12i** (1.10 g, 3.32 mmol, 93%) as colorless liquid.

^{1}H NMR (300 MHz, $CDCl_3$) δ= 0.60–0.69 (m, 2H), 1.23 (t, *J*= 7.0 Hz, 9H), 1.63–1.76 (m, 2H), 3.49 (td, *J*= 6.7, 1.2 Hz, 2H), 3.61 (dd, *J*= 10.9, 3.8 Hz, 1H), 3.68 (dd, *J*= 10.9, 4.2 Hz, 1H), 3.82 (q, *J*= 7.0 Hz, 6H), 4.40 (dd, *J*= 8.3, 6.2 Hz, 1H), 4.49 (dd, *J*= 8.3 Hz, 1H), 4.75–4.85 (m, 1H) ppm.

4,4'-(((Propane-2,2-diylbis(4,1-phenylene))bis(oxy))bis(methylene))bis(1,3-dioxolan-2-one) (12j)[6]

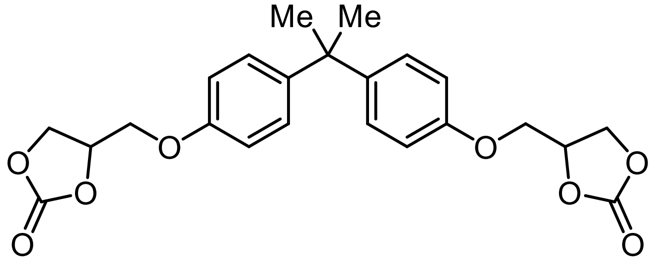

According to **GP1**, 2,2'-(((propane-2,2-diylbis(4,1-phenylene))bis(oxy))bis(methylene)) bis(oxirane) (**11j**, 1.00 g, 2.94 mmol), **8** (10 mol%, 132 mg, 0.294 mmol) and CO_2 were converted at 45 °C in *n*-butanol (1 mL) to yield **12j** (1.20 g, 2.80 mmol, 95%) as a colorless solid.

^{1}H NMR (400 MHz, $CDCl_3$) δ= 1.63 (s, 6H), 4.12 (dd, *J*= 10.6, 3.6 Hz, 2H), 4.21 (dd, *J*= 10.6, 4.3 Hz, 2H), 4.52 (dd, *J*= 8.5, 5.9 Hz, 2H), 4.60 (t, *J*= 8.4 Hz, 2H), 4.97–5.04 (m, 2H), 6.78–6.83 (m, 4H), 7.11–7.17 (m, 4H) ppm.

General procedure for the coupling reaction of CO_2 internal epoxides (GP2)

A 45 cm^3 stainless-steel autoclave was charged with (2-hydroxyphenyl)-diphenyl(propyl)phosphonium iodide (**8**, 5 mol%) and epoxide **13** (1.00 equiv). The autoclave was purged with CO_2 and was kept at 80 °C for 24 h, while *p*(CO_2) was kept constant at 2.50 MPa. Afterwards the reactor was cooled with an ice bath below 20 °C and CO_2 was released slowly. The reaction mixture was filtered over silica gel (SiO_2) with EtOAc as eluent. After the removal of all volatiles in vacuo the desired products **12** were obtained without further purification.

Hexahydrobenzo[*d*][1,3]dioxol-2-one (14a) [6]

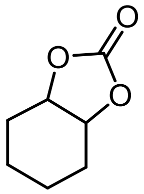

According to **GP2** 7-oxabicyclo[4.1.0]heptane (**13a**, 1.00 g, 10.2 mmol), **8** (228 mg, 0.508 mmol) and CO_2 were converted to yield **14a** (855 mg, 6.02 mmol, 59%) as a yellow solid.

^{1}H NMR (400 MHz, $CDCl_3$) δ= 1.34–1.51 (m, 2H), 1.52–1.73 (m, 2H), 1.75–2.02 (m, 4H), 4.62–4.76 (m, 2H) ppm.

Tetrahydro-4*H*-cyclopenta[*d*][1,3]dioxol-2-one (14b) [6]

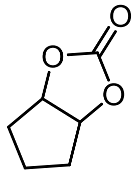

According to **GP2**, 6-oxabicyclo[3.1.0]hexane (**13b**, 1.00 g, 11.9 mmol), **8** (266 mg, 0.593 mmol) and CO_2 were converted to yield **14b** (1.10 g, 8.59 mmol, 72%) as a yellow solid.

^{1}H NMR (400 MHz, $CDCl_3$) δ= 1.62–1.85 (m, 4H), 2.09–2.22 (m, 2H), 5.01–5.19 (m, 2H) ppm.

Tetrahydrofuro[3,4-*d*][1,3]dioxol-2-one (14c)[6]

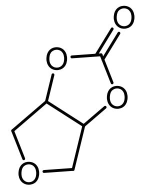

According to **GP2**, 3,6-dioxabicyclo[3.1.0]hexane (**13c**, 1.00 g, 11.6 mmol), **8** (261 mg, 0.582 mmol) and CO_2 were converted to yield **14c** (1.25 g, 9.61 mmol, 82%) as a colorless liquid.

^{1}H NMR (300 MHz, $CDCl_3$) δ = 3.49–3.65 (m, 2H), 4.20–4.30 (m, 2H), 5.18–5.24 (m, 2H) ppm.

4,5-Diphenyl-1,3-dioxalan-2-one (14d)[6]

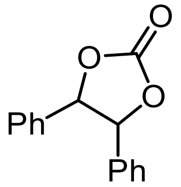

According to **GP2**, *cis*-2,3-diphenyloxiran (**13d**, 1.00 g, 5.09 mmol), **8** (114 mg, 0.254 mmol) were dissolved in *n*-butanol (1 mL) and converted with CO_2 to yield **14d** (424 mg, 1.77 mmol, 24%, *cis*/*trans* = 1:99) as a yellow solid.

^{1}H NMR (300 MHz, $CDCl_3$) δ = 5.45 (s, 2H), 7.30–7.36 (m, 4H), 7.42–7.48 (m, 6H) ppm.

4,5-Dimethyl-1,3-dioxalan-2-one (14e) [6]

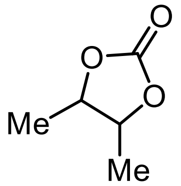

According to **GP2**, *cis*-2,3-dimethyloxiran (**13e**, 1.00 g, 13.9 mmol), **8** (311 mg, 0.694 mmol) and CO_2 were converted to yield **14e** (750 mg, 6.42 mmol, 84%, *cis*/*trans* = 25:75) as a yellow liquid.

cis-Isomer: ^{1}H NMR (400 MHz, $CDCl_3$) δ = 1.31–1.41 (m, 6H), 4.80–4.88 (m, 2H) ppm.
trans-Isomer: ^{1}H NMR (400 MHz, $CDCl_3$) δ = 1.41–1.51 (m, 6H), 4.28–4.40 (m, 2H) ppm.

Methyl 8-(5-octyl-2-oxo-1,3-dioxalan-4-yl)octanoate (14f)[9]

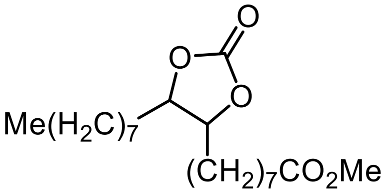

According to **GP2**, epoxidized *cis*-methyl oleate (**13f**, 1.00 g, 3.37 mmol), **8** (75.5 mg, 0.168 mmol) and CO_2 were converted in the desired carbonate. After purification *via* column chromatography (SiO_2, *c*Hex:EtOAc= 20:1) the product **14f** (690 mg, 1.92 mmol, 57%, *cis*/*trans*= 25:75) was obtained as a light yellow oil.

cis-isomer: ^{1}H NMR (300 MHz, $CDCl_3$) δ= 0.83–0.94 (m, 3H), 1.16–1.47 (m, 18H), 1.47–1.78 (m, 8H), 2.31 (t, *J*= 7.5 Hz, 2H), 3.67 (s, 3H), 4.58–4.67 (m, 2H) ppm.

trans-isomer: ^{1}H NMR (300 MHz, $CDCl_3$) δ= 0.83–0.94 (m, 3H), 1.16–1.47 (m, 18H), 1.47–1.78 (m, 8H), 2.31 (t, *J*= 7.5 Hz, 2H), 3.67 (s, 3H), 4.16–4.28 (m, 2H) ppm.

5. Synthesis of dropropizine

1-(Oxiran-2-ylmethyl)-4-phenylpiperazine (1j)[10]

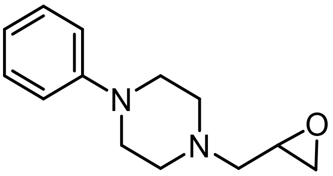

Epichlorohydrin (**1f**, 2.00 g, 21.6 mmol) was added dropwise to a mixture of 1-phenylpiperazin (**9**, 3.50 g, 21.6 mmol) and water (6.8 mL). The reaction mixture was stirred for 1 h at room temperature. Subsequently, aqueous NaOH (15.6 M, 16.2 mL) was added. The mixture was heated to 75 °C. After 15 min the mixture was cooled to room temperature and extracted with EtOAc (3×50 mL). The combined organic layers were dried over Na_2SO_4. Subsequently, all volatiles were removed in vacuo to yield **1j** (4.50 g, 20.6 mmol, 96%) as a colorless liquid.

^{1}H NMR (300 MHz, $CDCl_3$) δ= 2.34 (dd, J= 13.3, 6.9 Hz, 1H), 2.53 (dd, J= 5.0, 2.7 Hz, 1H), 2.63–2.73 (m, 2H), 2.74–2.89 (m, 4H), 3.12–3.20 (m, 1H), 3.25 (t, J= 5.1 Hz, 4H), 6.87 (tt, J= 7.3, 1.1 Hz, 1H), 6.92–6.99 (m, 2H), 7.24–7.33 (m, 2H) ppm.

^{13}C NMR (101 MHz, $CDCl_3$) δ= 44.84, 49.03, 50.27, 53.63, 61.00, 116.04, 119.73, 129.07, 151.21 ppm.

IR (ATR) Neat: 523, 690, 755, 927, 1009, 1140, 1228, 1302, 1332, 1451, 1496, 1598, 2758, 2815, 2938, 3038 cm^{-1}.

Elemental analysis calcd (%) for $C_{13}H_{18}N_2O$ (218.3 g mol^{-1}): C 71.53; H 8.31; N 12.83; found: C 71.33; H 8.40 N 12.47.

MS (EI, 70 eV): *m/z* (%)= 218 (85) [M^+], 217 (12), 203 (10), 201 (20), 176 (18), 175 (54), 173 (26), 162 (16), 161 (24), 160 (28), 147 (11), 134 (16), 133 (27), 132 (99), 120 (80), 119 (56), 118 (21), 117 (19), 112 (40), 106 (100), 105 (99), 104 (99), 98 (20), 96 (23), 91 (96), 87 (22), 84 (34), 82 (36), 79 (12), 78 (40), 77 (98), 71 (17), 70 (99), 68 (36), 65 (23), 63 (11), 58 (13), 57 (99), 56 (99), 55 (53), 54 (51), 52 (16), 51 (80), 50 (16), 44 (15), 43 (33), 42 (99), 41 (52), 39 (27), 31 (41), 30 (29), 29 (72).

(*R*)-(Oxiran-2-ylmethyl)-4-phenylpiperazine (*R*-1j)

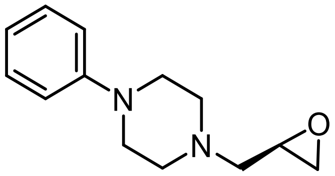

According to the synthesis of *rac*-**1j**, (*S*)-epichlorohydrin (*S*-**1f** 2.00 g, 21.6 mmol) were converted with 1-phenylpiperazin (**9**, 3.50 g, 21.6 mmol) in 6.80 mL water. After work-up the title compound ***R*-1j** (4.45 g, 20.3 mmol, 94%, 97% *ee*) was obtained as a colorless liquid.

The enantiomeric excess was determined by Amylose 2 column, heptane/EtOH= 98:2 v/v, 1.0 mL/min, t_r (minor)= 7.601 min, t_r (major) =9.778 min.

(*S*)-(Oxiran-2-ylmethyl)-4-phenylpiperazine (*S*-1j)

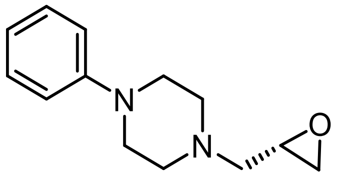

According to the synthesis of *rac*-**1j**, (*R*)-epichlorohydrin (*R*-**1f** 2.00 g, 21.6 mmol) and 1-phenylpiperazine (**9**, 3.50 g, 21.6 mmol) were converted in and 6.80 mL water. After the work-up the title compound *S*-**1j** (4.50 g, 20.6 mmol, 95%, 97% *ee*) was obtained as a colorless liquid.

The enantiomeric excess was determined by Amylose 2 column, heptane/EtOH= 98:2 v/v, 1.0 mL/min, t_r (minor)= 7.245 min, t_r (major) =9.882 min.

4-((4-Phenylpiperazin-1-yl)methyl)-1,3-dioxolan-2-one (2j)

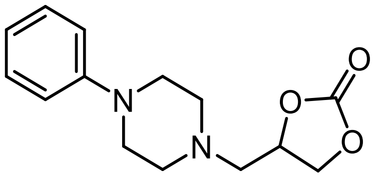

According to **GP1**, 1-(oxiran-2-ylmethyl)-4-phenylpiperazine (**1j**, 1.00 g, 4.60 mmol), **8** (103 mg, 0.230 mmol) and CO_2 were converted. After purification via column chromatography (SiO_2, *c*Hex:EtOAc= 5:1) the product **2j** (707 mg, 2.70 mmol, 59%) was obtained as a colorless solid.

^{1}H NMR (300 MHz, $CDCl_3$) δ= 2.64–2.86 (m, 6H), 3.21 (t, *J*= 5.0 Hz, 4H), 4.28 (dd, *J*= 8.5, 7.1 Hz, 1H), 4.48–4.63 (m, 1H), 4.83–4.94 (m, 1H), 6.85–6.95 (m, 3H), 7.23–7.31 (m, 2H) ppm.

^{13}C NMR (101 MHz, $CDCl_3$) δ= 49.13, 54.09, 59.95, 67.92, 75.11, 116.13, 119.96, 129.12, 151.02, 154.82 ppm.

IR (ATR) Neat: 517, 689, 753, 873, 917, 1004, 1051, 1076, 1149, 1170, 1234, 1260, 1317, 1382, 1449, 1493, 1597, 1775 (C=O), 2762, 2816, 2949, 3024, 3094, 3390 cm^{-1}

MS (EI, 70 eV): *m/z* (%)= 262 (18) [M^+], 201 (13), 200 (19), 175 (62), 132 (66), 119 (10), 106 (22), 105 (100), 104 (83), 91 (29), 87 (11), 78 (11), 77 (81), 70 (85), 57 (15), 56 (44), 51 (23), 43 (25), 42 (99), 41 (19), 29 (19).

(*R*)-4-((4-phenylpiperazin-1-yl)methyl)-1,3-dioxolan-2-one (*R*-2j)

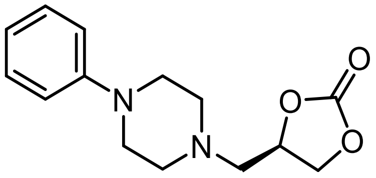

According to **GP1**, (*R*)-1-(oxiran-2-ylmethyl)-4-phenylpiperazine (***R*-2j**) (1.00 g, 4.60 mmol), **8** (103 mg, 0.230 mmol) and CO_2 were converted at 45 °C in 24 h. After purification *via* column chromatography (SiO_2, *c*Hex:EtOAc= 5:1) the product ***R*-2j** (1.08 g, 4.11 mmol, 90%, 24% *ee*) was obtained as an off white solid.

The enantiomeric excess was determined by Amylose 2 column, heptane/EtOH= 90:10 v/v, 1.0 mL/min, t_r (minor)= 21.909 min, t_r (major) =28.813 min.

(*S*)-4-((4-Phenylpiperazin-1-yl)methyl)-1,3-dioxolan-2-one (*S*-2j)

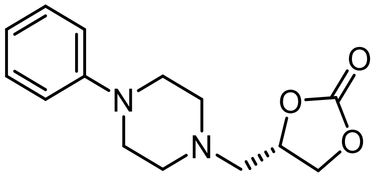

According to **GP3**, (*S*)-1-(oxiran-2-ylmethyl)-4-phenylpiperazine (***S*-1j**) (1.00 g, 4.60 mmol), **8** (103 mg, 0.230 mmol) and CO_2 were converted at 23 °C in 48 h. After purification *via* column chromatography (SiO_2, *c*Hex:EtOAc= 5:1, R_f= 0.59) the product *S*-**2j** (734 mg, 2.80 mmol, 61%, 54% *ee*) was obtained as an off-white solid.

The enantiomeric excess was determined by Amylose 2 column, heptane/EtOH= 90:10 v/v, 1.0 mL/min, t_r (major)= 21.847 min, t_r (major) =29.015 min.

3-(4-Phenylpiperazin-1-yl)propane-1,2-diol (*rac*-10)[11]

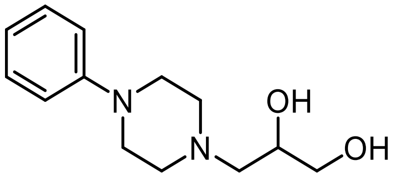

4-((4-Phenylpiperazin-1-yl)methyl)-1,3-dioxolan-2-one **2j** (262 mg, 1.00 mmol) was added to an aqueous NaOH solution (5 mL, 1 M). The mixture was stirred for 90 min during this time the starting material completely dissolved and a pale yellow solution was obtained. Subsequently, the reaction mixture was extracted with EtOAc (3×15 mL). The combined organic layers were dried over Na_2SO_4. After removal of all volatiles in vacuo product *rac*-**10** (225 mg, 0.950 mmol, 95%) was obtained as a colorless solid.

^{1}H NMR (300 MHz, $CDCl_3$) δ= 2.45 (dd, *J*= 12.5, 3.9 Hz, 1H), 2.57–2.70 (m, 3H), 2.73 (br s, 2H), 2.81–2.93 (m, 2H), 3.15–3.32 (m, 4H), 3.55 (dd, *J*= 11.4, 4.3 Hz, 1H), 3.79

(dd, J= 11.4, 3.8 Hz, 1H), 3.85–3.94 (m, 1H), 6.84–7.00 (m, 3H), 7.23–7.34 (m, 2H) ppm.

^{13}C NMR (101 MHz, $CDCl_3$) δ= 49.21, 53.37, 60.22, 64.77, 116.17, 119.99, 129.14, 132.46, 151.04 ppm.

IR (ATR) Neat: 427, 529, 697, 765, 922, 993, 1009, 1052, 1096, 1144, 1223, 1301, 1326, 1387, 1452, 1491, 1595, 2817, 2876, 2948, 3316 (O–H) cm^{-1}.

MS (EI, 70 eV): *m/z* (%)= 236 (18) [M^+], 205 (14), 176 (24), 175 (100), 160 (14), 132 (44), 120 (14), 105 (32), 104 (46), 91 (15), 77(39), 56 (17), 43 (18), 42 (28).

(*R*)-3-(4-Phenylpiperazin-1-yl)propane-1,2-diol (*R*-10)

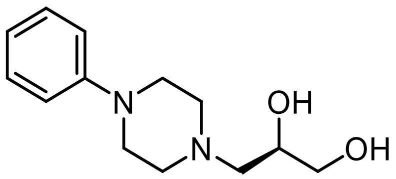

According to the synthesis of ***rac*-10**, (*R*)-4-((4-phenylpiperazin-1-yl)methyl)-1,3-dioxolan-2-one (***R*-2j**, 262 mg, 1.00 mmol) was converted in aq. NaOH (5 M, 5 mL). After work-up the title compound *R*-**10** (224 mg, 0.950 mmol, 95%, *ee*= 27%) was obtained as an colorless solid.

The enantiomeric excess was determined by Cellulose 3 column, heptane/EtOH= 95:5 v/v, 1.0 mL/min, t_r (minor)= 21.909 min, t_r (major) =28.813 min.

(*S*)-3-(4-Phenylpiperazin-1-yl)propane-1,2-diol (*S*-10)

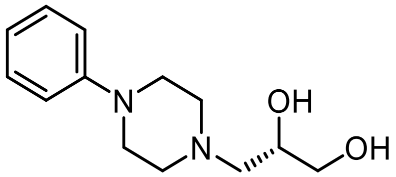

According to the synthesis of ***rac*-10**, 4-((4-phenylpiperazin-1-yl)methyl)-1,3-dioxolan-2-one (***S*-2j**, 262 mg, 1.00 mmol) was converted with aq. NaOH (5 M, 5 mL). After work-

up the title compound ***S*-10** (236 mg, 0.990 mmol, 99%, 54% *ee*) was obtained as an off-white solid.

The enantiomeric excess was determined on a Hydrodex, β-TBDAc (50 m), 190 °C, isotherm, heptane/EtOH= 80:20 v/v, *v*= 1.0 mL/min, t_r (minor)= 15.216 min, t_r (major)= 15.615 min, 54% *ee*.

6. Putative S_N1 and S_N2 reaction pathways

Based on the experimental results (Table 2 and 4) the two possible pathways of the reaction are postulated (Scheme S1).

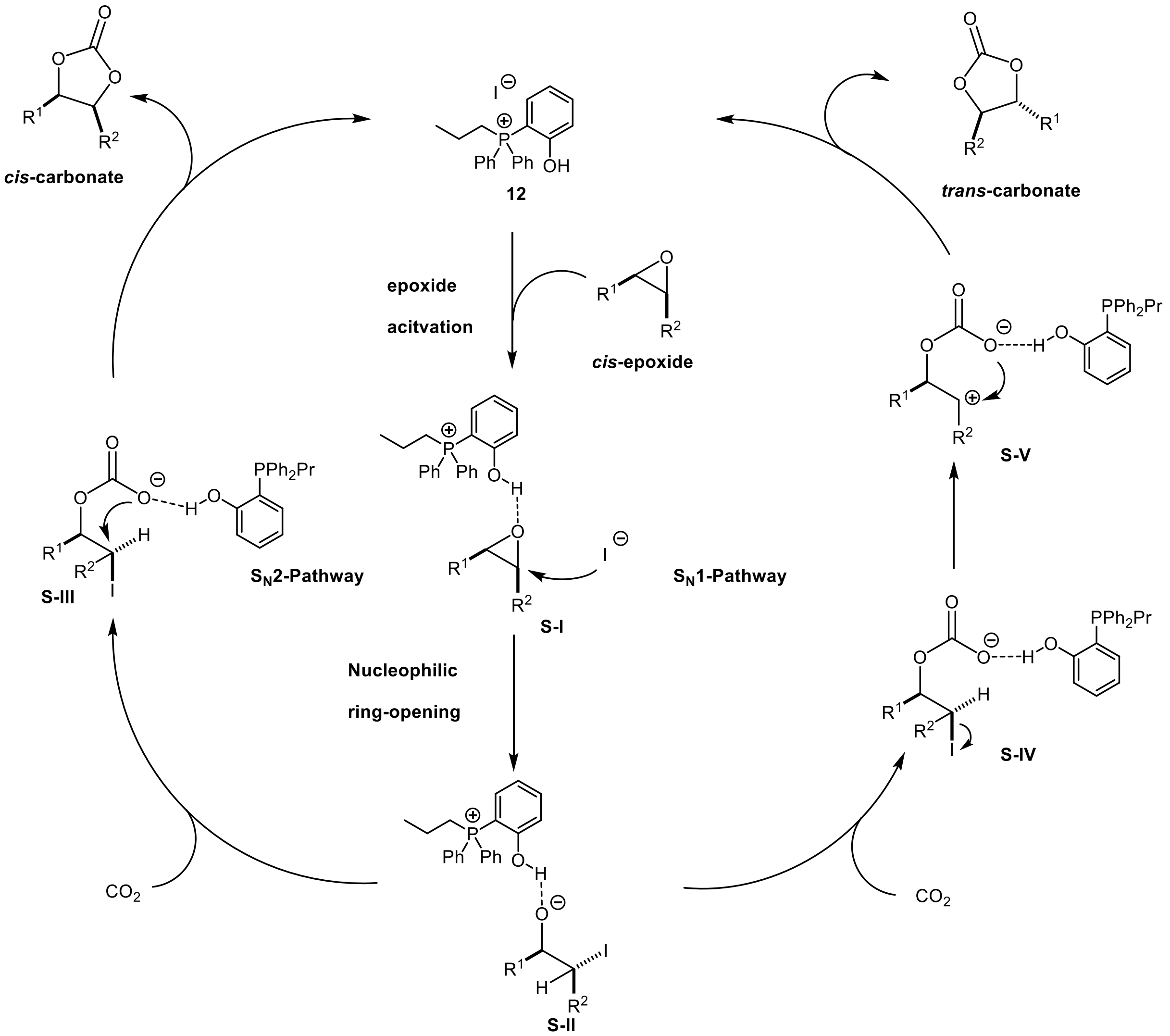

Scheme S1. Putative pathways in the synthesis of cyclic carbonates catalyzed by **8**.

After the epoxide activation (**S-I**) the reaction might proceed *via* a S_N1- or S_N2-pathway. Typically the reaction proceeded via two consecutive S_N2 steps (formation of **S-II** and

the intramolecular nucleophilic substitution to form **S-III**) thus as a result overall retention of stereochemistry is observed e.g. in the conversion of *S*-**1b** to *S*-**2b** However, when the substituent is capable of stabilizing a cationic intermediate (**S-V**), the reaction can hence also proceed via an S_N1 pathway, leading to partial racemization as observed e.g. in the conversion of *R*-**1e** to *R*-**2e**. This also explains the observation that in the conversion of *cis*-stilbene oxide *cis*-**13d** leads to *trans*-**14d** as the only product. In this case the phenyl group stabilized the cationic intermediate, in the ring closing step via S_N1-pathway, the thermodynamically preferred *trans*-product **14d** is obtained.

7. Infrared spectroscopic investigations.

In this section results from infrared spectroscopic measurements are presented. The initial objective was to identify possible differences in the location of the OH stretching bands for catalysts **7** and **8** in relevant liquid components: CH_3Cl, 1,2-butylene oxide **1a** and 1,2-butylene carbonate **2a**. By that approach, possibly information on the interaction between the catalyst and the substrate / product can be obtained which can relate to substrate activation and product inhibition. Another aim was to test whether differences in the band position in dependence of the anion (Br^-, I^-) are observable by infrared spectroscopy.

In the first step, we measured the solid catalyst materials **7** and **8** by ATR-IR-spectroscopy. For this purpose, an ALPHA I FTIR spectrometer from Bruker with a diamond ATR accessory (single reflection) was used. In the second step, we performed transmission-IR measurements of solutions in chloroform. These measurements have been conducted with a Bruker Tensor 27 spectrometer equipped with a MCT-detector and a transmission cell with CaF_2 windows featuring an optical pathlength of 100 µm. Measurements in transmission mode of solutions with 1,2-butylene oxide and 1,2-butylene carbonate were not successful, since bands for the OH stretching vibration of the catalysts and those for CH stretching vibrations of the solvents overlapped strongly and consequently no proper background subtraction was possible. Therefore, the liquid samples of the **7** and **8** in 1,2-butylene oxide and 1,2 butylene carbonate have been performed using ATR-IR-spectroscopy.

An overview of the experimental values and values calculated by DFT methods for the band position of the OH stretching vibrations is given in Table S1.

Table S1. Positions of OH stretching bands of catalyst **7** and **8**.

IR[a] ν(OH) / cm^{-1}	Solid	Solvent: $CHCl_3$	Solvent: 1,2-butylene oxide	Solvent: 1,2-butylene carbonate	DFT[b], uncorrected(gas phase)
[*n*PrP(Ph)$_2$PhOH]Br (7)	≈ 2850	≈ 2922	≈ 3079	≈ 2886	2676
[*n*PrP(Ph)$_2$PhOH]I (8)	≈ 2990	≈ 3051	≈ 3072	≈ 3066	2818

[a]All experimental values should be treated with some caution, because due to the broadness of the bands and overlapping with CH stretching bands uncertainties were unavoidable. [b]Gaussian 09, B3LYP, Def2-TZVP.

Solid samples of catalysts 7 and 8.

The experimental spectra of the solids **7** and **8** are displayed in Figures **S1** and **S2**. Bands for associated *ortho*-substituted phenolic hydroxyl groups are located at lower wavenumbers compared to unsubstituted aliphatic OH groups.[12] Band positions have been determined with ν(OH) ≈ 2850 cm^{-1} for **7** (Br^-) and ν(OH) ≈ 2990 cm^{-1} for **8** (I^-). The difference of these values can be explained by hydrogen bonding -O-H···X^-, which strength depends on the halide. Higher wavenumbers of the OH stretching vibration indicate a less strong interaction between the OH group and the respective anion. Thus, the experimental data is in accordance with a stronger hydrogen bonding between the OH group and the bromide compared to the iodide.

A similar trend was found from by comparing the uncorrected values of the OH stretching vibration frequencies of **7** and **8** by DFT calculations in the gas phase with ν(OH) ≈ 2676 cm^{-1} for **7** (Br^-) and ν(OH) ≈ 2818 cm^{-1} for **8** (I^-).

In a previous study on aliphatic systems in solution of the type [*n*Bu$_3$P(CH_2)$_2$OH]X (X: Cl^-, Br, I^-) it was also observed that the frequency of the OH stretching vibration showed

a dependency on the halide anion. The frequencies ν(OH) increased in the order of Cl^- < Br^- < I^-.[13]

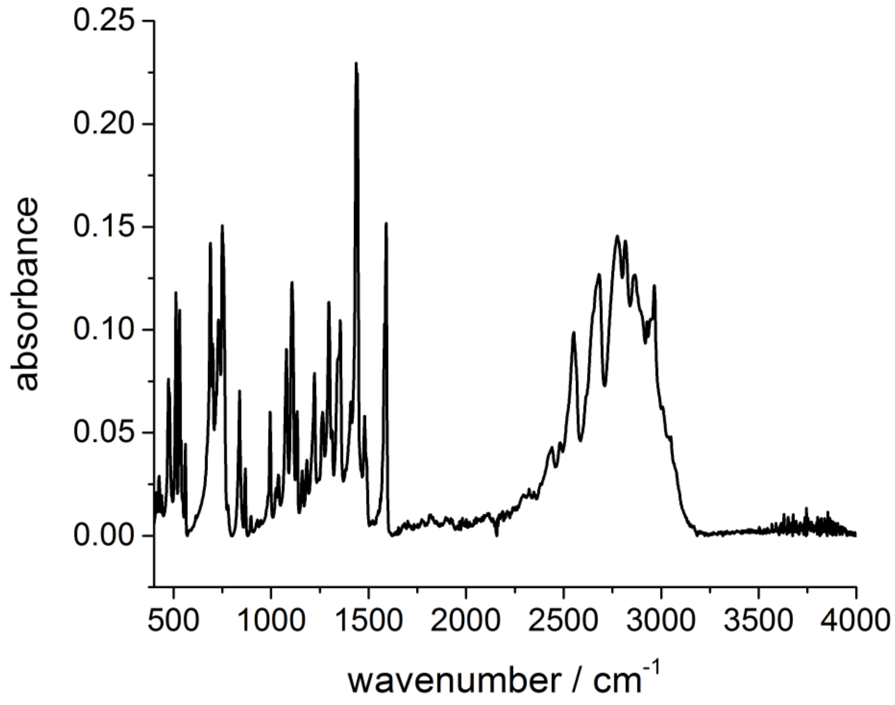

Figure S1. ATR-IR spectrum of the solid catalyst [*n*PrP(Ph)$_2$PhOH]Br (**7**) at room temperature (ν(OH) ≈ 2850 cm^{-1}).

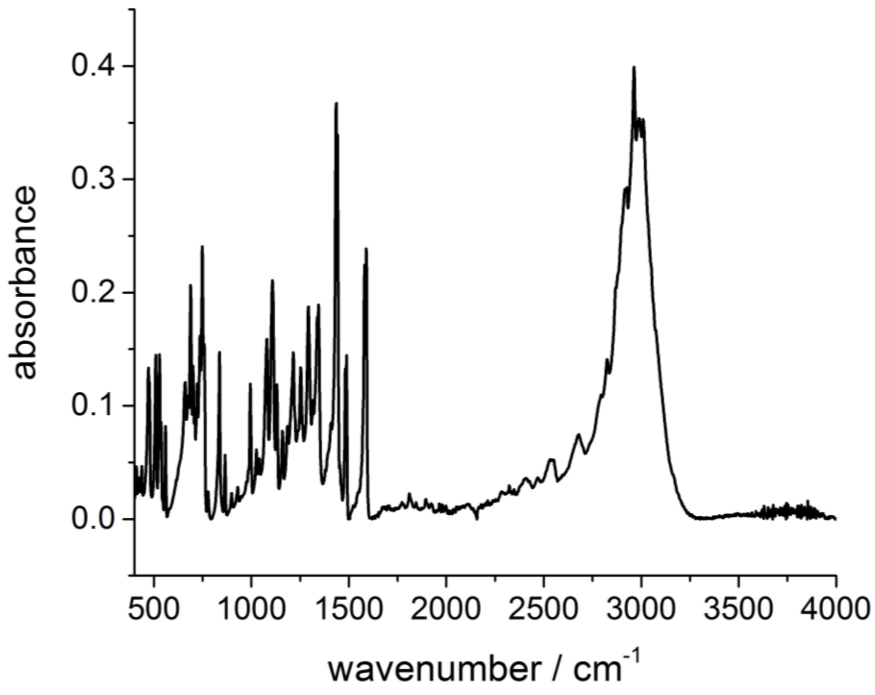

Figure S2. ATR-IR spectrum of the solid catalyst [*n*PrP(Ph)$_2$PhOH]I (**8**) at room temperature (ν(OH) ≈ 2990 cm^{-1}).

Liquid samples of solutions of catalysts 7 and 8 in chloroform.

The experimental transmission-IR spectra of **7** and **8** in chloroform are displayed in Figures **S3** and **S4**. The concentration of **7** and **8** was 2 mol%. The central band position of the OH group have been determined by a numerical curve fitting procedure implemented in Bruker OPUS 7.0. Besides the broad band for the hydroxyl group, a band for CH stretching vibrations within the catalysts were observed. Band positions have been determined with ν(OH) ≈ 2922 cm^{-1} for **7** (Br^-) and ν(OH) ≈ 3051 cm^{-1} for **8** (I^-). These experimental results are also in agreement with a stronger hydrogen bonding between the OH group and the bromide compared to the iodide.

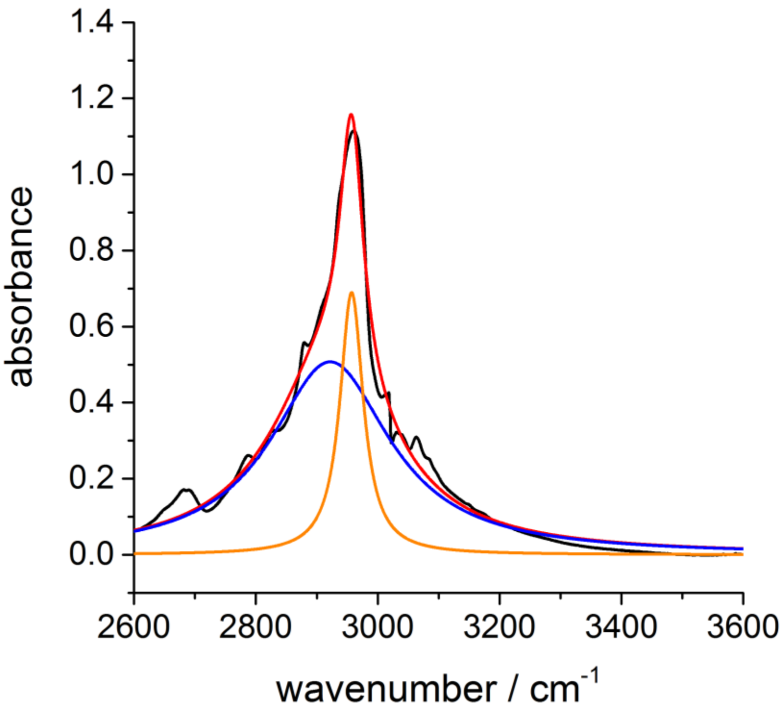

Figure S3. IR spectrum of the catalyst [*n*PrP(Ph)$_2$PhOH]Br **7** (2 mol%) in $CHCl_3$ at room temperature (ν(OH) ≈ 2922 cm^{-1}). (— Experimental spectrum, — fitted OH-band curve, — fitted CH-band curve, — sum of fitted band curves).

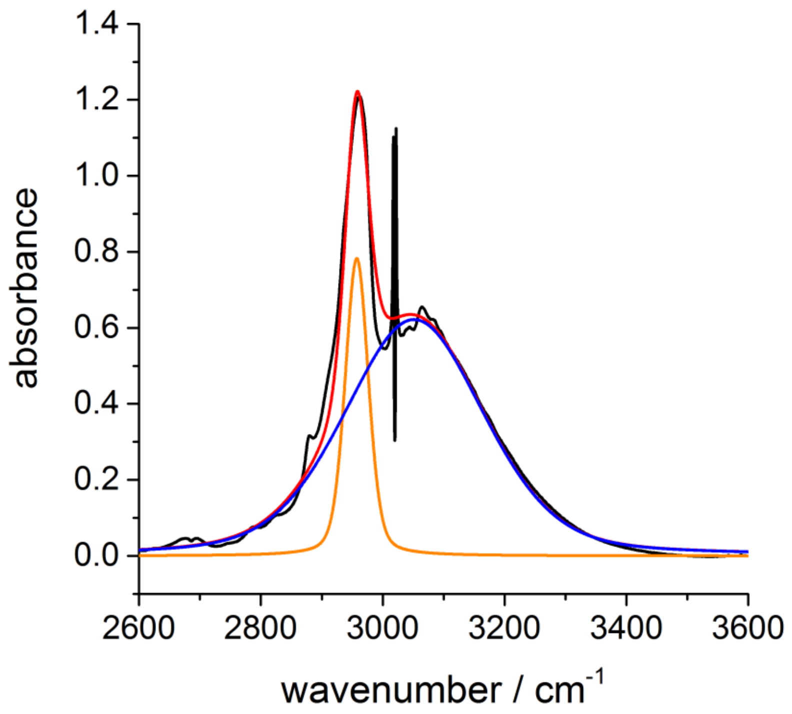

Figure S4. Transmission-IR spectrum of the catalyst [*n*PrP(Ph)$_2$PhOH]I **8** (2 mol%) in $CHCl_3$ at room temperature (ν(OH) ≈ 3051 cm^{-1}). (— Experimental spectrum, — fitted OH-band curve, — fitted CH-band curve, — sum of fitted band curves).

Liquid samples of solutions of catalysts 7 and 8 in 1,2-butylene oxide.

The experimental ATR-IR spectra of **7** and **8** (2 mol%) in 1,2-butylene oxide are displayed in Figures **S5** and **S6**. The location of the OH stretching band have been determined by a numerical curve fitting procedure implemented in Bruker OPUS 7.0. In addition to the broad band for the OH group with ν(OH) ≈ 3079 cm^{-1} for **7** (Br^-) and ν(OH) ≈ 3072 cm^{-1} for **8** (I^-) further bands for CH stretching vibrations of the catalysts have been observed. These experimental values should be treated with some caution, because the low signal intensity, the broadness of the OH bands and the overlapping with CH stretching bands lead to unavoidable uncertainties. These uncertainties could be the reason why it was apparently not possible to identify a significant effect of the halide on the position of the OH stretching vibration. It was not possible to draw any further information regarding the substrate activation from these measurements.

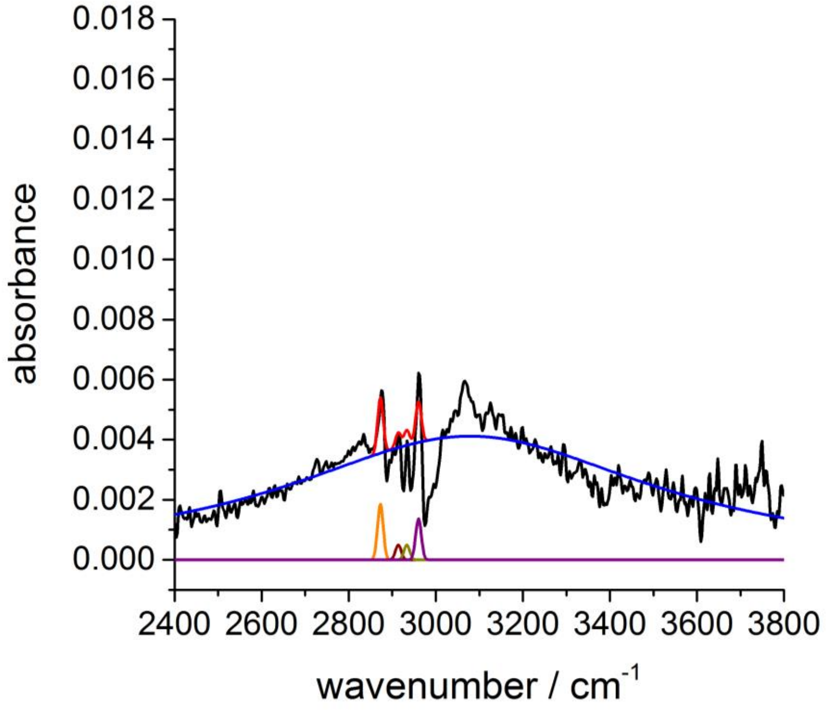

Figure S5. ATR-IR spectrum of a liquid sample of the catalyst [*n*PrP(Ph)$_2$PhOH]Br **7** (2 mol%) in 1,2-butylene oxide (**1a**) (ν(OH) ≈ 3079 cm^{-1}). (— Experimental spectrum, — fitted OH-band curve, colored curved: fitted CH-band curve, — sum of fitted band curves).

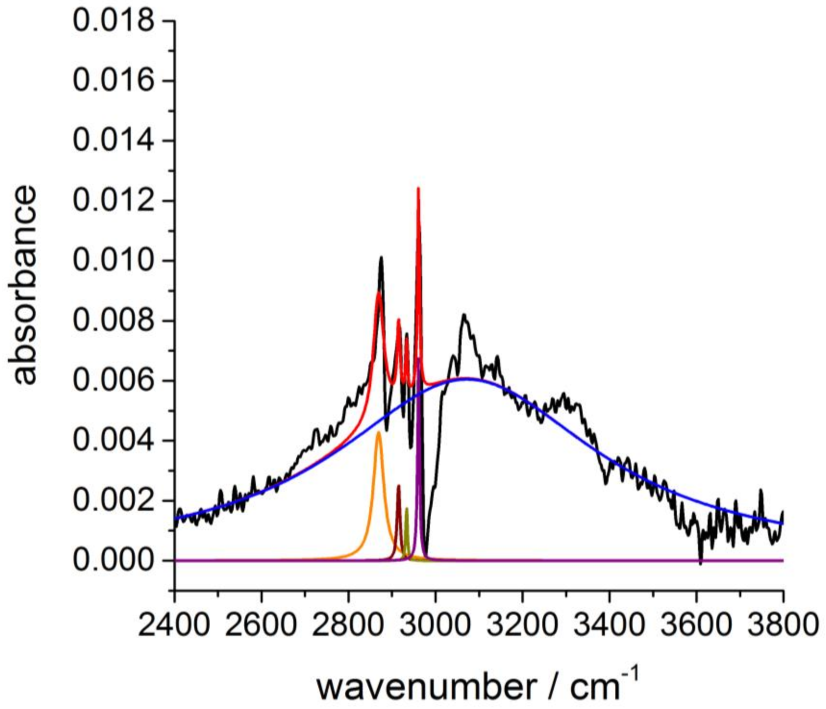

Figure S6. ATR-IR spectrum of a liquid sample of the catalyst [*n*PrP(Ph)$_2$PhOH]I **8** (2 mol%) in 1,2-butylene oxide (**1a**) (ν(OH) ≈ 3072 cm^{-1}). (— Experimental spectrum, — fitted OH-band curve, colored curved: fitted CH-band curve, — sum of fitted band curves).

Liquid samples of solutions of catalysts 7 and 8 in 1,2-butylene carbonate.

The experimental ATR-IR spectra of **7** and **8** (2 mol%) in 1,2-butylene carbonate are displayed in Figures **S7** and **S8**. The location of the OH stretching band have been estimated using a numerical curve fitting procedure implemented in Bruker OPUS 7.0. The broad bands for the OH group was found at ν(OH) ≈ 2886 cm^{-1} for **7** (Br^-) and ν(OH) ≈ 3066 cm^{-1} for **8** (I^-). In addition, further bands for CH stretching vibrations of the catalysts have been observed. These experimental values should be treated with reasonable caution, because due to the low signal intensities, the broadness of the OH bands and the overlapping with CH stretching bands uncertainties are unavoidable.

These experimental results are also consistent with a stronger hydrogen bonding between the OH group and the bromide compared to the iodide.

From these measurements it was not possible to identify any further bands which could relate to adducts formed by the interaction between the catalysts and the carbonate product.

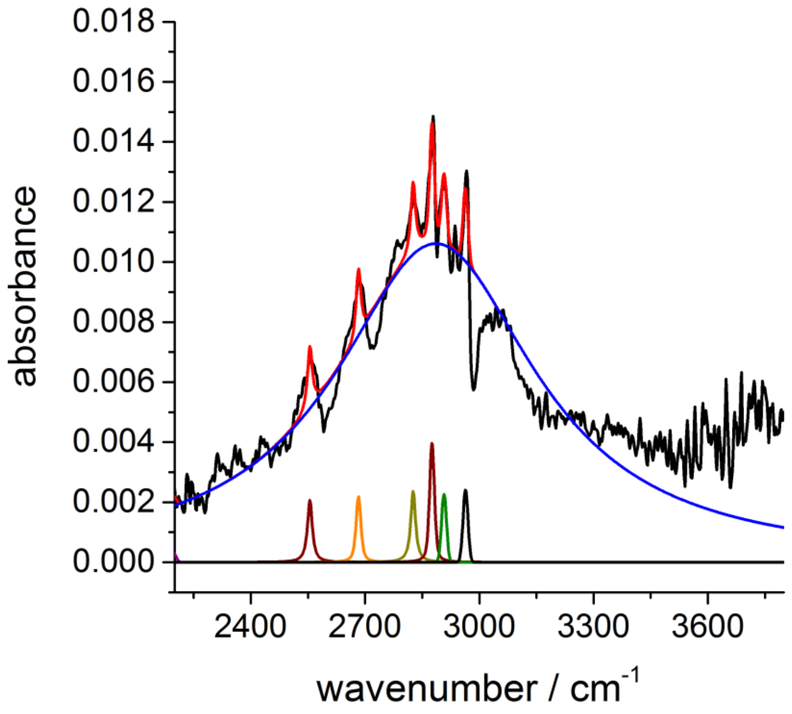

Figure S7. ATR-IR spectrum of a liquid sample of the catalyst [*n*PrP(Ph)$_2$PhOH]Br **7** (2 mol%) in 1,2-butylene carbonate (**2a**) (ν(OH) ≈ 2886 cm^{-1}). (— Experimental spectrum, — fitted OH-band curve, colored curved: fitted CH-band curve, — sum of fitted band curves).

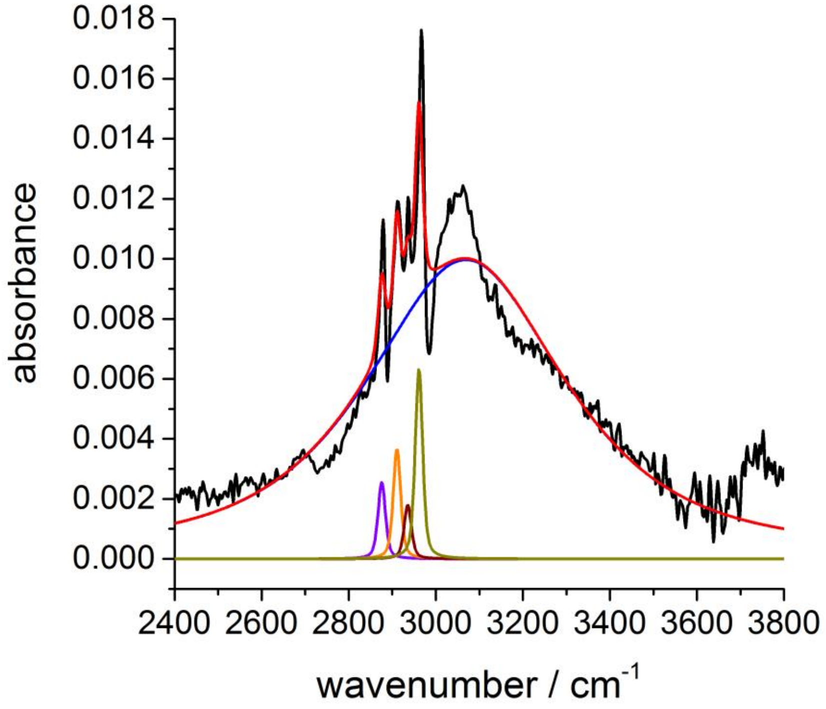

Figure S8. ATR-IR spectrum of a liquid sample of the catalyst [*n*PrP(Ph)$_2$PhOH]I **8** (2 mol%) in 1,2-butylene carbonate (**2a**) (ν(OH) ≈ 3066 cm^{-1}). (— Experimental spectrum, — fitted OH-band curve, colored curved: fitted CH-band curve, — sum of fitted band curves).

8. Control experiments with catalyst 3 and 8 at room temperature

As shown in Table 1, we obtained 25% and 65% of 1,2-butylene carbonate (**2a**) catalyzed by **3** and **8** with 2 mol% catalyst loading at 23 °C, respectively. The difference in catalytic ability might be attributed to the epoxide ring-opening step, thus ^1H NMR experiments using stoichiometric amount of catalyst **3** or **8** in the absence of CO_2 were investigated (Table S2). Iodohydrin **S1** was observed with catalyst **8** at 23 °C, however, it is not observed with catalyst **3**. When the temperature was increased to 45 °C, iodohydrin was obtained in only 2% with catalyst **3**.

Table S2. Ring-opening of 1,2-butylene oxide with stoichiometric amount of catalysts: ^1H NMR experiments.

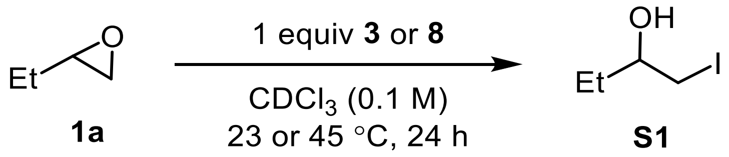

Entry	Catalyst	*T*/ °C	Yield S1/ %
1	**3**	23	0
2	**8**	23	58
3	**3**	45	2
4	**8**	45	60

To gain deep insights, kinetic studies were performed with both aliphatic **3** and phenolic phosphonium **8** iodides. The conversion of 1,2-butylene oxide **1a** with CO_2 under defined solvent-free conditions was chosen as the model reaction. The kinetic studies were firstly conducted under standard conditions (5 mol% catalyst, 23 °C, 36 h, $p(CO_2)$= 1.0 MPa) with the respective catalysts. (Figure S9) The phenolic catalyst **8** is more active than **3**, but an induction period was observed with catalyst **8**. Therefore, we studied the impact on the solubility of the catalyst at room temperature (Scheme S2).

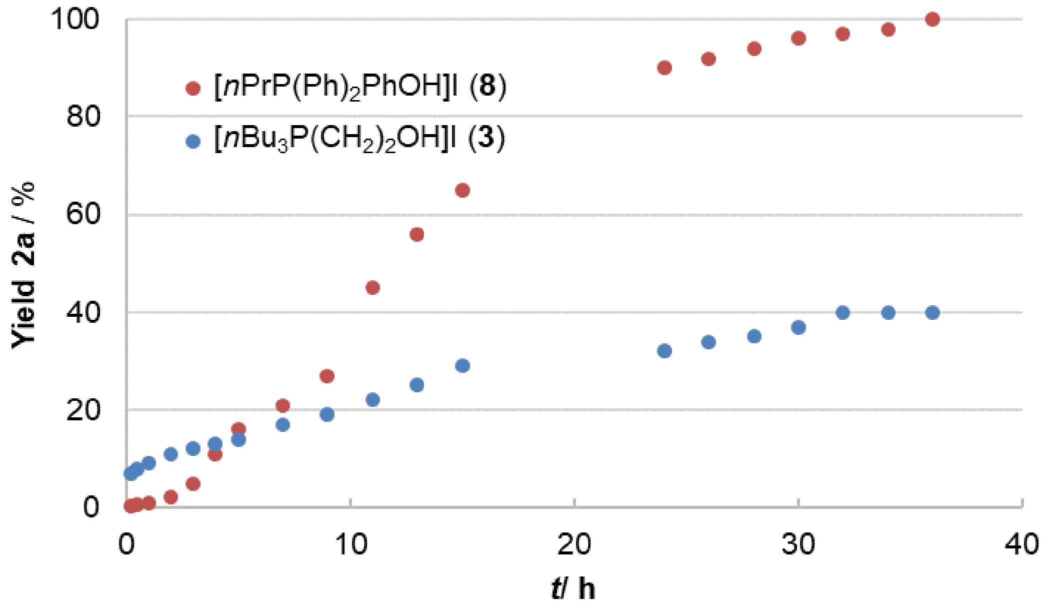

Figure S9. Yield vs. time data with catalysts **3** and **8** at room temperature. The observed selectivity was >99%. Reaction conditions: 1,2-butylene oxide (**1a**, 460 mmol), 5 mol% catalyst **3** or **8**, $p(CO_2)$= 1.0 MPa, 23 °C, 38 h.

In the first reaction, the substrate **1a** was converted under standard reaction conditions. The reaction was stopped after 1 h and only trace amount of product **2a** was observed (Scheme S2a). In the second reaction, the catalyst was stirred with the substrate **1a** for 4 h under argon atmosphere, then CO_2 was introduced, and the reaction proceeded for additional 1 h. In this case the amount of the product **2a** did not significantly increase (Scheme S2b). This indicates that no other catalytic species generated during the 1st hour. In the third reaction, the mixture of **1a** and **2a** (80:20) was stirred with catalyst **8** for 1 h. Notably, in this case **2a** was obtained in 22%. The yield increased to 78% after 8 h (Scheme S2c). This is comparable to the results attained from the Figure S9 (21% of **2a** after 7 h, and 65% after 15 h). Hence, the low conversion in the first hours can be addressed to low solubility of the catalyst in **1a** at room temperature. The solubility issue can be overcome by performing the reaction at 45 °C.

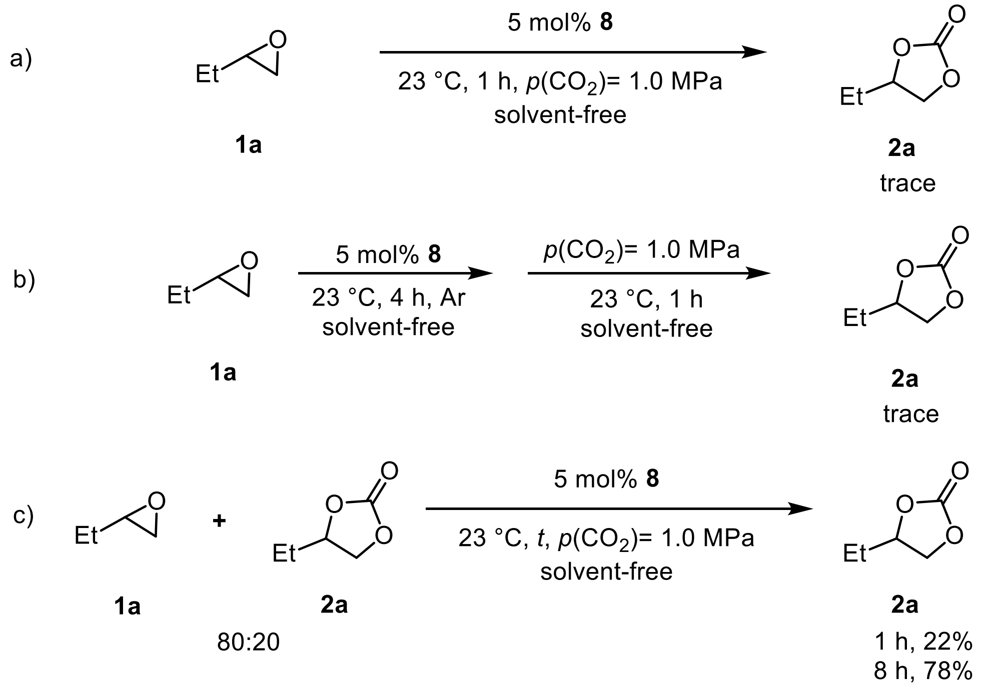

Scheme S2. Control experiments to investigate the induction period with catalyst **8** at room temperature.

9 Kinetic investigations

General procedure for the kinetic studies (GP3)

A 100 cm^3 stainless-steel autoclave was charged with the catalyst **3** or **8** (2 mol%) and 1,2-butylene oxide (**1a**, 460 mmol). The reaction solution was stirred for 24–48 h at different temperatures and under a CO_2 pressure of 1.0 MPa. For the kinetic data NMR samples were taken directly from the autoclave.

9.1 Kinetic evaluation of yield vs. time data for catalysts 3 and 8

In this section the results of the kinetic evaluation of collected yield vs. time data during the 1,2-butylene oxide/CO_2 coupling with bifunctional catalysts [*n*$Bu_3P(CH_2)_2OH$]I (**3**) and [*n*PrP(Ph)$_2$PhOH]I (**8**) are discussed (see Figure 1 in the manuscript).

We consider the following simplified kinetic models:

a) Michaelis-Menten Kinetics

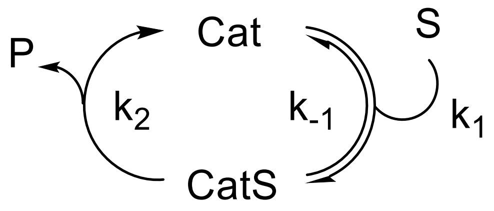

Scheme S3. Michaelis-Menten model. Cat: catalyst, S: substrate, P: product, CatS: catalyst-substrate complex.

$$\frac{d[P]}{dt} = \frac{k_2[\mathrm{Cat}]^0[\mathrm{S}]}{K_m + [\mathrm{S}]} \qquad \text{(Eq. SI-1)}$$

For our catalytic systems we assume the case, where $[S]^0 \ll K_m$, so that the rate equation for the product formation becomes Eq. SI-2.

$$\frac{d[P]}{dt} = k^{obs}[\mathrm{S}] \qquad \left(\text{with: } k^{obs} = \frac{k_2[\mathrm{Cat}]^0}{K_m}\right) \qquad \text{(Eq. SI-2)}$$

b) Michaelis-Menten Kinetics with product inhibition

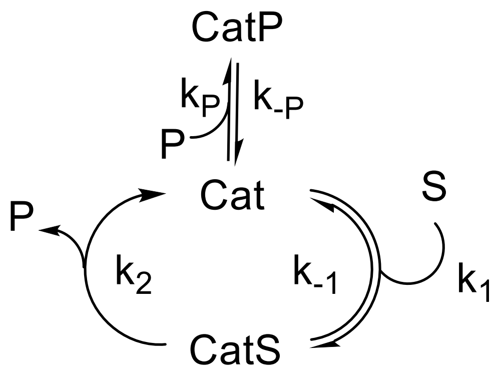

Scheme S4. Michaelis-Menten model including an inhibition equilibrium between catalyst and catalyst-product complex. Cat: catalyst, S: substrate, P: product, CatS: catalyst-substrate complex, CatP: catalyst-product complex.

For that kinetic model the resulting rate equation for product formation is given in Equation SI-3.

$$\frac{d[P]}{dt} = \frac{k_2[\text{Cat}]^0[\text{S}]}{K_m(1 + K_{\text{inh}}[\text{P}]) + [\text{S}]} \qquad \text{(Eq. SI-3)}$$

For our catalytic systems it is assumed that $[S]^0 \ll K_m$, by which the rate equation for the product formation becomes Eq. SI-4.

$$\frac{d[P]}{dt} = \frac{k^{obs}}{(1 + K_{\text{inh}}[\text{P}])}[\text{S}] \qquad \text{(with: } k^{obs} = \frac{k_2[\text{Cat}]^0}{K_m}\text{)} \qquad \text{(Eq. SI-4)}$$

For numeric parameter estimation we did a rearrangement of the Equations SI-2 and SI-4 using the dimensionless yield Y instead of [S] and [P]. Additionally, we used the linearized form of the 1st order model by integration.

In the following Table respected equations used for parameter estimation are given:

Table S3. Equations for estimation of kinetic parameters based on yield-vs-time data analysis.

a) 1st Order kinetics, linearized	$ln(1 - Y) = -k^{obs}t$ (Eq. SI-5)
b) 1st Order kinetics	$\frac{dY}{dt} = k^{obs}(1 - Y)$ (Eq. SI-6)
c) 1st Order kinetics with product inhibition	$\frac{dY}{dt} = \frac{k^{obs}(1 - Y)}{1 + K_{\text{inh}}[\text{S}]^0 Y}$ (Eq. SI-7)

For the integration/regression-procedure based on an ordinary differential equation (ODE) the implicit Runge-Kutta method Radau IIA with variable orders (5,9,13) was used for the integration of ordinary differential equations and an advanced Gauß-Newton code was used for the optimization.[14]

9.1.1 Kinetic evaluation of the yield vs. time data for the [nBu$_3$P(CH$_2$)$_2$OH]I (3) catalyst

In Figure S10 a), b) and c) the kinetic evaluation of the formation of 1,2-butylene carbonate (**2a**) using catalyst **3** is shown.

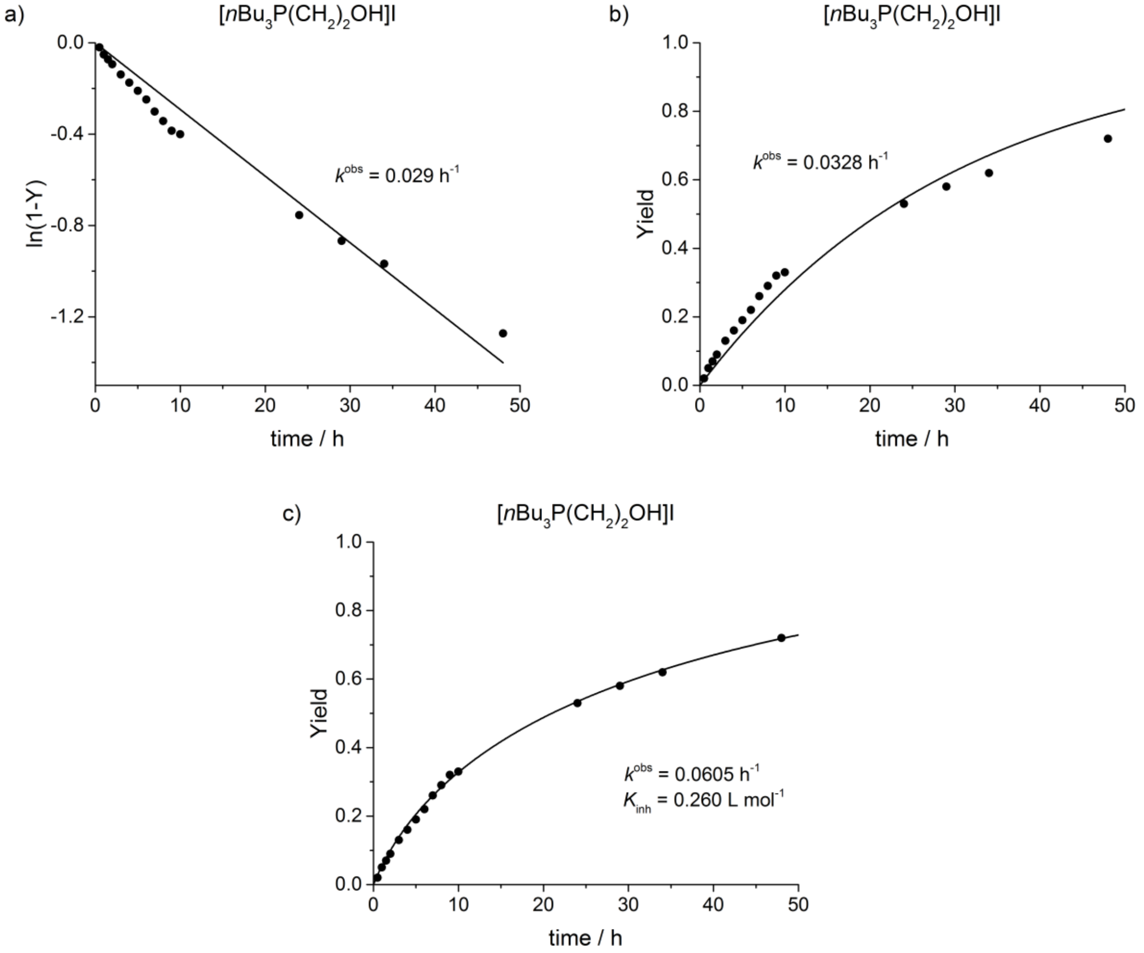

Figure S10. Kinetic evaluation for the formation of butylene carbonate from butylene oxide and CO_2 using [nBu$_3$P(CH$_2$)$_2$OH]I **3** as organocatalyst. Reaction conditions: Cat: **3** 2 mol%, T=45°C, p(CO_2)= 1.0 MPa. a) 1st Order kinetics (linearized), b) 1st Order kinetics, c) 1st Order kinetics with product inhibition.

9.1.2 Kinetic evaluation of the yield vs. time data for the [*n*PrP(Ph)$_2$PhOH]I (8) catalyst

In Figure S11 a), b) and c) the kinetic evaluation of the formation of 1,2-butylene carbonate (**2a**) using catalyst [*n*PrP(Ph)$_2$PhOH]I (**8**) is shown.

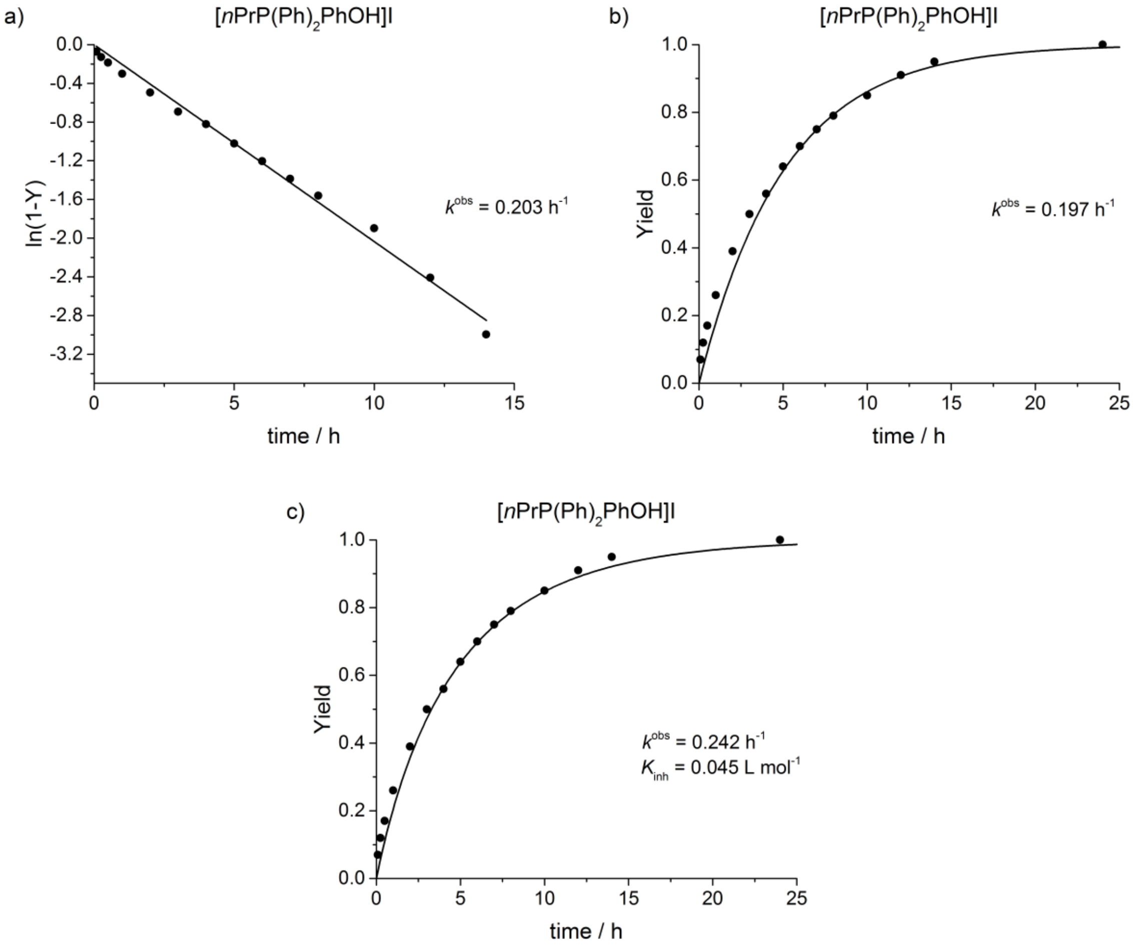

Figure S11. Kinetic evaluation for the formation of butylene carbonate from 1,2-butylene oxide and CO_2 using [*n*PrP(Ph)$_2$PhOH]I **8** as organocatalyst. Reaction conditions: Cat: **8** 2 mol%, T= 45°C, $p(CO_2)$= 1.0 MPa. a) 1st Order kinetics (linearized), b) 1st Order kinetics, c) 1st Order kinetics with product inhibition.

In Table S4 the obtained kinetic parameters are given for the different methods and kinetic models.

Table S4. Values for the kinetic parameters obtained via data analysis for reactions using [nBu$_3$P(CH$_2$)$_2$OH]I (**3**) and [nPrP(Ph)$_2$(Ph)OH]I (**8**) catalyst systems.

**Catalyst**	k^{obs}/h^{-1} (1st Order, lin.)	k^{obs}/h^{-1} (1st Order)	k^{obs}/h^{-1} (Product inh., Eq. S7)	$K_{inh}/L\,mol^{-1}$ (Product inh., Eq. S7)	$[S]^0K_{inh}$	$[S]^0$ $/mol\,L^{-1}$
[nBu$_3$P(CH$_2$)$_2$OH]I	0.029	0.0328	0.0605	0.260	2.9864	11.5
[nPrP(Ph)$_2$(Ph)OH]I	0.203	0.197	0.245	0.045	0.5177	11.5

For the appropriate description of the yield vs time profile of the catalytic system with [nBu$_3$P(CH$_2$)$_2$OH]I (**3**) as a catalyst the usage of the kinetic model with product inhibition (Eq. SI-7) was necessary which provided values for the rate constant with k^{obs} = 0.0605 h^{-1} and the inhibition constant with K_{inh} = 0.260 L mol^{-1}.

Since the yield vs time data for the system with the catalyst [nPrP(Ph)$_2$(Ph)OH]I (**8**) can be described adequately by a simple first-order kinetic model, we conclude that product inhibition is not significantly effective and can be neglected. Using the kinetic model (Eq. SI-7) for data fitting only a very small value for the inhibition constant K_{inh}= 0.045 L mol^{-1} was determined. Therefore, the value for the rate constant with k^{obs} = 0.197 h^{-1} was used.

9.2 The yield vs. time data of 1,2-butylene carbonate formation at various temperatures

In Figure S12 the yield vs. time data for the formation of 1,2-butylene carbonate (**2a**) for the temperature range of θ= 23 – 90 °C is depicted.

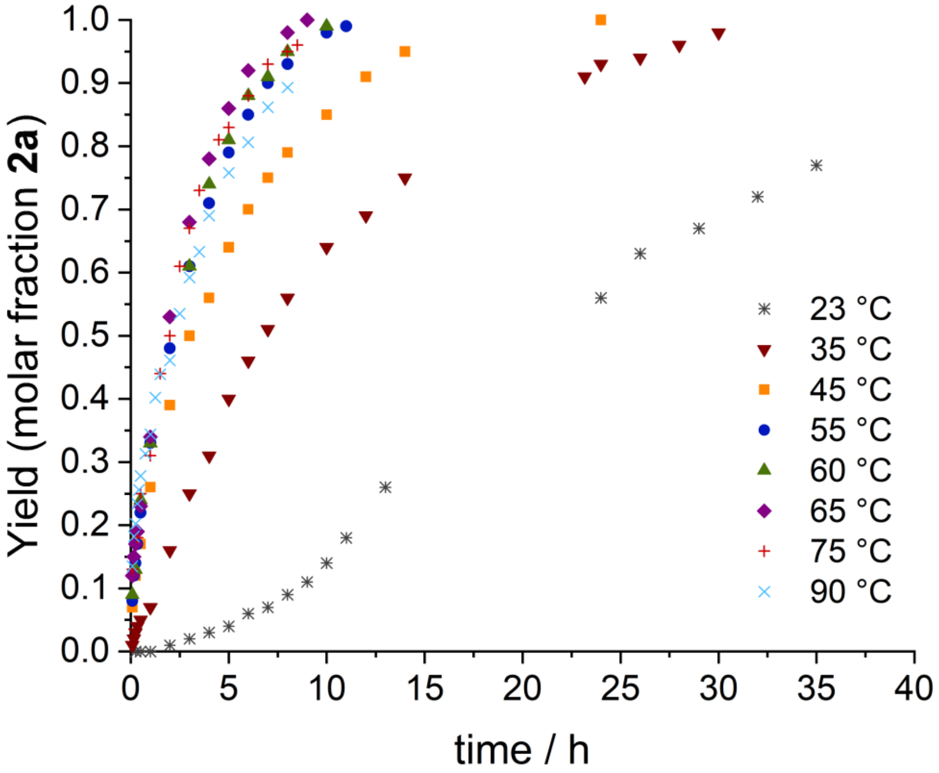

Figure S12. Formation of 1,2-butylene carbonate (**2a**) catalyzed by phenolic phosphonium iodide at various temperatures. Reaction conditions: 1,2-butylene oxide (**1a**, 460 mmol), 2 mol% catalyst **8**, p(CO_2)= 1.0 MPa. The observed selectivity was >99%.

As already noted, for θ= 23 °C a pronounced induction period was observed which is attributed this to the low solubility of the catalyst **8** in the pure 1,2-butylene oxide substrate at room temperature. The yield vs. time profiles for θ= 35 – 90 °C could be described by a first-order kinetic model with respect to the substrate concentration. For the calculation of the apparent rate constants k^{obs} the respective linearized model ($\ln(1-y) = -k^{obs}t$) was used (Table S5, Figure S13). Interestingly, the values of the rate constants start decreasing with ≥75 °C. One possible reason could be the decrease of the solubility of CO_2 at higher temperatures.[15]

Table S5. Values for the apparent rate constant k^{obs} from the fitting process using the linearized first-order kinetic model.

**θ / °C**	35	45	55	60	65	75	90
$\boldsymbol{k^{obs}/h^{-1}}$	0.102	0.203	0.324	0.353	0.401	0.369	0.287

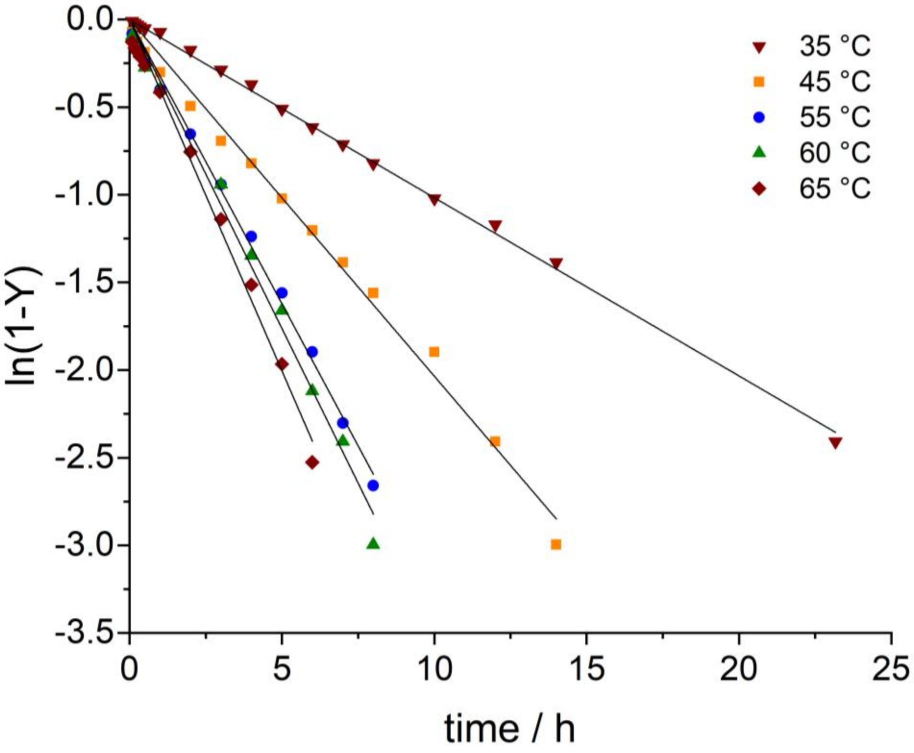

Figure S13. Graphical plotting of ln(1-y) vs. time for the calculation of the apparent rate constant k^{obs} from the slope of the linear fitting (only the data for the temperature range between θ= 35 – 65 °C is shown).

9.3 Calculation of the activation energy from an Arrhenius-plot

According to the Arrhenius equation (Equation SI-8) and the respective linearized expression (Equation SI-9) an Arrhenius-plot was created to estimate a value for the activation energy with $E_a = 39.6$ kJ/mol.

$$k = Ae^{-\frac{Ea}{RT}} \qquad \text{(Eq. SI-8)}$$

$$\ln(k) = -\frac{Ea}{RT} + \ln(A) \qquad \text{(Eq. SI-9)}$$

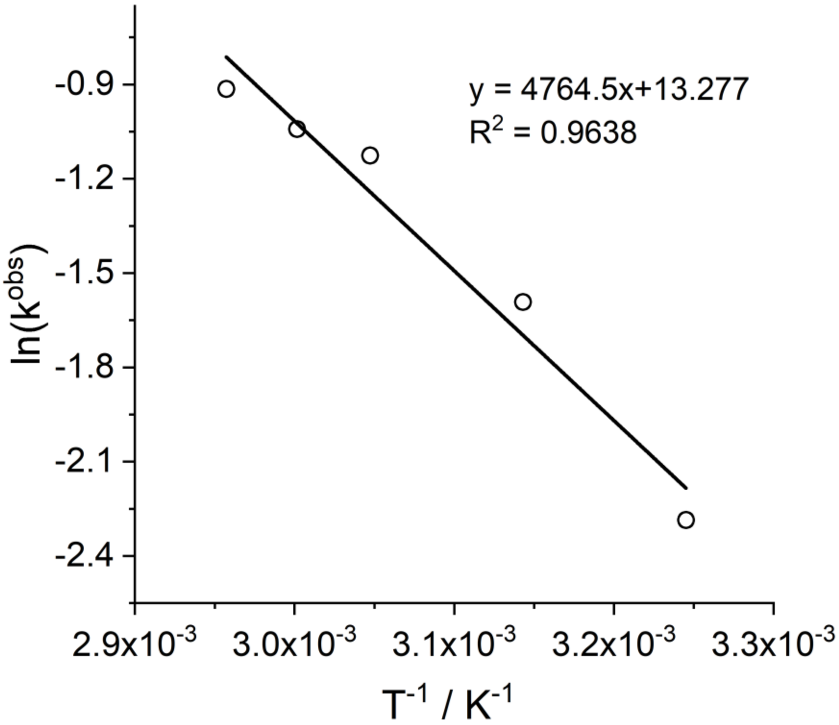

Figure S14. Arrhenius-plot for the estimation of the activation energy E_a of the conversion of epoxide **1a** with CO_2 in the presence of catalyst **8** over the temperature range of θ= 35 – 65 °C (T= 308.15 – 338.15 K).

9.4 Calculation of the enthalpy and entropy of activation

Starting from the Eyring equation (Eq. SI-10) and the Gibbs free energy equation (Eq. SI-11) a rearrangement leads to equation (Eq. SI-12) from which the enthalpy and entropy of activation can be calculated. Therefore a plot of $\ln(k/T)$ vs T^{-1} is constructed (Eyring-plot) from which the slope and the intercept allows for the calculation of $\Delta H^{\neq}$ and $\Delta S^{\neq}$.

$$k = \frac{k_B T}{h} e^{-\frac{\Delta G^{\neq}}{RT}} \qquad \text{(Eq. SI-10)}$$

$$\Delta G^{\neq} = \Delta H^{\neq} - T\Delta S^{\neq} \qquad \text{(Eq. SI-11)}$$

$$\ln\frac{k}{T} = -\frac{\Delta H^{\neq}}{RT} + \frac{\Delta S}{R} + \ln\frac{k_B}{h} \qquad \text{(Eq. SI-12)}$$

→ $\Delta H^{\neq} = 36.9\, kJ\, mol^{-1}$

→ $\Delta S^{\neq} = -0.212\, kJ\, mol^{-1}\, K^{-1}$

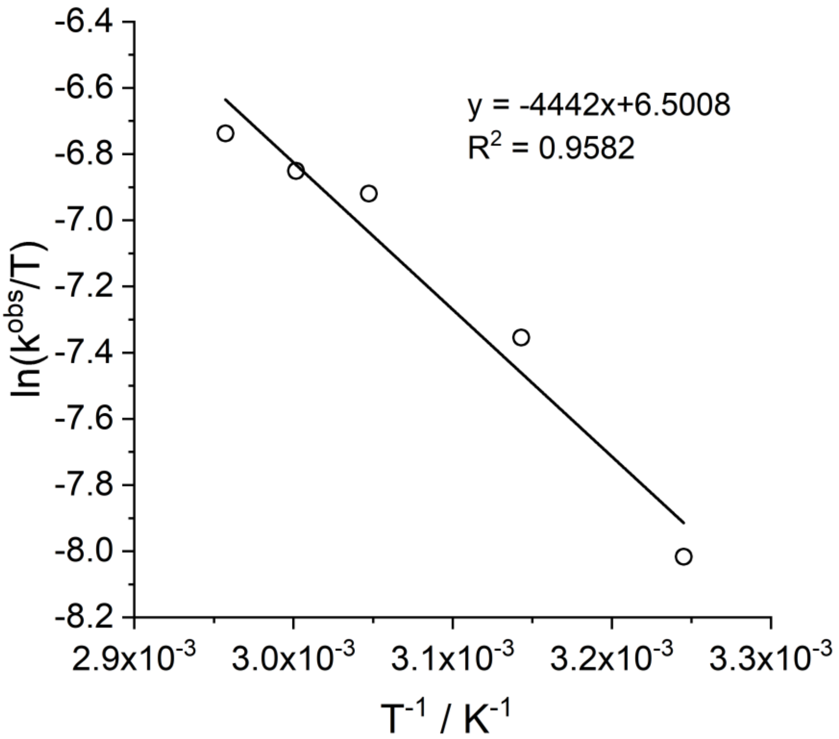

Figure S15. Eyring-plot for the estimation of the enthalpy and entropy of activation for the conversion of epoxide **1a** with CO_2 in the presence of catalyst **8** over the temperature range of θ= 35 – 65 °C (T= 308.15 – 338.15 K).

10 DFT calculation

All calculations were carried out by using the Gaussian 09 program. [16] Geometry optimizations were carried out in gas phase at the B3LYP[17] level with Def2-TZVP[18] basis set. All optimized structures were characterized either as energy minimums without imaginary frequencies or transition states with only one imaginary frequency by frequency calculations. With B3LYP/Def2-TZVP geometries obtained in gas phase, the energies were further refined by single-point calculations under the consideration of 1,2-butylene carbonate (ε= 57.5[19]) as solvent based on solute electron density (SMD) at the M06-2X/Def2-TZVP[20] level. Zero-point vibrational energies and thermodynamic corrections were calculated at the level of geometry optimization at 298.15 K under 1 atm. The Gibbs free energies were further corrected to standard state in solution with a standard concentration of 1 mol/L (p= 24.5 atm) from standard state in gas phase (p= 1 atm). The optimized structures were displayed by the CYLview visualization program. [21]

10.1 Catalytic cycle in both the C_α and C_β pathways

The ring-opening of the epoxide might take place at the α or β carbon, so both routes are considered in this work.

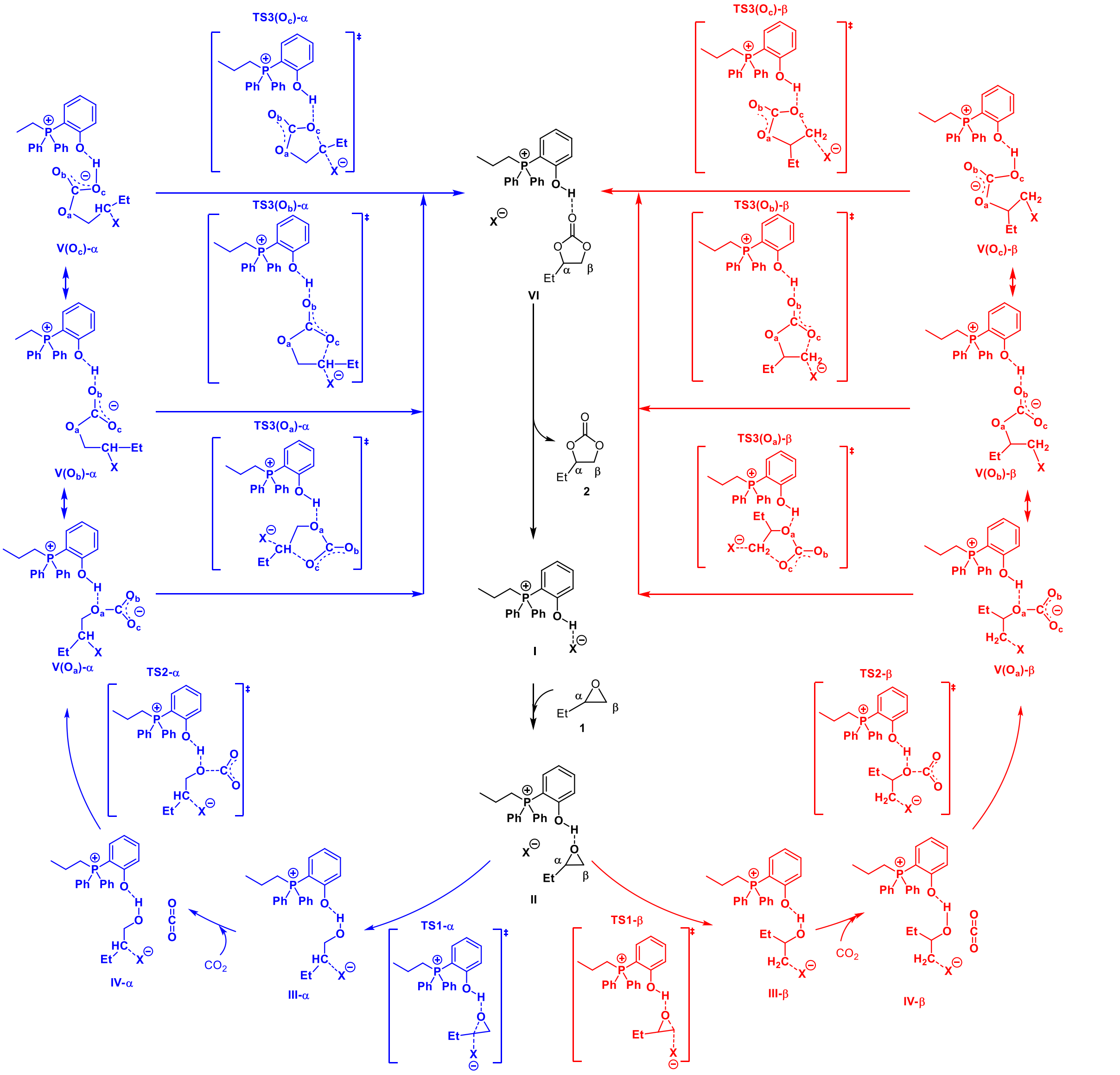

Scheme S5. Catalytic cycle in both the Cα and Cβ pathways.

10.2 Possibilities of the intermediates V and the transition states TS3 in the C$_{\alpha}$ pathway

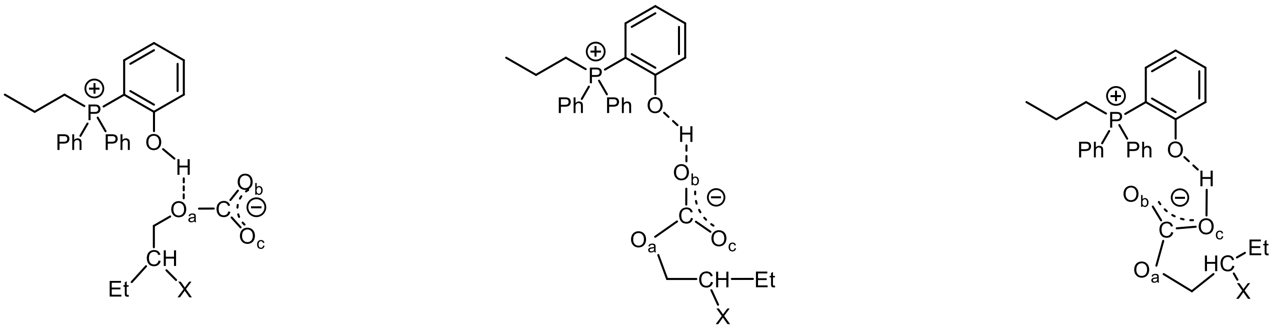

	V(O$_a$)-α	V(O$_b$)-α	V(O$_c$)-α
1) Br^-	2 kJ/mol	-14 kJ/mol	-8 kJ/mol
2) I^-	32 kJ/mol	14 kJ/mol	23 kJ/mol

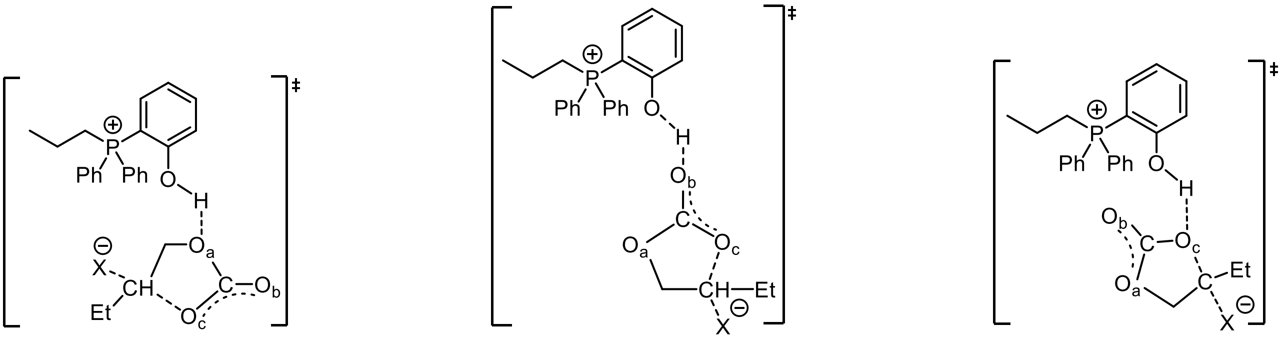

	TS3(O$_a$)-α	TS3(O$_b$)-α	TS3(O$_c$)-α
1) Br^-	98 kJ/mol	74 kJ/mol	84 kJ/mol
2) I^-	105 kJ/mol	77 kJ/mol	82 kJ/mol

Figure S16. Three possibilities of the intermediates **V** and the transition states **TS3** in the C$_{\alpha}$ pathway.

The structures of O$_b$ hydrogen bonding interaction has the lowest energy for both catalysts **7** and **8**.

10.3 Possibilities of the intermediates V and the transition states TS3 in the C_β pathway

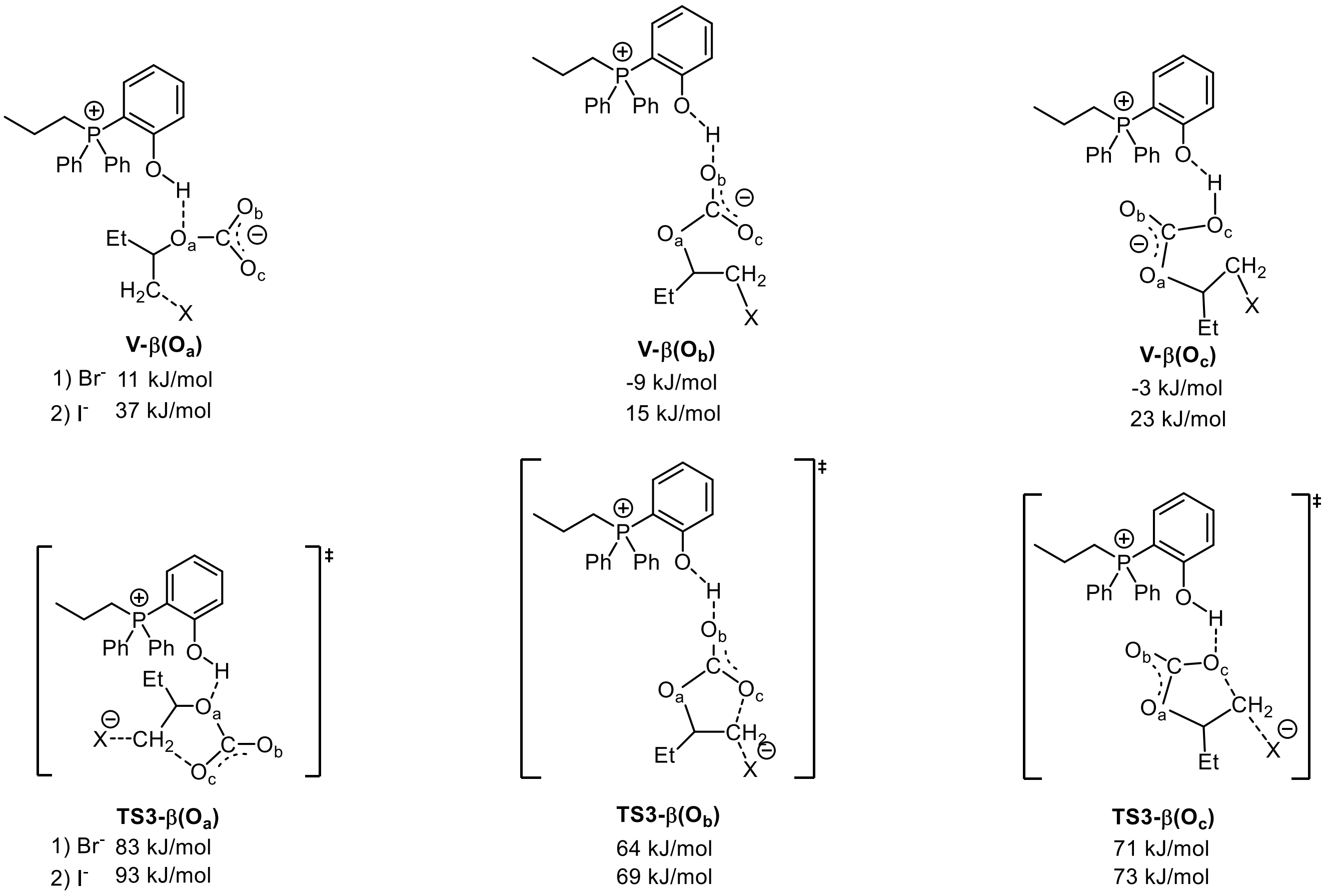

Figure S17. Three possibilities of the intermediates V and the transition states TS3 in the C_β pathway.

In the ring-closing step, the three possible pathways were calculated regarding that three oxygen (O_a/O_b/O_c) in carbonate may interact with OH group of phenol. For α route, the interaction of 2-iodobutyl carbonate through O_b and O_c with OH of phenol is more favored than through O_a by 18 and 9 kJ/mol, respectively. Therefore, the carboxylate anion can be highly stabilized through H-bond between OH of phenol and O_b/O_c of carboxylate. For the last step through α route, the ring-closing through O_a, O_b and O_c route from most stable V(O_b)-α-I has an energy barrier of 91, 63 and 68 kJ/mol, respectively.

10.4 Optimized structures for the transition states

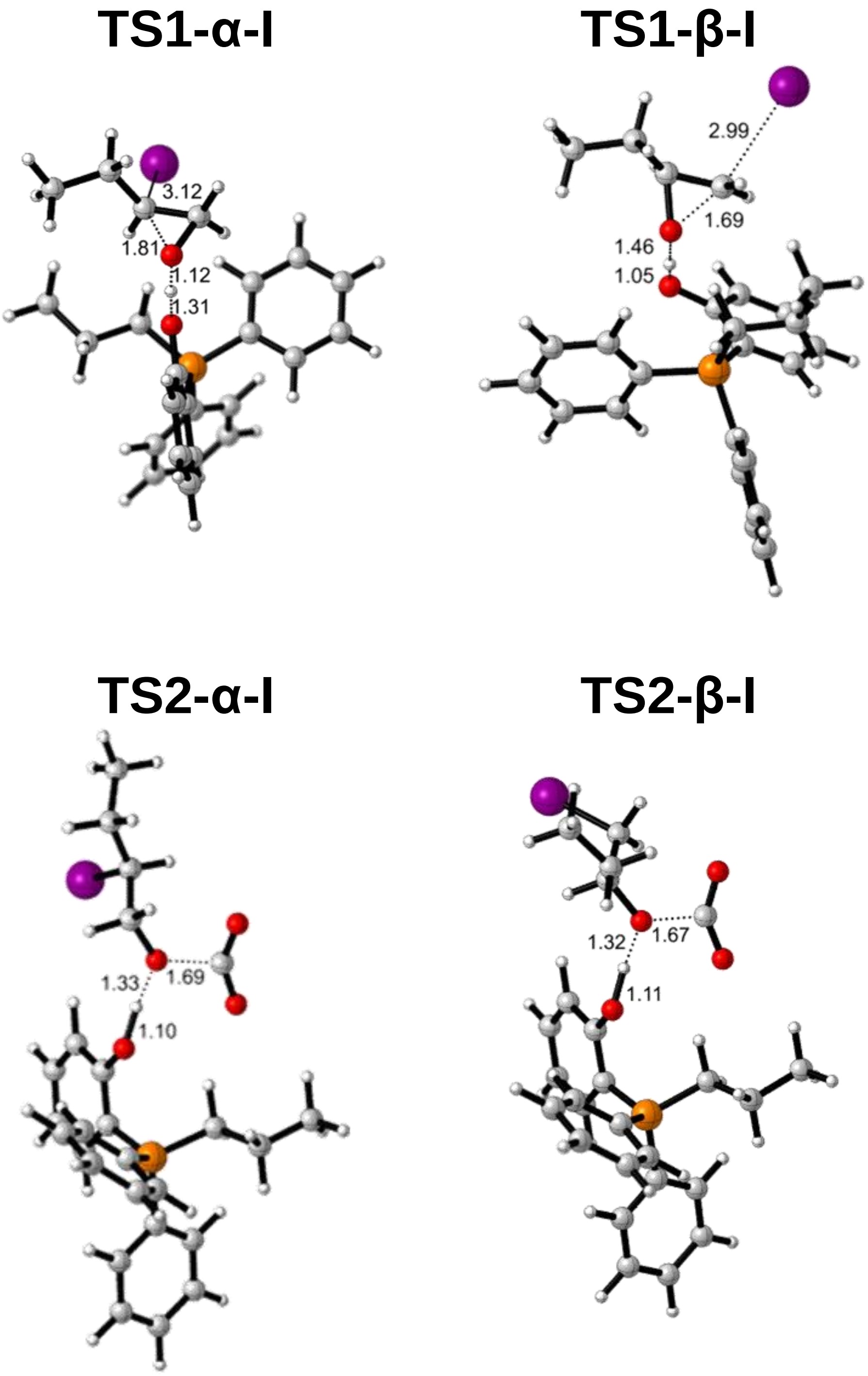

Figure S18. Optimized structures for transition states of TS1 and TS2 *via* α and β route for I^- as counterion.

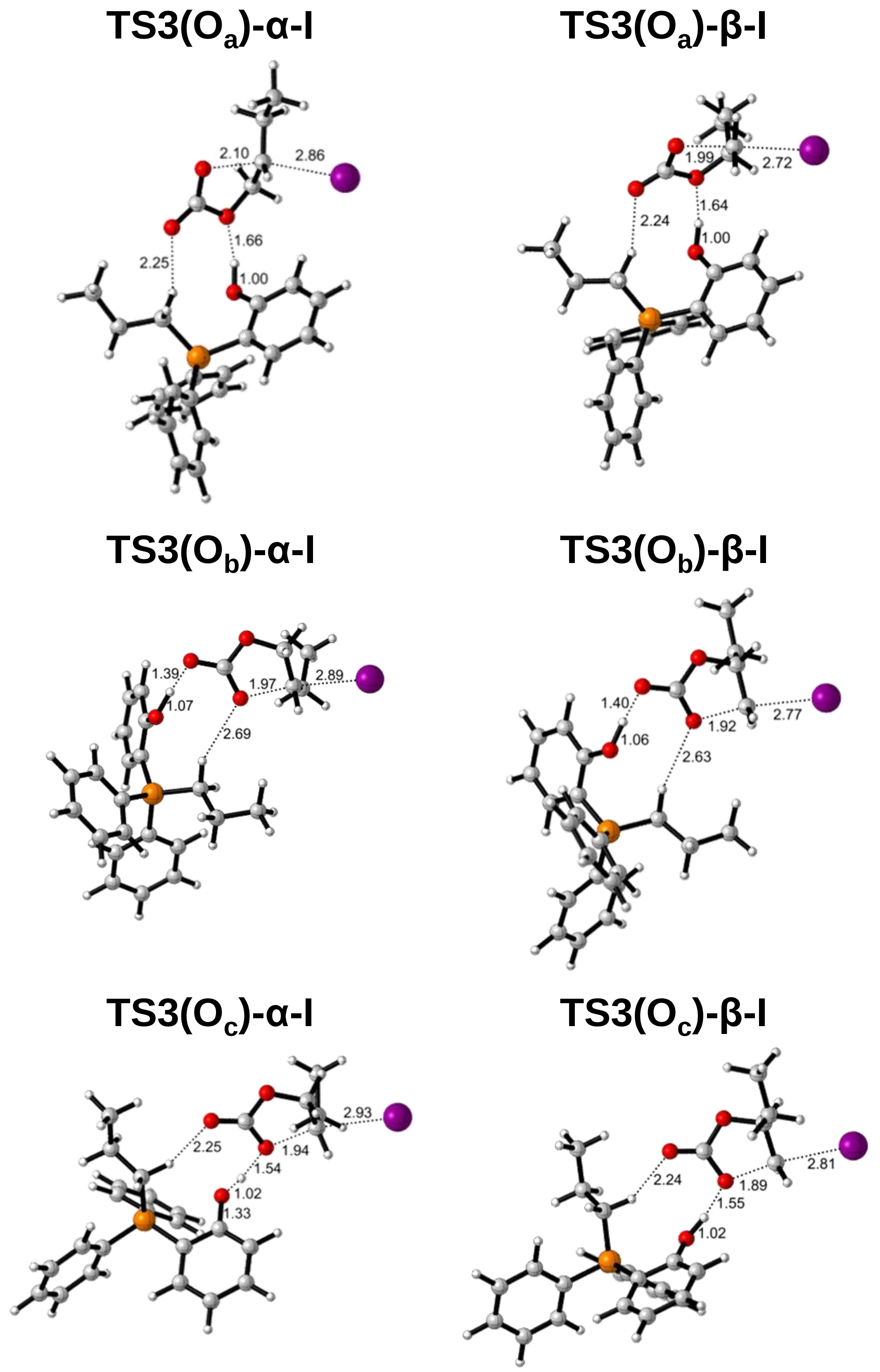

Figure S19. Optimized structures for transition states of TS3(O_a/O_b/O_c) *via* α and β route for I^- as counterion.

TS1-α-Br

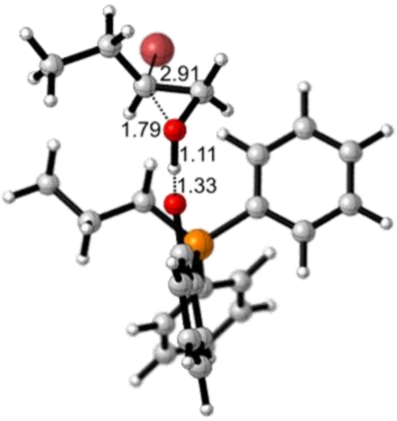

TS1-β-Br

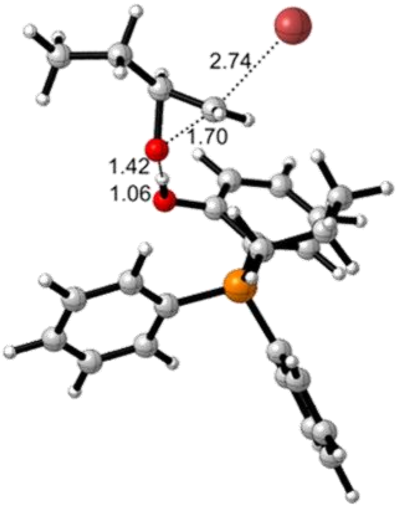

TS1-α-Br

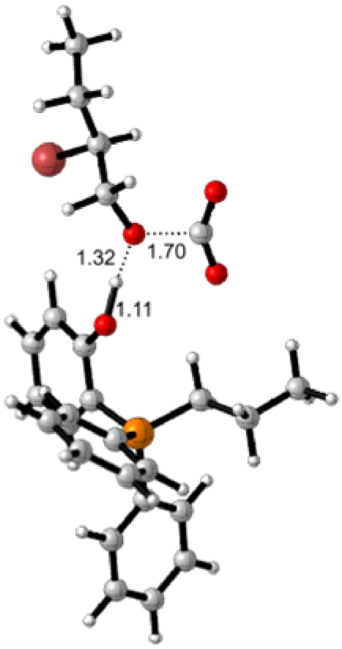

TS1-β-Br

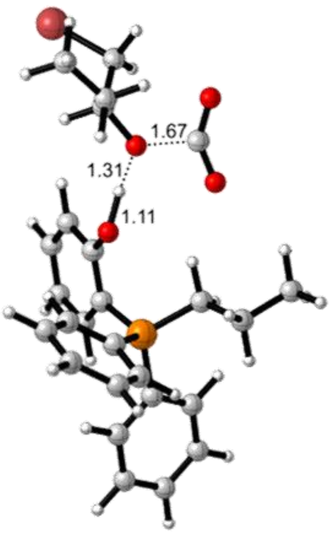

Figure S20. Optimized structures for transition states of TS1 and TS2 *via* α and β route for Br^- as counterion.

TS3(O$_a$)-α-Br

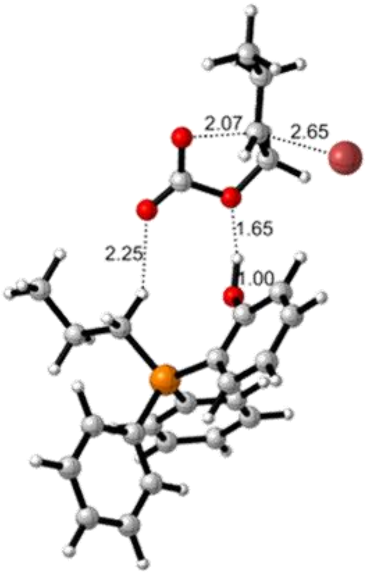

TS3(O$_a$)-β-Br

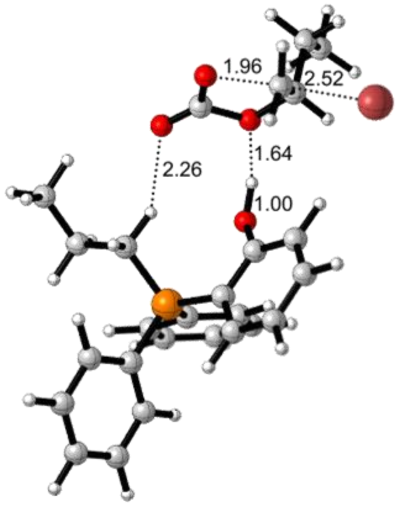

TS3(O$_b$)-α-Br

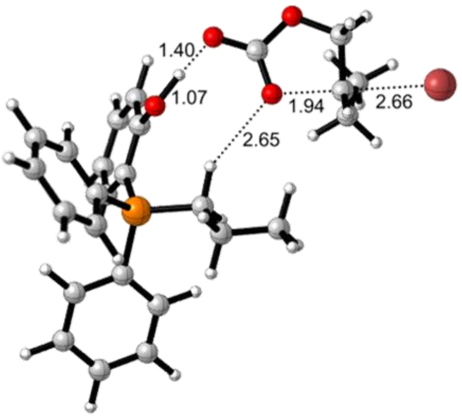

TS3(O$_b$)-β-Br

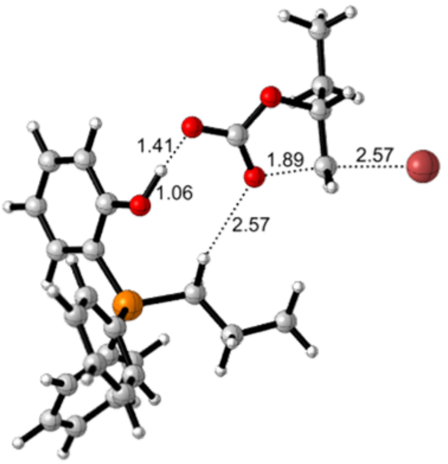

TS3(O$_c$)-α-Br

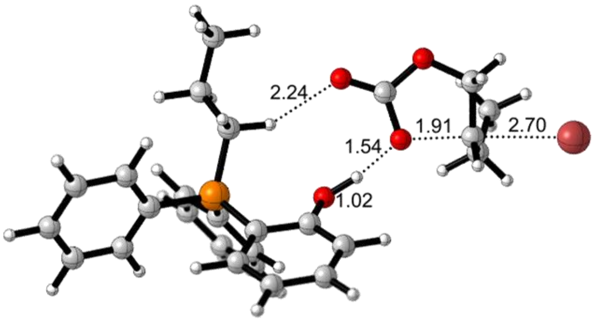

TS3(O$_c$)-β-Br

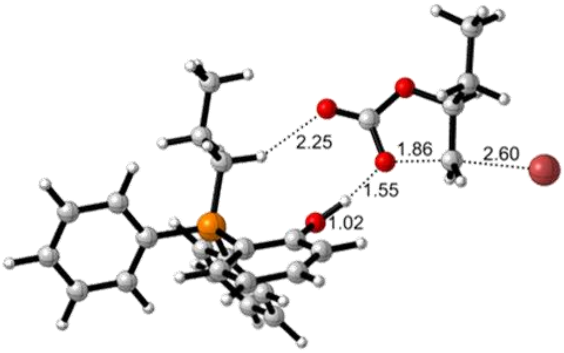

Figure S21. Optimized structures for transition states of TS3(O$_a$/O$_b$/O$_c$) *via* α and β route for Br$^-$ as counterion.

10.5 Gibbs free energy for the α and β routes with catalyst 7

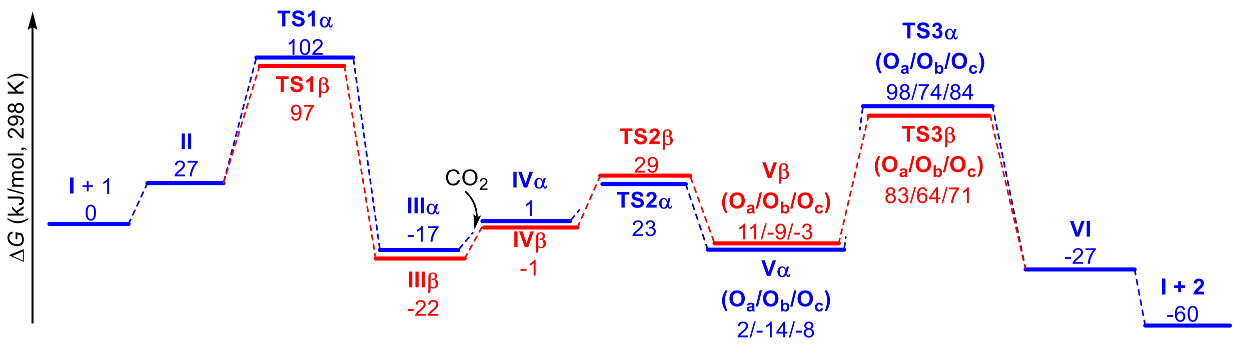

Figure S22. Gibbs free energy for the α and β routes. The β route is favored for both catalysts **7**, especially in the ring-opening step, the formation of the intermediate **III** and the transition state **TS3**.

10.6 Computational details

Table S6. Computed Energetic Data for All Optimized Structures

**CO_2**<br><br>E= -188.669936<br>ZPE= 0.011705<br>NImag= 0<br>Htot = -188.654674<br>Gtot = -188.67892<br>E(M06-2X/Def2-TZVP/SMD(BC))= -188.5924583	**1,2-epoxybutane**<br><br>E= -232.517842<br>ZPE= 0.113777<br>NImag= 0<br>Htot = -232.397426<br>Gtot = -232.432817<br>E(M06-2X/Def2-TZVP/SMD(BC))= -232.4095891
**1,2-butylene-carbonate**<br><br>E= -421.209118<br>ZPE= 0.130917<br>NImag= 0<br>Htot = -421.069639<br>Gtot = -421.110774<br>E(M06-2X/Def2-TZVP/SMD(BC))= -421.044019	
**I-I**<br><br>E= -1528.244331<br>ZPE= 0.373219<br>NImag= 0<br>Htot = -1527.84669<br>Gtot = -1527.927376<br>E(M06-2X/Def2-TZVP/SMD(BC))= -1527.690922	**I-Br**<br><br>E= -3804.625426<br>ZPE= 0.372653<br>NImag= 0<br>Htot = -3804.228297<br>Gtot = -3804.309001<br>E(M06-2X/Def2-TZVP/SMD(BC))= -3804.24325
**II-I**<br><br>E= -1760.767491<br>ZPE= 0.489438<br>NImag= 0<br>Htot = -1760.245765<br>Gtot = -1760.347337<br>E(M06-2X/Def2-TZVP/SMD(BC))= -1760.116808	**II-Br**<br><br>E= -4037.145716<br>ZPE= 0.489429<br>NImag= 0<br>Htot = -4036.624128<br>Gtot = -4036.723899<br>E(M06-2X/Def2-TZVP/SMD(BC))= -4036.66011
**TS1-α-I**<br><br>E= -1760.750745<br>ZPE= 0.485783<br>NImag= 1 (-458.7127)	**TS1-α-Br**<br><br>E= -4037.129357<br>ZPE= 0.486007<br>NImag= 1 (-423.8905)

Htot = -1760.233286<br>Gtot = -1760.332119<br>E(M06-2X/Def2-TZVP/SMD(BC))= -1760.080556	Htot = -4036.611758<br>Gtot = -4036.709668<br>E(M06-2X/Def2-TZVP/SMD(BC))= -4036.629357
**TS1-β-I**<br><br>E= -1760.733271<br>ZPE= 0.48667<br>NImag= 1 (-379.7353)<br>Htot = -1760.214872<br>Gtot = -1760.315751<br>E(M06-2X/Def2-TZVP/SMD(BC))= -1760.08714	**TS1-β-Br**<br><br>E= -4037.109901<br>ZPE= 0.486317<br>NImag= 1 (-389.9174)<br>Htot = -4036.591963<br>Gtot = -4036.691483<br>E(M06-2X/Def2-TZVP/SMD(BC))= -4036.629826
**III-α-I**<br><br>E= -1760.792648<br>ZPE= 0.489286<br>NImag= 0<br>Htot = -1760.271308<br>Gtot = -1760.373562<br>E(M06-2X/Def2-TZVP/SMD(BC))= -1760.110022	**III-α-Br**<br><br>E= -4037.175971<br>ZPE= 0.48983<br>NImag= 0<br>Htot = -4036.654246<br>Gtot = -4036.75516<br>E(M06-2X/Def2-TZVP/SMD(BC))= -4036.67578
**III-β-I**<br><br>E= -1760.795184<br>ZPE= 0.489111<br>NImag= 0<br>Htot = -1760.27414<br>Gtot = -1760.375286<br>E(M06-2X/Def2-TZVP/SMD(BC))= -1760.113095	**III-β-Br**<br><br>E= -4037.177007<br>ZPE= 0.489409<br>NImag= 0<br>Htot = -4036.655694<br>Gtot = -4036.756527<br>E(M06-2X/Def2-TZVP/SMD(BC))= -4036.677323
**IV-α-I**<br><br>E= -1949.463259<br>ZPE= 0.501239<br>NImag= 0<br>Htot = -1948.925655<br>Gtot = -1949.04126<br>E(M06-2X/Def2-TZVP/SMD(BC))= -1948.706197	**IV-α-Br**<br><br>E= -4225.846495<br>ZPE= 0.501934<br>NImag= 0<br>Htot = -4225.308413<br>Gtot = -4225.4224<br>E(M06-2X/Def2-TZVP/SMD(BC))= -4225.270483
**IV-β-I**<br><br>E= -1949.462016<br>ZPE= 0.501219<br>NImag= 0<br>Htot = -1948.924428<br>Gtot = -1949.040275<br>E(M06-2X/Def2-TZVP/SMD(BC))= -1948.703415	**IV-β-Br**<br><br>E= -4225.844498<br>ZPE= 0.501701<br>NImag= 0<br>Htot = -4225.306512<br>Gtot = -4225.422991<br>E(M06-2X/Def2-TZVP/SMD(BC))= -4225.268615
**TS2-α-I**<br><br>E= -1949.441402<br>ZPE= 0.500277<br>NImag= 1 (-227.0785)<br>Htot = -1948.907064<br>Gtot = -1949.011702<br>E(M06-2X/Def2-TZVP/SMD(BC))= -1948.703912	**TS2-α-Br**<br><br>E= -4225.824917<br>ZPE= 0.500436<br>NImag= 1 (-242.1395)<br>Htot = -4225.290423<br>Gtot = -4225.395168<br>E(M06-2X/Def2-TZVP/SMD(BC))= -4225.267932
**TS2-β-I**<br><br>E= -1949.436045<br>ZPE= 0.500018<br>NImag= 1 (-244.4133)<br>Htot = -1948.901941<br>Gtot = -1949.006682<br>E(M06-2X/Def2-TZVP/SMD(BC))= -1948.699436	**TS2-β-Br**<br><br>E= -4225.81844<br>ZPE= 0.500212<br>NImag= 1 (-251.4355)<br>Htot = -4225.284139<br>Gtot = -4225.389965<br>E(M06-2X/Def2-TZVP/SMD(BC))=
**V(O$_a$)-α-I**<br><br>E= -1949.441829<br>ZPE= 0.502647<br>NImag= 0<br>Htot = -1948.904708<br>Gtot = -1949.010917<br>E(M06-2X/Def2-TZVP/SMD(BC))= -1948.712793	**V(O$_a$)-α-Br**<br><br>E= -4225.825503<br>ZPE= 0.503232<br>NImag= 0<br>Htot = -4225.287905<br>Gtot = -4225.393969<br>E(M06-2X/Def2-TZVP/SMD(BC))= -4225.2776
**V(O$_a$)-β-I**	**V(O$_a$)-β-Br**

E= -1949.439993<br>ZPE= 0.502293<br>NImag= 0<br>Htot = -1948.903179<br>Gtot = -1949.009269<br>E(M06-2X/Def2-TZVP/SMD(BC))= -1948.710736	E= -4225.822082<br>ZPE= 0.503013<br>NImag= 0<br>Htot = -4225.284728<br>Gtot = -4225.38959<br>E(M06-2X/Def2-TZVP/SMD(BC))= -4225.275006
**V(O$_b$)-α-I**<br><br>E= -1949.463759<br>ZPE= 0.504037<br>NImag= 0<br>Htot = -1948.925536<br>Gtot = -1949.031883<br>E(M06-2X/Def2-TZVP/SMD(BC))= -1948.720581	**V(O$_b$)-α-Br**<br><br>E= -4225.846594<br>ZPE= 0.504511<br>NImag= 0<br>Htot = -4225.308047<br>Gtot = -4225.413237<br>E(M06-2X/Def2-TZVP/SMD(BC))= -4225.285435
**V(O$_b$)-β-I**<br><br>E= -1949.461965<br>ZPE= 0.50351<br>NImag= 0<br>Htot = -1948.924107<br>Gtot = -1949.031019<br>E(M06-2X/Def2-TZVP/SMD(BC))= -1948.719353	**V(O$_b$)-β-Br**<br><br>E= -4225.844013<br>ZPE= 0.504068<br>NImag= 0<br>Htot = -4225.305759<br>Gtot = -4225.411187<br>E(M06-2X/Def2-TZVP/SMD(BC))= -4225.283141
**V(O$_c$)-α-I**<br><br>E= -1949.461006<br>ZPE= 0.503632<br>NImag= 0<br>Htot = -1948.92321<br>Gtot = -1949.029527<br>E(M06-2X/Def2-TZVP/SMD(BC))= -1948.718354	**V(O$_c$)-α-Br**<br><br>E= -4225.84384<br>ZPE= 0.504263<br>NImag= 0<br>Htot = -4225.305609<br>Gtot = -4225.410664<br>E(M06-2X/Def2-TZVP/SMD(BC))= -4225.282856
**V(O$_c$)-β-I**<br><br>E= -1949.459152<br>ZPE= 0.503005<br>NImag= 0<br>Htot = -1948.921807<br>Gtot = -1949.028572<br>E(M06-2X/Def2-TZVP/SMD(BC))= -1948.715863	**V(O$_c$)-β-Br**<br><br>E= -4225.841184<br>ZPE= 0.503752<br>NImag= 0<br>Htot = -4225.303302<br>Gtot = -4225.409081<br>E(M06-2X/Def2-TZVP/SMD(BC))= -4225.279868
**TS3(O$_a$)-α-I**<br><br>E= -1949.418173<br>ZPE= 0.504029<br>NImag= 1 (-343.7945)<br>Htot = -1948.880342<br>Gtot = -1948.983847<br>E(M06-2X/Def2-TZVP/SMD(BC))= -1948.688249	**TS3(O$_a$)-α-Br**<br><br>E= -4225.798659<br>ZPE= 0.50431<br>NImag= 1 (-364.9227)<br>Htot = -4225.260677<br>Gtot = -4225.363199<br>E(M06-2X/Def2-TZVP/SMD(BC))= -4225.244829
**TS3(O$_a$)-β-I**<br><br>E= -1949.420797<br>ZPE= 0.50421<br>NImag= 1 (-401.1077)<br>Htot = -1948.882934<br>Gtot = -1948.986541<br>E(M06-2X/Def2-TZVP/SMD(BC))= -1948.692933	**TS3(O$_a$)-β-Br**<br><br>E= -4225.800434<br>ZPE= 0.504563<br>NImag= 1 (-412.4115)<br>Htot = -4225.262331<br>Gtot = -4225.365136<br>E(M06-2X/Def2-TZVP/SMD(BC))= -4225.250347
**TS3(O$_b$)-α-I**<br><br>E= -1949.425331<br>ZPE= 0.503454<br>NImag= 1 (-362.263)<br>Htot = -1948.88832<br>Gtot = -1948.992635<br>E(M06-2X/Def2-TZVP/SMD(BC))= -1948.697348	**TS3(O$_b$)-α-Br**<br><br>E= -4225.80489<br>ZPE= 0.50366<br>NImag= 1 (-375.8347)<br>Htot = -4225.26771<br>Gtot = -4225.372053<br>E(M06-2X/Def2-TZVP/SMD(BC))= -4225.251456
**TS3(O$_b$)-β-I**<br><br>E= -1949.432142<br>ZPE= 0.50327<br>NImag= 1 (-398.6086)	**TS3(O$_b$)-β-Br**<br><br>E=<br>ZPE=<br>NImag= 1 ()

Htot = -1948.89532<br>Gtot = -1948.999362<br>E(M06-2X/Def2-TZVP/SMD(BC))= -1948.700612	Htot =<br>Gtot =<br>E(M06-2X/Def2-TZVP/SMD(BC))=
**TS3(O_c)-α-I**<br><br>E= -1949.420156<br>ZPE= 0.504259<br>NImag= 1 (-382.9789)<br>Htot = -1948.882181<br>Gtot = -1948.987047<br>E(M06-2X/Def2-TZVP/SMD(BC))= -1948.69602	**TS3(O_c)-α-Br**<br><br>E= -4225.811506<br>ZPE= 0.503595<br>NImag= 1 (-410.2911)<br>Htot = -4225.274479<br>Gtot = -4225.377617<br>E(M06-2X/Def2-TZVP/SMD(BC))= -4225.256356
**TS3(O_c)-β-I**<br><br>E= -1948.698968<br>ZPE= 0.504029<br>NImag= 1 (-419.9629)<br>Htot = -1948.889065<br>Gtot = -1948.993813<br>E(M06-2X/Def2-TZVP/SMD(BC))= -1949.426761	**TS3(O_c)-β-Br**<br><br>E= -4225.799397<br>ZPE= 0.504535<br>NImag= 1 (-393.0968)<br>Htot = -4225.261271<br>Gtot = -4225.365034<br>E(M06-2X/Def2-TZVP/SMD(BC))= -4225.249021
**VI-I**<br><br>E= -1949.466678<br>ZPE= 0.506382<br>NImag= 0<br>Htot = -1948.926069<br>Gtot = -1949.031632<br>E(M06-2X/Def2-TZVP/SMD(BC))= -1948.74641	**VI-Br**<br><br>E= -4225.845741<br>ZPE= 0.506389<br>NImag= 0<br>Htot = -4225.305176<br>Gtot = -4225.410397<br>E(M06-2X/Def2-TZVP/SMD(BC))= -4225.292542

Table S7. The Cartesian Coordinates (xyz) for All Optimized Structures

**CO_2**<br><br>C 0.00000000 0.00000000 0.00000000<br>O 0.00000000 0.00000000 1.15968600<br>O 0.00000000 0.00000000 -1.15968600	**1,2-epoxybutane**<br><br>C -0.36716600 -0.09223000 0.44517100<br>C -1.36163700 0.78636700 -0.17495300<br>O -1.61552400 -0.61356200 -0.02240400<br>H -1.21284400 1.11842700 -1.19900500<br>H -1.97446600 1.43816800 0.44086300<br>C 0.87714700 -0.54289100 -0.27314700<br>H 1.08123600 -1.58211300 0.00311800<br>H 0.68221600 -0.53784500 -1.34854400<br>H -0.29101400 -0.06563600 1.53187200<br>C 2.08972200 0.32879000 0.05699000<br>H 2.30138100 0.32565000 1.12884400<br>H 2.98162400 -0.03381100 -0.45605200<br>H 1.92766700 1.36544200 -0.24622800
**1,2-butylene-carbonate**<br><br>O 2.39573200 -1.24317200 -0.13876900<br>C 1.50735500 -0.46362400 -0.02120400<br>O 1.65997500 0.88563500 0.00279400<br>O 0.19821500 -0.78986500 0.10757100<br>C -0.58662200 0.38964100 0.38883300<br>C 0.37373500 1.51595200 -0.00620100<br>H 0.38445100 2.34371100 0.70009800<br>H 0.17325000 1.89450900 -1.01171100<br>C -1.89692400 0.35824900 -0.37223700<br>H -0.77386600 0.39816700 1.46745300<br>H -2.39085100 1.32360000 -0.21607300<br>C -2.82436000 -0.77793600 0.05216200<br>H -1.67378600 0.28810100 -1.44082200<br>H -3.75244700 -0.75266000 -0.52035300<br>H -2.35370600 -1.74789900 -0.11015900<br>H -3.08352100 -0.70201500 1.11069000	
**I-I**<br><br>H -2.22878900 -0.19213900 -1.12197800<br>O -1.31866600 -0.27984200 -1.56367800<br>C -0.72731000 0.90006500 -1.79865100	**I-Br**<br><br>H -2.45723500 -0.05223400 -0.86109300<br>O -1.86177500 -0.05907800 -1.69379300<br>C -1.10233500 1.04060600 -1.78121600

C	0.50960300	1.15641200	-1.17680800	C	0.05187700	1.22687600	-0.98741800
C	-1.28691100	1.86502900	-2.63989200	C	-1.44200900	2.03124500	-2.71129300
C	1.16266800	2.37482200	-1.40763400	C	0.77170500	2.43013400	-1.08768500
C	-0.62428500	3.05962800	-2.86247600	C	-0.70552400	3.19599200	-2.81488600
C	0.60313600	3.31827000	-2.25294900	C	0.39837000	3.40923000	-1.98941300
H	2.09455300	2.60004400	-0.90921800	H	1.63298000	2.59957300	-0.45655400
H	-1.06842900	3.80177900	-3.51401000	H	-0.99958300	3.95399900	-3.53032000
H	1.11292000	4.25671000	-2.42494900	H	0.96303000	4.32988900	-2.05164900
H	-2.24275500	1.65527700	-3.10088200	H	-2.31872800	1.86016900	-3.32134800
P	1.04346000	-0.01121600	0.09106700	P	0.58385000	-0.02461100	0.19969100
C	0.00534800	0.22976600	1.56030100	C	-0.21939900	0.23462600	1.80359900
H	0.33779100	-0.48712000	2.31626800	H	0.14588400	-0.53955500	2.48401100
H	-1.01177300	-0.06607300	1.26510700	H	-1.28815700	0.03588100	1.62429600
C	-0.04116800	1.66155500	2.11140700	C	-0.07013500	1.63942000	2.39939100
H	0.96467300	2.02985600	2.33071600	H	0.98405600	1.89632900	2.53703700
H	-0.45904000	2.31942500	1.34756400	H	-0.48501200	2.36662400	1.69899200
C	-0.91002100	1.73226000	3.36751400	C	-0.81151500	1.74725700	3.73236400
H	-1.92317900	1.39040800	3.15157800	H	-1.87108400	1.52375500	3.60119100
H	-0.50221600	1.11016600	4.16851500	H	-0.40633000	1.05059700	4.47058000
C	0.97617700	-1.77032300	-0.36256400	C	0.27958800	-1.73427700	-0.32519900
C	2.05863400	-2.37561400	-1.00637500	C	1.20055100	-2.38460000	-1.15209400
C	-0.15072100	-2.53540800	-0.04190600	C	-0.86299000	-2.41411800	0.10805000
C	2.01389700	-3.72525100	-1.33041500	C	0.98048800	-3.69804900	-1.54232200
H	2.94468200	-1.80733100	-1.25142300	H	2.09506700	-1.87989700	-1.48905600
C	-0.18078900	-3.88625400	-0.35756400	C	-1.06944400	-3.73042900	-0.28228900
H	-1.01977800	-2.08709000	0.42033600	H	-1.61994300	-1.91804000	0.70341000
C	0.89640500	-4.48298500	-1.00265700	C	-0.15284500	-4.37381100	-1.10465400
H	2.85613800	-4.18266100	-1.83330900	H	1.69768700	-4.19275300	-2.18457500
H	-1.05987800	-4.46604000	-0.10868500	H	-1.96290400	-4.24318000	0.04908600
H	0.86367400	-5.53607400	-1.25180800	H	-0.32234700	-5.39921800	-1.40821200
C	2.78128400	0.33390500	0.49710800	C	2.38815900	0.12738300	0.39283400
C	3.72023600	0.59407900	-0.50784300	C	3.21423900	0.40233600	-0.70231400
C	3.21528700	0.25086700	1.82276900	C	2.97443300	-0.12666500	1.63572500
C	5.05989300	0.77369500	-0.18929700	C	4.59461500	0.42606800	-0.55295500
H	3.40888700	0.66105300	-1.54163900	H	2.78239800	0.60627000	-1.67259800
C	4.55597200	0.43325100	2.13754800	C	4.35555600	-0.09855000	1.78194100
H	2.51390500	0.04163100	2.61830000	H	2.36008900	-0.34891700	2.49709000
C	5.47976400	0.69591600	1.13362500	C	5.16771300	0.17725100	0.68886100
H	5.77441800	0.97563200	-0.97679100	H	5.22130100	0.64115900	-1.40883000
H	4.87608500	0.37007900	3.16939600	H	4.79468900	-0.29245700	2.75191000
H	6.52381000	0.83915200	1.38075100	H	6.24379100	0.19925200	0.80416700
H	-0.96565900	2.75771500	3.73646800	H	-0.72274500	2.75530000	4.14094300
I	-3.80368300	-0.13584100	0.54931400	Br	-3.65648700	-0.03652300	0.81492400
**II-I**				**II-Br**			
C	-4.17888000	1.52931800	0.56997400	C	4.50275600	0.48554100	-0.90902700
C	-4.40703100	1.58237100	-0.87081600	C	4.68882000	1.40333900	0.21068800
O	-3.17867000	2.11219900	-0.30949200	O	3.55607200	1.56515200	-0.68230100
H	-5.05627700	2.34885000	-1.28236100	H	5.41493300	2.20660600	0.13034600
H	-4.28482200	0.66519900	-1.43801300	H	4.43000600	1.04420300	1.20164300
I	-2.95730500	-2.02003800	-0.68543100	Br	2.83723300	-1.18553800	1.92759600
C	-4.83753700	2.47241100	1.53778200	C	5.30756900	0.56651400	-2.17622600
H	-4.13146100	2.69983600	2.34282000	H	4.66515100	0.29481100	-3.01999700
H	-5.05119400	3.41642500	1.02906700	H	5.62053600	1.60160900	-2.33791100
H	-3.87663400	0.55647500	0.95164000	H	4.09137700	-0.48414000	-0.63829600
H	-1.63207300	1.47530900	-0.40754000	H	1.95071500	1.21727200	-0.33054900
O	-0.65216800	1.30885700	-0.32443100	O	0.95954700	1.10610100	-0.36637500
C	-0.11329900	0.73086300	-1.40387000	C	0.35282600	1.35443100	0.79864000
C	1.21947200	0.28602900	-1.30165200	C	-1.00702600	1.00308000	0.90798100
C	-0.79825300	0.56219800	-2.60973700	C	0.99221700	1.95170100	1.88819400
C	1.84977400	-0.29154600	-2.41489200	C	-1.70844200	1.28468500	2.09107600
C	-0.15969600	-0.01186800	-3.69267300	C	0.28455300	2.21656000	3.04449300
C	1.16689600	-0.43561000	-3.60695700	C	-1.06926500	1.89398900	3.15279300
H	2.87387600	-0.62854800	-2.34008700	H	-2.75309800	1.02061400	2.17449100
H	-0.70925600	-0.14786600	-4.61522000	H	0.79833100	2.66804400	3.88356400
H	1.65498300	-0.88707300	-4.45979200	H	-1.61250400	2.10424800	4.06395000
H	-1.83442900	0.86097300	-2.67203600	H	2.04718000	2.17209300	1.82024800
P	2.06027700	0.45712700	0.26571100	P	-1.78541300	0.16508300	-0.46432800

C	2.04593200	2.19479400	0.84685100	C	-1.61161000	1.11211800	-2.02354800
H	2.84259700	2.28367500	1.59064900	H	-2.38179400	0.73881100	-2.70429400
H	1.09721300	2.29994600	1.37408200	H	-0.64646200	0.80094500	-2.42466200
C	2.16169300	3.28230100	-0.22882300	C	-1.65442500	2.64165600	-1.91614400
H	3.07185600	3.15184100	-0.81856800	H	-2.58834600	2.97514700	-1.45808200
H	1.32861700	3.17990600	-0.92457300	H	-0.85303400	2.97066700	-1.25463200
C	2.14631500	4.68062700	0.38783000	C	-1.49140500	3.29933200	-3.28618500
H	1.22625700	4.85023900	0.95078900	H	-0.54262900	3.01515400	-3.74568000
H	2.98785400	4.82915000	1.06880200	H	-2.29423100	3.01022900	-3.96880800
C	1.34122200	-0.56389100	1.56638400	C	-1.11855500	-1.48565200	-0.74940400
C	0.21809200	-1.35495900	1.33031500	C	-0.04505200	-1.96966200	-0.00349800
C	1.93279500	-0.54133500	2.83817800	C	-1.69252200	-2.27618300	-1.75675700
C	-0.31223700	-2.12097900	2.36407200	C	0.45321100	-3.24262200	-0.26639700
H	-0.28649400	-1.38536900	0.37091500	H	0.45486600	-1.38799100	0.76458700
C	1.39780200	-1.30900700	3.85970600	C	-1.19063600	-3.54288100	-2.00652000
H	2.81208600	0.06183400	3.03056200	H	-2.53270100	-1.91278900	-2.33660800
C	0.27448000	-2.10038600	3.62132900	C	-0.11662900	-4.02644300	-1.25904900
H	-1.19644000	-2.71075500	2.15670000	H	1.30181000	-3.58191000	0.31407700
H	1.85558100	-1.29193200	4.84059700	H	-1.63504200	-4.15349300	-2.78223200
H	-0.14167900	-2.69790500	4.42289200	H	0.27404700	-5.01648300	-1.45940100
C	3.78060900	-0.08995800	0.07199900	C	-3.55029500	-0.04713600	-0.09288600
C	4.05108300	-1.46286800	0.07648600	C	-3.93595300	-1.09515800	0.75011600
C	4.83417400	0.81288000	-0.08718900	C	-4.52681600	0.80563400	-0.61241200
C	5.35149500	-1.91998800	-0.08463400	C	-5.27370600	-1.27843500	1.07126500
H	3.24716100	-2.17463900	0.21234200	H	-3.19173200	-1.77205600	1.14917400
C	6.13559400	0.35029800	-0.24187500	C	-5.86584400	0.61412000	-0.29314100
H	4.65265700	1.87825300	-0.08968000	H	-4.25558800	1.62098500	-1.26774600
C	6.39525200	-1.01459500	-0.24354100	C	-6.24018600	-0.42501900	0.54974600
H	5.54867500	-2.98405400	-0.08017800	H	-5.56049900	-2.09214200	1.72457100
H	6.94503700	1.05881600	-0.36084700	H	-6.61472500	1.27852400	-0.70440400
H	7.40955500	-1.37288300	-0.36416700	H	-7.28361400	-0.57236200	0.79741800
H	2.20689200	5.44466100	-0.38859100	H	-1.50516900	4.38653500	-3.19620600
C	-6.11870500	1.87896400	2.12803800	C	6.52416100	-0.36126200	-2.13561800
H	-5.91726300	0.93958300	2.64647900	H	6.22440700	-1.39946800	-1.97973700
H	-6.57030800	2.56696700	2.84453100	H	7.08290500	-0.31071000	-3.07160700
H	-6.85510200	1.67336600	1.34863400	H	7.20151700	-0.08886400	-1.32381600
**TS1-α-I**				**TS1-α-Br**			
C	3.40588900	1.25864900	0.16714700	C	-3.70584900	0.61180600	-0.11627600
C	3.03482900	1.22375400	1.56627100	C	-3.35717000	0.47556600	-1.51210700
O	2.54437100	2.51629800	1.13397900	O	-3.11309600	1.87957700	-1.22474300
H	3.82940300	1.31804600	2.30069800	H	-4.16501700	0.34670600	-2.22583100
H	2.25425400	0.53151500	1.86084100	H	-2.46292200	-0.08499700	-1.75608400
I	3.44396500	-1.79554100	-0.44620200	Br	-3.20756700	-2.12296500	0.74054300
C	4.71116200	1.78067100	-0.31744800	C	-5.08159000	0.91327100	0.35717700
H	5.36175400	0.90743600	-0.41896900	H	-5.51505200	-0.05938300	0.60880500
H	5.14830200	2.41063400	0.46106000	H	-5.66499000	1.31718300	-0.47367000
H	2.65017800	0.96204900	-0.53791700	H	-2.90026900	0.53712500	0.59061600
H	1.48533700	2.33652600	0.81429500	H	-2.04527500	1.92036800	-0.91917900
O	0.34038800	1.84176400	0.40730000	O	-0.82714900	1.62977200	-0.47613000
C	4.62295200	2.52533500	-1.64786400	C	-5.13013700	1.84032900	1.56990600
H	3.97327500	3.39830400	-1.56668100	H	-4.69815400	2.81507000	1.33739000
H	5.61063200	2.86534300	-1.96187100	H	-6.16072500	1.99428300	1.89233900
H	4.23008200	1.87344200	-2.43011500	H	-4.57901800	1.41028100	2.40805500
C	-0.80876700	2.43783100	0.48447600	C	0.25372400	2.32890500	-0.62340000
C	-1.98780400	1.68627500	0.20902900	C	1.50745300	1.72235700	-0.31852900
C	-0.97467000	3.79999900	0.82947600	C	0.27660700	3.67039600	-1.07320900
C	-3.25626400	2.27516700	0.30685900	C	2.70777400	2.42721200	-0.48486900
C	-2.23301300	4.36052400	0.90693200	C	1.47020100	4.34703200	-1.21888400
C	-3.38523600	3.60788300	0.65095500	C	2.69616000	3.73605900	-0.92993300
H	-4.14370200	1.68579700	0.11999300	H	3.65310300	1.94611500	-0.27302000
H	-2.32949700	5.40580100	1.17635900	H	1.45633600	5.37301600	-1.56783300
H	-4.36494800	4.05997900	0.72278500	H	3.62374800	4.27780700	-1.05450800
H	-0.09046900	4.38987200	1.03694800	H	-0.66596400	4.15062300	-1.30522200
P	-1.72133900	-0.04833100	-0.12130500	P	1.42572900	-0.00385400	0.13426900
C	-0.50590300	-0.41792300	-1.43409700	C	0.28001600	-0.40847500	1.49714200
H	-0.71125200	-1.44955500	-1.73438600	H	0.61643900	-1.37681800	1.87927000
H	0.48451400	-0.40758500	-0.97908800	H	-0.70881000	-0.56846700	1.06547700

C	-0.53445800	0.51148400	-2.65224900
H	-1.53629600	0.54782100	-3.08677100
H	-0.29023700	1.52493600	-2.32978500
C	0.45945800	0.04801300	-3.71840200
H	1.46969200	-0.02462100	-3.31254900
H	0.19283800	-0.93804500	-4.10487700
C	-3.29276700	-0.78616600	-0.67105500
C	-3.99364400	-0.21721400	-1.74204100
C	-3.77065300	-1.96782400	-0.09975400
C	-5.14701200	-0.81892600	-2.22556000
H	-3.64794200	0.70157800	-2.19569100
C	-4.92711400	-2.56608100	-0.58746500
H	-3.24260400	-2.42477900	0.72562500
C	-5.61596800	-1.99473800	-1.64941000
H	-5.67944200	-0.36776100	-3.05294600
H	-5.28646900	-3.48094500	-0.13428200
H	-6.51584200	-2.46268400	-2.02769200
C	-1.20783700	-0.89215200	1.39505100
C	-0.15786200	-1.81259200	1.40436900
C	-1.88833200	-0.60203100	2.58331200
C	0.20023600	-2.43982000	2.59319100
H	0.42288000	-2.02795900	0.51717700
C	-1.53095400	-1.23911500	3.76213300
H	-2.68579100	0.13022400	2.59035600
C	-0.48680500	-2.16063000	3.76710800
H	1.03310500	-3.13056600	2.58921600
H	-2.05870200	-1.00797500	4.67853800
H	-0.20255700	-2.64971600	4.69038300
H	0.47457000	0.74478500	-4.55841500

C	0.20068200	0.61930700	2.63123200
H	1.19272500	0.82019400	3.04341800
H	-0.17241200	1.56199100	2.22792400
C	-0.71926700	0.12781600	3.75014800
H	-1.71066200	-0.11737200	3.36615300
H	-0.32128800	-0.77400500	4.22054100
C	3.07692600	-0.53893100	0.68851900
C	3.72895700	0.15027800	1.71880900
C	3.67129900	-1.68661500	0.15972500
C	4.94857500	-0.30156900	2.20374700
H	3.29246800	1.04593700	2.13957700
C	4.89363600	-2.13446100	0.64791500
H	3.18191500	-2.23488700	-0.63287400
C	5.53341900	-1.44482500	1.66929300
H	5.44208900	0.24139800	2.99953300
H	5.34249700	-3.02508900	0.22739300
H	6.48480200	-1.79520600	2.04878400
C	0.98317500	-0.99959100	-1.31234100
C	0.02848600	-2.01629100	-1.24017600
C	1.62468100	-0.73195100	-2.52746500
C	-0.26905800	-2.76301900	-2.37608000
H	-0.52990400	-2.21864100	-0.33464900
C	1.32664400	-1.48656400	-3.65215300
H	2.34571900	0.07274300	-2.59781000
C	0.38039700	-2.50558700	-3.57602100
H	-1.02640000	-3.53315700	-2.31079800
H	1.82380300	-1.27253500	-4.58960300
H	0.14299900	-3.08890400	-4.45691700
H	-0.82538100	0.89069300	4.52366800

TS1-β-I

C	2.67793800	1.66094300	-0.18423100
C	2.54731900	0.27221500	0.19228300
O	1.28096700	1.39010700	0.09385800
H	2.63871600	-0.01679000	1.22330900
H	2.39808500	-0.48911800	-0.55199600
I	5.24358200	-1.01298100	0.21096800
C	3.34513200	2.65785100	0.72458100
H	4.42391900	2.52530800	0.60964400
H	3.10774200	2.40109700	1.76078300
H	2.85979600	1.84863200	-1.24278800
H	0.46140500	1.16597700	-1.09339200
O	-0.08842900	0.98356000	-1.96345800
C	2.93861400	4.09904400	0.42381100
H	1.85948500	4.23455200	0.52740200
H	3.43175500	4.79436700	1.10512800
H	3.21360900	4.38354300	-0.59477900
C	-0.47157700	-0.28280200	-2.11764500
C	-1.30650200	-0.96553400	-1.19734900
C	-0.05219700	-0.97190100	-3.26671000
C	-1.59488700	-2.32882300	-1.40376500
C	-0.37048900	-2.30027900	-3.46279500
C	-1.12954800	-2.99525400	-2.51882400
H	-2.19753500	-2.86680600	-0.68484400
H	-0.00862500	-2.81108500	-4.34625200
H	-1.35509100	-4.04389000	-2.65674300
H	0.56420900	-0.43054500	-3.97185300
P	-1.99661800	-0.14367700	0.24188200
C	-0.91108700	-0.31585500	1.69116900
H	-1.40105700	0.17678000	2.53512900
H	-0.01845200	0.26847400	1.45172000
C	-0.52376100	-1.76007100	2.04252400
H	-1.41902100	-2.36567500	2.20556500
H	0.00841300	-2.20638000	1.20077000
C	0.36214600	-1.81231100	3.28739600
H	1.28984000	-1.25829700	3.13789800
H	-0.14783100	-1.39134400	4.15732600
C	-3.60867700	-0.89763600	0.62301200

TS1-β-Br

C	-3.32699300	1.32088400	0.25404100
C	-2.82729700	0.11150100	-0.35998200
O	-1.94919800	1.55028000	-0.12158100
H	-2.90541900	-0.01650900	-1.42312000
H	-2.37880000	-0.65819200	0.23894400
Br	-4.66356600	-1.91406800	-0.53245100
C	-4.34698000	2.18295800	-0.44115800
H	-5.31932700	1.69985600	-0.31319500
H	-4.13353200	2.17998200	-1.51347100
H	-3.46283800	1.28088300	1.33576500
H	-1.00916000	1.50506800	0.94439700
O	-0.31546600	1.51809400	1.74534100
C	-4.37617400	3.61351900	0.09315000
H	-3.40469500	4.09797400	-0.02606400
H	-5.11848100	4.21342200	-0.43604500
H	-4.63157200	3.63377900	1.15558700
C	0.08473800	0.32733600	2.18181400
C	0.90129700	-0.54733700	1.42076000
C	-0.28401000	-0.07753200	3.47582200
C	1.22788500	-1.81696200	1.93559200
C	0.06861800	-1.31644100	3.97096000
C	0.81422500	-2.20490700	3.19366800
H	1.81744100	-2.50359900	1.34356800
H	-0.25239000	-1.60659500	4.96362800
H	1.06835800	-3.18686100	3.56872800
H	-0.88353100	0.61074000	4.05665300
P	1.51230800	-0.09991100	-0.20768200
C	0.38796000	-0.68795700	-1.51365400
H	0.81185100	-0.38869200	-2.47583600
H	-0.53096700	-0.11421400	-1.38392800
C	0.08497200	-2.19446300	-1.48203900
H	1.01448100	-2.75945500	-1.59009600
H	-0.33544500	-2.46196600	-0.51102900
C	-0.90066600	-2.60052900	-2.57872700
H	-1.88925000	-2.17191400	-2.40680700
H	-0.54703100	-2.29427800	-3.56677600
C	3.14081400	-0.87352400	-0.45038400

C	-4.04682300	-0.96834100	1.94857100
C	-4.46789300	-1.31428800	-0.39922900
C	-5.31345500	-1.45439900	2.24550100
H	-3.40855600	-0.64565400	2.75916700
C	-5.73456400	-1.79599800	-0.09775400
H	-4.14727600	-1.27656600	-1.43112400
C	-6.15923400	-1.86847500	1.22388100
H	-5.63681100	-1.50901500	3.27677500
H	-6.38804000	-2.11801700	-0.89808300
H	-7.14582600	-2.24777600	1.45675100
C	-2.32683000	1.61281400	-0.05161000
C	-1.67375000	2.60760900	0.67689900
C	-3.28118300	1.97099600	-1.00851400
C	-1.97893800	3.94452400	0.45108600
H	-0.90437400	2.35847500	1.39166200
C	-3.57464400	3.30656300	-1.23357500
H	-3.79400200	1.21358300	-1.58612200
C	-2.92628400	4.29584800	-0.50077400
H	-1.46309000	4.71026000	1.01537200
H	-4.30878500	3.57530200	-1.98181800
H	-3.15668800	5.33866900	-0.67712300
H	0.63025400	-2.84286100	3.52216900

C	3.54761300	-1.23935200	-1.73693800
C	4.03904100	-1.01674400	0.61206800
C	4.82224200	-1.74581800	-1.95394400
H	2.87789100	-1.13251800	-2.57884200
C	5.31428700	-1.51954100	0.38937000
H	3.74321100	-0.74848100	1.61691200
C	5.70760200	-1.88646700	-0.89216600
H	5.12082100	-2.03109300	-2.95422500
H	5.99845100	-1.62764000	1.22094800
H	6.70044900	-2.28230600	-1.06273700
C	1.77399000	1.68180300	-0.41266300
C	0.93472600	2.45000700	-1.22138300
C	2.85159300	2.28913500	0.23862500
C	1.17739600	3.80889100	-1.37649100
H	0.06949200	2.01520100	-1.69869500
C	3.08266900	3.64751600	0.08470800
H	3.51464500	1.70866900	0.86571100
C	2.24790200	4.40881800	-0.72651500
H	0.51647000	4.39830400	-1.99823300
H	3.91582000	4.11071400	0.59707800
H	2.43009900	5.46904000	-0.84708200
H	-1.02422600	-3.68402200	-2.58802600

III-α-I

C	3.96692600	0.30084300	0.53032700
C	3.23529000	-0.77546400	1.33681800
O	2.40717800	-0.17830200	2.30696300
H	3.96564300	-1.40452900	1.85376000
H	2.66149600	-1.41637600	0.66067500
I	5.02203600	-0.70341200	-1.14107300
C	4.92345100	1.14277500	1.35560000
H	5.74358700	0.51412400	1.71383800
H	4.35203200	1.44014300	2.24112100
H	3.24249000	0.92149900	0.00505900
H	1.55315100	0.06722100	1.87356700
O	0.11627700	0.40978100	0.99803500
C	5.47134000	2.38560600	0.66143800
H	4.66083300	3.03843700	0.32767500
H	6.10018700	2.96100100	1.34361000
H	6.07316100	2.12651100	-0.21110400
C	-0.67928000	1.35218900	1.32277600
C	-2.01996300	1.35735000	0.80767700
C	-0.34160900	2.43726200	2.18931100
C	-2.94702600	2.35765100	1.14805300
C	-1.26325700	3.40875800	2.50019700
C	-2.57470500	3.38428600	1.98887700
H	-3.95535100	2.32309500	0.75554700
H	-0.97284000	4.21689100	3.16240600
H	-3.28146700	4.15868500	2.25386900
H	0.66117200	2.46465200	2.59718200
P	-2.36615600	-0.03223500	-0.22159100
C	-1.18950200	-0.26926500	-1.61222100
H	-1.70183900	-0.88564200	-2.35636500
H	-0.36779100	-0.84585700	-1.18941700
C	-0.63984300	1.00750400	-2.25642600
H	-1.44915000	1.58953900	-2.70299500
H	-0.18915300	1.62745400	-1.48133900
C	0.39967400	0.68386200	-3.32954100
H	1.24570000	0.13933200	-2.90595600
H	-0.02712000	0.07328100	-4.12920800
C	-4.02693600	0.17230500	-0.94364200
C	-4.27742200	1.24132500	-1.81297300
C	-5.06352000	-0.71668300	-0.65148600
C	-5.53227300	1.40689700	-2.38230800
H	-3.49866900	1.95763000	-2.03633900
C	-6.32028100	-0.54561700	-1.22171200
H	-4.89529700	-1.54469100	0.02270200
C	-6.55652200	0.51265000	-2.08902200

III-α-Br

C	4.07402000	0.46892900	-0.35487600
C	3.66422300	1.36955900	0.81238800
O	2.88178300	2.44483100	0.34923000
H	4.56120800	1.77796600	1.28650300
H	3.13058800	0.77408700	1.55926100
Br	5.00281700	-1.14368800	0.40892900
C	4.95509300	1.15586700	-1.38127800
H	5.90988700	1.41436800	-0.91482900
H	4.45227800	2.10096500	-1.60862400
H	3.18497300	0.04146800	-0.81625100
H	1.94483700	2.13671500	0.26703000
O	0.36351600	1.48935700	0.18790500
C	5.18825700	0.37103700	-2.66849000
H	4.24150100	0.13400400	-3.16103400
H	5.78924600	0.95238900	-3.37043900
H	5.70983800	-0.56749700	-2.47608300
C	-0.57309400	1.91715200	0.94029700
C	-1.86669400	1.29240200	0.89639500
C	-0.43694700	3.00248100	1.86069700
C	-2.92049000	1.70219800	1.73290700
C	-1.48595100	3.39077600	2.65906700
C	-2.73766300	2.74740900	2.61189100
H	-3.87638100	1.19581700	1.69302200
H	-1.34473000	4.21533600	3.34906900
H	-3.54454100	3.06840100	3.25641000
H	0.52325700	3.50060000	1.91282200
P	-1.94523600	-0.06064100	-0.23243800
C	-1.36924600	0.32603800	-1.93105800
H	-1.74130300	-0.47424500	-2.57730800
H	-0.28326900	0.26357200	-1.88127300
C	-1.78186900	1.69463900	-2.48162100
H	-2.86916100	1.75815000	-2.56619500
H	-1.46662200	2.46937000	-1.78203100
C	-1.15485400	1.95435800	-3.85134900
H	-0.06496600	1.94311300	-3.78962200
H	-1.45955900	1.20030000	-4.58136200
C	-3.67266100	-0.62186600	-0.37604700
C	-4.66297600	0.28722700	-0.76935100
C	-4.03120600	-1.94947600	-0.13304500
C	-5.97810400	-0.12863700	-0.92322200
H	-4.41270300	1.32560300	-0.93903700
C	-5.35046800	-2.36131600	-0.28673500
H	-3.28362400	-2.66479500	0.18017900
C	-6.32429100	-1.45447100	-0.68395300

H	-5.71058600	2.23855700	-3.05181200	H	-6.73352200	0.58528400	-1.22545200
H	-7.11386800	-1.24214600	-0.98342500	H	-5.61425100	-3.39290400	-0.09190600
H	-7.53537100	0.64439300	-2.53222800	H	-7.35096200	-1.77684500	-0.80222700
C	-2.38120300	-1.58692700	0.71526600	C	-0.95953900	-1.47136000	0.34325200
C	-2.28968000	-2.81923900	0.06234500	C	-0.48975800	-2.44347100	-0.54433800
C	-2.49547900	-1.55240100	2.10491900	C	-0.67078400	-1.58747900	1.70364800
C	-2.31494700	-3.99961700	0.79327000	C	0.26020700	-3.51366900	-0.07459000
H	-2.19497700	-2.86748700	-1.01473700	H	-0.69709400	-2.37039000	-1.60375300
C	-2.51918600	-2.73578900	2.83195800	C	0.07876200	-2.66026700	2.16856600
H	-2.54483800	-0.60062200	2.61654100	H	-1.01326100	-0.82695000	2.39271500
C	-2.43002800	-3.95907000	2.17866100	C	0.54608500	-3.62271200	1.28168100
H	-2.23803900	-4.94996400	0.28082800	H	0.63036300	-4.25587900	-0.76998400
H	-2.59466000	-2.69910800	3.91098000	H	0.30987300	-2.73480900	3.22324100
H	-2.44201900	-4.87994000	2.74772400	H	1.14188200	-4.45050600	1.64406400
H	0.78623100	1.59930400	-3.78040100	H	-1.45654800	2.93039700	-4.23494200
**III-β-I**				**III-β-Br**			
C	3.00125000	-0.02630700	1.22422300	C	3.54785600	0.32541400	0.89843500
C	3.85059900	-0.94200700	0.34406000	C	4.30968100	-0.81860400	0.23186300
O	1.90531500	-0.80013200	1.69143400	O	2.39016800	-0.23856400	1.49648000
H	4.34514300	-1.71717500	0.92346800	H	4.73034700	-1.50220400	0.96469200
H	3.24615200	-1.38537100	-0.44083100	H	3.66365200	-1.35425300	-0.45666500
I	5.44684800	0.08752200	-0.73559000	Br	5.83445900	-0.20226600	-0.87603700
C	3.74458500	0.52002800	2.43716800	C	4.34671400	1.03680600	1.98306300
H	4.66384000	1.00316000	2.09974700	H	5.30483500	1.35052600	1.56389400
H	4.03958400	-0.33068500	3.06027700	H	4.56375000	0.30846500	2.77143000
H	2.63868200	0.80985600	0.61151000	H	3.26233100	1.04846300	0.12277800
H	1.32111000	-1.01083400	0.92731300	H	1.79020900	-0.56221100	0.78542700
O	0.20619800	-1.09147000	-0.37488200	O	0.63464200	-0.90448200	-0.43058200
C	2.91665000	1.50388000	3.25991600	C	3.61913300	2.24168400	2.57434000
H	2.00168200	1.03616600	3.62426300	H	2.66376700	1.94675300	3.00930600
H	3.48101100	1.86251300	4.12312000	H	4.21685900	2.71409400	3.35654700
H	2.63551000	2.37564700	2.66290500	H	3.42150000	2.99577000	1.80783400
C	-0.68409500	-1.84233500	-0.89438200	C	-0.21377100	-1.79634600	-0.76351100
C	-2.06779300	-1.46726700	-0.80965300	C	-1.61655500	-1.54146600	-0.59234600
C	-0.41377000	-3.06157900	-1.58660500	C	0.12120800	-3.06634800	-1.32348800
C	-3.09144400	-2.25237100	-1.36469900	C	-2.59735000	-2.48314200	-0.94351300
C	-1.43223500	-3.81234300	-2.12671200	C	-0.85654300	-3.97215100	-1.66447700
C	-2.78000500	-3.42215100	-2.02528000	C	-2.22395300	-3.69735800	-1.48025100
H	-4.12538400	-1.94495400	-1.27208500	H	-3.64579900	-2.26052300	-0.79086100
H	-1.18971800	-4.73343800	-2.64513600	H	-0.56508900	-4.92768300	-2.08639400
H	-3.56182500	-4.03256700	-2.45608900	H	-2.97210100	-4.42863700	-1.75400100
H	0.61914300	-3.37579900	-1.67031400	H	1.17000500	-3.29296300	-1.46950600
P	-2.32832700	0.02045100	0.10664600	P	-1.94804400	0.03243300	0.13666400
C	-1.64236800	-0.03520800	1.80488700	C	-1.14711600	0.27421100	1.76723200
H	-2.16475100	0.72800500	2.38887600	H	-1.70511200	1.05730700	2.28855500
H	-0.60259600	0.27460100	1.70526400	H	-0.15302700	0.66270200	1.54677500
C	-1.69973600	-1.40060100	2.50223900	C	-1.02006600	-0.98388700	2.63649800
H	-2.73203200	-1.74874200	2.59218100	H	-2.00411800	-1.40842300	2.85188300
H	-1.18153900	-2.13255500	1.88281400	H	-0.47355100	-1.74329700	2.07745100
C	-1.04483000	-1.34565500	3.88195400	C	-0.28430400	-0.68379900	3.94202700
H	0.00659600	-1.06781100	3.79371800	H	0.72707900	-0.33065700	3.73619800
H	-1.53973400	-0.62040000	4.53345500	H	-0.80387000	0.07746300	4.53011900
C	-4.11877500	0.33324100	0.21918700	C	-3.74862800	0.20593200	0.34803600
C	-4.84176900	0.07798000	1.38606600	C	-4.36724900	0.03393700	1.58798100
C	-4.79370500	0.82628400	-0.90260500	C	-4.53887800	0.49288900	-0.76993700
C	-6.21190100	0.31041100	1.43049900	C	-5.74787400	0.14585500	1.70750900
H	-4.34521900	-0.30306600	2.26714200	H	-3.78103400	-0.18938300	2.46803200
C	-6.16216800	1.05323400	-0.85696400	C	-5.91733300	0.60009200	-0.64862800
H	-4.24794100	1.04067900	-1.81240100	H	-4.07598000	0.63887200	-1.73738100
C	-6.87382400	0.79636600	0.31019000	C	-6.52450400	0.42718100	0.59072600
H	-6.75944100	0.11032700	2.34252800	H	-6.21394100	0.01162100	2.67519400
H	-6.67160900	1.43581300	-1.73201600	H	-6.51667200	0.82271900	-1.52204200
H	-7.94048700	0.97762600	0.34619900	H	-7.59945700	0.51371600	0.68514600
C	-1.64410600	1.49616500	-0.70273000	C	-1.46046100	1.42547800	-0.92241500
C	-1.77383300	2.74139100	-0.08139400	C	-1.57729200	2.73218800	-0.44116600
C	-0.99952100	1.40435300	-1.93529400	C	-0.99027000	1.20761200	-2.21648000
C	-1.25906800	3.88008100	-0.68585000	C	-1.22444800	3.80677900	-1.24619300

H -2.28033100 2.83080500 0.87175600	H -1.94511000 2.92012000 0.55994400
C -0.49001300 2.54789400 -2.53859800	C -0.64392200 2.28665100 -3.02057000
H -0.88297700 0.44146200 -2.41171200	H -0.87983000 0.19789700 -2.58646000
C -0.61653300 3.78382000 -1.91575600	C -0.75846800 3.58475600 -2.53767700
H -1.36023200 4.84074100 -0.19735900	H -1.31350400 4.81592900 -0.86483400
H 0.01653400 2.46850400 -3.49176300	H -0.27293300 2.10950300 -4.02181500
H -0.21288700 4.67152900 -2.38602000	H -0.48177900 4.42296900 -3.16453400
H -1.09598500 -2.32047000 4.37012200	H -0.20892700 -1.58367100 4.55508000
**IV-α-I**	**IV-α-Br**
C -3.18382800 -0.91034000 -0.71816900	C 3.62716200 -0.73811800 0.63606400
C -4.67542100 -0.73687300 -0.48838200	C 5.04241800 -0.27515400 0.32828300
O -2.45592200 -0.78167900 0.47292600	O 2.83261300 -0.80664000 -0.51749700
H -4.97945600 -1.32413500 0.37648000	H 5.40855400 -0.80002700 -0.55259700
H -2.83565700 -0.20318800 -1.47990200	H 3.17852000 -0.08615600 1.39312500
H -1.55468300 -0.41904900 0.26923200	H 1.94812100 -0.39593200 -0.33727400
O -0.01034700 0.07752100 -0.10684600	O 0.40964500 0.15164600 0.02355700
C 0.43371700 1.27483700 -0.05657400	C -0.06726200 1.31999100 -0.17681000
C 1.85016500 1.52020600 -0.02703300	C -1.49039400 1.52380700 -0.21145200
C -0.39804700 2.43519100 -0.04001800	C 0.73162400 2.48917100 -0.36128600
C 2.37629800 2.82459600 -0.01302900	C -2.05275200 2.80121600 -0.38759500
C 0.14211500 3.69941400 -0.02892100	C 0.15650900 3.72603600 -0.53136600
C 1.53225000 3.91368700 -0.02150300	C -1.23915900 3.90226800 -0.54188100
H 3.44770300 2.97679900 0.00969100	H -3.12823700 2.92302100 -0.40681000
H -0.52350000 4.55537800 -0.02297000	H 0.79816300 4.59027500 -0.66245300
H 1.93396500 4.91774200 -0.01440800	H -1.66860700 4.88588600 -0.67487000
C -2.42022100 -2.69891100 2.47015100	C 2.78057000 -3.09106500 -2.03247900
O -1.36308600 -2.37680100 2.82343000	O 1.72564600 -2.86243800 -2.45806600
O -3.47427400 -3.07682900 2.16779500	O 3.83345600 -3.38841900 -1.64706500
H -1.47089900 2.28572000 -0.03571400	H 1.80766700 2.36820500 -0.36004200
P 2.86439800 0.07448300 0.08953700	P -2.46243900 0.05076200 -0.10282100
C 2.36009800 -0.92186700 1.53749700	C -1.96717800 -1.11941600 -1.41842400
H 2.47445900 -0.25946300 2.40021900	H -2.12509900 -0.58060800 -2.35705300
H 1.28702200 -1.05753400 1.39372500	H -0.88720600 -1.20786100 -1.29099000
C 3.06455500 -2.25998700 1.77309000	C -2.63886100 -2.49402500 -1.45635700
H 4.14853200 -2.12178700 1.82105300	H -3.72751200 -2.38992500 -1.47526200
H 2.87046100 -2.92542400 0.92964500	H -2.39091000 -3.04353800 -0.54631300
C 2.57782200 -2.92360200 3.06258600	C -2.18210400 -3.30088600 -2.67287600
H 2.80038600 -2.30462300 3.93500400	H -2.45722000 -2.80181900 -3.60508900
H 1.49879900 -3.08458400 3.03796200	H -1.09871700 -3.43084600 -2.67313100
C 2.86004700 -0.95974700 -1.40710200	C -2.38980400 -0.76117800 1.52253600
C 3.83453600 -1.94919300 -1.57913700	C -3.28941300 -1.78494500 1.84057200
C 1.88727000 -0.77727300 -2.39164600	C -1.44478200 -0.36029000 2.46849800
C 3.83100400 -2.74684800 -2.71548000	C -3.23818500 -2.40152000 3.08347600
H 4.60747400 -2.09322600 -0.83592800	H -4.04203900 -2.09542200 1.12809500
C 1.89179500 -1.57626200 -3.52868700	C -1.40179500 -0.97822100 3.71250300
H 1.11816200 -0.03251300 -2.25584900	H -0.73666200 0.41697900 2.22517700
C 2.85821400 -2.56131500 -3.69178200	C -2.29318600 -1.99851100 4.02087700
H 4.58976700 -3.50903700 -2.83878300	H -3.93857200 -3.19231000 3.31992200
H 1.13219700 -1.42904200 -4.28556700	H -0.66412000 -0.66250000 4.43888700
H 2.85581900 -3.18268900 -4.57849900	H -2.25408400 -2.47857800 4.99060600
C 4.59107300 0.59844700 0.32596100	C -4.20929400 0.49248700 -0.36094300
C 5.38121400 0.92246300 -0.78110700	C -4.99576500 0.90450700 0.71888600
C 5.12677600 0.74947200 1.60748400	C -4.76355300 0.49931000 -1.64382200
C 6.68118900 1.37951500 -0.60842800	C -6.30977600 1.30774900 0.51943500
H 4.98258200 0.81625300 -1.78116400	H -4.58290100 0.91072000 1.71869900
C 6.42941200 1.20237800 1.77722400	C -6.07954400 0.89904000 -1.83986100
H 4.53363400 0.51958900 2.48184800	H -4.17373200 0.19881500 -2.49892500
C 7.20877400 1.51663600 0.67051600	C -6.85472500 1.30300600 -0.75935800
H 7.28125700 1.62747400 -1.47447200	H -6.90663000 1.62559500 1.36461200
H 6.83270200 1.31041700 2.77582700	H -6.49677800 0.89667800 -2.83859100
H 8.22357600 1.86908300 0.80415700	H -7.87979100 1.61491700 -0.91388900
H 3.06275400 -3.89061400 3.20788100	H -2.64309300 -4.29025900 -2.67645600
H -3.06003200 -1.92361100 -1.13776000	H 3.72708700 -1.73965400 1.08427500
C -5.51333700 -1.07590600 -1.71295600	C 5.99543400 -0.43178800 1.50342800
H -5.16287200 -2.05479000 -2.06431500	H 5.89144200 -1.46707200 1.84974900
H -5.29361300 -0.36646500 -2.51540900	H 5.66032500 0.20468300 2.32709400
C -7.01909200 -1.15257200 -1.47754000	C 7.46427200 -0.15970900 1.19159400

H	-7.53313400	-1.46748100	-2.38771400	H	8.08438000	-0.35153000	2.06931900
H	-7.25730600	-1.87459200	-0.69284400	H	7.81976200	-0.80502900	0.38463000
H	-7.42526900	-0.18616400	-1.17823400	H	7.61999800	0.87510800	0.88676000
I	-5.08249100	1.32565500	0.19458900	Br	5.00116800	1.63288600	-0.28246100
**IV-β-I**				**IV-β-Br**			
C	-3.30105300	-0.49044500	-0.19167600	C	-3.72710400	0.05912200	-0.07613200
C	-4.61721200	-0.42857500	0.59246700	C	-5.10970700	-0.53996400	0.18676800
O	-2.33524700	-0.97244900	0.73420900	O	-2.84361500	-1.05328100	-0.02117800
H	-5.18357700	-1.35474200	0.54528700	H	-5.56833500	-0.94370900	-0.71170700
H	-4.40943500	-0.17666800	1.62579600	H	-5.02714400	-1.31626600	0.93895700
I	-6.01481600	1.12592900	-0.03958800	Br	-6.42651200	0.73801600	0.93130200
C	-3.33208000	-1.41323300	-1.41719900	C	-3.57113700	0.77642800	-1.42412000
H	-2.30007600	-1.48433400	-1.77073400	H	-2.49952200	0.95252800	-1.55012400
H	-3.60801700	-2.41470000	-1.07161500	H	-3.86229000	0.07431000	-2.21244800
H	-3.02555200	0.52155200	-0.50918500	H	-3.49221400	0.75986200	0.73489900
H	-1.46857100	-0.52098700	0.55975400	H	-1.92779100	-0.72479800	0.17758400
O	0.01434000	0.18356500	0.22701200	O	-0.37207800	-0.16486300	0.47368700
C	0.52467800	1.18661000	0.83135500	C	0.24617600	-0.30268500	1.58302200
C	1.95010500	1.37801000	0.84428500	C	1.68169000	-0.22543500	1.63144000
C	-0.23884300	2.18454000	1.50996000	C	-0.40367700	-0.52321900	2.83568500
C	2.54321100	2.49340500	1.46174200	C	2.38873300	-0.33297900	2.84252600
C	0.36539600	3.26575300	2.10677500	C	0.31099000	-0.62411200	4.00558100
C	1.76058800	3.43973400	2.08744900	C	1.71356800	-0.52555000	4.02823200
H	3.61937200	2.61038500	1.45165300	H	3.46937400	-0.27076700	2.84690000
H	-0.25147800	4.00468700	2.60595300	H	-0.22163300	-0.78546900	4.93619900
H	2.21428000	4.29980600	2.56059800	H	2.25596200	-0.60604100	4.96031200
C	-2.12231600	-3.46402400	1.97133100	C	-2.69583200	-3.28845000	-1.72542400
O	-0.97446900	-3.33863100	2.08775500	O	-1.53986600	-3.32034400	-1.62756800
O	-3.26288600	-3.65285100	1.88490800	O	-3.84627900	-3.31024600	-1.86693600
H	-1.31446800	2.06293100	1.53519300	H	-1.48321200	-0.60718900	2.83153100
C	-4.23528300	-0.99082600	-2.57545000	C	-4.31887000	2.09887600	-1.59519700
H	-4.10859600	-1.67148500	-3.42039300	H	-4.05024700	2.56294500	-2.54697600
H	-5.29084900	-0.99791200	-2.30212800	H	-5.40056800	1.96857100	-1.58222700
H	-3.99530600	0.01699000	-2.92247000	H	-4.06629600	2.80390000	-0.79955300
P	2.89056800	0.07610100	0.10151100	P	2.48524800	-0.07413700	0.06224400
C	2.46752100	-1.52020300	0.88681900	C	1.96720000	-1.44356200	-1.03325700
H	2.69669100	-1.38649300	1.94771100	H	2.26354300	-2.35825800	-0.51213100
H	1.38040400	-1.56842300	0.81116100	H	0.87735300	-1.40764700	-0.99518700
C	3.12252200	-2.79200700	0.34192600	C	2.47573400	-1.44516900	-2.47696600
H	4.21129200	-2.68787000	0.32425800	H	3.56711700	-1.37535600	-2.50119800
H	2.80455300	-2.94965900	-0.69038800	H	2.09328200	-0.56476800	-2.99693200
C	2.74235300	-4.01444600	1.17894200	C	2.03047300	-2.70465500	-3.22180700
H	3.09178600	-3.91312400	2.20911100	H	2.43807400	-3.60513500	-2.75649100
H	1.66002000	-4.14876700	1.20897200	H	0.94324100	-2.79688400	-3.22259400
C	2.68825100	-0.02957600	-1.70165200	C	2.20646900	1.52605300	-0.75462800
C	3.53126900	-0.84898800	-2.46075500	C	2.97670000	1.89415900	-1.86336700
C	1.69508900	0.71309600	-2.34246100	C	1.22834300	2.40053700	-0.27836100
C	3.37751600	-0.92713200	-3.83830600	C	2.76467800	3.11505400	-2.48921500
H	4.31862000	-1.41833000	-1.98494600	H	3.75281000	1.23819400	-2.23457200
C	1.54914300	0.63400700	-3.72213300	C	1.02378300	3.62318400	-0.90654700
H	1.02857100	1.33310000	-1.76254600	H	0.61855600	2.11706500	0.56576200
C	2.38528300	-0.18553400	-4.47047600	C	1.78669700	3.98084500	-2.01144700
H	4.03491400	-1.56368900	-4.41658100	H	3.36577900	3.39062600	-3.34627300
H	0.77435200	1.21144900	-4.20962700	H	0.26090300	4.29330800	-0.53196200
H	2.26611600	-0.24585200	-5.54492700	H	1.62199700	4.93333000	-2.49923600
C	4.65735300	0.39947500	0.39302000	C	4.28272500	-0.19640000	0.31855900
C	5.38900500	1.17731100	-0.50891200	C	5.02122900	0.94384200	0.64894100
C	5.28359900	-0.05908300	1.55565800	C	4.93219600	-1.43241800	0.26455300
C	6.72065700	1.48170500	-0.25678700	C	6.38132400	0.84889500	0.91310800
H	4.91986600	1.54590900	-1.41104500	H	4.53462400	1.90869100	0.69984600
C	6.61650000	0.24376300	1.80314500	C	6.29396300	-1.52364900	0.52463000
H	4.73706300	-0.64881700	2.27894200	H	4.38235600	-2.33213300	0.02485500
C	7.33732800	1.01312500	0.89734600	C	7.02040500	-0.38400600	0.84852900
H	7.27492200	2.08573500	-0.96354100	H	6.94068100	1.73939500	1.16941000
H	7.08967000	-0.12024700	2.70597400	H	6.78509300	-2.48693500	0.47615800
H	8.37589000	1.24873200	1.09175700	H	8.08118200	-0.45673200	1.05191000
H	3.18687700	-4.92080400	0.76393200	H	2.37074100	-2.68173700	-4.25860800

**TS2-α-I**				**TS2-α-Br**			
C	3.25825100	1.29837900	-0.38786300	C	-3.65583000	-1.08281900	-0.34883000
C	4.66014800	0.88042400	0.03139500	C	-4.99862700	-0.48494800	0.04291300
O	2.25029500	0.84637500	0.48164500	O	-2.60538300	-0.70655900	0.50591400
H	4.78221000	1.05102900	1.09789100	H	-5.12770100	-0.56332300	1.11956500
H	3.04118800	0.94467600	-1.40137300	H	-3.40264000	-0.80052700	-1.37626300
H	1.10516000	0.42776200	-0.04838900	H	-1.45936300	-0.32869900	-0.02568200
O	0.09843500	0.17471600	-0.42279200	O	-0.44504900	-0.09221000	-0.40485800
C	-0.25322400	-1.09431500	-0.41429500	C	-0.06007600	1.16472200	-0.33643100
C	-1.63373800	-1.39871000	-0.30643700	C	1.32841600	1.42842500	-0.22297400
C	0.65555100	-2.16066000	-0.53116200	C	-0.94233200	2.25847700	-0.39322500
C	-2.07779500	-2.72732300	-0.37628100	C	1.80707900	2.74681900	-0.22810500
C	0.19631200	-3.46241700	-0.58668700	C	-0.44899200	3.54886500	-0.38596700
C	-1.16914700	-3.75776000	-0.52626300	C	0.92429800	3.80608900	-0.31938400
H	-3.13343200	-2.95007700	-0.29889400	H	2.86873900	2.93739700	-0.14687300
H	0.91351500	-4.26875400	-0.68152000	H	-1.14524400	4.37737600	-0.43537100
H	-1.51282700	-4.78176300	-0.58064400	H	1.29352100	4.82262000	-0.32439800
C	1.93035300	1.83410600	1.82106300	C	-2.34736700	-1.70330100	1.85634600
O	0.91624900	1.41656000	2.32313300	O	-1.29184300	-1.36878800	2.33359800
O	2.77786400	2.67843700	1.92075500	O	-3.26545900	-2.46578700	1.98126300
H	1.71447800	-1.94100600	-0.57340300	H	-2.00662300	2.06634400	-0.43996200
P	-2.73035300	-0.04545400	0.09592500	P	2.38849000	0.02624200	0.09849000
C	-2.15644200	0.71016000	1.65039700	C	1.80471400	-0.78979900	1.61834400
H	-2.22396900	-0.09187600	2.39164100	H	1.90844000	-0.02975900	2.39855000
H	-1.08837000	0.89910100	1.51775000	H	0.72930900	-0.93262300	1.48906200
C	-2.86383600	1.97430900	2.14947600	C	2.47081800	-2.10140400	2.04766200
H	-3.94600400	1.82130300	2.20801500	H	3.55870200	-1.99146100	2.09652000
H	-2.69491900	2.78511400	1.43825000	H	2.26143300	-2.87202400	1.30332000
C	-2.32416600	2.38697300	3.52051300	C	1.93504700	-2.55582400	3.40689200
H	-2.54014800	1.62531000	4.27373100	H	2.19088000	-1.83798400	4.19001100
H	-1.24266000	2.52230000	3.48418700	H	0.84867400	-2.64795700	3.38226200
C	-2.88646500	1.16715200	-1.24377600	C	2.49334200	-1.11473300	-1.30722900
C	-3.82861400	2.19676700	-1.13565000	C	3.37982600	-2.19691200	-1.26026200
C	-2.09607800	1.07807400	-2.39054200	C	1.71754700	-0.91259200	-2.44957900
C	-3.96679200	3.12757300	-2.15532500	C	3.47703500	-3.06770800	-2.33631800
H	-4.46414800	2.27102600	-0.26363800	H	4.00445500	-2.35850400	-0.39224600
C	-2.24352300	2.01023800	-3.41059400	C	1.82398000	-1.78473400	-3.52596600
H	-1.35909300	0.29480100	-2.48169000	H	1.02451400	-0.08628100	-2.49517900
C	-3.17336900	3.03563500	-3.29403400	C	2.69817800	-2.86286700	-3.47043400
H	-4.69481300	3.92282300	-2.06055500	H	4.16229900	-3.90412100	-2.28896000
H	-1.62340800	1.93538800	-4.29437000	H	1.21539400	-1.62196000	-4.40593600
H	-3.28144900	3.76248200	-4.08903700	H	2.77441700	-3.54297100	-4.30927300
C	-4.39813100	-0.72342300	0.35016300	C	4.07812900	0.64059100	0.37200500
C	-5.25910700	-0.88795900	-0.73929900	C	4.94840700	0.80425900	-0.70995500
C	-4.81765800	-1.13965200	1.61675400	C	4.50667700	1.00884500	1.65054600
C	-6.51468100	-1.45424800	-0.56322300	C	6.22249000	1.32115500	-0.51465100
H	-4.95181800	-0.57109500	-1.72688600	H	4.63414500	0.52480900	-1.70654000
C	-6.07654400	-1.70143500	1.78970700	C	5.78343700	1.52157900	1.84250800
H	-4.16877400	-1.03180000	2.47473700	H	3.85041000	0.90215700	2.50306900
C	-6.92625700	-1.85937800	0.70143900	C	6.64280800	1.67803400	0.76147800
H	-7.17143500	-1.57621400	-1.41480300	H	6.88660100	1.44262400	-1.36059200
H	-6.39106200	-2.01538700	2.77652100	H	6.10442700	1.79866000	2.83824100
H	-7.90703300	-2.29651900	0.83856300	H	7.63769400	2.07677200	0.91324700
H	-2.78345900	3.32099400	3.84900000	H	2.36243400	-3.52066100	3.68556100
H	3.26198000	2.39491000	-0.40246400	H	-3.78162000	-2.17059500	-0.31190200
C	5.75101600	1.57235400	-0.77165000	C	-6.16895900	-1.10441400	-0.70411300
H	5.52435300	2.64452000	-0.72062600	H	-6.07859300	-2.18727700	-0.56074100
H	5.67046300	1.29054000	-1.82555500	H	-6.05468600	-0.92146600	-1.77650900
C	7.17654800	1.35151300	-0.27456800	C	-7.55114900	-0.65769200	-0.23625600
H	7.88087500	1.94767500	-0.85791000	H	-8.33068700	-1.19693500	-0.77774100
H	7.27559500	1.64739600	0.77229700	H	-7.68591900	-0.85725100	0.82929700
H	7.47161600	0.30506600	-0.35728600	H	-7.70178800	0.40941600	-0.40016000
I	4.85611800	-1.32216800	-0.11403600	Br	-4.96344200	1.50396200	-0.26037600
**TS2-β-I**				**TS2-β-Br**			
C	-3.33218200	-0.77450000	0.04377300	C	-3.76026100	-0.47016800	0.01270500
C	-4.51333000	-0.21631500	0.85334300	C	-4.95260200	0.18055800	0.72895700

O	-2.12893600	-0.59891900	0.79132900	O	-2.57339500	-0.24341700	0.77292100
H	-5.12825900	-0.98166600	1.31119500	H	-5.57105400	-0.52461400	1.27068400
H	-4.16284800	0.47268800	1.61140100	H	-4.61271800	0.96041900	1.39913200
I	-5.90751200	0.99588800	-0.32876600	Br	-6.18830700	1.11132000	-0.52626300
C	-3.50413600	-2.23299700	-0.39867300	C	-3.94268700	-1.95987200	-0.30034500
H	-2.56046800	-2.53490900	-0.86189100	H	-2.99231400	-2.31478200	-0.70912400
H	-3.62995100	-2.84899300	0.49281600	H	-4.10003400	-2.49042300	0.64004800
H	-3.20046200	-0.15776100	-0.85170000	H	-3.60258000	0.06548800	-0.92963200
H	-1.01907500	-0.31312500	0.14580500	H	-1.44126200	-0.08337900	0.12940400
O	-0.03632600	-0.10624800	-0.32774500	O	-0.44054300	0.02009400	-0.34561600
C	0.32783600	1.15587600	-0.40733300	C	0.00650700	1.24490000	-0.52265500
C	1.71286400	1.45473900	-0.35841100	C	1.40790100	1.45594600	-0.48034000
C	-0.57500300	2.22304800	-0.56550300	C	-0.82225500	2.35256500	-0.77850500
C	2.16283900	2.77301800	-0.51801000	C	1.94577600	2.72416700	-0.74022600
C	-0.11023800	3.51618000	-0.71037000	C	-0.27145100	3.59616100	-1.02223300
C	1.25794000	3.80186200	-0.70193100	C	1.11254000	3.79143900	-1.01933700
H	3.22158100	2.99145300	-0.48465400	H	3.01635900	2.87522100	-0.70981500
H	-0.82384400	4.32123500	-0.83715400	H	-0.92894700	4.43329200	-1.22314600
H	1.60772300	4.81767500	-0.82535200	H	1.52972000	4.76865500	-1.22050900
C	-1.89638300	-1.37587400	2.25194300	C	-2.40369600	-0.87272000	2.30886300
O	-0.72330100	-1.26595700	2.51883500	O	-1.23368900	-0.77950700	2.59558100
O	-2.93140700	-1.82326300	2.66721700	O	-3.46494800	-1.23683200	2.73993800
H	-1.63572500	2.01040800	-0.57727000	H	-1.89452600	2.20897900	-0.78554500
C	-4.65098100	-2.47846900	-1.37832700	C	-5.06860700	-2.27951000	-1.28324300
H	-4.66992700	-3.52725000	-1.68184900	H	-5.09032900	-3.35026800	-1.49644300
H	-5.62260800	-2.24688700	-0.93974800	H	-6.04756900	-2.00236400	-0.89023800
H	-4.54858300	-1.87231100	-2.28173700	H	-4.93821900	-1.75269100	-2.23169200
P	2.81631200	0.11507300	0.07817600	P	2.41445800	0.08608100	0.08024100
C	2.29102900	-0.56755000	1.68243800	C	1.83287100	-0.41297300	1.73041800
H	2.37626200	0.26665100	2.38538100	H	1.97496500	0.47285600	2.35700600
H	1.22088700	-0.76886800	1.59099200	H	0.75129600	-0.54227400	1.64422100
C	3.02016400	-1.80679800	2.21212600	C	2.46583100	-1.64879500	2.37950700
H	4.10291200	-1.64828200	2.23014500	H	3.55735000	-1.57025300	2.39272500
H	2.83263600	-2.64625000	1.53987200	H	2.21769900	-2.53157500	1.78709800
C	2.52532000	-2.16441700	3.61521000	C	1.94316000	-1.83588200	3.80531100
H	2.76322900	-1.37275100	4.32992000	H	2.23644900	-0.99942500	4.44441600
H	1.44364400	-2.30222100	3.61909500	H	0.85427500	-1.89440700	3.81295200
C	2.92966200	-1.16246300	-1.20431200	C	2.44443300	-1.29768100	-1.09232200
C	3.89284100	-2.17120000	-1.08363800	C	3.32217100	-2.36587700	-0.87373200
C	2.08480500	-1.14980800	-2.31506200	C	1.62211600	-1.30409200	-2.21979500
C	3.99838500	-3.15656700	-2.05459900	C	3.36647200	-3.42726100	-1.76628600
H	4.56942700	-2.18701000	-0.23987600	H	3.98027200	-2.36985700	-0.01530400
C	2.19983700	-2.13648400	-3.28695900	C	1.67572000	-2.36741900	-3.11284600
H	1.33084500	-0.38407000	-2.41477500	H	0.93278500	-0.49174900	-2.39269900
C	3.15085800	-3.14066800	-3.15736100	C	2.54234800	-3.42927800	-2.88681800
H	4.74311100	-3.93495400	-1.95018900	H	4.04566600	-4.25068800	-1.58728600
H	1.53809700	-2.12048400	-4.14311600	H	1.03214200	-2.36559800	-3.98289700
H	3.23346100	-3.91001800	-3.91451100	H	2.57734900	-4.25791200	-3.58261900
C	4.49210100	0.80000600	0.24611100	C	4.13307800	0.66614600	0.20673200
C	5.30925000	0.93103200	-0.88117200	C	4.95965400	0.64927400	-0.92111300
C	4.96014000	1.25478600	1.48196900	C	4.62901400	1.18407600	1.40617300
C	6.56955100	1.50339600	-0.77182600	C	6.25603200	1.14115300	-0.84855200
H	4.96421200	0.58369200	-1.84586900	H	4.59290200	0.24814100	-1.85652600
C	6.22388900	1.82236000	1.58813700	C	5.92870900	1.66992900	1.47627100
H	4.34562200	1.17199700	2.36762700	H	4.00878400	1.21351500	2.29111000
C	7.02960700	1.94742500	0.46283300	C	6.74304100	1.64999200	0.35034300
H	7.19240400	1.59963800	-1.65169800	H	6.88581600	1.12371000	-1.72859300
H	6.57665700	2.16611100	2.55179600	H	6.30284200	2.06382300	2.41230300
H	8.01430000	2.38899800	0.54768500	H	7.75548800	2.02883100	0.40709900
H	2.99799000	-3.08349800	3.96615000	H	2.34696000	-2.74949400	4.24527500
**V(O_a)-α-I**				**V(O_a)-α-Br**			
C	0.27341800	-1.06898700	0.49903600	C	0.04936000	1.11872900	0.44145000
C	1.64207500	-1.37981400	0.33240800	C	-1.32229200	1.39995300	0.25167200
C	-0.64952000	-2.11665800	0.61452700	C	0.95817700	2.18312700	0.49950700
C	2.06773700	-2.71518500	0.35147400	C	-1.76606600	2.72816100	0.18968100
C	-0.20883200	-3.42757000	0.61685200	C	0.49957800	3.48575900	0.42290700
C	1.14854900	-3.73713200	0.50488400	C	-0.86125400	3.76962100	0.28647000

H	3.11606300	-2.95060900	0.22830800	H	-2.81672200	2.94144400	0.04781500
H	-0.93450200	-4.22607100	0.71080400	H	1.21394300	4.29828900	0.47299100
H	1.47971800	-4.76662100	0.51941400	H	-1.20605200	4.79363900	0.23878000
H	-1.70290600	-1.88120600	0.69657800	H	2.01388500	1.96572500	0.60042100
P	2.74230200	-0.03310200	-0.10181700	P	-2.40122100	0.01497500	-0.10816900
C	2.10922400	0.73276900	-1.62450900	C	-1.75001300	-0.82371000	-1.58341000
H	2.16044500	-0.06145500	-2.37569900	H	-1.82106100	-0.07681000	-2.38008800
H	1.04069500	0.90991400	-1.46319500	H	-0.67679000	-0.96107100	-1.41424800
C	2.78501600	2.00960300	-2.13503700	C	-2.39007000	-2.14613200	-2.01884900
H	3.86651300	1.87174200	-2.23419100	H	-3.47502500	-2.04479500	-2.12386200
H	2.63049400	2.81146400	-1.41044300	H	-2.21307200	-2.90067200	-1.24986700
C	2.18797100	2.42668800	-3.48102500	C	-1.78161800	-2.62117600	-3.34018300
H	2.39328300	1.67823300	-4.25042200	H	-2.01344100	-1.92626000	-4.15104500
H	1.10558000	2.53590400	-3.40296000	H	-0.69590000	-2.68806400	-3.26078300
C	2.96317300	1.15879800	1.24698200	C	-2.60717000	-1.09897600	1.30744300
C	3.89679900	2.19185900	1.10432600	C	-3.51499500	-2.16103700	1.22211200
C	2.23515500	1.05065400	2.43262700	C	-1.89523700	-0.89836000	2.49079200
C	4.08626400	3.10832700	2.12859800	C	-3.69525300	-3.01421500	2.30135200
H	4.48480500	2.27997600	0.20095700	H	-4.09085000	-2.32099100	0.32069300
C	2.43392900	1.96842000	3.45701100	C	-2.08493800	-1.75281400	3.57014400
H	1.50654800	0.26336200	2.55289500	H	-1.18725200	-0.08718500	2.56787200
C	3.35384800	2.99817400	3.30600400	C	-2.97933800	-2.81143000	3.47664300
H	4.80629200	3.90716500	2.00697100	H	-4.39557100	-3.83577100	2.22414700
H	1.86160700	1.87907100	4.37113200	H	-1.52561600	-1.59137400	4.48243500
H	3.50183100	3.71439700	4.10418900	H	-3.12027300	-3.47805800	4.31789100
C	4.39064200	-0.73026100	-0.42148500	C	-4.05927600	0.66819300	-0.46928600
C	5.28363400	-0.92462000	0.63708400	C	-4.95848600	0.90741900	0.57475500
C	4.76583500	-1.12435700	-1.70879300	C	-4.43620300	0.98433000	-1.77742700
C	6.52657400	-1.50046100	0.41062500	C	-6.20917700	1.45084900	0.31345500
H	5.01144300	-0.62214100	1.63944800	H	-4.68510900	0.66515300	1.59306200
C	6.01259500	-1.69544600	-1.93203700	C	-5.69071000	1.52289900	-2.03524600
H	4.09255700	-0.99078100	-2.54390100	H	-3.75821000	0.81536000	-2.60223800
C	6.89377500	-1.88460900	-0.87423600	C	-6.57792300	1.75742500	-0.99167400
H	7.20858000	-1.64542500	1.23840600	H	-6.89603400	1.63134100	1.13017600
H	6.29311300	-1.99184400	-2.93439200	H	-5.97245700	1.75855300	-3.05325500
H	7.86493800	-2.32897700	-1.05078000	H	-7.55503900	2.17662600	-1.19491700
H	2.61596500	3.37397500	-3.81394000	H	-2.17772900	-3.60024900	-3.61556200
C	-3.36519800	1.39347500	0.38789600	C	3.79104800	-1.20494000	0.36122100
C	-4.71618200	0.88935700	-0.09675400	C	5.04771300	-0.48838400	-0.10815300
H	-3.40791900	2.48606200	0.33398000	H	3.97322400	-2.27532400	0.23151200
I	-4.83512000	-1.30786800	0.17990600	Br	4.94249200	1.45728100	0.40616900
O	-2.82707500	2.53862800	-1.96439400	O	3.32576200	-2.28636300	-2.03219100
C	-1.96878200	1.72602900	-1.68838800	C	2.38838900	-1.59183300	-1.69424200
O	-0.90494200	1.33786500	-2.15006900	O	1.27689700	-1.30479600	-2.11941000
O	-2.27116300	0.93574400	-0.37748300	O	2.62952200	-0.84529500	-0.35579400
O	-0.05719600	0.21516400	0.56704600	O	0.39860800	-0.15528300	0.58469300
H	-1.00441000	0.45188600	0.22100200	H	1.34649200	-0.38754400	0.24711700
H	-3.19565800	1.10129500	1.42807400	H	3.61080700	-1.00264500	1.42036500
C	-5.88420800	1.58048300	0.58963500	C	6.32336500	-1.10323200	0.44414300
H	-5.86332900	1.36707000	1.66237200	H	6.31591600	-1.04264400	1.53644000
H	-5.69601000	2.65603400	0.48436300	H	6.27493400	-2.16800800	0.18936400
C	-7.26353300	1.26961600	0.01599600	C	7.62119300	-0.52136400	-0.10862500
H	-8.02738000	1.87094800	0.51270600	H	8.48134600	-1.06765100	0.28299300
H	-7.30211600	1.49612200	-1.05182300	H	7.64793100	-0.59329200	-1.19823400
H	-7.52497600	0.21908100	0.14646300	H	7.73588800	0.52827700	0.16230900
H	-4.77117800	0.99084500	-1.17709600	H	5.05979300	-0.45473200	-1.19393900
**V(O$_a$)-β-I**				**V(O$_a$)-β-Br**			
C	-0.37349100	-1.17943200	-0.55304000	C	0.00126100	-1.19991800	-0.67497300
C	-1.75694600	-1.45722600	-0.46786800	C	-1.38890000	-1.43503700	-0.57277200
C	0.51346000	-2.24000300	-0.78510500	C	0.84308700	-2.27196000	-1.00279700
C	-2.22588500	-2.76233100	-0.66699700	C	-1.91058400	-2.70515100	-0.85026600
C	0.03042000	-3.52338200	-0.96645200	C	0.30868800	-3.52163500	-1.26037400
C	-1.33869600	-3.79249600	-0.92383400	C	-1.06805600	-3.74502700	-1.20126400
H	-3.28491400	-2.97201600	-0.60485000	H	-2.97501700	-2.88112500	-0.77537900
H	0.72988000	-4.32957200	-1.15025100	H	0.97347100	-4.33691700	-1.51797400
H	-1.70513200	-4.79839300	-1.07670500	H	-1.47449400	-4.72415000	-1.41483700
H	1.57444100	-2.03351700	-0.82779000	H	1.90972400	-2.10050800	-1.05840700

P	-2.83172300	-0.12860400	0.08228500	P	-2.39884600	-0.10894700	0.09252700
C	-2.23889500	0.43525900	1.70424800	C	-1.75237100	0.31306800	1.73734600
H	-2.33390700	-0.43853500	2.35642700	H	-1.86718500	-0.60407400	2.32370200
H	-1.16213300	0.60562500	1.60070500	H	-0.67210900	0.45233000	1.62272500
C	-2.90595700	1.66353400	2.33353200	C	-2.35948500	1.51185800	2.47465500
H	-3.99329100	1.54498900	2.37684900	H	-3.44965100	1.43094100	2.53189900
H	-2.70504600	2.53729300	1.71057700	H	-2.13576800	2.42375800	1.91775100
C	-2.35339100	1.90988500	3.73922600	C	-1.77042200	1.62443700	3.88253800
H	-2.60637900	1.08543100	4.41049900	H	-2.04284300	0.75941100	4.49235800
H	-1.26663500	1.99775100	3.71468000	H	-0.68176500	1.67258700	3.83962100
C	-2.96173000	1.21679100	-1.12668800	C	-2.49175200	1.31767800	-1.02239100
C	-3.89320300	2.23987100	-0.91347300	C	-3.33448800	2.38822500	-0.70110700
C	-2.16609900	1.24040900	-2.27279500	C	-1.75701500	1.35395800	-2.20790600
C	-4.01418700	3.27545000	-1.82872000	C	-3.42719100	3.48203500	-1.54969200
H	-4.53234400	2.22780300	-0.04096800	H	-3.92592100	2.36960700	0.20421400
C	-2.29654800	2.27753000	-3.18839100	C	-1.85861400	2.44988900	-3.05605600
H	-1.43733800	0.46331700	-2.44515500	H	-1.09822700	0.53825000	-2.46409700
C	-3.21480900	3.29584400	-2.96688400	C	-2.68794900	3.51463300	-2.72752400
H	-4.73318500	4.06507500	-1.65304200	H	-4.07749600	4.30754900	-1.29091900
H	-1.67200600	2.28999500	-4.07203400	H	-1.28075500	2.47163400	-3.97074000
H	-3.30929100	4.10507400	-3.67973100	H	-2.75998200	4.36950300	-3.38775500
C	-4.51089200	-0.80294100	0.25362600	C	-4.10127200	-0.72624900	0.25282400
C	-5.34826900	-0.88117300	-0.86392200	C	-4.97199700	-0.66325900	-0.83963400
C	-4.96439500	-1.29546600	1.48025200	C	-4.54235700	-1.31191400	1.44264100
C	-6.61365800	-1.44162400	-0.75411900	C	-6.25793200	-1.17763000	-0.74213400
H	-5.01504000	-0.50148600	-1.82068400	H	-4.64854000	-0.20752700	-1.76594200
C	-6.23377300	-1.85044700	1.58701700	C	-5.83176800	-1.82040100	1.53775000
H	-4.33467400	-1.25042100	2.35780600	H	-3.88759800	-1.37580100	2.30047300
C	-7.05895700	-1.92475600	0.47148700	C	-6.69015300	-1.75490000	0.44678400
H	-7.25250500	-1.49747200	-1.62596900	H	-6.92274900	-1.12359700	-1.59446700
H	-6.57564000	-2.22361600	2.54361300	H	-6.16355300	-2.26683600	2.46609800
H	-8.04787500	-2.35666800	0.55681200	H	-7.69466600	-2.15099400	0.52321400
H	-2.77330100	2.82469400	4.16134800	H	-2.14528300	2.51805700	4.38471300
C	4.48506900	-0.20945900	0.83639400	C	4.93668300	-0.46627700	0.67783900
C	3.45750200	0.64096000	0.08978400	C	3.90936100	0.44105700	0.00087400
H	4.91518400	0.29807300	1.68995700	H	5.41597500	-0.00362600	1.53038600
H	4.05550400	-1.15750800	1.14271600	H	4.48807100	-1.41098300	0.96688700
H	3.30489500	0.19971800	-0.90147900	H	3.73301900	0.04784400	-1.00642500
I	6.17746000	-0.77164000	-0.42943900	Br	6.40039000	-0.95382600	-0.57625000
O	2.17839200	0.50650400	0.72579600	O	2.63989600	0.30582100	0.65282400
C	1.94109000	0.99993200	2.17895000	C	2.45255400	0.70677300	2.13738800
O	0.74304500	0.93572700	2.42167500	O	1.25916200	0.66411500	2.40806900
O	2.95483600	1.32599800	2.76037000	O	3.49016800	0.96092100	2.71358000
C	3.87981600	2.09945000	-0.09154000	C	4.36603500	1.89487400	-0.12052200
H	4.86486700	2.10546200	-0.56582500	H	5.34990700	1.89429500	-0.59739800
H	3.99213300	2.54863800	0.89559100	H	4.49249900	2.29846700	0.88464900
C	2.89942900	2.90888000	-0.93701400	C	3.40414700	2.76371500	-0.92730100
H	1.91165800	2.94189700	-0.47512500	H	2.41675200	2.79637800	-0.46443000
H	3.24727100	3.93712200	-1.05392800	H	3.77362800	3.78875500	-0.99639100
H	2.78822900	2.48122500	-1.93775200	H	3.28472500	2.38550800	-1.94681700
O	0.00684800	0.08605200	-0.44091200	O	0.42855600	0.04117700	-0.48660800
H	0.94004000	0.23338400	-0.00180300	H	1.37554200	0.12604600	-0.06235400
**V(O_b)-α-I**				**V(O_b)-α-Br**			
C	-1.78885700	2.24304900	-0.61971100	C	-1.26183600	2.25213400	-0.43001600
C	-2.84912500	1.37145200	-1.03041500	C	-2.37771200	1.48049300	-0.89001300
C	-1.58979100	3.40941400	-1.41007400	C	-1.02161500	3.47923800	-1.10930100
C	-3.66062000	1.67350600	-2.13602700	C	-3.20295700	1.93233200	-1.93237400
C	-2.39811800	3.68314700	-2.49051500	C	-1.84410900	3.89993500	-2.13035700
C	-3.44480400	2.82578000	-2.86426700	C	-2.94615000	3.13898100	-2.55081600
H	-4.45542300	0.99873900	-2.42637900	H	-4.04119200	1.33154500	-2.26073800
H	-2.22213400	4.58492400	-3.06607600	H	-1.63546900	4.84421500	-2.62053000
H	-4.07118100	3.06033400	-3.71406200	H	-3.58273300	3.48889400	-3.35194300
H	-0.78771400	4.08002100	-1.12796900	H	-0.17714800	4.07713300	-0.78974000
P	-2.97918900	-0.14814800	-0.11889000	P	-2.56807900	-0.11316800	-0.12728500
C	-1.39581000	-1.05087400	-0.20219900	C	-1.03740900	-1.08545400	-0.33649500
H	-1.21791700	-1.21212100	-1.26964900	H	-0.89248200	-1.16102100	-1.41833700
H	-0.64089800	-0.33494900	0.12859300	H	-0.23774900	-0.44291200	0.03683900

C	-1.26983900	-2.36273500	0.57860800	C	-0.96659200	-2.46729800	0.32104000
H	-2.07961400	-3.05120600	0.31927100	H	-1.81621800	-3.08771700	0.02024400
H	-1.36070800	-2.15559400	1.64652800	H	-1.02622300	-2.35266900	1.40491800
C	0.07968400	-3.03053300	0.30880600	C	0.34068100	-3.17538800	-0.03897200
H	0.18054200	-3.30445900	-0.74434300	H	0.40795200	-3.35640300	-1.11467700
H	0.90035100	-2.35750000	0.55892100	H	1.19976200	-2.57056300	0.25296300
C	-3.51131900	0.07704000	1.60420400	C	-3.05240200	-0.03107800	1.62247600
C	-3.89739100	-1.03333400	2.36367600	C	-3.48572500	-1.18738500	2.28108000
C	-3.54530400	1.34764800	2.18103100	C	-3.00359700	1.17747700	2.31911400
C	-4.30294100	-0.87377300	3.68164600	C	-3.85673300	-1.13436500	3.61766300
H	-3.89512600	-2.02400500	1.92887300	H	-3.54675700	-2.12976300	1.75336500
C	-3.95767700	1.50104700	3.49918600	C	-3.38113900	1.22465800	3.65567300
H	-3.22882500	2.20767300	1.61138300	H	-2.65150600	2.07053800	1.82617600
C	-4.33299200	0.39464600	4.25102300	C	-3.80426000	0.07249100	4.30667700
H	-4.59759200	-1.73934600	4.26102800	H	-4.18899000	-2.03467200	4.11845400
H	-3.97676100	2.48974200	3.93908700	H	-3.33613500	2.16558600	4.18857100
H	-4.64939200	0.51919000	5.27897100	H	-4.09403200	0.11346400	5.34910800
C	-4.26663900	-1.16946000	-0.90179300	C	-3.92381300	-0.98352100	-0.97470000
C	-5.60999300	-0.98261400	-0.56178200	C	-5.24739100	-0.77658600	-0.57455300
C	-3.94239200	-2.09793100	-1.89450500	C	-3.66926500	-1.80818400	-2.07351800
C	-6.60537100	-1.71200400	-1.19880600	C	-6.29204400	-1.38625500	-1.25685400
H	-5.88092900	-0.26770500	0.20361300	H	-5.46417100	-0.13961100	0.27257300
C	-4.94012200	-2.82975900	-2.52679800	C	-4.71607700	-2.42102500	-2.75110100
H	-2.91294300	-2.25681000	-2.18461900	H	-2.65569200	-1.97548900	-2.41068400
C	-6.27216800	-2.63842900	-2.18040500	C	-6.02827300	-2.21136000	-2.34432300
H	-7.64123800	-1.55625900	-0.92621700	H	-7.31211200	-1.21648800	-0.93711400
H	-4.67392600	-3.54843400	-3.29109300	H	-4.50399100	-3.06062200	-3.59812300
H	-7.04839200	-3.20911800	-2.67408900	H	-6.84302200	-2.68884100	-2.87359900
H	0.18518400	-3.94136800	0.90101900	H	0.40861200	-4.14100200	0.46547200
C	4.27384800	0.60136900	1.05192100	C	4.73273700	0.14125700	1.02008600
C	4.76801800	0.28346000	-0.35338000	C	5.19407600	-0.10977400	-0.41076400
H	3.79863300	-0.27071600	1.49448700	H	4.22084800	-0.73606300	1.40844700
H	3.95380500	-0.14193100	-0.93364700	H	4.35322700	-0.45729200	-1.00464300
I	6.13673300	-1.43864500	-0.17283600	Br	6.38747800	-1.71367600	-0.34733100
O	1.65694100	0.24386300	0.60091800	O	2.09733400	-0.04181700	0.56443000
C	2.04388100	1.38255400	0.78085400	C	2.54661600	1.05690800	0.82815900
O	1.33109600	2.47336500	0.78308800	O	1.89518500	2.18137400	0.92252300
O	3.34456800	1.69611900	1.01683100	O	3.86397500	1.28100900	1.07378200
O	-1.09574700	1.95315000	0.42214100	O	-0.55940400	1.82464400	0.55657100
H	0.32301400	2.26914400	0.61562600	H	0.87608200	2.04694500	0.75278000
H	5.08690000	0.93742900	1.69214800	H	5.57362100	0.38854300	1.66476100
C	5.41204600	1.43727100	-1.11310200	C	5.88161700	1.05873200	-1.10481900
H	5.71641100	1.07514000	-2.09713300	H	6.15427700	0.74227700	-2.11379300
H	4.61202700	2.16553300	-1.28731400	H	5.11694300	1.83412700	-1.21980800
C	6.58301600	2.13993000	-0.43123800	C	7.09755800	1.65139600	-0.39703700
H	6.28435500	2.60390700	0.50988800	H	6.83284500	2.08127800	0.57013800
H	7.40138600	1.44759100	-0.22690000	H	7.87169700	0.89937200	-0.23771900
H	6.96886900	2.93138600	-1.07576900	H	7.52736400	2.45154900	-1.00168600
**V(O$_b$)-β-I**				**V(O$_b$)-β-Br**			
C	-1.92156400	2.19717700	0.82623900	C	-1.34933200	2.14838500	0.89534100
C	-3.02143000	1.62672600	0.10668400	C	-2.48619500	1.66120100	0.17221700
C	-1.90044100	3.61706700	0.92261700	C	-1.25776100	3.56062100	1.04707900
C	-4.03512900	2.42984700	-0.44164000	C	-3.46615300	2.53310100	-0.33006800
C	-2.90510500	4.38257200	0.37524900	C	-2.23079500	4.39480900	0.54432900
C	-3.98633200	3.80204000	-0.30655600	C	-3.34779500	3.89505500	-0.14369700
H	-4.85710900	1.97579600	-0.97953100	H	-4.31607600	2.14004900	-0.87255500
H	-2.86019700	5.46124700	0.47458100	H	-2.13162900	5.46527700	0.68469900
H	-4.76818100	4.42033300	-0.72599200	H	-4.10327500	4.56684200	-0.52768200
H	-1.07270700	4.07490900	1.44967100	H	-0.40214400	3.95710200	1.57931200
P	-2.94918700	-0.13222200	-0.12969300	P	-2.50537000	-0.08889300	-0.12980600
C	-1.38288000	-0.58650100	-0.94998200	C	-0.97776300	-0.58594500	-0.99671400
H	-1.39149000	-0.05575000	-1.90651000	H	-0.97541700	-0.01430400	-1.92941000
H	-0.60034600	-0.11927000	-0.34894800	H	-0.16392300	-0.18333900	-0.39048400
C	-1.09276700	-2.07690700	-1.15107900	C	-0.76305000	-2.07815400	-1.26794100
H	-1.91566000	-2.56663500	-1.68020400	H	-1.61770000	-2.50499000	-1.80125800
H	-1.01313800	-2.56125700	-0.17610100	H	-0.68833600	-2.60885100	-0.31701100
C	0.21055600	-2.28186600	-1.92472000	C	0.51463900	-2.30943100	-2.07618000

H	0.14295700	-1.86197300	-2.93130800	H	0.44988200	-1.84001900	-3.06089200
H	1.04128500	-1.79225600	-1.41538000	H	1.37743500	-1.88520200	-1.56161700
C	-3.17271800	-1.09304400	1.39733200	C	-2.74898800	-1.09214300	1.36642300
C	-3.44226800	-2.46440000	1.32306900	C	-3.06928200	-2.44941400	1.24881900
C	-3.08198700	-0.47914400	2.64786100	C	-2.62635300	-0.52417800	2.63562100
C	-3.61352300	-3.20876500	2.48211500	C	-3.25785800	-3.22565000	2.38399700
H	-3.53300300	-2.95489500	0.36317100	H	-3.18625500	-2.90325400	0.27381900
C	-3.25930200	-1.22931900	3.80428400	C	-2.82153700	-1.30571500	3.76809800
H	-2.84959700	0.57229200	2.71636200	H	-2.35714000	0.51572600	2.73724200
C	-3.52252700	-2.59126400	3.72487700	C	-3.13438100	-2.65390600	3.64573300
H	-3.82154200	-4.26882400	2.41321700	H	-3.50511700	-4.27454500	2.28190400
H	-3.18391400	-0.74492500	4.76920900	H	-2.72113200	-0.85701200	4.74788000
H	-3.65704700	-3.17165100	4.62894400	H	-3.28255100	-3.25921400	4.53112800
C	-4.32950900	-0.61048700	-1.21636700	C	-3.92712900	-0.45855500	-1.20504500
C	-5.59799400	-0.83923400	-0.67416000	C	-5.19421600	-0.65078600	-0.64592800
C	-4.16181000	-0.68461300	-2.60148500	C	-3.78911100	-0.48455600	-2.59513100
C	-6.67250100	-1.14139100	-1.50016900	C	-6.29699300	-0.87077200	-1.46066100
H	-5.74862400	-0.78283000	0.39568400	H	-5.32154500	-0.62989500	0.42820300
C	-5.23812400	-0.99080300	-3.42535000	C	-4.89368700	-0.70912000	-3.40781700
H	-3.19442900	-0.50176400	-3.04853600	H	-2.82305100	-0.32676500	-3.05452500
C	-6.49397900	-1.22050600	-2.87672100	C	-6.14820600	-0.90345400	-2.84259400
H	-7.64895200	-1.31549700	-1.06678700	H	-7.27210500	-1.01727400	-1.01425700
H	-5.09298300	-1.04802000	-4.49640500	H	-4.77168500	-0.73029700	-4.48306900
H	-7.33144900	-1.45913300	-3.51985300	H	-7.00773400	-1.07834400	-3.47704500
H	0.44029900	-3.34451300	-2.02184400	H	0.68987300	-3.37625200	-2.22661200
C	4.47377800	1.11217100	0.14178600	C	4.97368600	0.74262900	0.09065600
C	4.53019500	-0.40179900	0.32315100	C	4.93999600	-0.77944700	0.20106300
H	5.37268900	1.54684400	0.58174300	H	5.90249500	1.09275000	0.54330900
H	4.41028900	-0.67011800	1.36808400	H	4.78312800	-1.08855100	1.22979400
I	6.47169300	-1.22329600	-0.22644000	Br	6.68144600	-1.56366000	-0.31632000
O	1.77700100	0.47424100	0.00911300	O	2.24951600	0.25275200	-0.02991000
C	2.14464900	1.31973200	0.80448200	C	2.66826800	1.05634100	0.78359800
O	1.39347600	2.01841800	1.61139400	O	1.96334700	1.77292700	1.61548000
O	3.43778700	1.67452600	0.98905100	O	3.98051900	1.33432300	0.96470100
O	-1.03769000	1.42830100	1.35274700	O	-0.49683300	1.31642600	1.37567300
H	0.38835400	1.77898300	1.50072800	H	0.94545700	1.58883700	1.51235700
C	4.35447100	1.57947800	-1.30675000	C	4.88420900	1.27493300	-1.33711100
H	5.17959200	1.12699000	-1.86241700	H	5.66575400	0.77648400	-1.91609200
H	3.42992700	1.18477900	-1.72988200	H	3.92732400	0.97383400	-1.76577000
C	4.40699400	3.09859300	-1.45758700	C	5.06129200	2.78927400	-1.42875300
H	4.32926300	3.38262600	-2.50852600	H	4.29241700	3.31665000	-0.86287900
H	3.59169500	3.58252000	-0.91865800	H	6.03333200	3.09871400	-1.03661000
H	5.34620200	3.50379700	-1.07274300	H	5.00099900	3.12132200	-2.46665500
H	3.80876500	-0.92983400	-0.28655300	H	4.20322800	-1.23616700	-0.44651100
**V(O$_c$)-α-I**				**V(O$_c$)-α-Br**			
C	-1.20160600	-1.86747700	-0.18096100	C	-0.79437100	-1.88278800	-0.39132800
C	-2.61270300	-1.69848300	-0.00793100	C	-2.19848900	-1.69540000	-0.18286100
C	-0.71414500	-3.20215000	-0.13035900	C	-0.34215200	-3.22803400	-0.47601000
C	-3.46543800	-2.79960200	0.16995700	C	-3.07777600	-2.78708700	-0.10111300
C	-1.57043200	-4.26617100	0.04738700	C	-1.22411100	-4.28241400	-0.39088100
C	-2.95358100	-4.08131700	0.19276000	C	-2.60009000	-4.07759400	-0.20976200
H	-4.52919200	-2.64715700	0.29746300	H	-4.13562200	-2.62118600	0.05525900
H	-1.16495500	-5.27124800	0.07369300	H	-0.84493700	-5.29518300	-0.46732000
H	-3.61077800	-4.92953600	0.32729500	H	-3.27823600	-4.91782100	-0.14964200
H	0.35126900	-3.35652300	-0.24704800	H	0.71745900	-3.39712700	-0.62164700
P	-3.17944000	-0.01489400	0.07441700	P	-2.72214900	-0.01540300	0.08119900
C	-2.31183200	0.86303900	1.41680900	C	-1.80408400	0.70485000	1.48163000
H	-2.55687700	0.30607000	2.32603000	H	-2.03032300	0.05940900	2.33558300
H	-1.24825900	0.69605200	1.23501300	H	-0.74932300	0.54548400	1.24889700
C	-2.59274400	2.35815200	1.59783800	C	-2.05595700	2.17771200	1.82142500
H	-3.66702300	2.54719100	1.68494300	H	-3.12320500	2.36980000	1.96748000
H	-2.24754600	2.89713100	0.71352900	H	-1.73565600	2.79786200	0.98193500
C	-1.87186800	2.90309700	2.83243700	C	-1.28188400	2.58922300	3.07535200
H	-2.23687000	2.42447400	3.74474800	H	-1.62223400	2.02886200	3.94988000
H	-0.79897600	2.72054100	2.76532600	H	-0.21553100	2.39923800	2.95023000
C	-3.02974800	0.88217000	-1.49918900	C	-2.58229100	1.03865700	-1.39300800
C	-3.67144500	2.11548300	-1.66067000	C	-3.23577600	2.27596700	-1.42493400

C	-2.28855100	0.35197400	-2.55641300	C	-1.83211600	0.63129700	-2.49768300
C	-3.56511600	2.80901800	-2.85834300	C	-3.13470000	3.09330100	-2.54230200
H	-4.26613900	2.53403600	-0.85985300	H	-3.83548100	2.60221400	-0.58571200
C	-2.19033900	1.04884700	-3.75463000	C	-1.73888400	1.45257800	-3.61497300
H	-1.77308100	-0.58809000	-2.43591000	H	-1.30353600	-0.30902400	-2.47328700
C	-2.82342000	2.27616300	-3.90720700	C	-2.38540500	2.68208400	-3.63932500
H	-4.06428500	3.76275900	-2.97207500	H	-3.64351700	4.04862000	-2.55597700
H	-1.61031400	0.63090800	-4.56722700	H	-1.15219200	1.12928800	-4.46511900
H	-2.74096500	2.81706000	-4.84151300	H	-2.30703600	3.31939000	-4.51111200
C	-4.95910800	-0.03897700	0.45768400	C	-4.49338000	-0.03961500	0.50047100
C	-5.89355500	-0.19946800	-0.57015000	C	-5.45304600	-0.09101900	-0.51552800
C	-5.41100400	0.03478900	1.77776900	C	-4.91466500	-0.07355200	1.83201300
C	-7.24976900	-0.27959700	-0.28246700	C	-6.80402300	-0.16935300	-0.20404100
H	-5.56286500	-0.26069000	-1.59841200	H	-5.14705400	-0.06762800	-1.55304000
C	-6.76911200	-0.04097000	2.06220600	C	-6.26762200	-0.14672100	2.14073200
H	-4.71096300	0.14945900	2.59368200	H	-4.19373000	-0.04542300	2.63717500
C	-7.69013200	-0.19777800	1.03371900	C	-7.21381300	-0.19430500	1.12436800
H	-7.96170400	-0.40480400	-1.08811600	H	-7.53586600	-0.20943700	-1.00052800
H	-7.10493400	0.02199400	3.08922100	H	-6.57971600	-0.16724300	3.17691200
H	-8.74782200	-0.25641000	1.25684900	H	-8.26746600	-0.25122200	1.36625100
H	-2.03727200	3.97761900	2.93178400	H	-1.42520100	3.65103500	3.28476000
C	4.29298900	-0.10334900	1.32796600	C	4.77186600	-0.35302100	1.15901200
C	4.82444300	0.55585700	0.06147000	C	5.29162800	0.34944400	-0.08959600
H	4.01690800	-1.13676200	1.13623100	H	4.46987000	-1.37014600	0.92379400
H	4.12015500	0.40462800	-0.75175200	H	4.56143900	0.25928000	-0.88885700
I	6.54531900	-0.65255800	-0.60697400	Br	6.82067400	-0.75252300	-0.76053700
O	1.87077200	-0.52098900	0.42548300	O	2.32553500	-0.65667400	0.28121600
C	1.91830900	0.39967100	1.35796000	C	2.41381400	0.20563000	1.26527300
O	0.99118000	1.03608100	1.80521300	O	1.51148200	0.83430800	1.76999800
O	3.16591300	0.62871800	1.84327400	O	3.67668100	0.37852200	1.73437700
O	-0.45928900	-0.83720000	-0.38823500	O	-0.02628900	-0.85738300	-0.50951600
H	0.88663500	-0.67778900	0.08571200	H	1.33269100	-0.76715800	-0.04924300
H	5.03196300	-0.07187500	2.12642300	H	5.53394200	-0.37453400	1.93560500
C	5.18505000	2.03208400	0.18057200	C	5.70083600	1.80596500	0.08800000
H	5.54896400	2.37766600	-0.78907900	H	6.05374300	2.17877500	-0.87580100
H	4.24103800	2.55832600	0.36039900	H	4.77996200	2.35367900	0.31389900
C	6.17898300	2.40884200	1.27601100	C	6.73661300	2.09551800	1.17158600
H	5.79396400	2.17267300	2.26911300	H	6.36462500	1.83962100	2.16478100
H	7.13198700	1.89319700	1.14619300	H	7.66090700	1.54254900	0.99808600
H	6.37423600	3.48213000	1.24948000	H	6.97985000	3.15925000	1.17987800
**V(O_c)-β-I**				**V(O_c)-β-Br**			
C	1.13941200	-1.13875100	1.45557300	C	0.61648400	-1.08995400	1.43677200
C	2.55175400	-1.13230000	1.21842700	C	2.02283100	-1.16631600	1.17817400
C	0.65750200	-2.11905400	2.36626400	C	0.08229700	-2.07190500	2.31568000
C	3.40865100	-2.02769900	1.87825500	C	2.82642600	-2.14256200	1.78858100
C	1.51786900	-2.98847300	2.99921400	C	0.89097400	-3.02081100	2.90086000
C	2.90139400	-2.95119800	2.76998500	C	2.27060500	-3.06570800	2.65132200
H	4.47294400	-2.00279500	1.68424100	H	3.88716300	-2.18116100	1.57835100
H	1.11540300	-3.71789400	3.69300900	H	0.44999700	-3.74916200	3.57198800
H	3.56247800	-3.63853200	3.27987000	H	2.89116300	-3.81531800	3.12295300
H	-0.40806300	-2.15192100	2.55624500	H	-0.98049100	-2.04169600	2.52096200
P	3.11891700	-0.00884000	-0.03901000	P	2.64858100	-0.03459000	-0.04440000
C	2.25600800	-0.34950000	-1.60891700	C	1.74463800	-0.25764100	-1.61199300
H	2.49686100	-1.38925800	-1.85004600	H	1.91412700	-1.30068000	-1.89570600
H	1.19153300	-0.33268500	-1.36573000	H	0.68726700	-0.17992900	-1.35041600
C	2.54663200	0.57151500	-2.79818700	C	2.07738000	0.68876400	-2.77011000
H	3.62116900	0.62562400	-2.99800000	H	3.15063800	0.68418400	-2.98336200
H	2.21988100	1.58426000	-2.55449900	H	1.81681100	1.70954700	-2.48390600
C	1.81207800	0.09314100	-4.05214100	C	1.29951100	0.30469800	-4.03031700
H	2.16206900	-0.89512700	-4.36112400	H	1.58242800	-0.69164200	-4.37949200
H	0.73930000	0.02551800	-3.86927500	H	0.22677700	0.29896300	-3.83528800
C	2.96252500	1.74406300	0.41745600	C	2.61935900	1.70576700	0.47961700
C	3.62645000	2.71874100	-0.33634300	C	3.35507000	2.65738100	-0.23600700
C	2.18695500	2.13344300	1.51081700	C	1.87243700	2.10842400	1.58810300
C	3.51146000	4.06114800	-0.00267000	C	3.34003600	3.99031100	0.15083100
H	4.24370400	2.43512500	-1.17824000	H	3.95087700	2.36227500	-1.08932900
C	2.07964300	3.47885400	1.84203800	C	1.86592900	3.44377600	1.97295100

H	1.65029500	1.39184200	2.08193100	H	1.27935500	1.38711900	2.12860800
C	2.73734600	4.44271000	1.08783400	C	2.59525000	4.38499800	1.25699700
H	4.02785500	4.80699600	-0.59323600	H	3.91165300	4.71833100	-0.41037400
H	1.47305500	3.77152800	2.68930700	H	1.28151000	3.74702600	2.83200800
H	2.64768600	5.48987200	1.34806500	H	2.58394600	5.42469100	1.55901900
C	4.90019700	-0.29569600	-0.28189300	C	4.40312000	-0.43114200	-0.32485700
C	5.83095400	0.33578600	0.54887800	C	5.38160000	0.08504000	0.53049500
C	5.35638000	-1.19901500	-1.24519100	C	4.78755800	-1.30308000	-1.34655400
C	7.18780700	0.07354500	0.41297000	C	6.71579200	-0.26112500	0.36301000
H	5.49743500	1.03554300	1.30346900	H	5.10272600	0.76048700	1.32829700
C	6.71506800	-1.45627500	-1.38191500	C	6.12419500	-1.64414100	-1.51456500
H	4.65818000	-1.71119100	-1.89252900	H	4.04985700	-1.72286900	-2.01628800
C	7.63259700	-0.82055500	-0.55439800	C	7.08963700	-1.12411400	-0.66113700
H	7.89685700	0.56929900	1.06341400	H	7.46286800	0.14476600	1.03287800
H	7.05408400	-2.15516700	-2.13564000	H	6.40861900	-2.31720800	-2.31303900
H	8.69081200	-1.02184700	-0.66197000	H	8.13049200	-1.39082700	-0.79310700
H	1.98189400	0.78039700	-4.88320100	H	1.50348500	1.01011900	-4.83813700
C	-4.60450400	0.49129100	-0.11679700	C	-5.09859300	0.73041500	0.07010200
C	-4.44733500	-0.74523500	-0.99695900	C	-4.95123400	-0.42206600	-0.92026800
H	-4.07004000	0.42017900	0.82184400	H	-4.54735400	0.57703000	0.98888600
H	-4.30733000	1.38658500	-0.65367200	H	-4.81324800	1.67138400	-0.38993800
H	-5.20896500	-0.70766400	-1.77743300	H	-5.73036400	-0.31304700	-1.67647100
I	-6.68293900	0.85268500	0.42521000	Br	-6.98807700	0.96578900	0.60480700
O	-3.23181600	-0.64366500	-1.78979800	O	-3.75209100	-0.25089400	-1.72169500
C	-1.98651100	-0.57599700	-1.26238900	C	-2.49751200	-0.23903000	-1.21180100
O	-1.03826900	-0.51950900	-2.01254300	O	-1.55981900	-0.15268100	-1.97213700
O	-1.94603000	-0.57141600	0.05111100	O	-2.43793400	-0.31532500	0.09871100
C	-4.56553000	-2.07741600	-0.26194400	C	-5.05989100	-1.81199400	-0.30044900
H	-5.51818100	-2.06537400	0.27391100	H	-6.00416500	-1.84306900	0.24900500
H	-3.77868300	-2.14267700	0.49011600	H	-4.26156900	-1.94111200	0.43127100
C	-4.51775700	-3.28662100	-1.19416000	C	-5.02769000	-2.93841000	-1.33148500
H	-3.57483600	-3.33439100	-1.74009600	H	-4.09295300	-2.94057900	-1.89339700
H	-4.62438000	-4.21286100	-0.62689800	H	-5.12716400	-3.90893500	-0.84230900
H	-5.32570600	-3.25185000	-1.92919800	H	-5.84610500	-2.84262700	-2.04944100
O	0.39167300	-0.27218000	0.86970100	O	-0.08090600	-0.15400400	0.89619700
H	-0.96207700	-0.47981100	0.40379400	H	-1.44561900	-0.27403300	0.44117200
**TS3(O_a)-α-I**				**TS3(O_a)-α-Br**			
C	0.14934800	-0.82314500	0.40255400	C	0.17977100	0.80969300	0.68366900
C	1.40015200	-1.20944900	-0.12093400	C	-1.07391800	1.25217800	0.21392200
C	-0.94705700	-1.67561900	0.26515800	C	1.23968700	1.71347800	0.77479500
C	1.53888500	-2.46290300	-0.73365500	C	-1.25757700	2.60475800	-0.10662000
C	-0.79212500	-2.90176600	-0.35934500	C	1.04091600	3.04122800	0.43434200
C	0.44970500	-3.30884600	-0.84735500	C	-0.20708800	3.49788100	0.01017300
H	2.49645200	-2.76506100	-1.13458800	H	-2.21762800	2.95009100	-0.46441300
H	-1.65777100	-3.54422300	-0.45978600	H	1.87863700	3.72328500	0.50561300
H	0.56220200	-4.27347900	-1.32341400	H	-0.35314200	4.53951800	-0.24184400
H	-1.91959500	-1.38500800	0.64541700	H	2.22020500	1.38722200	1.10376300
P	2.72668600	-0.00109600	-0.12349700	P	-2.33384700	0.02053400	-0.12738300
C	2.14496800	1.47693100	-1.01355800	C	-1.64785500	-1.17963300	-1.31229900
H	1.91124400	1.12157300	-2.02165900	H	-1.39970800	-0.58847100	-2.19887300
H	1.18316000	1.74806700	-0.57171300	H	-0.69028400	-1.50914000	-0.90202400
C	3.06775700	2.69955100	-1.06865500	C	-2.50448800	-2.39397600	-1.68798500
H	4.06382000	2.42337700	-1.42787900	H	-3.49965000	-2.08413000	-2.02124800
H	3.19200700	3.10089700	-0.06102100	H	-2.64428600	-3.01989400	-0.80455700
C	2.47214600	3.78025100	-1.97358100	C	-1.82277700	-3.21405800	-2.78553900
H	2.40947500	3.43549400	-3.00834300	H	-1.74135000	-2.64024200	-3.71176100
H	1.46384000	4.04260700	-1.65215200	H	-0.81499100	-3.50152600	-2.48473000
C	3.31266800	0.37705800	1.54888100	C	-2.96087900	-0.77202600	1.37665100
C	4.42675900	1.20790400	1.71922500	C	-4.05103100	-1.64630600	1.28898100
C	2.68138000	-0.16288400	2.67059100	C	-2.38866900	-0.51084100	2.62261500
C	4.88957000	1.50170900	2.99357000	C	-4.54912700	-2.25724300	2.43050100
H	4.94289300	1.61867600	0.86223600	H	-4.52108800	-1.84446700	0.33527800
C	3.15329300	0.13165800	3.94393800	C	-2.89563500	-1.12275600	3.76257200
H	1.81968400	-0.80326900	2.55646400	H	-1.54541300	0.15786300	2.70629300
C	4.25222500	0.96510300	4.10763200	C	-3.97049700	-1.99744100	3.66860000
H	5.74925100	2.14769700	3.11574700	H	-5.39014000	-2.93395200	2.35277600
H	2.65477200	-0.28917600	4.80726300	H	-2.44315400	-0.91665200	4.72368900

H	4.61446000	1.19563500	5.10133900
C	4.13980200	-0.71271400	-1.01428300
C	5.08070400	-1.48792700	-0.32926800
C	4.27284200	-0.54111200	-2.39528000
C	6.13486000	-2.07778000	-1.01403400
H	4.99425300	-1.62926700	0.73971400
C	5.33186200	-1.12895400	-3.07513000
H	3.55475400	0.04709100	-2.94955000
C	6.26314300	-1.89750100	-2.38663200
H	6.85656700	-2.67618000	-0.47340300
H	5.42691400	-0.98556800	-4.14355200
H	7.08754400	-2.35453700	-2.91875000
H	3.09211700	4.67826400	-1.95742700
C	-3.49304400	1.53585600	0.91211900
C	-4.23480600	1.16380300	-0.34082700
H	-3.91347800	2.46042700	1.31866200
I	-5.05903600	-1.45827300	0.45729800
O	-3.03053300	2.65113600	-1.21138100
C	-1.94013000	2.46944900	-0.61372100
O	-0.79142600	2.77380400	-0.88262600
O	-2.11069300	1.73609200	0.61129100
O	0.09340100	0.35419200	1.04621100
H	-0.79395000	0.80102300	0.97580900
H	-3.54777900	0.76010100	1.66829900
C	-5.57377500	1.75069900	-0.64928800
H	-6.25601800	1.52378800	0.17288000
H	-5.43586000	2.83677500	-0.64183600
C	-6.16529700	1.30506600	-1.98091000
H	-7.12192900	1.79949600	-2.15773400
H	-5.49622500	1.56164600	-2.80412100
H	-6.33082000	0.22710800	-1.98685700
H	-3.75437700	0.51251900	-1.04564300

H	-4.35971900	-2.47536700	4.55850900
C	-3.75173900	0.86328000	-0.88800500
C	-4.74780900	1.42350900	-0.08218300
C	-3.83273800	1.01233500	-2.27558800
C	-5.80508100	2.11716500	-0.65588000
H	-4.70175000	1.31694000	0.99331200
C	-4.89485800	1.70298800	-2.84521700
H	-3.07115600	0.59583900	-2.92015500
C	-5.88121300	2.25597200	-2.03718900
H	-6.56993600	2.54733200	-0.02246700
H	-4.94915100	1.80809200	-3.92090500
H	-6.70809600	2.79376200	-2.48299300
H	-2.39705400	-4.11663700	-3.00164900
C	3.89263200	-1.38755400	0.76581300
C	4.56220900	-0.70257800	-0.39148100
H	4.41193100	-2.32495900	0.98459500
Br	5.11199500	1.55849200	0.86813700
O	3.52136100	-2.07180400	-1.53783200
C	2.42215800	-2.13309300	-0.92746100
O	1.30830300	-2.48308700	-1.27369700
O	2.53243400	-1.66902000	0.42623100
O	0.27033400	-0.47913100	1.05100600
H	1.17826500	-0.86866600	0.92013800
H	3.88563300	-0.76517000	1.65424000
C	5.95352100	-1.06067500	-0.80319000
H	6.61266900	-0.92211300	0.05634000
H	5.94406300	-2.13323800	-1.01967900
C	6.46911100	-0.28018200	-2.00604200
H	7.47698900	-0.60547700	-2.26951000
H	5.82631200	-0.43963500	-2.87372500
H	6.49980200	0.78652200	-1.78238500
H	4.00947200	0.03576700	-0.93906600

TS3(O_a)-β-I

C	0.07114200	0.62037000	-0.69000900
C	1.34024600	1.18515500	-0.44799100
C	-1.01851300	1.46031400	-0.93076000
C	1.50149400	2.57657900	-0.49295200
C	-0.84146500	2.83394500	-0.95110300
C	0.41718600	3.39842700	-0.74743000
H	2.47330800	3.01402100	-0.31021900
H	-1.70115400	3.46653300	-1.13201400
H	0.54701700	4.47171700	-0.77505500
H	-2.00132300	1.04156800	-1.10715700
P	2.66772100	0.09450500	0.07767000
C	2.12518600	-0.76373500	1.58800000
H	1.93149800	0.03659000	2.30847900
H	1.14877700	-1.20060900	1.36567300
C	3.05070100	-1.82665900	2.19152300
H	4.06370600	-1.43510500	2.32514200
H	3.12215700	-2.67045700	1.50283300
C	2.50208000	-2.31517800	3.53379100
H	2.49367100	-1.50932200	4.27142300
H	1.47896100	-2.67614400	3.42581100
C	3.17849000	-1.04401200	-1.23508200
C	4.26697600	-1.89898300	-1.02296900
C	2.52048300	-1.06213100	-2.46589700
C	4.67789800	-2.76592200	-2.02501700
H	4.80376800	-1.88522500	-0.08415100
C	2.94074700	-1.92945800	-3.46672600
H	1.67829200	-0.41038800	-2.64374300
C	4.01423500	-2.78310700	-3.24756900
H	5.51779100	-3.42590800	-1.85127800
H	2.42187500	-1.93897700	-4.41626700
H	4.33593400	-3.46056000	-4.02816500
C	4.11773100	1.11749400	0.46235100
C	5.03194900	1.44271000	-0.54440500
C	4.30702100	1.63105300	1.74887200

TS3(O_a)-β-Br

C	0.28141800	-0.66881800	-0.76912000
C	-1.01319100	-1.17597400	-0.53480500
C	1.31878800	-1.55252800	-1.07576900
C	-1.25299500	-2.55170600	-0.65561600
C	1.06462000	-2.91113500	-1.16866700
C	-0.22050500	-3.41623700	-0.97512900
H	-2.24513800	-2.94443600	-0.48149900
H	1.88602500	-3.57783900	-1.39827600
H	-0.41105900	-4.47731900	-1.06113500
H	2.32235100	-1.18304200	-1.24573100
P	-2.27321700	-0.04895200	0.07241000
C	-1.66434200	0.71575000	1.60865900
H	-1.48007500	-0.12362500	2.28569700
H	-0.67844900	1.12849100	1.38357800
C	-2.53957400	1.77725200	2.28440000
H	-3.56127400	1.41166600	2.42494500
H	-2.60053100	2.65535100	1.63873100
C	-1.94372200	2.18276800	3.63431300
H	-1.94476100	1.34250100	4.33248200
H	-0.91146600	2.51411400	3.51884500
C	-2.75690700	1.17727200	-1.16980200
C	-3.81751100	2.05019500	-0.89784600
C	-2.10245200	1.25434000	-2.40024000
C	-4.20515700	2.99189800	-1.83984000
H	-4.35089700	1.99179200	0.04111000
C	-2.49923900	2.19687700	-3.34101900
H	-1.28058300	0.59092900	-2.62351400
C	-3.54509400	3.06723100	-3.06208200
H	-5.02348800	3.66495200	-1.61950700
H	-1.98322400	2.25175300	-4.29057600
H	-3.84792300	3.80313100	-3.79592700
C	-3.76194900	-1.01987200	0.44419300
C	-4.71772900	-1.24914200	-0.55033700
C	-3.94020800	-1.59167100	1.70760700

C	6.11594500	2.26484200	-0.26647600	C	-5.83156500	-2.03415900	-0.28299200
H	4.90180700	1.05178100	-1.54459900	H	-4.59630100	-0.81297100	-1.53273000
C	5.39548800	2.44917100	2.02297900	C	-5.05822800	-2.37222300	1.97138600
H	3.61035400	1.39984500	2.54271600	H	-3.21182600	-1.43587800	2.49136000
C	6.30021400	2.76695200	1.01677000	C	-6.00428600	-2.59450400	0.97754500
H	6.81739800	2.51057500	-1.05311000	H	-6.56487500	-2.20565600	-1.06022700
H	5.53451300	2.83718700	3.02355900	H	-5.18805800	-2.80594400	2.95428100
H	7.14800300	3.40443500	1.23251700	H	-6.87500600	-3.20310200	1.18521200
H	3.12072900	-3.12286100	3.92858500	H	-2.52518500	2.99036200	4.08222100
C	-3.96276600	-0.25990000	1.20485500	C	4.28825900	-0.24263000	1.06673900
C	-3.54745700	-1.27419500	0.16555400	C	3.97975900	0.90030400	0.12923900
H	-4.85021800	-0.38901800	1.79417700	H	5.19351600	-0.26726000	1.64189000
H	-3.37223600	0.61973900	1.37301400	H	3.61669100	-1.07426900	1.15366800
H	-3.50983300	-0.81346700	-0.81832700	H	3.88551800	0.53253100	-0.88901500
I	-5.41497200	1.38281900	-0.40676800	Br	5.45582100	-1.69974900	-0.62386100
O	-2.17403900	-1.64881300	0.45041600	O	2.65933500	1.39670900	0.47328000
C	-1.89175000	-1.65967200	1.84709300	C	2.39106600	1.28772500	1.86647700
O	-0.77569000	-2.03065700	2.16353400	O	1.32524500	1.73712300	2.24606500
O	-2.86375100	-1.24167100	2.53552700	O	3.31955900	0.69408300	2.48588700
C	-4.45716600	-2.49652600	0.12460800	C	5.02033900	2.01225500	0.17703200
H	-5.47121600	-2.13186200	-0.05711100	H	5.98319900	1.55130900	-0.05650000
H	-4.45556100	-2.96657300	1.11112600	H	5.08262000	2.39128600	1.20031900
C	-4.06419700	-3.50586400	-0.95064400	C	4.73381900	3.15005700	-0.79884000
H	-3.05242400	-3.88151500	-0.79097400	H	3.77303100	3.62192900	-0.58760000
H	-4.74366500	-4.35984600	-0.94465300	H	5.50627400	3.91816900	-0.73265700
H	-4.10601800	-3.05713400	-1.94638900	H	4.71448900	2.78859000	-1.83015200
O	-0.00824300	-0.71954800	-0.70635100	O	0.43435500	0.66446400	-0.71435000
H	-0.88579700	-1.07052300	-0.38140400	H	1.33248200	0.94899800	-0.38029800
**TS3(O_b)-α-I**				**TS3(O_b)-α-Br**			
C	-1.65774100	2.25632000	-0.33526000	C	-1.03540200	2.12559300	-0.71238000
C	-2.66040100	1.38750700	-0.82786200	C	-2.05908700	1.22322800	-1.08753700
C	-1.41109700	3.45634700	-1.02242300	C	-0.68045000	3.14870800	-1.60696000
C	-3.41615400	1.74831800	-1.95283800	C	-2.72877800	1.38426500	-2.30953900
C	-2.16165200	3.78591700	-2.13521800	C	-1.34690400	3.28119800	-2.81041000
C	-3.17511300	2.94469500	-2.60203800	C	-2.38145000	2.41198700	-3.16638200
H	-4.18390700	1.08371700	-2.32465600	H	-3.51273600	0.69490600	-2.59168200
H	-1.96050900	4.71744500	-2.64995300	H	-1.06197500	4.07836600	-3.48603400
H	-3.76019200	3.21900600	-3.46908400	H	-2.90033100	2.53240200	-4.10744400
H	-0.62910400	4.10959700	-0.65824800	H	0.11787800	3.82437700	-1.32957400
P	-2.82087400	-0.21899200	-0.05237900	P	-2.35505300	-0.18475200	-0.02132500
C	-1.19661500	-1.05245000	-0.09610700	C	-0.78244200	-1.08350400	0.20785300
H	-0.93674900	-1.11617000	-1.15668000	H	-0.47390300	-1.38175200	-0.79834400
H	-0.49337200	-0.34245500	0.34212000	H	-0.06093300	-0.33146500	0.53028600
C	-1.08228300	-2.42406100	0.57787400	C	-0.78218100	-2.28608200	1.15790700
H	-1.83744400	-3.11093200	0.18525700	H	-1.55049500	-3.00815900	0.86707500
H	-1.27405400	-2.31934800	1.64730100	H	-1.02783500	-1.95029900	2.16707400
C	0.31053700	-3.02112000	0.37135900	C	0.58516000	-2.97129300	1.17380300
H	0.52359000	-3.17637000	-0.68843100	H	0.84866200	-3.35746400	0.18693100
H	1.08210100	-2.36286700	0.77240400	H	1.36794800	-2.27522000	1.47742200
C	-3.47747600	-0.12444200	1.63343000	C	-3.09847700	0.28348300	1.56340700
C	-3.85024300	-1.30037900	2.29609300	C	-3.57359500	-0.71268000	2.42535400
C	-3.62119900	1.10420700	2.28038100	C	-3.20941600	1.62440900	1.93554500
C	-4.34776800	-1.24400800	3.58986700	C	-4.14040400	-0.36867000	3.64396400
H	-3.76719800	-2.25957600	1.80322700	H	-3.51558400	-1.75582300	2.14517400
C	-4.12412600	1.15319600	3.57471800	C	-3.78201000	1.96152700	3.15589000
H	-3.32971900	2.01765100	1.78490000	H	-2.83971500	2.40281400	1.28558000
C	-4.48343200	-0.01697000	4.23120300	C	-4.24347300	0.96914300	4.01118600
H	-4.63104700	-2.15789300	4.09563900	H	-4.50291000	-1.14512200	4.30499100
H	-4.22743500	2.10968400	4.07003900	H	-3.85978900	3.00332700	3.43785300
H	-4.87026100	0.02515200	5.24133800	H	-4.68479400	1.23613900	4.96289200
C	-4.00580100	-1.19719000	-1.01913800	C	-3.54025900	-1.28359300	-0.84829300
C	-5.37313100	-1.12016300	-0.73646000	C	-4.91431200	-1.08985100	-0.67568000
C	-3.57422700	-1.98296300	-2.09184200	C	-3.09601700	-2.29046700	-1.71028100
C	-6.28913900	-1.81993800	-1.51087900	C	-5.82488000	-1.89219800	-1.35052100
H	-5.72473800	-0.51679300	0.08966500	H	-5.27531800	-0.31579900	-0.01190600
C	-4.49368200	-2.68508400	-2.86042300	C	-4.01046200	-3.09374400	-2.37928200
H	-2.52360300	-2.05242300	-2.33771200	H	-2.03898600	-2.45399500	-1.86792600

C	-5.85091800	-2.60471500	-2.57144800	C	-5.37463300	-2.89602500	-2.20047000
H	-7.34496500	-1.75255300	-1.28305900	H	-6.88606100	-1.73335600	-1.20952200
H	-4.14740000	-3.29395800	-3.68530600	H	-3.65486000	-3.87384100	-3.03961600
H	-6.56564700	-3.15289600	-3.17167200	H	-6.08557800	-3.52349200	-2.72245400
H	0.39441400	-3.98588500	0.87336300	H	0.58794700	-3.80964200	1.87172800
C	4.34083800	1.23913600	0.80100300	C	4.82041500	0.97981100	0.68016200
C	3.76094100	0.14490800	-0.06986000	C	4.12626800	-0.21227200	0.05310700
H	4.54643900	0.86048700	1.79984800	H	5.05665500	0.77620000	1.72159600
H	3.53009700	-0.79701100	0.38668100	H	3.84912000	-1.02707100	0.69195300
I	6.27982500	-1.26353600	0.10289100	Br	6.39582900	-1.56804200	0.38887300
O	1.95146200	0.78089400	0.37833700	O	2.40392400	0.62059000	0.36456500
C	2.13761700	1.97476500	0.76633100	C	2.67230300	1.84967400	0.54443000
O	1.25432300	2.82477400	1.00639300	O	1.84556000	2.78135600	0.63557300
O	3.41579000	2.35486000	0.91291300	O	3.97166600	2.16064500	0.62527800
O	-1.01603000	1.90252200	0.76444500	O	-0.47676600	1.97373800	0.47605000
H	-0.04580200	2.33833500	0.87366700	H	0.51675400	2.36188500	0.54795000
H	5.25027500	1.65355100	0.38298200	H	5.72944900	1.24128300	0.15287600
C	3.68962700	0.22721400	-1.57543100	C	4.02757800	-0.43727200	-1.43798100
H	4.01910000	-0.73176000	-1.97275200	H	4.32352900	-1.46776400	-1.62784400
H	2.63168300	0.32781800	-1.83567200	H	2.97005500	-0.35433000	-1.70411900
C	4.48700900	1.35538000	-2.22675000	C	4.85438100	0.50335200	-2.31168600
H	4.17753600	2.33799300	-1.86546200	H	4.58429600	1.55029900	-2.15870600
H	5.55378500	1.23217300	-2.03624900	H	5.91808600	0.38192600	-2.10471200
H	4.33590100	1.33760700	-3.30743700	H	4.68931400	0.27044000	-3.36517200
**TS3(O_b)-β-I**				**TS3(O_b)-β-Br**			
C	1.70299800	-2.31917600	0.19199500	C	1.12827800	-2.29336600	0.25432600
C	2.75230000	-1.58828300	-0.41350400	C	2.21882700	-1.64234000	-0.36894800
C	1.53839600	-3.67079300	-0.15194900	C	0.87702100	-3.63834500	-0.06170700
C	3.63088200	-2.22241400	-1.30389800	C	3.05174000	-2.34874500	-1.24877600
C	2.41122800	-4.27206000	-1.03921700	C	1.70585300	-4.31140300	-0.93953800
C	3.46838200	-3.56029100	-1.61199100	C	2.80369600	-3.67959500	-1.52929100
H	4.43331700	-1.66159600	-1.76301900	H	3.88603400	-1.84927100	-1.72167000
H	2.27221100	-5.31666300	-1.28948400	H	1.49948100	-5.34969200	-1.16870800
H	4.14844400	-4.04543800	-2.29862600	H	3.44891600	-4.22035600	-2.20796800
H	0.72150800	-4.22257200	0.29429800	H	0.02826800	-4.12794200	0.39742000
P	2.82005300	0.17588500	-0.10861300	P	2.39522700	0.12171800	-0.10605700
C	1.21409900	0.91112200	-0.57155700	C	0.83680700	0.94264100	-0.58450100
H	1.08267100	0.66471700	-1.62937400	H	0.68428500	0.67303300	-1.63371700
H	0.46025200	0.33385500	-0.03318500	H	0.04987100	0.43194700	-0.02644600
C	1.02826200	2.41341400	-0.33117900	C	0.74900400	2.46112800	-0.39150600
H	1.83754300	2.98225100	-0.79786700	H	1.59461800	2.96125000	-0.87233800
H	1.07631800	2.61592500	0.74043000	H	0.80744800	2.69367800	0.67351700
C	-0.31667100	2.89516400	-0.87683100	C	-0.55983000	3.01386200	-0.95757200
H	-0.38349300	2.74693900	-1.95687900	H	-0.63570800	2.83256200	-2.03199900
H	-1.14436900	2.35600400	-0.41529900	H	-1.42320900	2.55024600	-0.47987600
C	3.27323100	0.57792300	1.59874100	C	2.87793400	0.53366700	1.59054200
C	3.56985300	1.90347900	1.93994100	C	3.25018700	1.84698600	1.90360700
C	3.33557600	-0.41430100	2.57887500	C	2.88918700	-0.44056800	2.59041400
C	3.91343000	2.22780800	3.24426300	C	3.61651600	2.17776200	3.20010300
H	3.54498600	2.68376000	1.19121500	H	3.26742500	2.61171500	1.13881200
C	3.68432900	-0.08230700	3.88225500	C	3.26133500	-0.10258800	3.88571400
H	3.09917700	-1.43837300	2.33327500	H	2.59521200	-1.45451600	2.36605100
C	3.96972900	1.23512900	4.21718600	C	3.62103800	1.20334600	4.19291900
H	4.13821800	3.25515700	3.49969700	H	3.89975900	3.19576600	3.43387800
H	3.72550800	-0.85728500	4.63631200	H	3.26245100	-0.86340000	4.65516800
H	4.23683000	1.49006800	5.23476100	H	3.90606500	1.46339900	5.20431700
C	4.11454700	0.88186900	-1.16777600	C	3.72714400	0.72038900	-1.18496700
C	5.44091300	0.90690800	-0.72525800	C	5.05351300	0.68622500	-0.74330700
C	3.81466400	1.34667500	-2.45142200	C	3.45154000	1.16086300	-2.48263200
C	6.44634100	1.39007000	-1.55207300	C	6.08263200	1.08807700	-1.58452800
H	5.69014900	0.55251500	0.26596900	H	5.28466200	0.34932400	0.25831400
C	4.82309200	1.83388200	-3.27313400	C	4.48374200	1.56699700	-3.31871100
H	2.79837200	1.33169000	-2.82016500	H	2.43548400	1.18980100	-2.85128200
C	6.13876100	1.85603700	-2.82532200	C	5.79930300	1.53111600	-2.87143400
H	7.46936500	1.40410600	-1.19932100	H	7.10540600	1.05674400	-1.23213800
H	4.57851600	2.19533200	-4.26338500	H	4.25769300	1.91081800	-4.31958300
H	6.92282000	2.23639500	-3.46735700	H	6.60210700	1.84831100	-3.52452000

H	-0.45498900	3.95848800	-0.67720200	H	-0.62464800	4.09061800	-0.79597700
C	-4.37168600	-1.21067400	0.22391800	C	-4.81934800	-0.70096900	0.19799000
C	-3.66120400	0.11659100	0.06377500	C	-3.98666700	0.55744800	0.06713200
H	-5.23800500	-1.09276300	0.86843500	H	-5.68129200	-0.50587500	0.82908500
H	-3.48690200	0.74037500	0.91999100	H	-3.80536000	1.17285900	0.92778000
I	-5.92226400	1.70810300	-0.16854900	Br	-5.98641400	2.13755300	-0.24595800
O	-1.94012900	-0.71896500	0.19580800	O	-2.36737900	-0.39329600	0.25963700
C	-2.19079400	-1.80058100	0.81650100	C	-2.71415700	-1.47148000	0.84264900
O	-1.34969600	-2.58249800	1.30456600	O	-1.94378600	-2.32123400	1.33281700
O	-3.48697700	-2.11062700	0.96333400	O	-4.03140700	-1.68889900	0.93768800
O	0.94146500	-1.69416200	1.07336100	O	0.41062400	-1.60413700	1.12510400
H	-0.03047900	-2.11210600	1.19475600	H	-0.58936100	-1.94834400	1.24328200
C	-4.76482200	-1.85477700	-1.09910700	C	-5.24792200	-1.28815200	-1.13951600
H	-5.37820900	-1.13003800	-1.63763600	H	-5.77969000	-0.49814100	-1.67261700
H	-3.85735600	-2.01440500	-1.69059600	H	-4.34923200	-1.52586300	-1.71862800
C	-5.52769200	-3.16595800	-0.92970000	C	-6.13456500	-2.52290600	-1.00266300
H	-5.78189300	-3.59157900	-1.90210000	H	-5.63005300	-3.31927300	-0.45374000
H	-4.93910800	-3.90256100	-0.38128200	H	-7.05864900	-2.28070100	-0.47270500
H	-6.46061200	-3.00535100	-0.38460400	H	-6.40916700	-2.91064000	-1.98539800
H	-3.44778800	0.51869600	-0.90722100	H	-3.71398300	0.94206000	-0.89572300
**TS3(O$_c$)-α-I**				**TS3(O$_c$)-α-Br**			
C	-0.75270400	-1.27516900	-0.79167200	C	-0.31552600	-1.26788600	-0.89433100
C	-2.14212100	-1.44885200	-0.61515400	C	-1.70566100	-1.41720800	-0.70196100
C	0.03221500	-2.37848300	-1.14483500	C	0.43386300	-2.36603700	-1.33109300
C	-2.72385200	-2.70263800	-0.84492500	C	-2.32377400	-2.63924400	-0.99836300
C	-0.56092100	-3.61161300	-1.35291400	C	-0.19476500	-3.56842000	-1.60431800
C	-1.93973600	-3.77940600	-1.21973200	C	-1.57492800	-3.70958900	-1.45486000
H	-3.78877500	-2.83595300	-0.71355300	H	-3.38933700	-2.75423100	-0.85553800
H	0.05880500	-4.45515400	-1.63015500	H	0.39746300	-4.40797800	-1.94620200
H	-2.39372000	-4.74497000	-1.39563900	H	-2.05731900	-4.65035500	-1.68208900
H	1.10005300	-2.24682100	-1.25876600	H	1.50246500	-2.25369800	-1.45789200
P	-3.08105100	-0.07668400	0.06467900	P	-2.59815000	-0.06500200	0.07451800
C	-2.33151900	0.38471400	1.65875900	C	-1.80899400	0.29309000	1.67610500
H	-2.40050900	-0.51991700	2.27076700	H	-1.88584700	-0.64069200	2.24166200
H	-1.26513000	0.53421900	1.47024400	H	-0.74324400	0.43150800	1.47547700
C	-2.90811000	1.59184100	2.40775700	C	-2.34809100	1.47100200	2.49570100
H	-3.99243100	1.50126000	2.52345100	H	-3.43137600	1.39317600	2.62927700
H	-2.72336800	2.49664200	1.82586000	H	-2.15845500	2.40006500	1.95491500
C	-2.24975200	1.73333000	3.78181900	C	-1.66012600	1.53398600	3.86117200
H	-2.47408100	0.87203000	4.41564200	H	-1.88614000	0.64607100	4.45657900
H	-1.16584800	1.80378800	3.68601300	H	-0.57733500	1.59200100	3.74631200
C	-3.19321800	1.31928500	-1.08365900	C	-2.70063300	1.39630200	-0.99064300
C	-3.99461900	2.41901200	-0.75375900	C	-3.48368000	2.48594100	-0.59036800
C	-2.50863000	1.30774000	-2.29969800	C	-2.02417300	1.44807300	-2.21030400
C	-4.09550600	3.49409800	-1.62474000	C	-3.57364400	3.61281800	-1.39453400
H	-4.54887100	2.43654900	0.17476100	H	-4.03291200	2.45570100	0.34073500
C	-2.61774500	2.38599000	-3.16926100	C	-2.12197000	2.57834200	-3.01258300
H	-1.88429700	0.46826800	-2.56559800	H	-1.41399000	0.61752800	-2.53129100
C	-3.40547100	3.47941500	-2.83261000	C	-2.89084800	3.66114700	-2.60558400
H	-4.71404800	4.34188300	-1.36041400	H	-4.17797500	4.45195000	-1.07536700
H	-2.07971700	2.37051600	-4.10797600	H	-1.58962700	2.61128300	-3.95408100
H	-3.48410600	4.31974000	-3.51041600	H	-2.96060700	4.54197600	-3.23089000
C	-4.78110600	-0.64807400	0.34956900	C	-4.30499600	-0.61494900	0.36273300
C	-5.73305000	-0.55259900	-0.67038500	C	-5.27709600	-0.43230100	-0.62564000
C	-5.14070700	-1.23619400	1.56585500	C	-4.65122200	-1.27376200	1.54629100
C	-7.02005700	-1.03586600	-0.47454500	C	-6.57086900	-0.89762700	-0.43070300
H	-5.47370400	-0.09701900	-1.61667700	H	-5.02764900	0.07717400	-1.54662400
C	-6.43066300	-1.71370300	1.75861100	C	-5.94755500	-1.73387500	1.73839800
H	-4.42139600	-1.32725400	2.36795500	H	-3.91672600	-1.43423600	2.32335200
C	-7.37104300	-1.61479400	0.73977700	C	-6.90831000	-1.54678100	0.75138900
H	-7.74864100	-0.95681700	-1.27087900	H	-7.31494000	-0.75004200	-1.20258900
H	-6.69899000	-2.16253300	2.70604000	H	-6.20497800	-2.23826000	2.66058000
H	-8.37594400	-1.98698100	0.89235400	H	-7.91815900	-1.90543300	0.90371200
H	-2.61517100	2.62643200	4.29131600	H	-2.00020800	2.40648800	4.42146200
C	4.20288400	0.19929300	1.57761300	C	4.71309500	0.00301900	1.47105100
C	3.94104100	0.23187500	0.08629400	C	4.39815200	0.01499600	-0.01191500
H	4.38567900	-0.82141300	1.90774000	H	4.91892100	-1.01123400	1.80491900

H	3.90888100	-0.70187600	-0.43960100	H	4.33484800	-0.92908200	-0.51640800
I	6.70372500	-0.63051200	-0.35826900	Br	6.94844100	-0.76755500	-0.42311200
O	2.04042300	0.11923800	0.44651000	O	2.53062400	-0.01946900	0.38260300
C	1.89184300	0.55420900	1.66946200	C	2.41090300	0.38959400	1.61932300
O	0.83603100	0.80471300	2.21995700	O	1.36694400	0.64530500	2.19010000
O	3.06729700	0.73112700	2.30693300	O	3.59735600	0.52773200	2.23842600
O	-0.26454200	-0.04376000	-0.64180100	O	0.20773900	-0.06112600	-0.67831400
H	0.69175900	-0.01190900	-0.29419600	H	1.16940700	-0.07488500	-0.34449300
H	5.04882400	0.81775500	1.85272500	H	5.56262800	0.63247200	1.70475000
C	4.00050800	1.49450800	-0.73970600	C	4.47883900	1.25271500	-0.87579700
H	4.55466100	1.26397500	-1.64844300	H	5.06560200	0.99035300	-1.75465000
H	2.97675100	1.72420800	-1.04951700	H	3.46574400	1.47142600	-1.22450400
C	4.61417500	2.71528000	-0.05677400	C	5.07499300	2.49256100	-0.21305800
H	4.07720400	2.98828500	0.85352500	H	4.51892500	2.79032200	0.67815000
H	5.65911600	2.52906300	0.19405300	H	6.11431800	2.31448200	0.06464500
H	4.57886700	3.57168000	-0.73224100	H	5.05410600	3.33062600	-0.91192900
**TS3(O_c)-β-I**				**TS3(O_c)-β-Br**			
C	-0.87268200	0.20646400	1.61779800	C	0.43986600	-0.22910600	1.64231400
C	-2.27272800	0.36715400	1.54083100	C	1.83245400	-0.43159100	1.53504800
C	-0.19950100	0.63583300	2.76713800	C	-0.22718600	-0.67282800	2.78974600
C	-2.97398700	0.90476600	2.62814100	C	2.53500300	-1.02562000	2.59158600
C	-0.91039300	1.17697600	3.82403300	C	0.48438100	-1.26961500	3.81587900
C	-2.29903700	1.30215800	3.76893000	C	1.86711500	-1.43745900	3.73147400
H	-4.04716800	1.02449100	2.57276300	H	3.60260000	-1.17772400	2.51246200
H	-0.37674900	1.50028600	4.70904800	H	-0.04396200	-1.60358600	4.70011700
H	-2.84561000	1.71642400	4.60498700	H	2.41412900	-1.89546700	4.54410300
H	0.87613600	0.52868300	2.81347000	H	-1.29777400	-0.53263900	2.85958100
P	-3.07358500	0.02020700	-0.02982500	P	2.61388300	-0.05800100	-0.03900200
C	-2.28658000	1.05801200	-1.30235700	C	1.77509200	-1.03613000	-1.32634100
H	-2.46602100	2.08656500	-0.97463800	H	1.92897000	-2.07831000	-1.02957100
H	-1.20876400	0.90257900	-1.20811600	H	0.70432900	-0.84941500	-1.21037100
C	-2.72340600	0.86760300	-2.75930400	C	2.19444900	-0.82043600	-2.78485500
H	-3.81061300	0.94577100	-2.85573900	H	3.27715900	-0.92863000	-2.90084500
H	-2.44499900	-0.13510100	-3.08903800	H	1.94079600	0.19812100	-3.08455700
C	-2.04732400	1.90105400	-3.66278400	C	1.47275700	-1.80918200	-3.70299900
H	-2.36042400	2.91529400	-3.40361200	H	1.75664300	-2.83902400	-3.47314100
H	-0.96252600	1.85159800	-3.56353500	H	0.39164600	-1.72669000	-3.58654400
C	-3.04427800	-1.74138300	-0.44728600	C	2.62382900	1.71607500	-0.40067600
C	-3.74413300	-2.19315300	-1.57312400	C	3.30141900	2.18481200	-1.53311100
C	-2.34889600	-2.65835700	0.34264500	C	1.98109400	2.62580900	0.44057500
C	-3.73396500	-3.53970200	-1.90613000	C	3.32127200	3.54156800	-1.82149200
H	-4.30562100	-1.50185800	-2.18675400	H	3.82201000	1.49852500	-2.18708500
C	-2.34674500	-4.00627400	0.00539600	C	2.00869100	3.98371000	0.14767300
H	-1.80024600	-2.32560800	1.21065000	H	1.44993000	2.28004000	1.31442900
C	-3.03362100	-4.44754400	-1.11823700	C	2.67330100	4.44231700	-0.98237600
H	-4.27367800	-3.87947400	-2.78047300	H	3.84310500	3.89466900	-2.70137500
H	-1.80014400	-4.70866900	0.62075800	H	1.50236000	4.68056200	0.80252800
H	-3.02507500	-5.49760800	-1.38088200	H	2.68776800	5.50037900	-1.21038800
C	-4.82256300	0.48580700	0.11899400	C	4.35195600	-0.57343000	0.06248500
C	-5.75448100	-0.43994400	0.59854800	C	5.31943000	0.31748100	0.53739000
C	-5.24468600	1.78253100	-0.18848800	C	4.73155700	-1.87512500	-0.27799000
C	-7.08344300	-0.07368300	0.76550400	C	6.64124500	-0.08765600	0.66710300
H	-5.44505000	-1.44831500	0.83869900	H	5.04359400	1.32924900	0.80263200
C	-6.57597700	2.14300000	-0.02392600	C	6.05573800	-2.27453400	-0.15058500
H	-4.54260800	2.51804900	-0.55580200	H	4.00122300	-2.58429400	-0.64212400
C	-7.49605500	1.21670200	0.45283600	C	7.01139800	-1.38252800	0.32176800
H	-7.79608500	-0.79826200	1.13740400	H	7.38159000	0.61039400	1.03564600
H	-6.89237900	3.14829300	-0.26965900	H	6.33872000	-3.28322300	-0.42183200
H	-8.53314600	1.49967300	0.57937000	H	8.04293500	-1.69563300	0.41937200
H	-2.31143800	1.72732400	-4.70722500	H	1.72761700	-1.61823800	-4.74673700
C	3.86436200	-0.04422700	0.03605900	C	-4.25922600	0.29691400	0.17270200
C	4.28119800	1.09434500	-0.87170200	C	-4.78331000	-0.71111200	-0.82992000
H	3.93731000	0.04112000	1.10298400	H	-4.33414000	0.11614800	1.22716100
H	3.67474300	-1.01767300	-0.37727100	H	-4.01772400	1.29301100	-0.14800800
H	5.03091300	0.74527800	-1.57609900	H	-5.49974000	-0.22815600	-1.48778100
I	6.43495800	-1.13358300	0.37787900	Br	-6.57861400	1.39397700	0.60379800
O	3.13453400	1.45181200	-1.70098300	O	-3.67403100	-1.09845500	-1.69891500

C	1.94433500	1.16605400	-1.13952700	C	-2.46439000	-0.93964400	-1.13458900
O	0.90241200	1.50117100	-1.66990600	O	-1.44942200	-1.30649900	-1.69479000
O	2.05593400	0.50121900	-0.01628200	O	-2.52348500	-0.35016000	0.03617000
C	4.77965400	2.32153000	-0.11981700	C	-5.39139800	-1.94965000	-0.18636600
H	5.60388600	1.99223700	0.51583500	H	-6.17404000	-1.60045300	0.48948000
H	3.98050600	2.67493900	0.54019300	H	-4.62486800	-2.43900800	0.42398200
C	5.24259300	3.44616000	-1.04192400	C	-5.96953000	-2.93316400	-1.20058100
H	4.43790400	3.78077900	-1.69786400	H	-5.20694100	-3.28255000	-1.89807900
H	5.58513700	4.30403400	-0.46045700	H	-6.39123500	-3.80420000	-0.69559700
H	6.07488000	3.11748500	-1.66859300	H	-6.76959200	-2.46680200	-1.78011000
O	-0.26543300	-0.38474000	0.58959500	O	-0.16326400	0.41339700	0.64326000
H	0.69238300	-0.07850800	0.43650700	H	-1.13430300	0.14823500	0.49577000
**VI-I**				**VI-Br**			
P	1.80406800	-0.13897900	-0.09407000	P	1.58528800	-0.13138900	-0.09159800
O	0.50016000	-2.54913600	0.75969100	O	0.18979400	-2.44033100	0.88675000
C	0.26197000	0.06856300	-1.01861900	C	0.04317300	0.12988200	-0.99546800
C	0.35945100	-0.21604300	-2.52531300	C	0.11394500	-0.14070600	-2.50571300
C	-1.01881300	-0.15587600	-3.18553000	C	-1.26494800	0.01485800	-3.14974100
O	-1.95759100	-3.46092300	0.22499200	O	-2.37585000	-2.97864700	0.29510900
C	-2.74440800	-2.57229500	0.46138200	C	-3.04779000	-1.99240600	0.49568900
I	-2.14439600	2.84901000	-0.19206800	Br	-1.80411700	3.02296600	-0.26230900
H	-0.29731600	-3.11809600	0.63273500	H	-0.67543900	-2.88992000	0.73436100
H	-0.15496100	1.06665900	-0.82210200	H	-0.33850300	1.14304600	-0.78758700
H	-0.42706300	-0.62868200	-0.54089300	H	-0.66053100	-0.55056100	-0.51521900
H	1.02113600	0.51060600	-3.00161300	H	0.81163400	0.55437200	-2.97766300
H	0.80590700	-1.19898800	-2.69536300	H	0.49919900	-1.14592700	-2.69560900
H	-1.67596200	-0.93248800	-2.78804400	H	-1.96582100	-0.72464800	-2.75583700
H	-1.49685500	0.80849600	-3.00890100	H	-1.67459700	1.00644100	-2.95296000
O	-2.56946000	-1.66247900	1.42059800	O	-2.76026300	-1.07072000	1.41517100
O	-3.87871500	-2.38380900	-0.21028400	O	-4.15508100	-1.69677600	-0.18378300
C	-4.63837300	-1.28357000	0.38702800	C	-4.77370400	-0.48890200	0.36549100
C	-3.60745000	-0.65153200	1.33114600	C	-3.67408200	0.05149500	1.28899900
H	-3.99077200	-0.47773200	2.33372000	H	-4.03284700	0.30033600	2.28494300
H	-3.16591100	0.26664600	0.93340100	H	-3.13049200	0.89861300	0.85960900
C	-5.17700500	-0.35875800	-0.68274400	C	-5.19215200	0.45270100	-0.74330100
H	-5.45571900	-1.75553400	0.93910200	H	-5.64375200	-0.83474300	0.93028100
H	-5.63931700	0.48902900	-0.16762600	H	-5.55103400	1.36896700	-0.26410100
C	-6.18521700	-1.02340600	-1.61648100	C	-6.26894700	-0.12138700	-1.66024700
H	-4.33745500	0.06189000	-1.24072400	H	-4.30463800	0.74697900	-1.30798900
H	-6.55096200	-0.30766200	-2.35352600	H	-6.54135100	0.60278700	-2.42883900
H	-5.73564700	-1.85928600	-2.15425300	H	-5.92124800	-1.02671500	-2.15963300
H	-7.04892400	-1.40412800	-1.06524300	H	-7.17528400	-0.37229900	-1.10304800
C	1.86540000	-4.18225000	-0.36749600	C	1.39527400	-4.17257800	-0.27539600
C	3.53153200	-2.10170900	-1.17993700	C	3.15592500	-2.21738400	-1.19340300
C	2.99313700	-4.44195600	-1.13055600	C	2.47019900	-4.51696100	-1.08071400
H	1.20893300	-4.98316000	-0.05174600	H	0.70351900	-4.92400000	0.08358000
C	3.83344000	-3.40783500	-1.53472700	C	3.35712700	-3.54590100	-1.53735000
H	4.16460100	-1.29794400	-1.52809700	H	3.82260200	-1.46164900	-1.58363000
H	3.21841500	-5.46246100	-1.41372600	H	2.61697600	-5.55400600	-1.35530300
H	4.70949100	-3.61585100	-2.13380000	H	4.19130700	-3.81958500	-2.16903400
C	1.56241400	-2.87195300	0.00362200	C	1.19417200	-2.84042000	0.08498300
C	2.40310400	-1.81530000	-0.40585000	C	2.08254500	-1.84719800	-0.37791000
C	1.55657000	0.14384400	1.67216000	C	1.39376700	0.21086200	1.67149800
C	0.65050900	1.12092300	2.09079000	C	0.56579000	1.25987400	2.07834700
C	2.32376700	-0.55205100	2.60967600	C	2.13239500	-0.51077300	2.61201100
C	0.51969000	1.39286000	3.44748000	C	0.48528200	1.57561800	3.42980300
H	0.02801600	1.66733300	1.38722200	H	-0.02936000	1.83062100	1.36856700
C	2.18690600	-0.26876900	3.96084100	C	2.04511100	-0.18313800	3.95719100
H	3.01211900	-1.32466200	2.29181000	H	2.75989900	-1.33634100	2.30127000
C	1.28587100	0.70523300	4.38037300	C	1.22225000	0.86167300	4.36666400
H	-0.19025900	2.14591900	3.76407300	H	-0.16150600	2.38595800	3.74052000
H	2.77728800	-0.81428700	4.68585300	H	2.61281000	-0.74830100	4.68530000
H	1.17872300	0.92247300	5.43575700	H	1.15354400	1.11432000	5.41742200
C	3.02402700	1.08842900	-0.63164900	C	2.84993000	1.02463400	-0.68060600
C	2.60655400	2.21678500	-1.34303500	C	2.45194300	2.19716200	-1.33037100
C	4.35881600	0.98992100	-0.22157200	C	4.20043200	0.83685100	-0.36523300
C	3.51992400	3.21468100	-1.66290400	C	3.40072300	3.14825700	-1.68732800

H	1.57062300	2.34359300	-1.62622100	H	1.40710300	2.39603800	-1.53041100
C	5.26555900	1.98680100	-0.54958600	C	5.14157500	1.78801600	-0.73109000
H	4.69211700	0.14419300	0.36572400	H	4.52117600	-0.04238300	0.17788700
C	4.84813000	3.09838700	-1.27528400	C	4.74338100	2.94224000	-1.39836000
H	3.18346800	4.08676400	-2.20792900	H	3.08073800	4.05505600	-2.18349000
H	6.29602800	1.90062800	-0.22986200	H	6.18438800	1.63246300	-0.48624100
H	5.55620800	3.87789500	-1.52590900	H	5.47860400	3.68557600	-1.67917600
H	-0.93218900	-0.31197900	-4.26242200	H	-1.20097300	-0.12987700	-4.22985100

11 Crystallographic Data

Data were collected on a Bruker Kappa APEX II Duo diffractometer. The structure was solved by direct methods (SHELXS-97: Sheldrick, G. M. *Acta Cryst.* **2008**, *A64*, 112.) and refined by full-matrix least-squares procedures on F^2 (SHELXL-2014: Sheldrick, G. M. *Acta Cryst.* **2015**, *C71*, 3.). XP (Bruker AXS) was used for graphical representations.

Crystal data: $C_{21}H_{22}IOP$, M = 448.25, orthorhombic, space group *Pbca*, $\bar{1}a$ = 13.3336(10), b = 16.8996(12), c = 17.2615(13) Å, V = 3889.6(5) Å^3, T = 150(2) K, Z = 8, 19862 reflections measured, 3434 independent reflections (R_{int} = 0.0367), final R values ($I > 2\sigma(I)$): R_1 = 0.0293, wR_2 = 0.0670, final R values (all data): R_1 = 0.0306, wR_2 = 0.0677, 234 parameters.

CCDC 2024624 contains the supplementary crystallographic data for this paper. These data were provided free of charge by the joint Cambridge Crystallographic Data Centre and Fachinformationszentrum Karlsruhe Access Structures service www.ccdc.cam.ac.uk/structures.

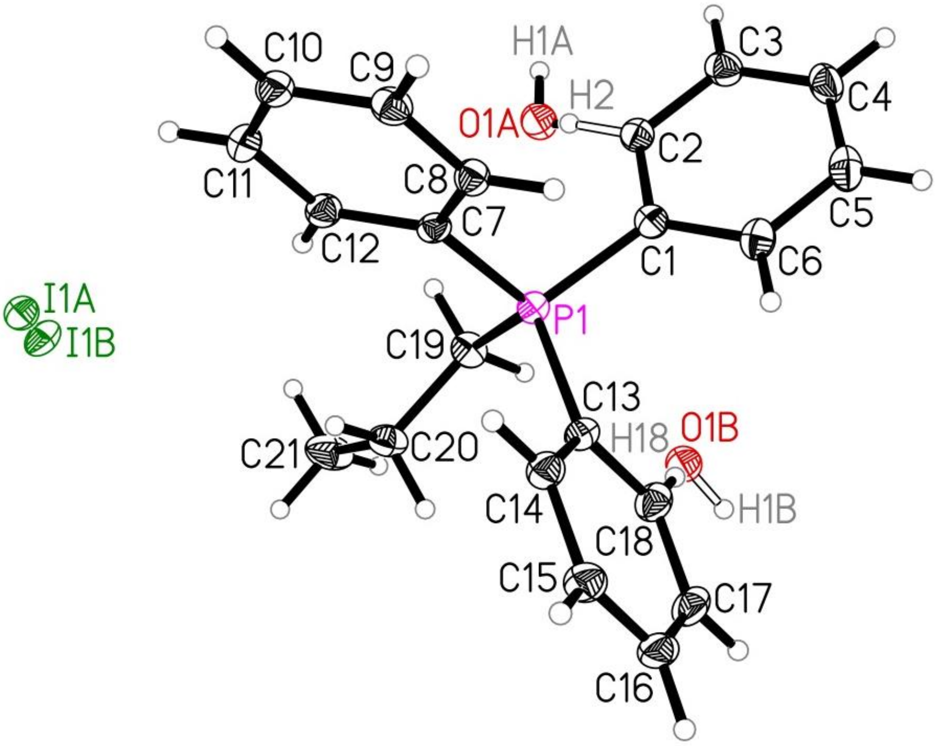

Figure S23. Molecular structure of **8** in the crystal. Displacement ellipsoids correspond to 30% probability. The anion (I1A/I1B) and the OH group (O1A-H1A/O1B-H1B; H18/H2) are disordered over two sites with occupancies of 0.908(4):0.092(4). Lower occupancy sites are depicted with unfilled lines.

12. NMR-spectra of catalysts and synthesized carbonates

^{1}H NMR (2-Hydroxyphenyl)diphenyl(propyl)phosphonium bromide (7)

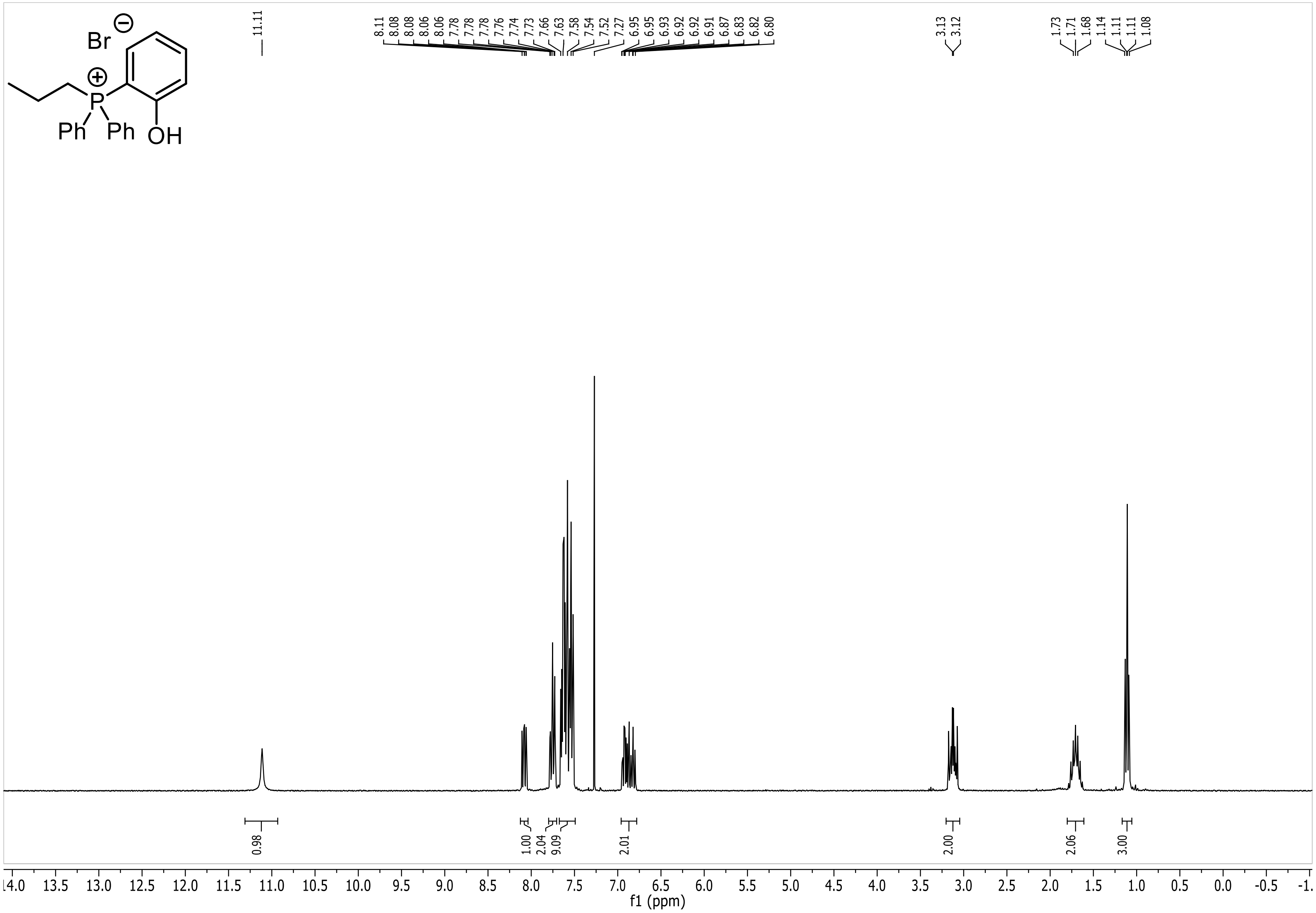

^{31}P NMR (2-Hydroxyphenyl)diphenyl(propyl)phosphonium bromide (7)

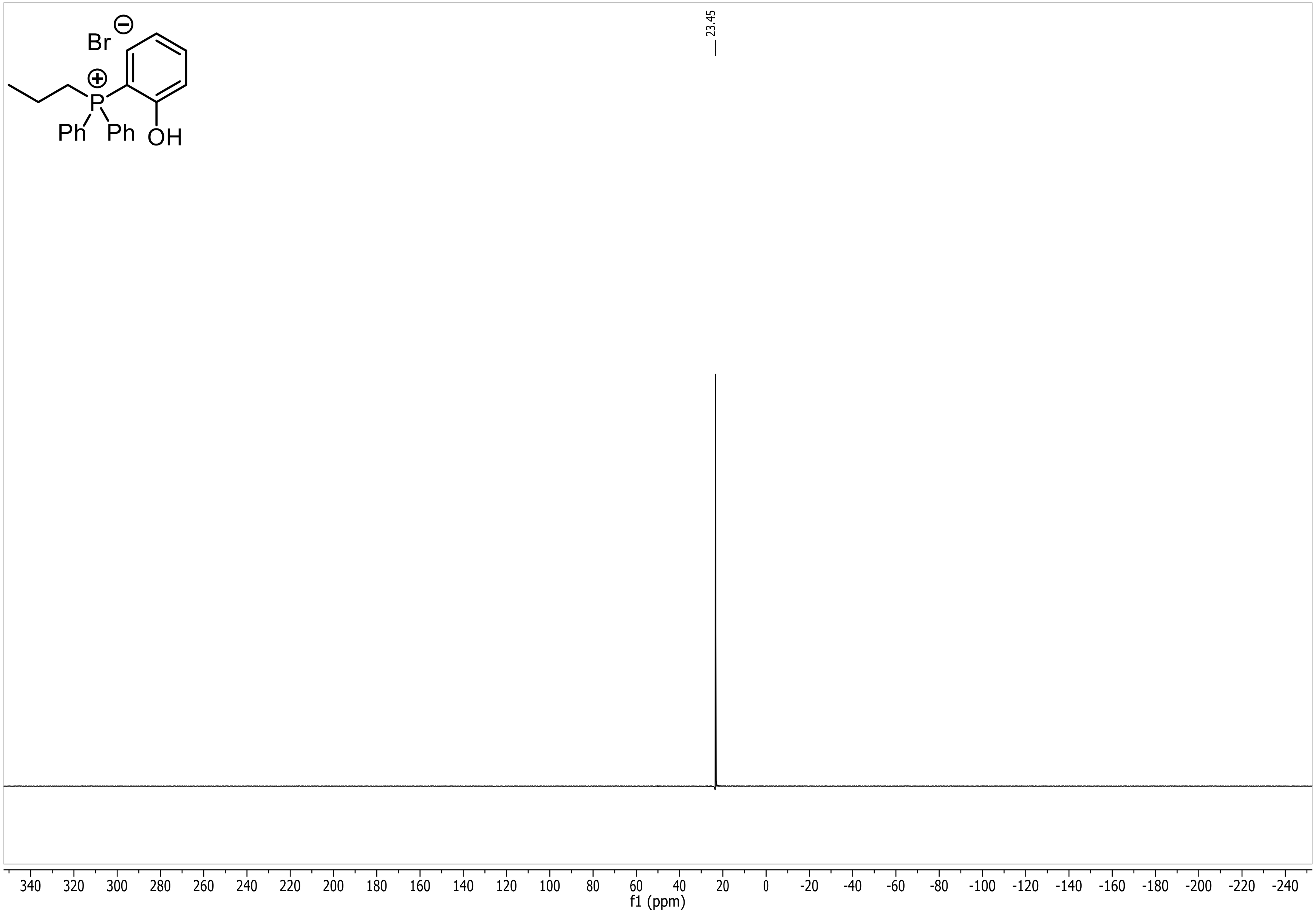

^{1}H NMR (2-Hydroxyphenyl)diphenyl(propyl)phosphonium iodide (8)

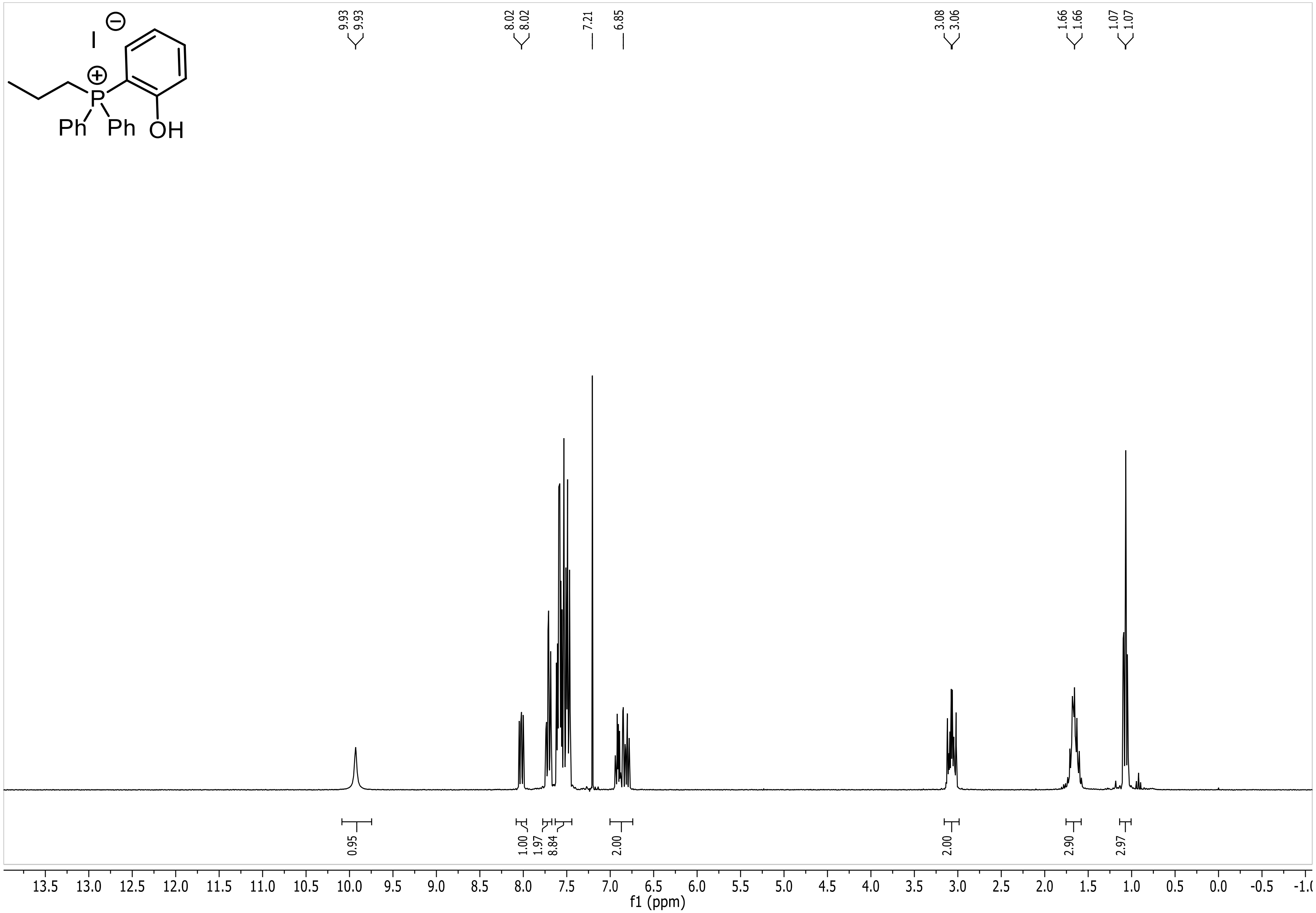

^{31}P NMR (2-Hydroxyphenyl)diphenyl(propyl)phosphonium iodide (8)

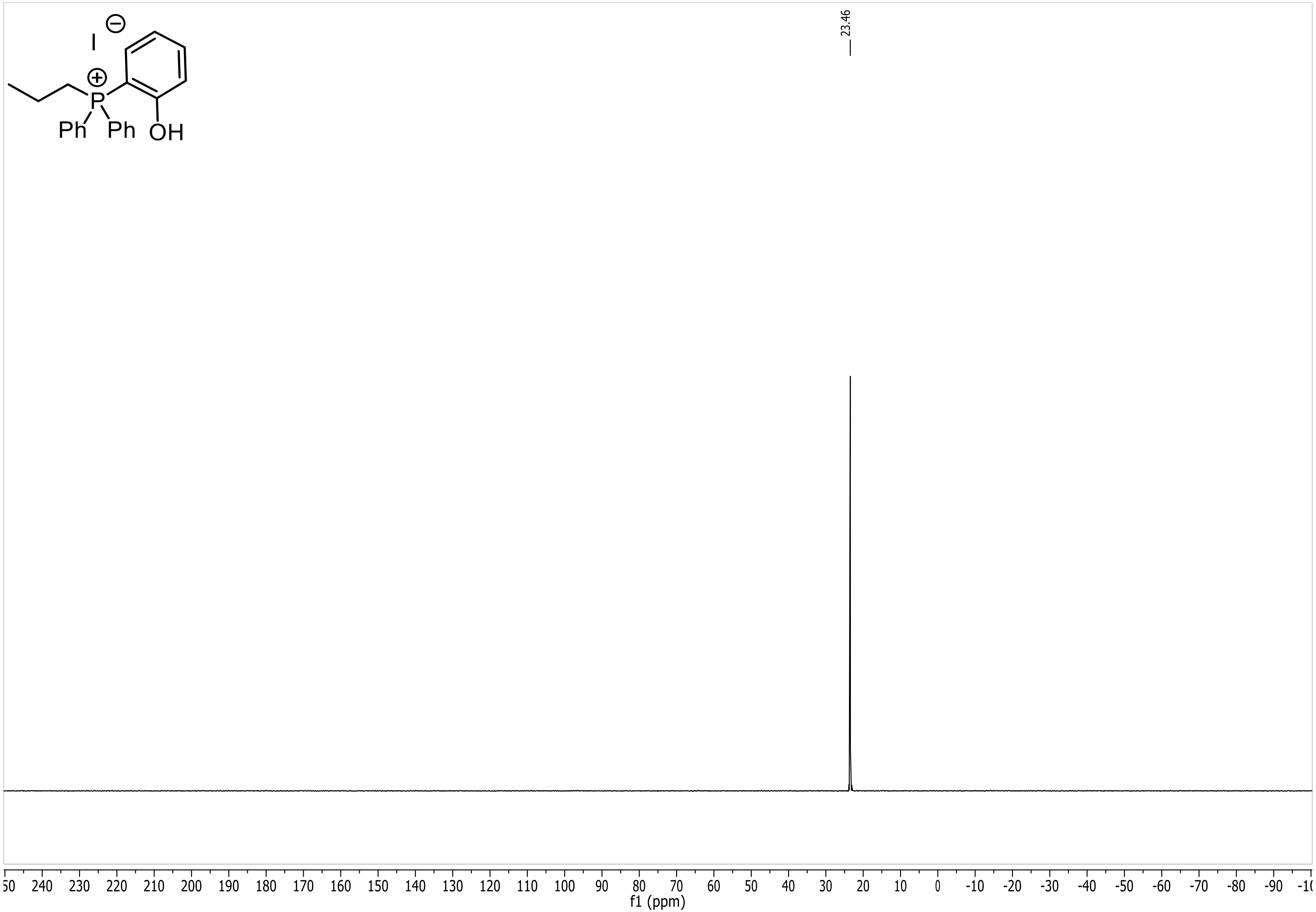

^{1}H NMR 4-Ethyl-1,3-dioxalan-2-one (2a)

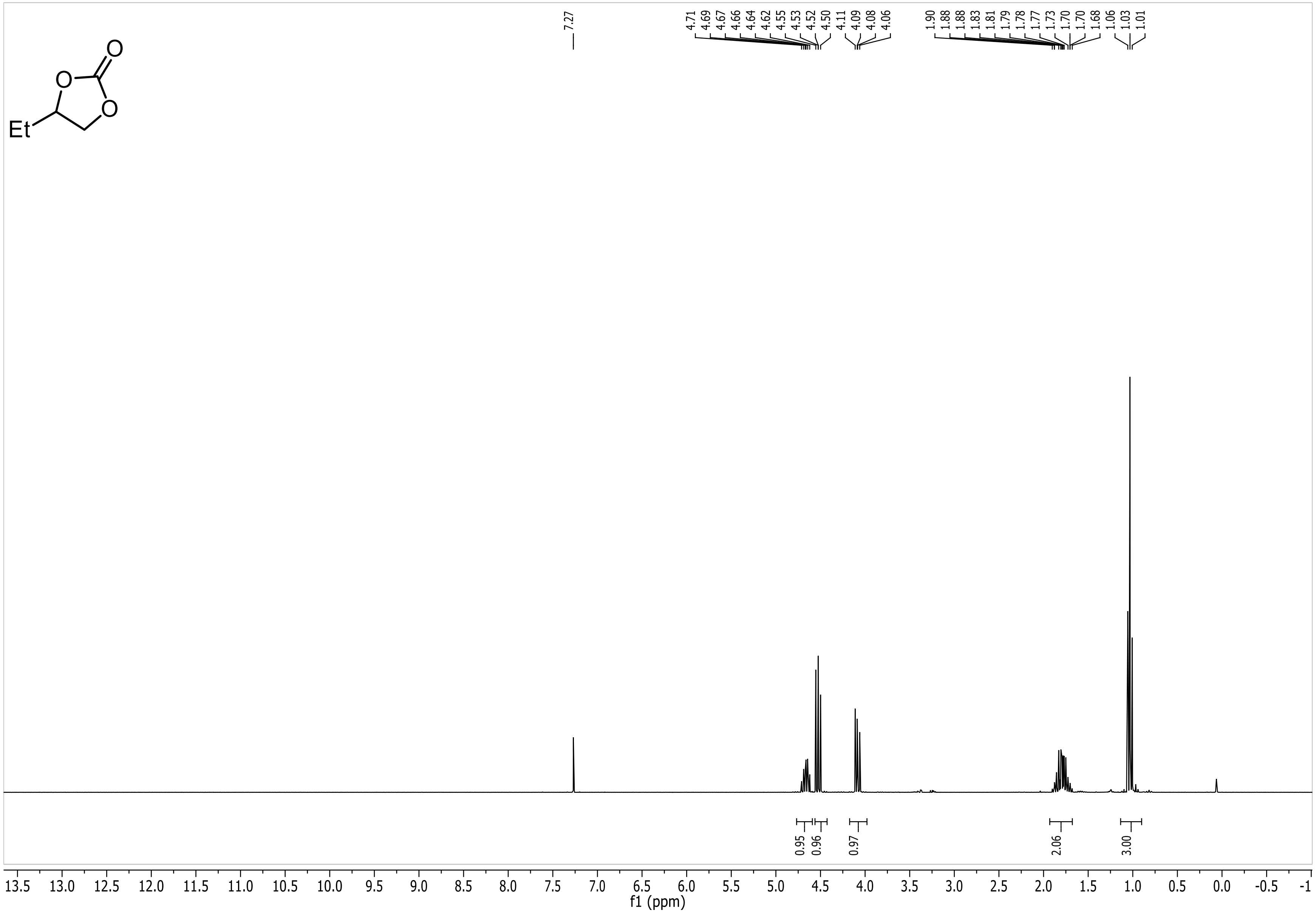

^{1}H NMR 4-Methyl-1,3-dioxalan-2-one (2b)

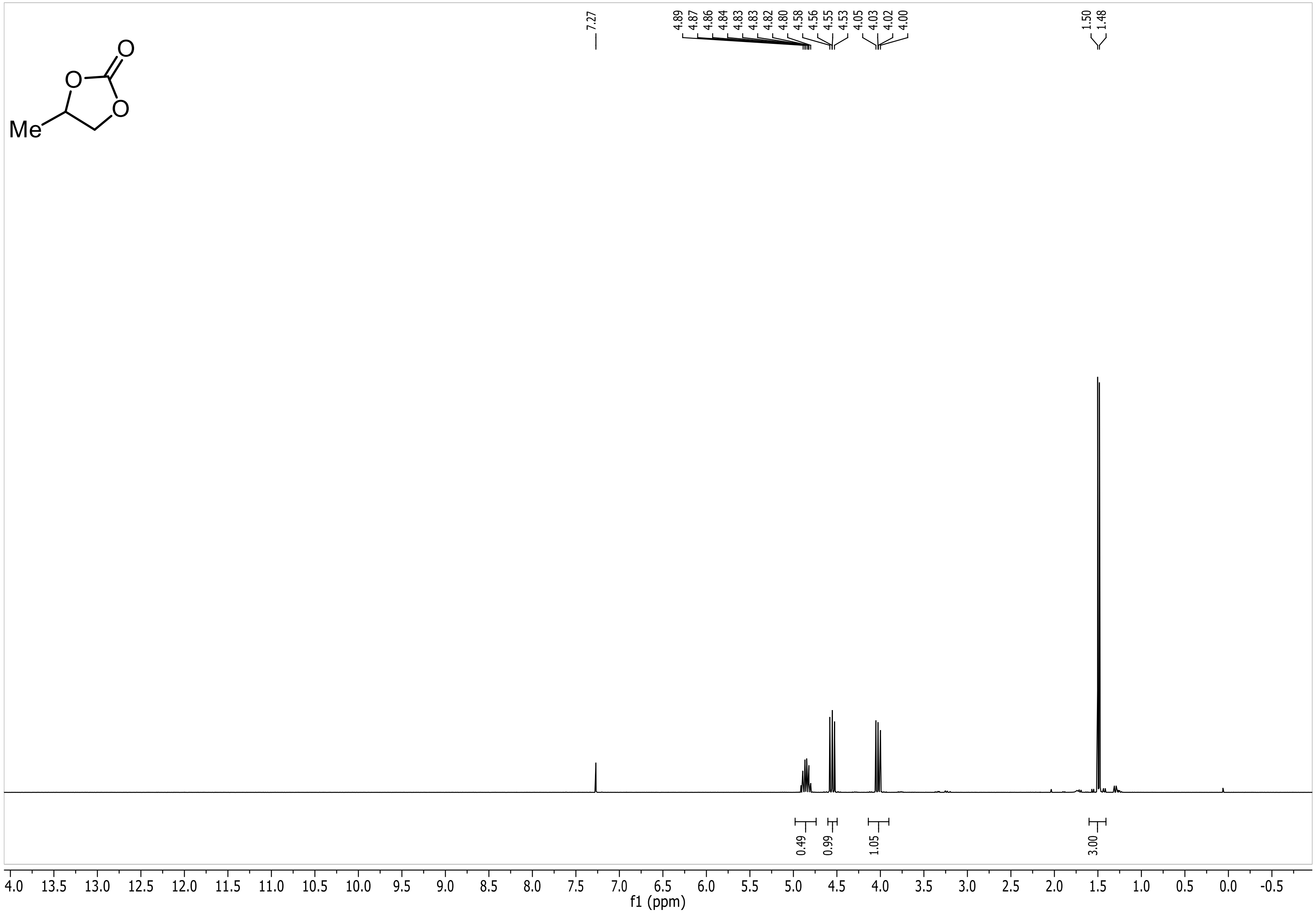

¹H NMR 4-Butyl-1,3-dioxolan-2-one (2c)

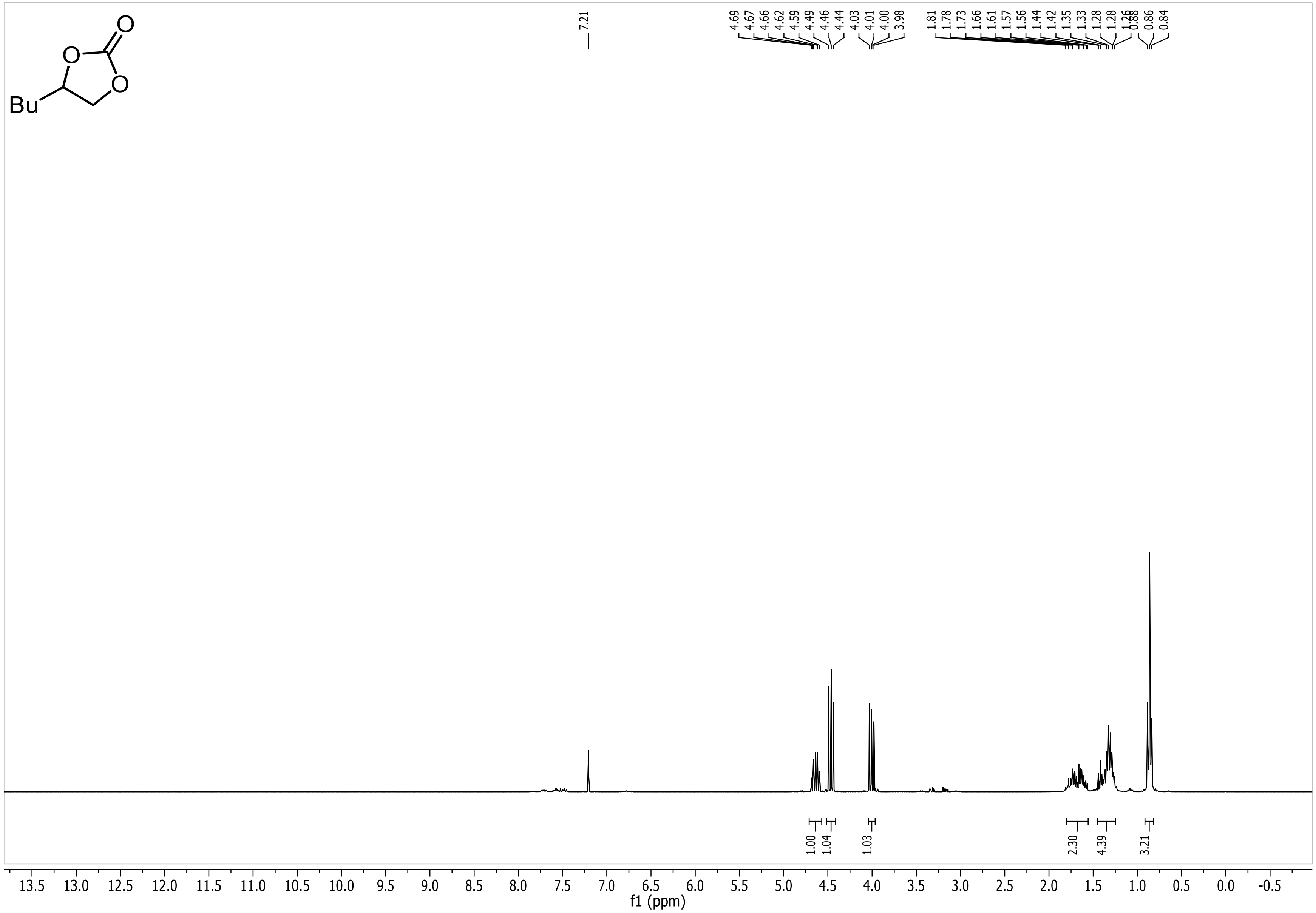

^{1}H NMR 4-Hexyl-1,3-dioxolan-2-one (2d)

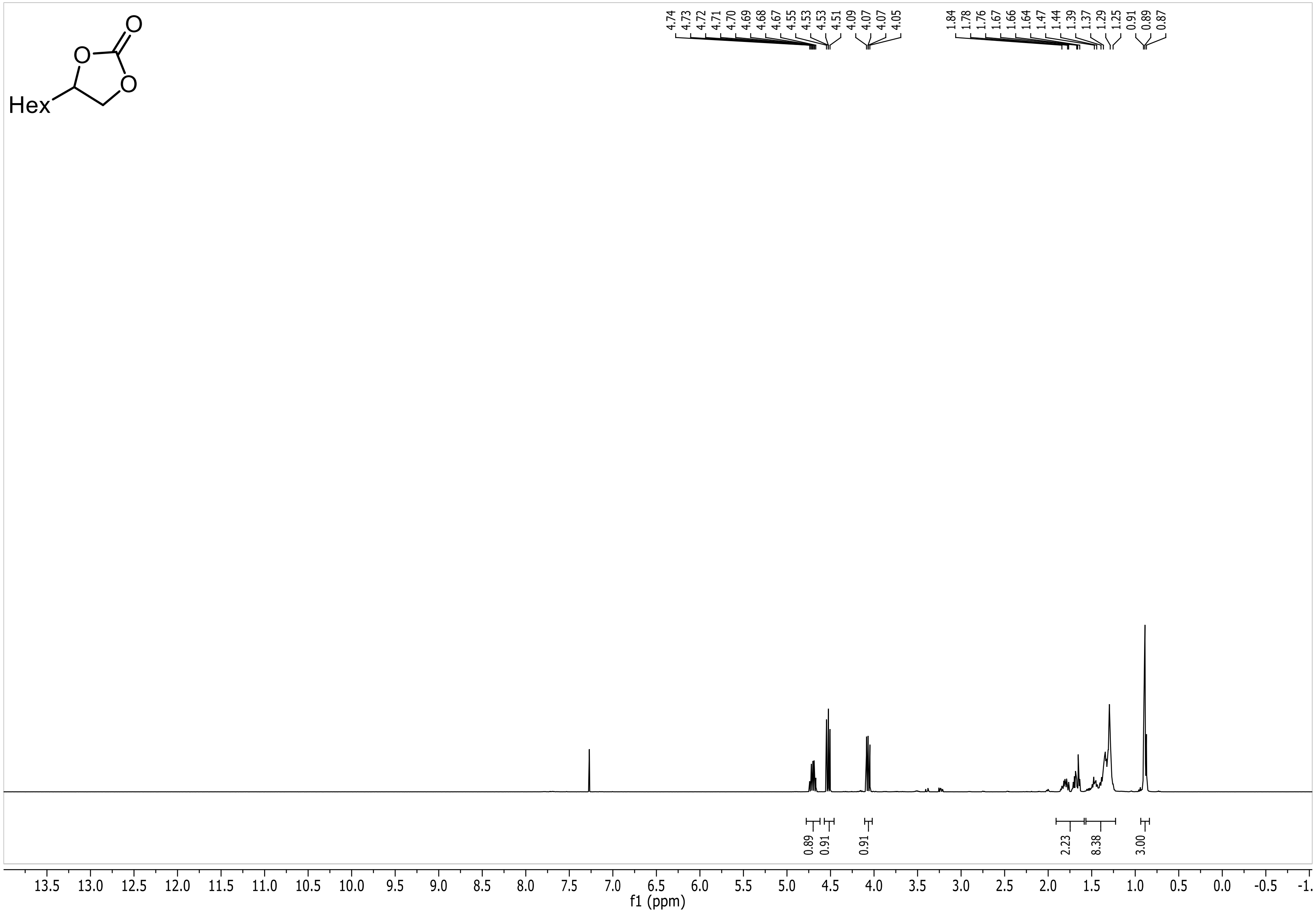

¹H NMR 4-Phenyl-1,3-dioxalan-2-one (2e)

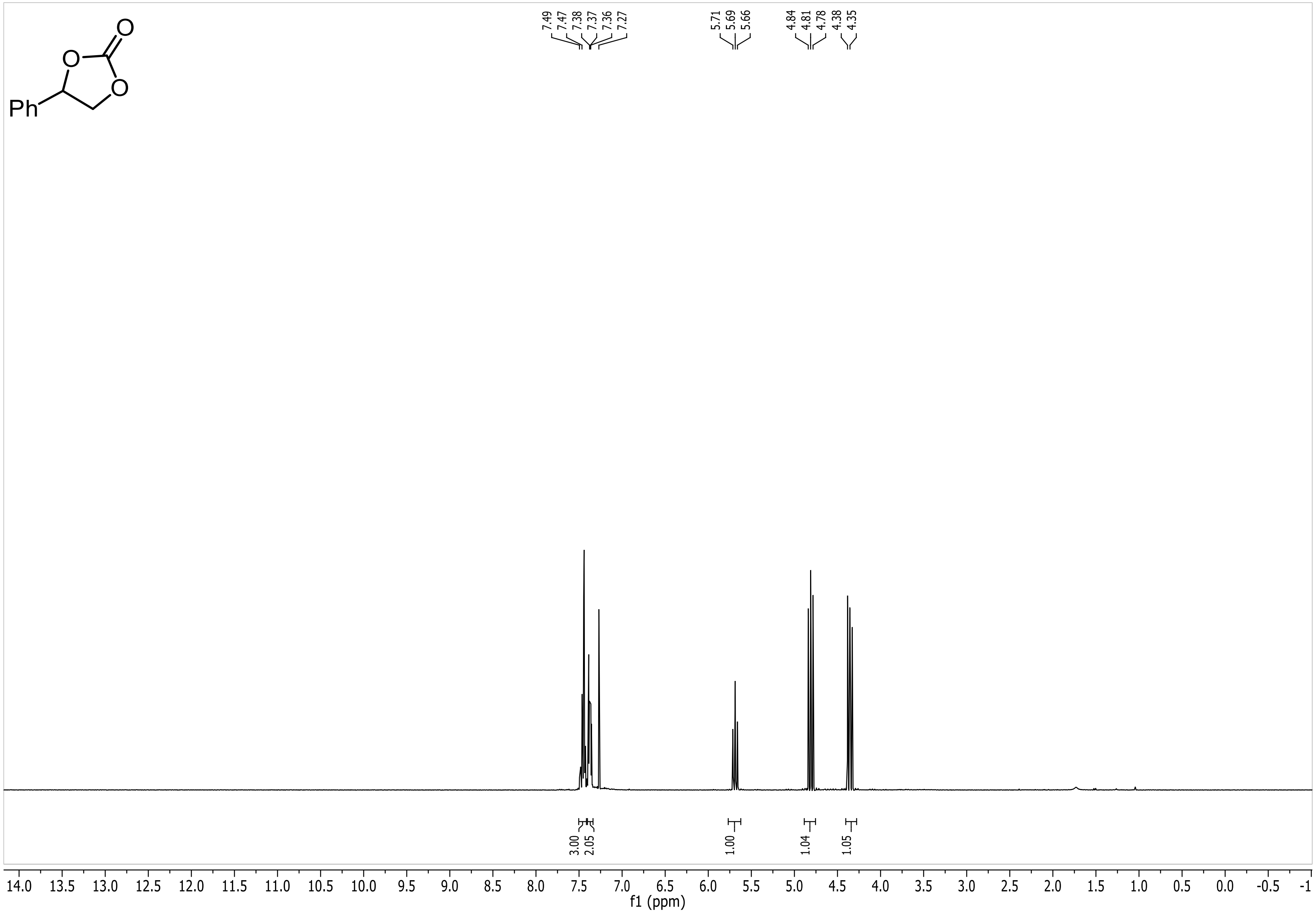

^{1}H NMR 4-(Chloromethyl)-1,3-dioxalan-2-one (2f)

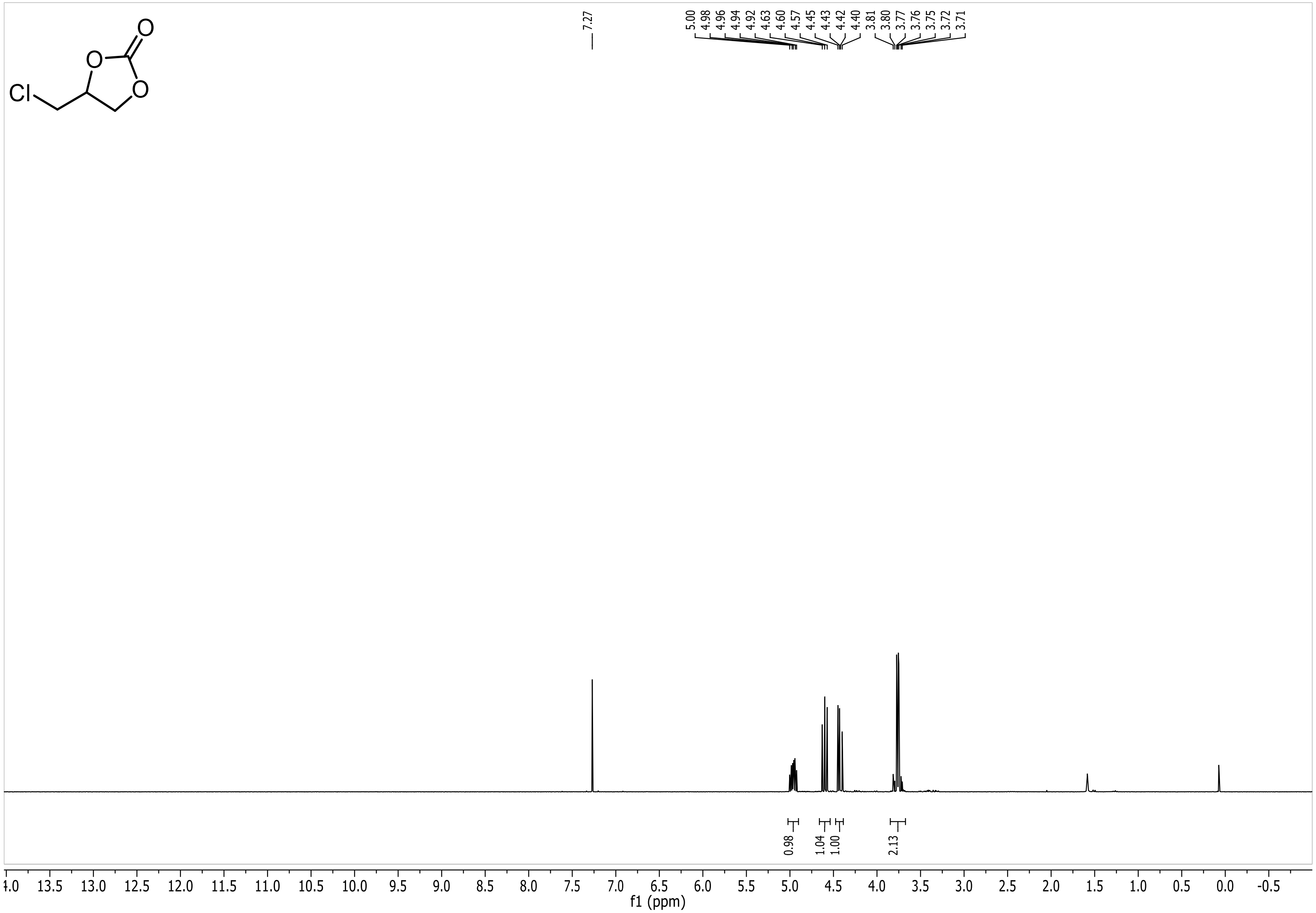

^{1}H NMR 4-(Morpholinomethyl)-1,3-dioxolan-2-one (2g)

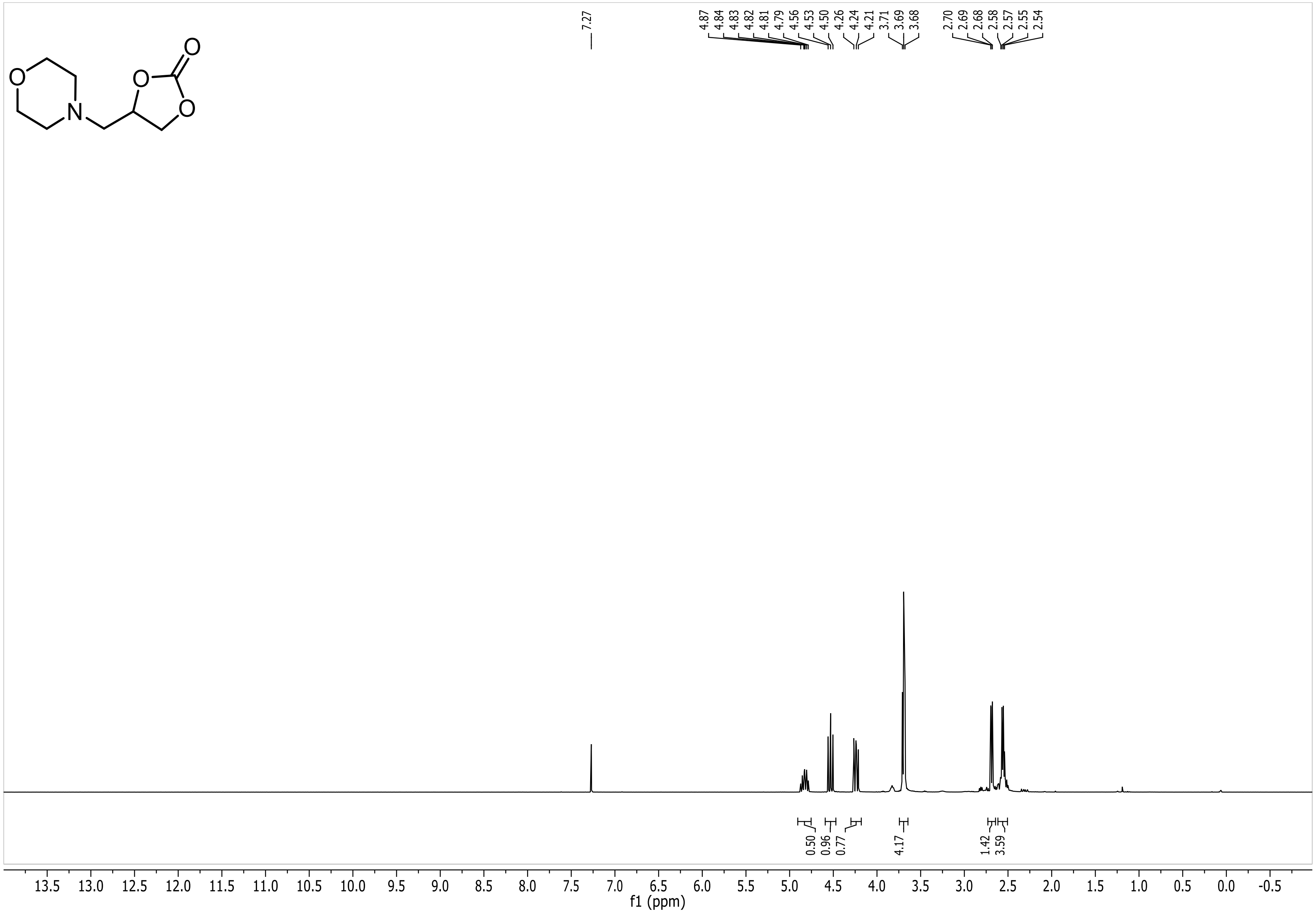

^{1}H NMR 4,4-Dimethyl-1,3-dioxalan-2-one (2h)

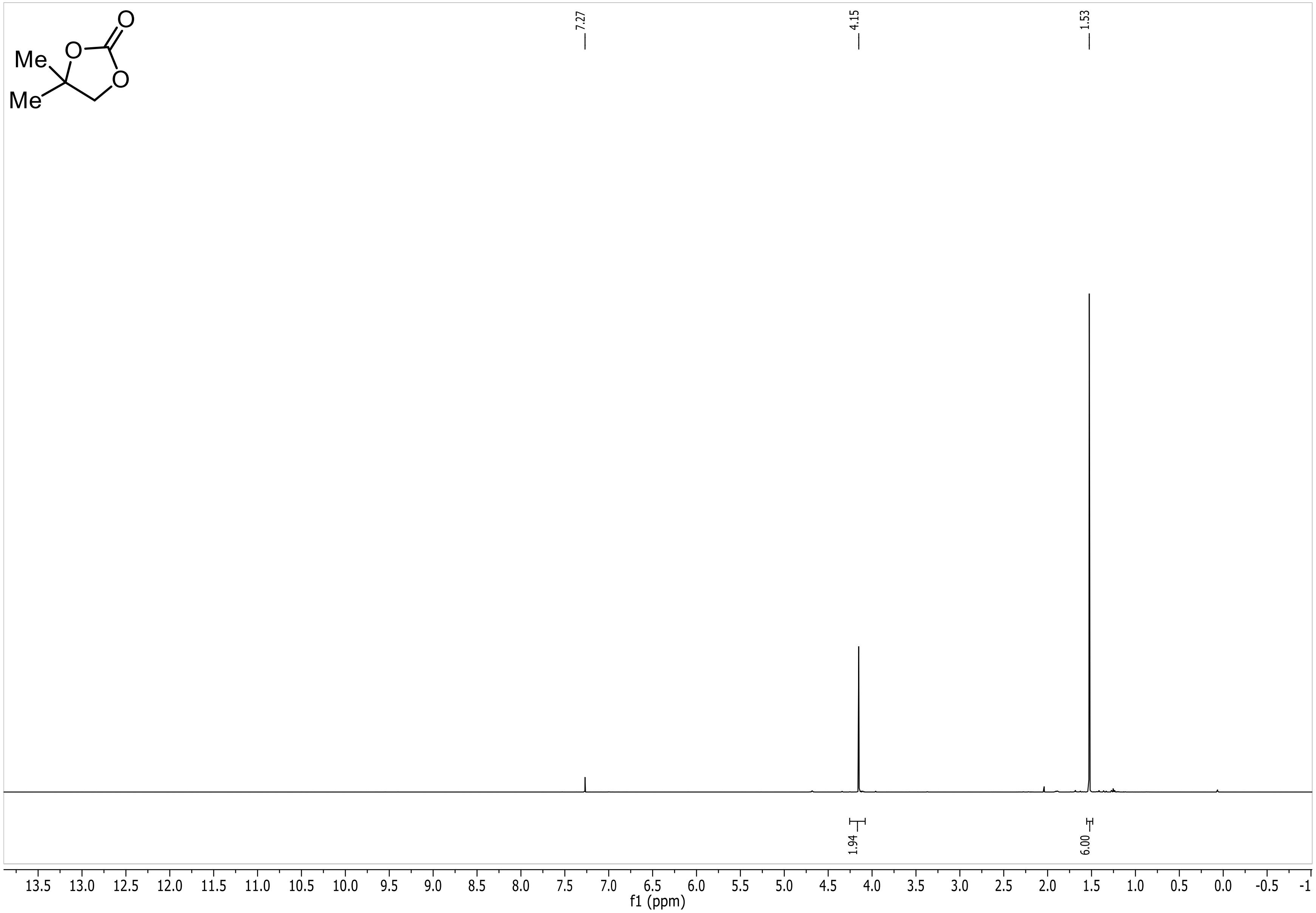

^{1}H NMR 4-(Chloromethyl)-4-methyl-1,3-dioxolan-2-one (2i)

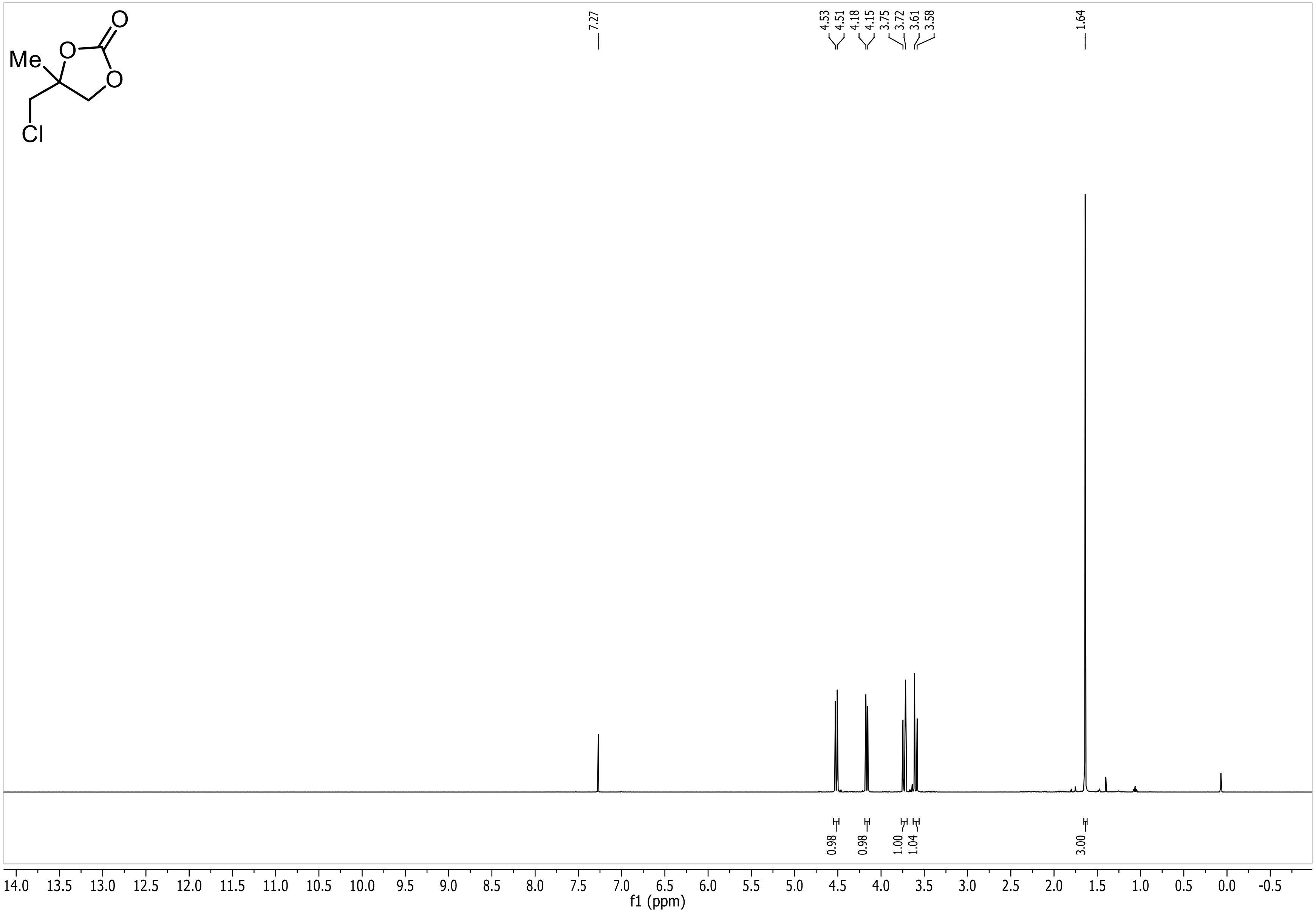

1-(Oxiran-2-ylmethyl)-4-phenylpiperazine (1j)

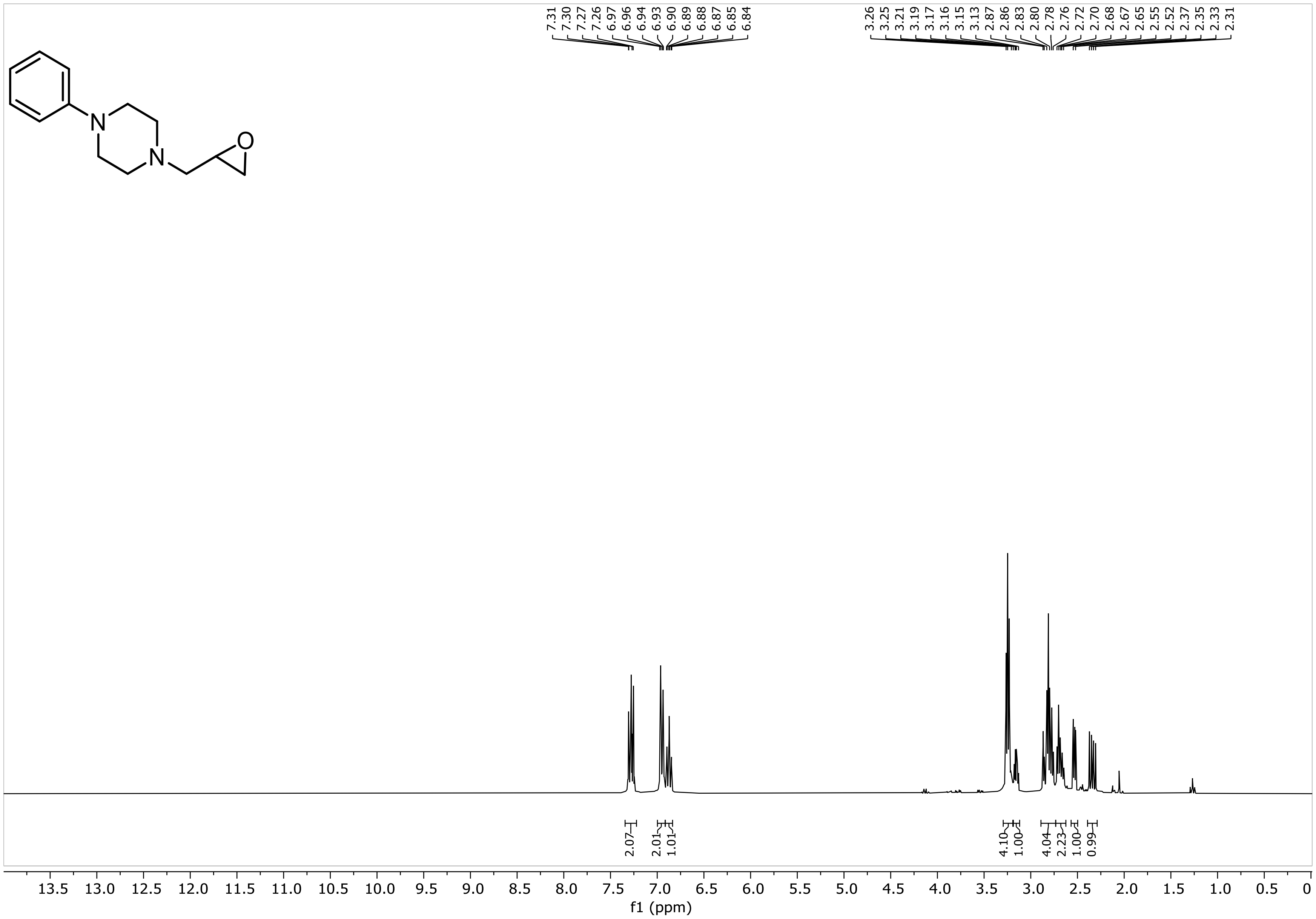

^{1}H NMR 4-((4-Phenylpiperazin-1-yl)methyl)-1,3-dioxolan-2-one (2j)

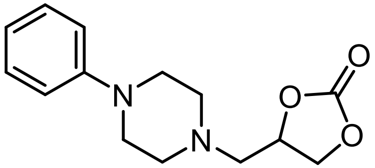

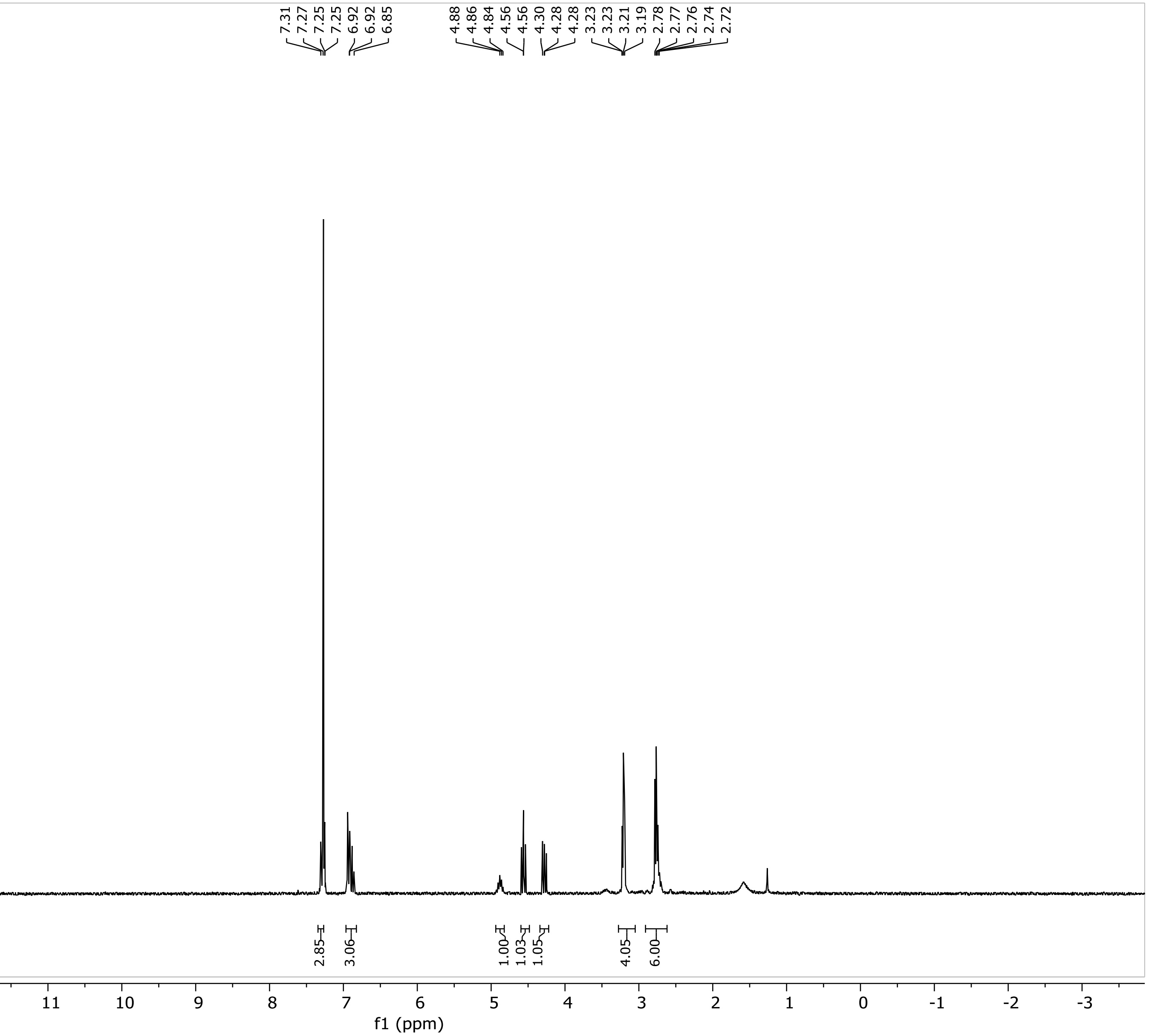

^{13}C NMR 4-((4-Phenylpiperazin-1-yl)methyl)-1,3-dioxolan-2-one (2j)

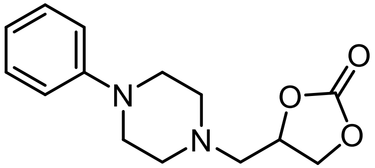

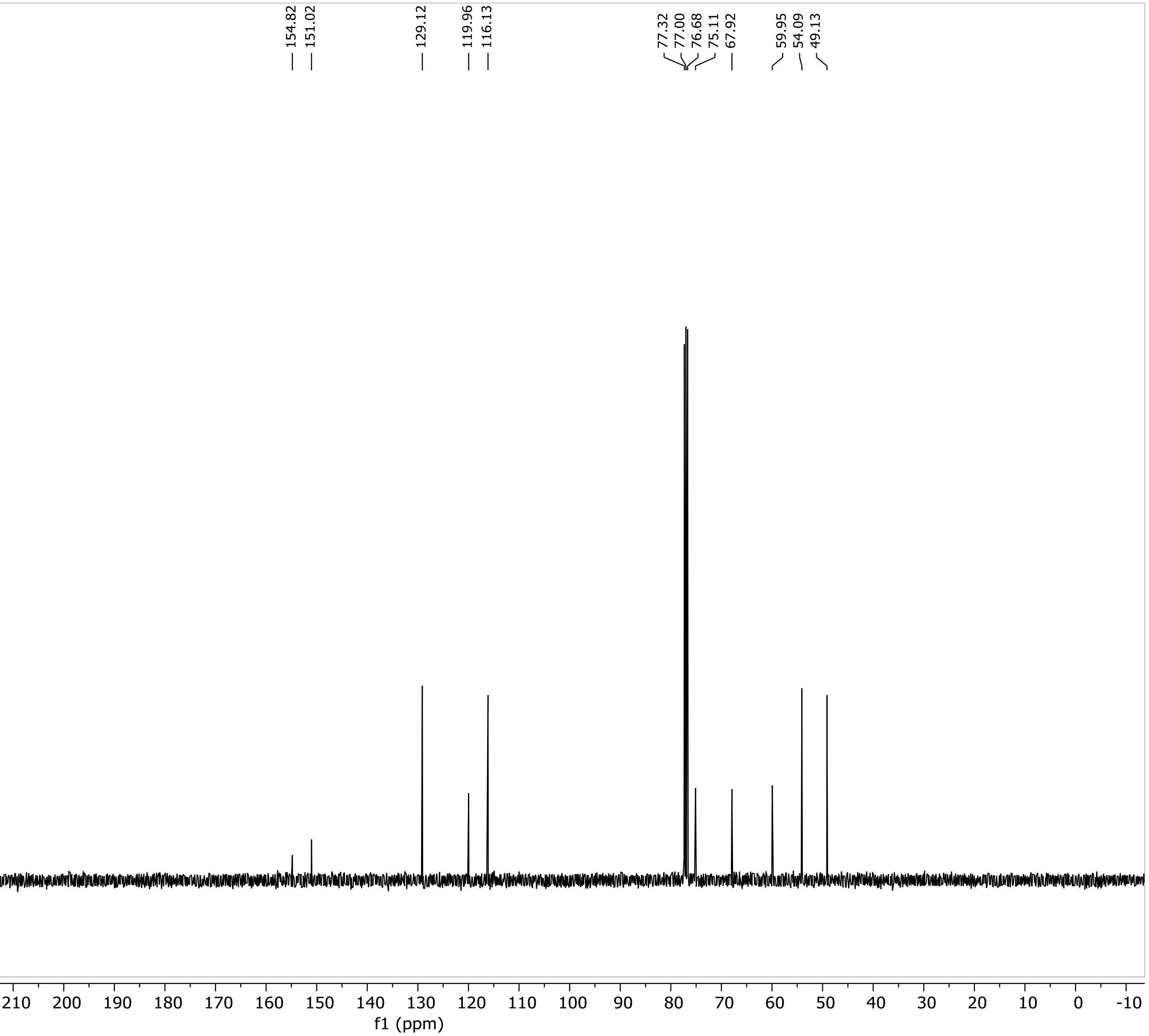

^{1}H NMR 4-(Methoxymethyl)-1,3-dioxalan-2-one (12a)

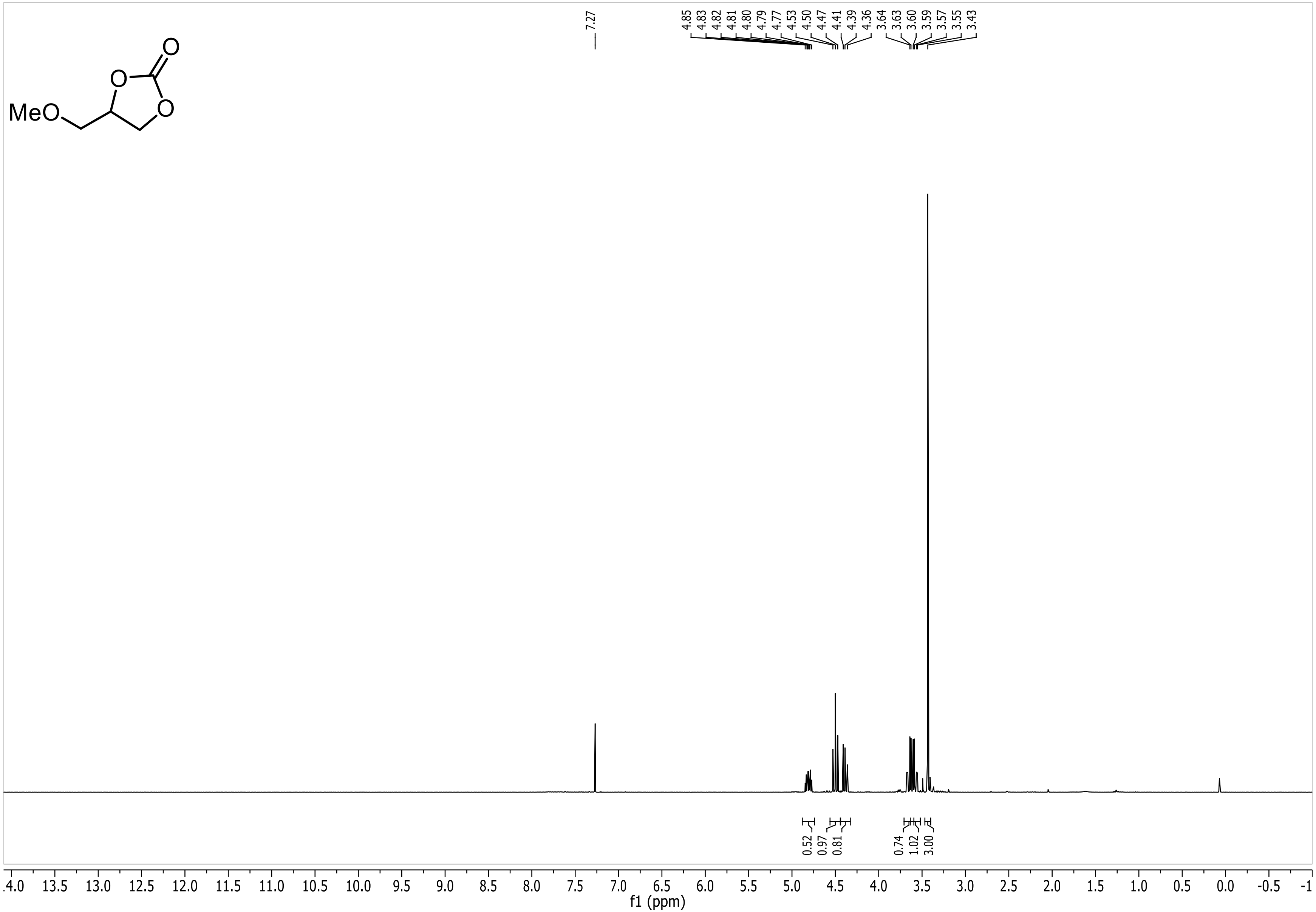

^{1}H NMR 4-(*tert*-Butoxymethyl)-1,2-dioxalan-2-one (12b)

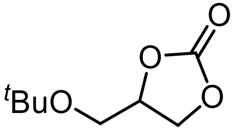

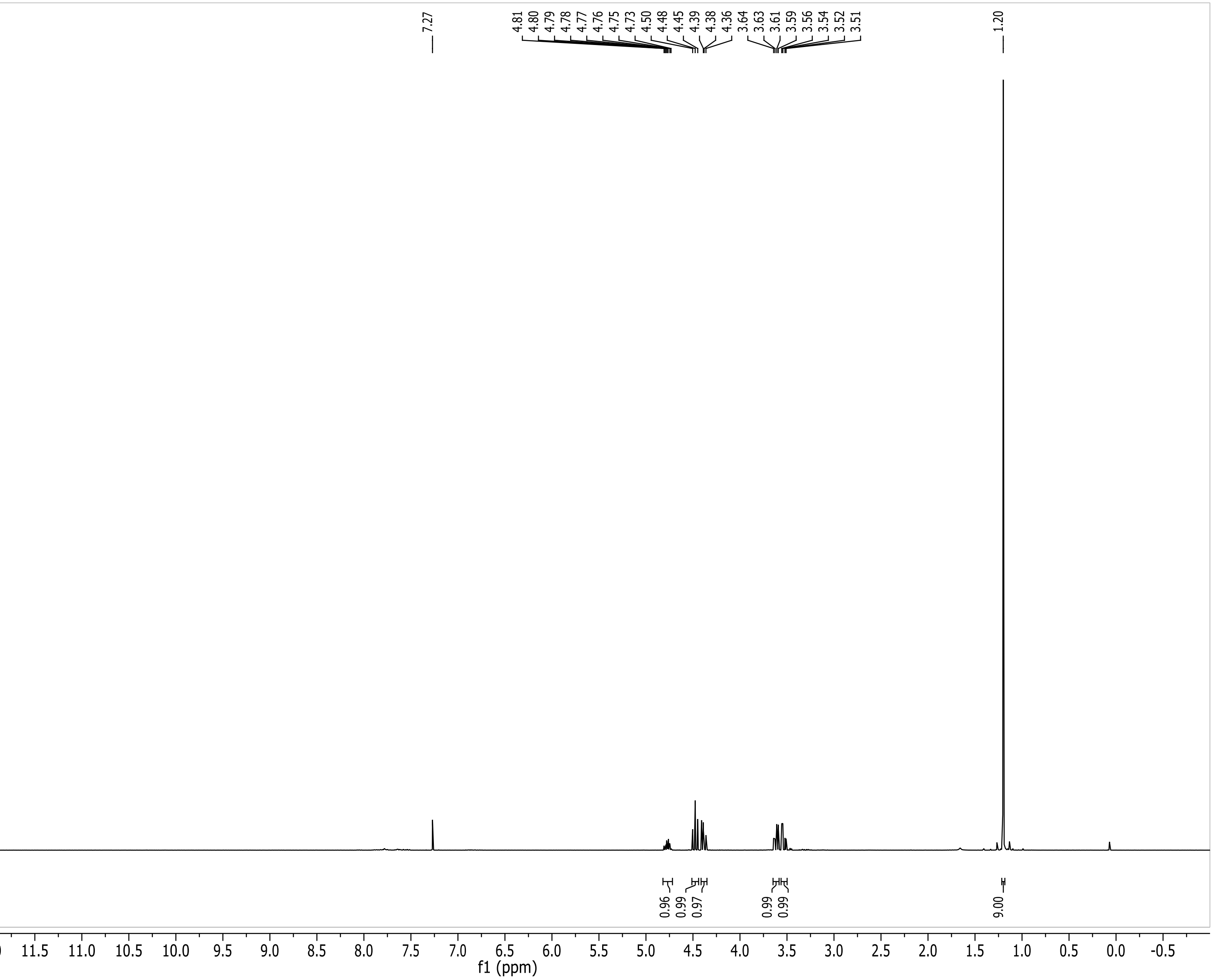

^{1}H NMR 4-(Hydroxymethyl)-1,3-dioxalan-2-one (12c)

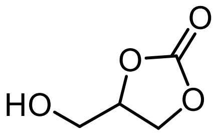

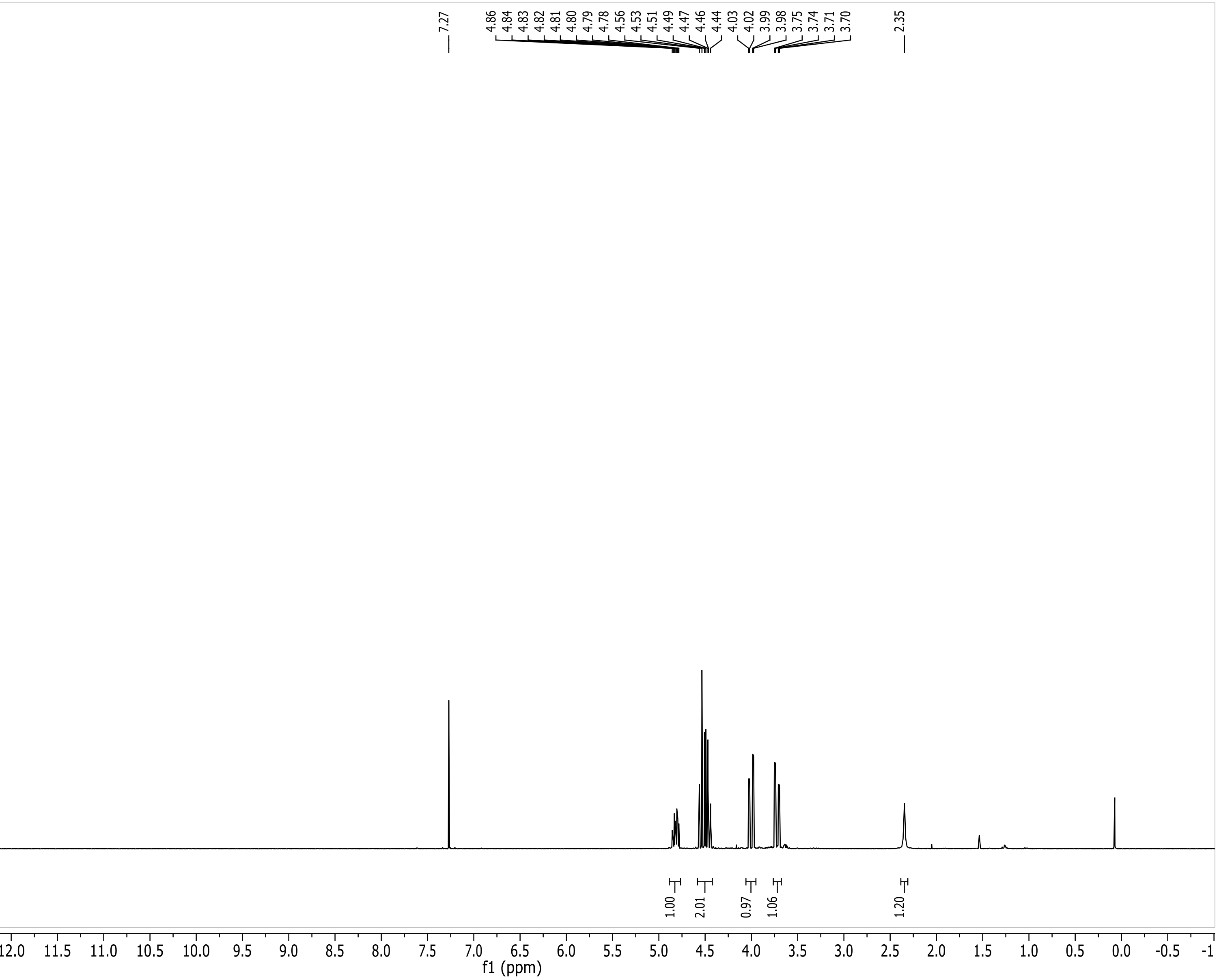

^{1}H NMR 4-((2,2,3,3-Tetraflouropropoxy)methyl-1,3-dioxalan-2-one (12d)

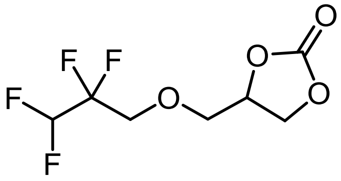

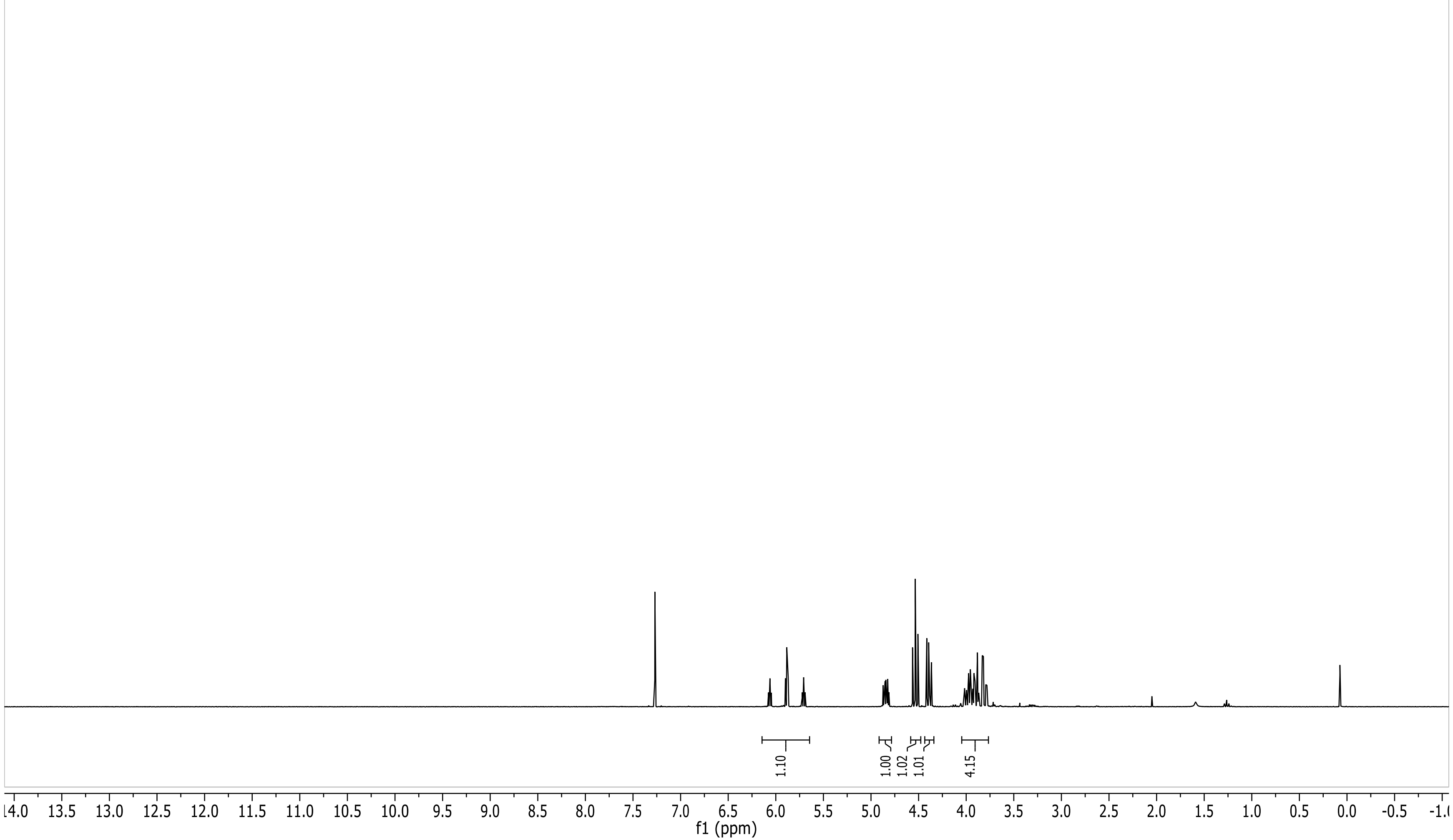

¹H NMR 4-(((2,2,3,3,4,4,5,5-Octafluoropentyl)oxy)methyl)-1,3-dioxalan-2-one (12e)

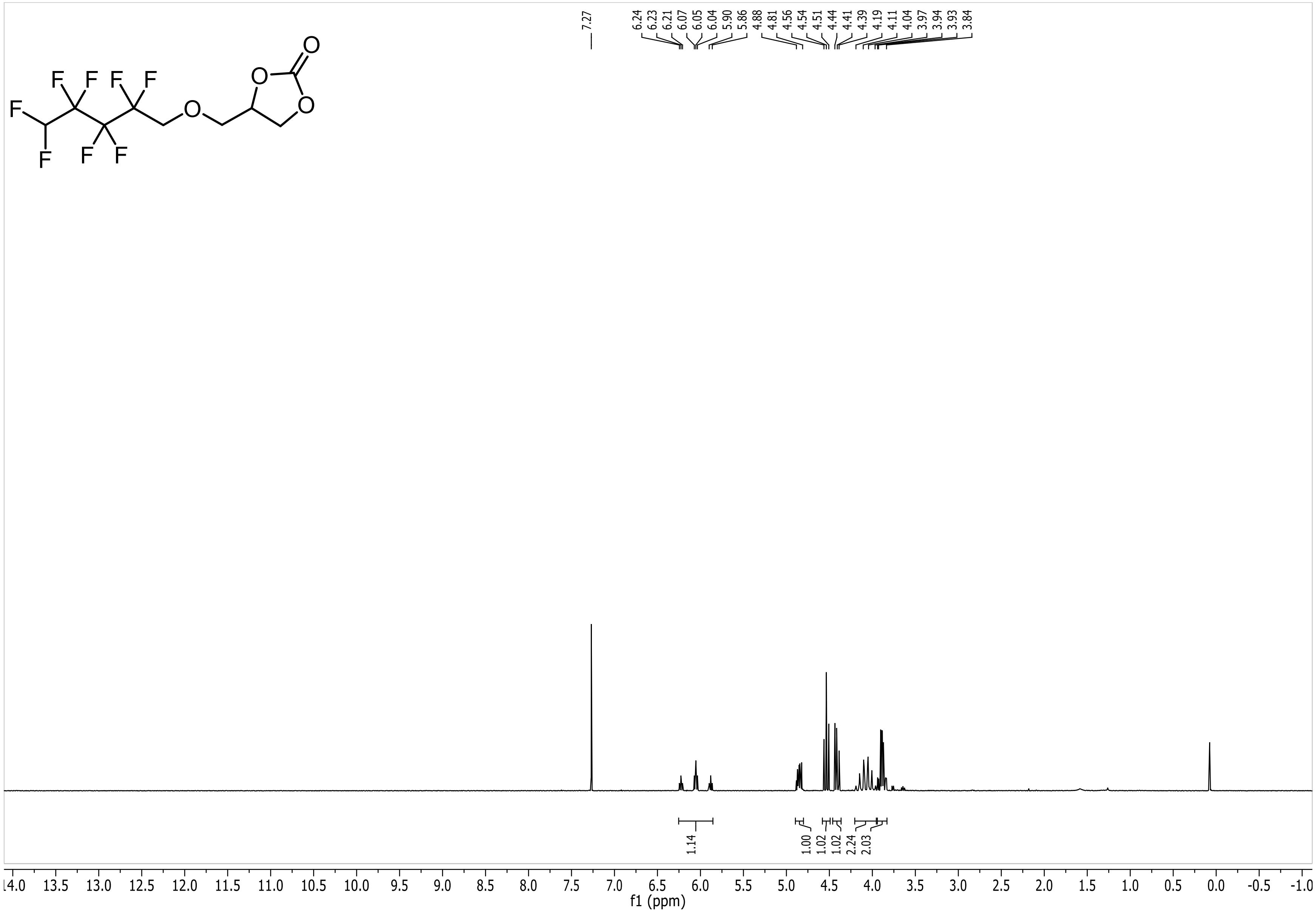

^{1}H NMR 2-(Oxo-1,3-dioxalan-4-yl)methyl methacrylate (12f)

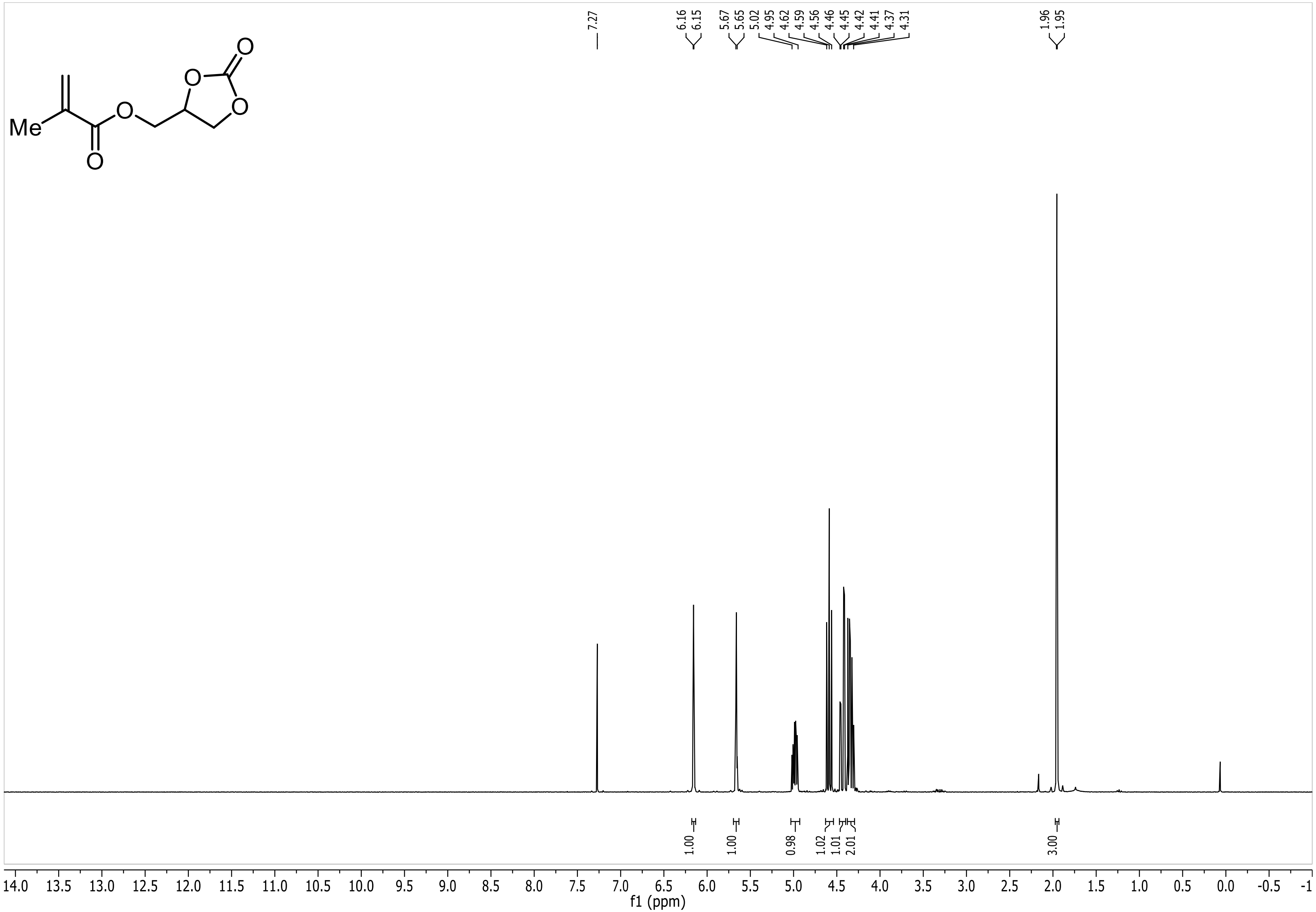

¹H NMR 4-((Allyloxy)methyl)-1,3-dioxolan-2-one (12g)

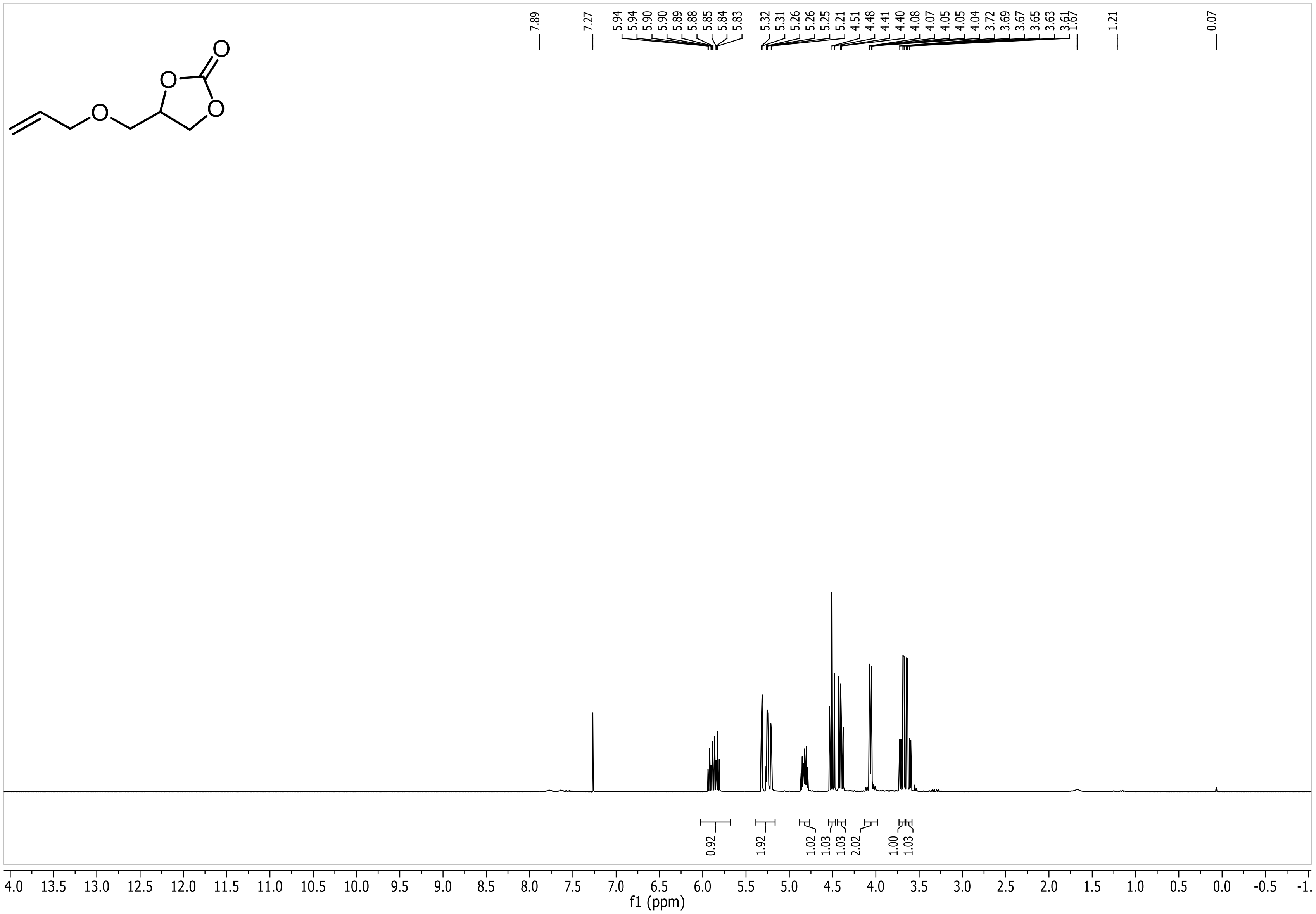

^{1}H NMR 4-((Furan-2-ylmethoxy)methyl)-1,3-dioxolan-2-one (12h)

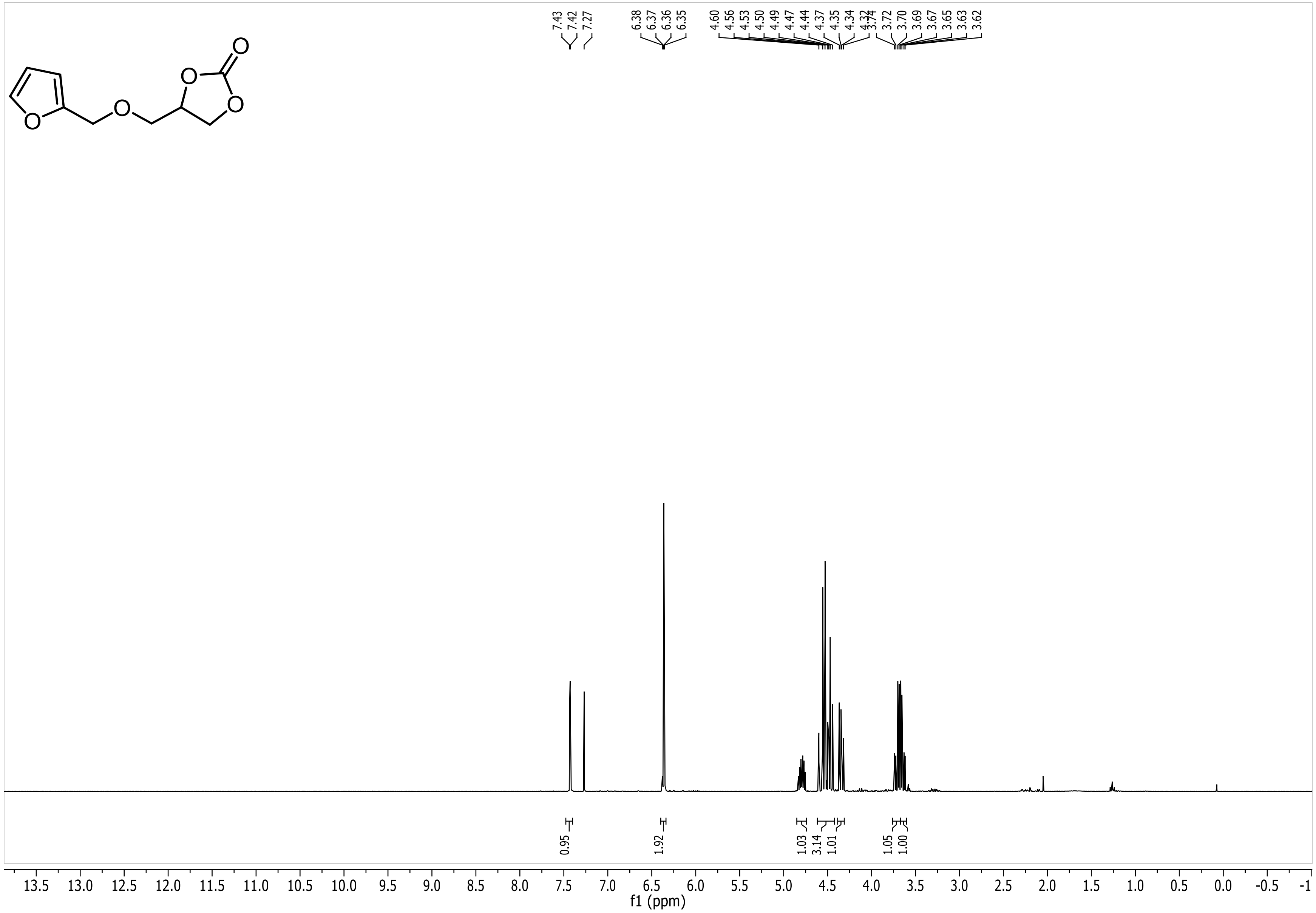

^{1}H NMR 4-((3-(Triethoxysilyl)propoxy)methyl)-1,3-dioxolan-2-one (12i)

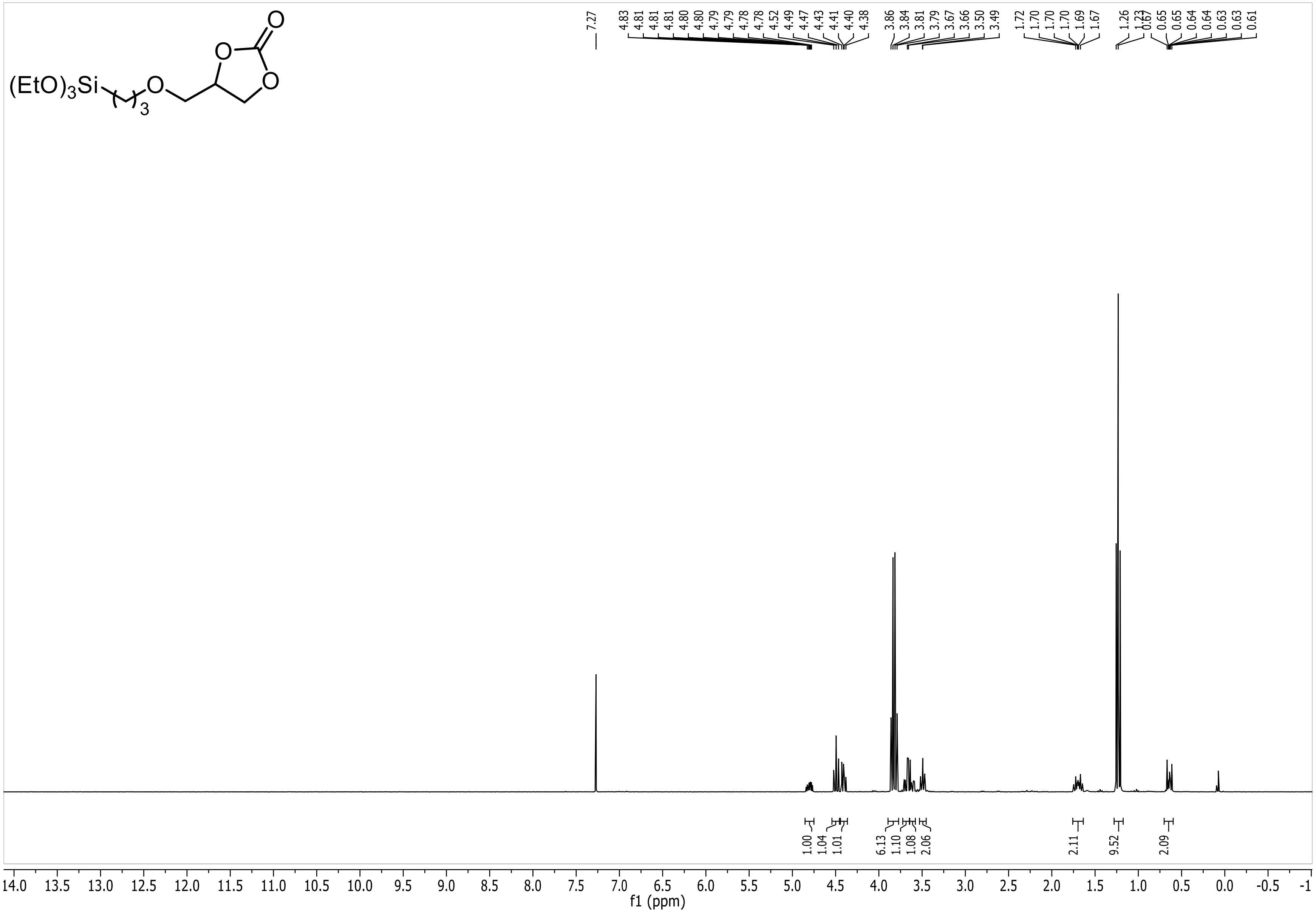

^1H NMR 4,4'-(((Propane-2,2-diylbis(4,1-phenylene))bis(oxy))bis(methylene))bis(1,3-dioxolan-2-one) (12j)

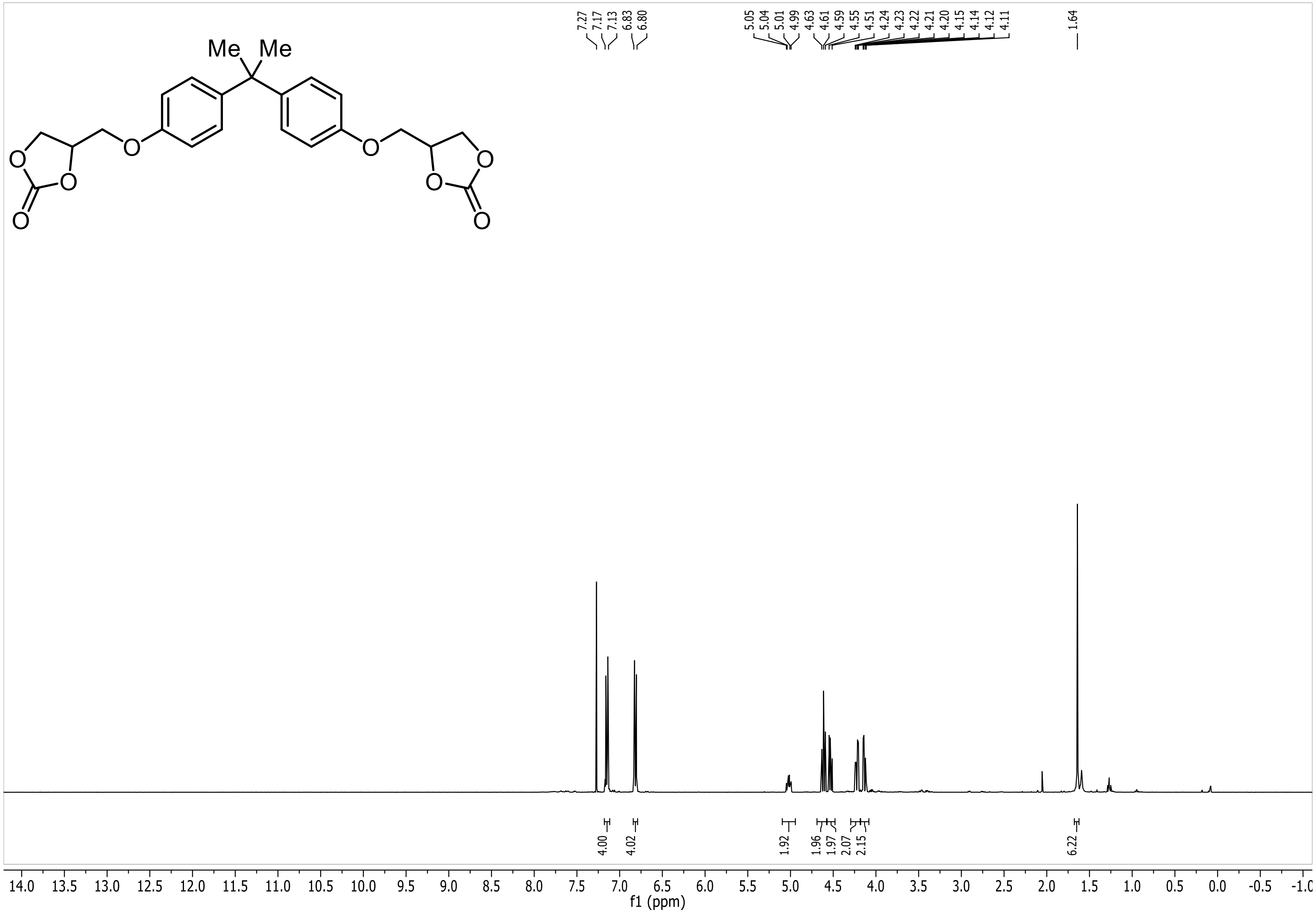

^{1}H NMR Hexahydrobenzo[d][1,3]dioxol-2-one (14a)

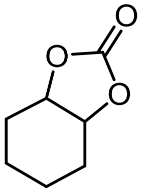

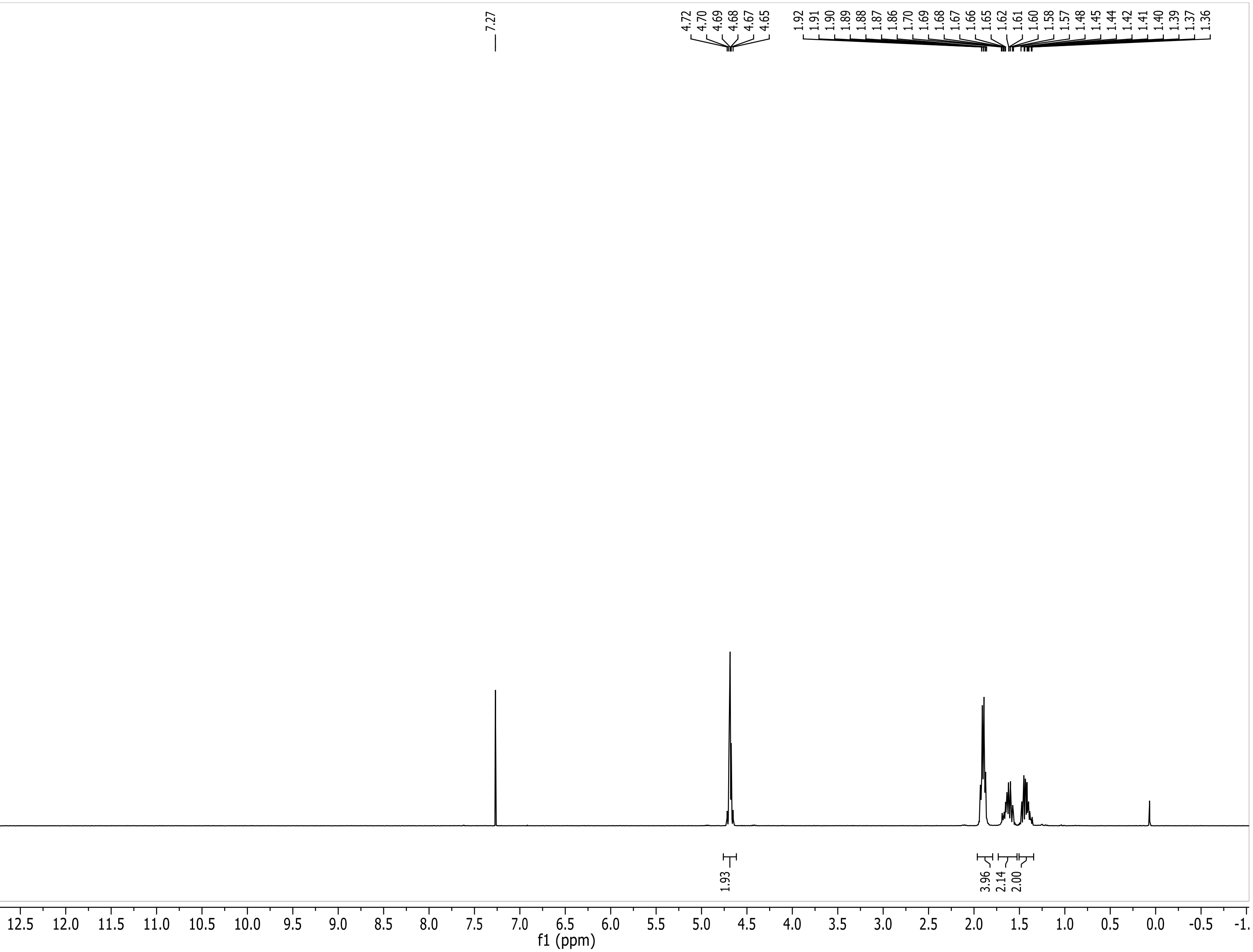

¹H NMR Tetrahydro-4H-cyclopenta[d][1,3]dioxol-2-one (14b)

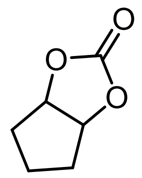

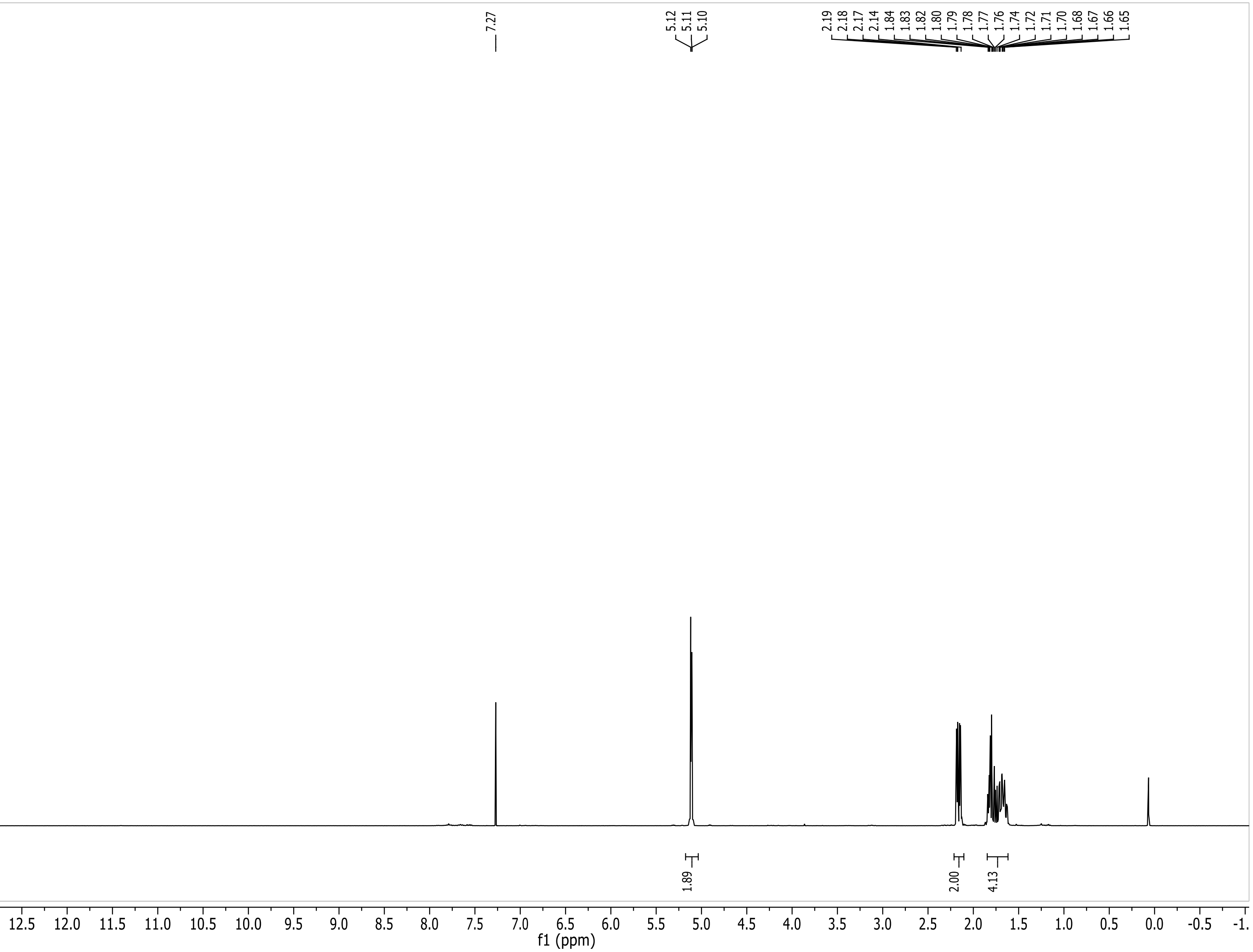

^{1}H NMR Tetrahydrofuro[3,4-d][1,3]dioxol-2-one (14c)

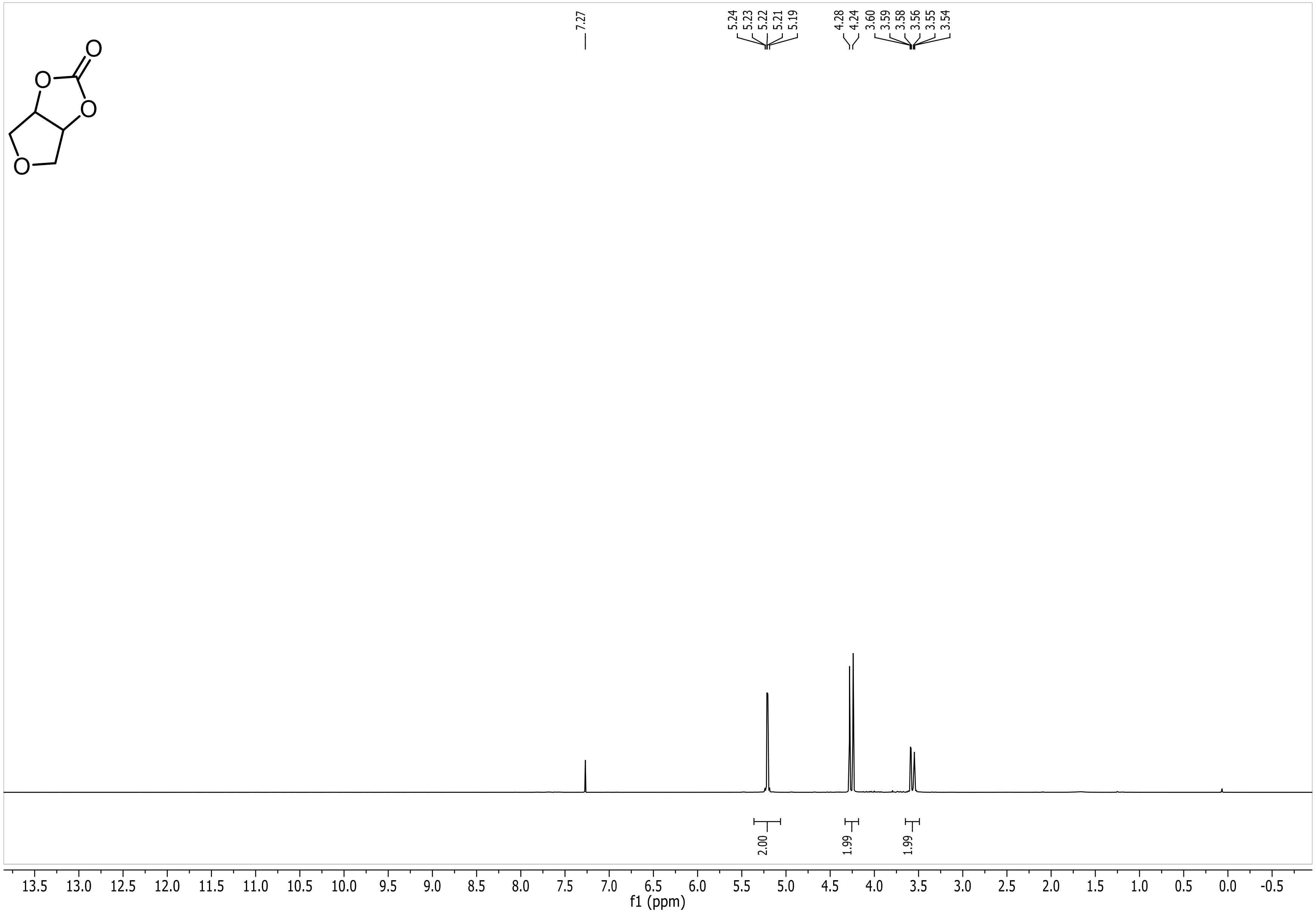

^{1}H NMR 4,5-Diphenyl-1,3-dioxalan-2-one (14d)

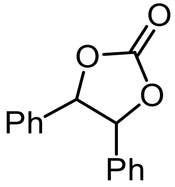

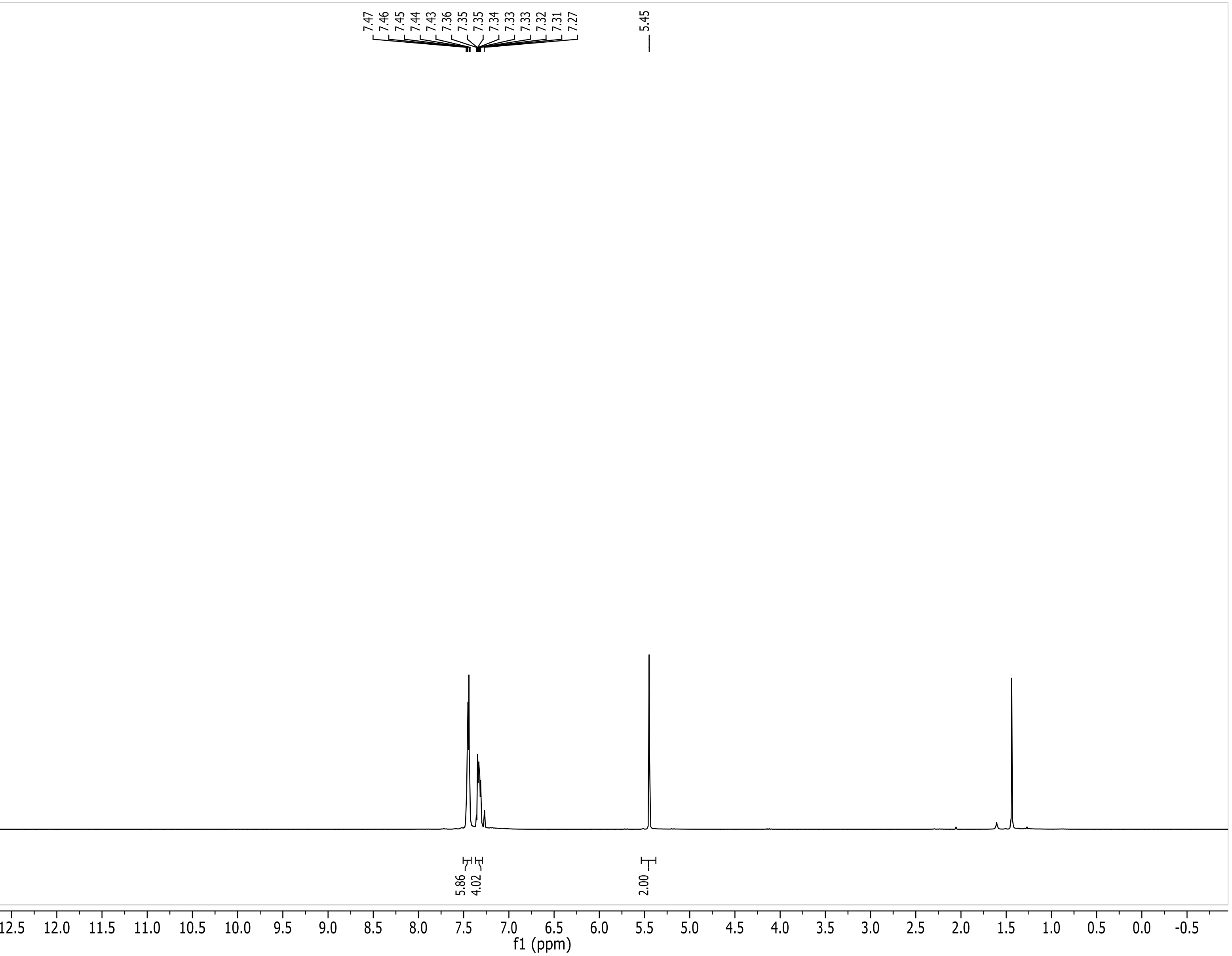

¹H NMR 4,5-Dimethyl-1,3-dioxalan-2-one (14e)

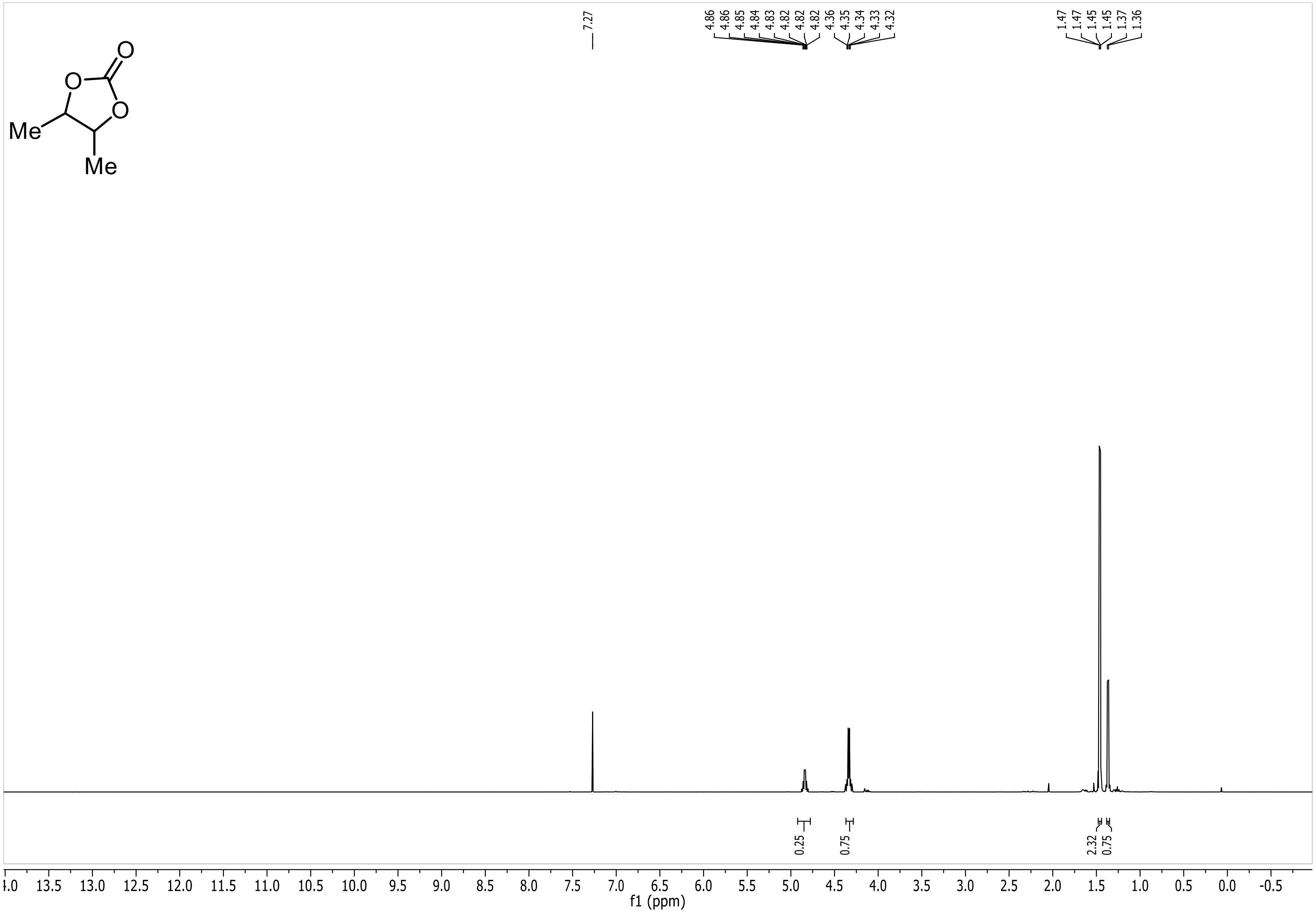

¹H NMR Methyl 8-(5-octyl-2-oxo-1,3-dioxalan-4-yl)octanoate (14f)

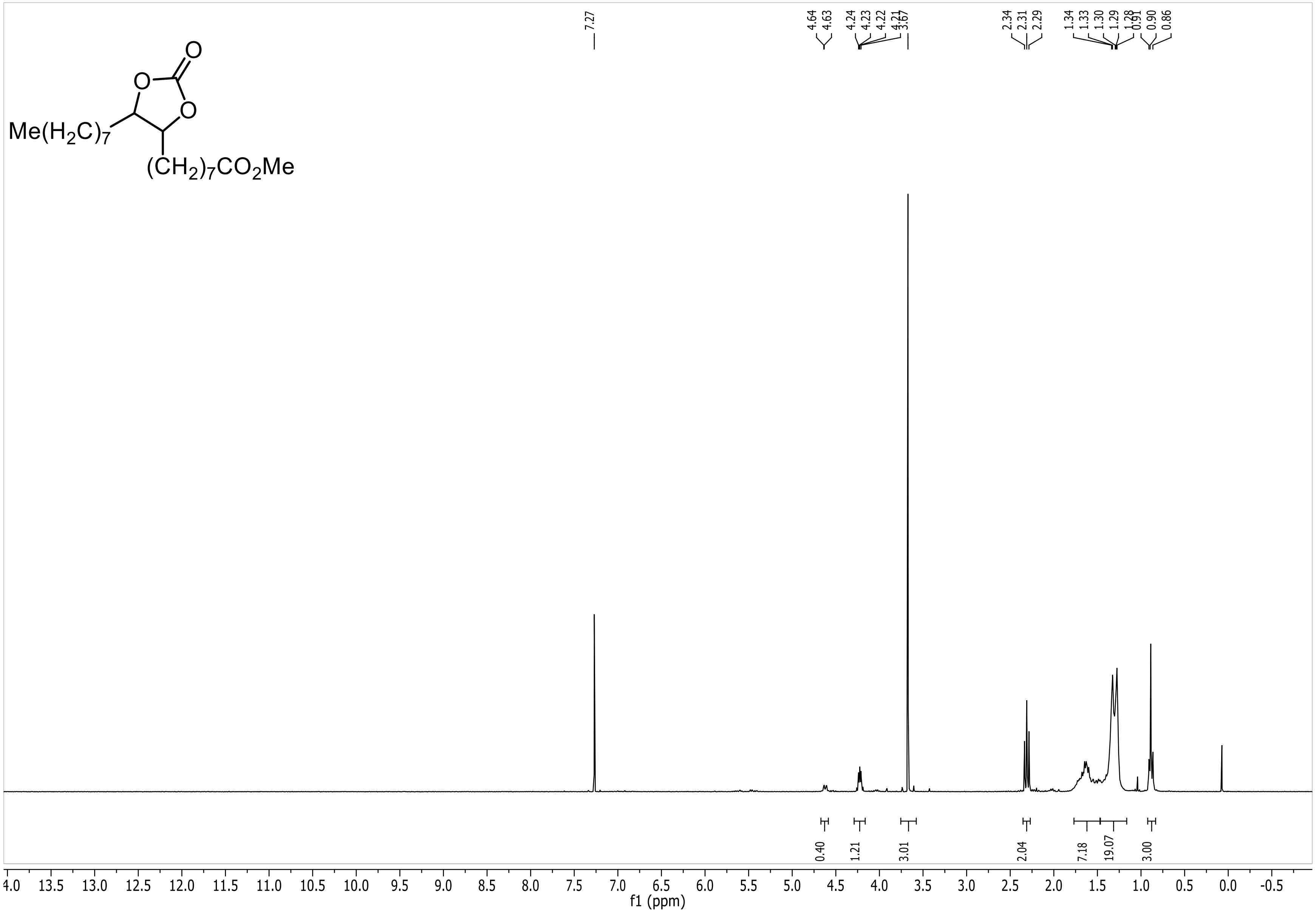

13 HPLC-spectra of chiral carbonates

4-Ethyl-1,3-dioxalan-2-one (*rac*-2b) [6] [6] [6] [6] [6] [6] [6] [6] [6] [6] [6]

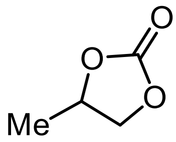

Additional Info : Peak(s) manually integrated

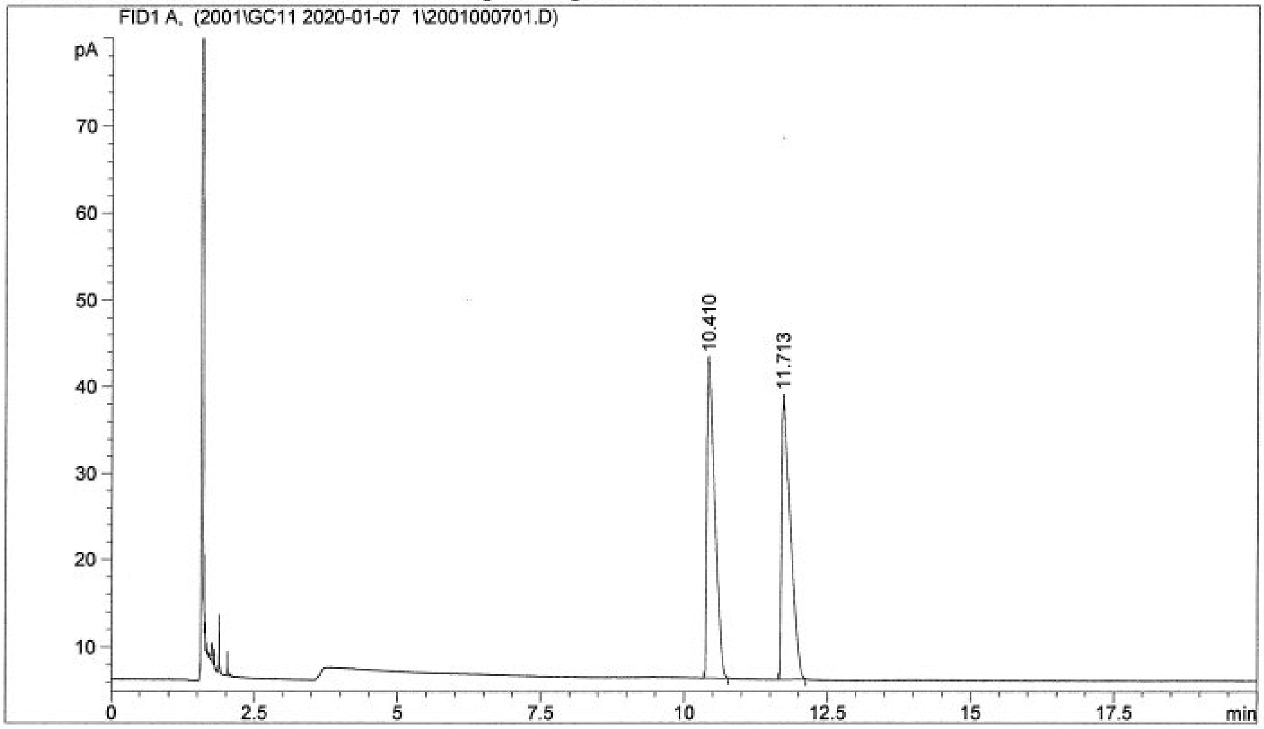

===
Area Percent Report
===

Sorted By : Signal
Multiplier : 1.0000
Dilution : 1.0000
Use Multiplier & Dilution Factor with ISTDs

Signal 1: FID1 A,

Peak #	RetTime [min]	Type	Width [min]	Area [pA*s]	Height [pA]	Area %
1	10.410	BB	0.1189	343.20883	36.89164	50.01613
2	11.713	BB	0.1250	342.98749	32.63432	49.98387
Totals :				686.19632	69.52596	

(*S*)-4-Ethyl-1,3-dioxalan-2-one (*S*-2b) [6] [6] [6] [6] [6] [6] [6] [6] [6] [6] [6]

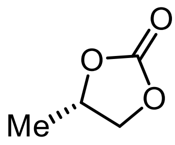

Additional Info : Peak(s) manually integrated

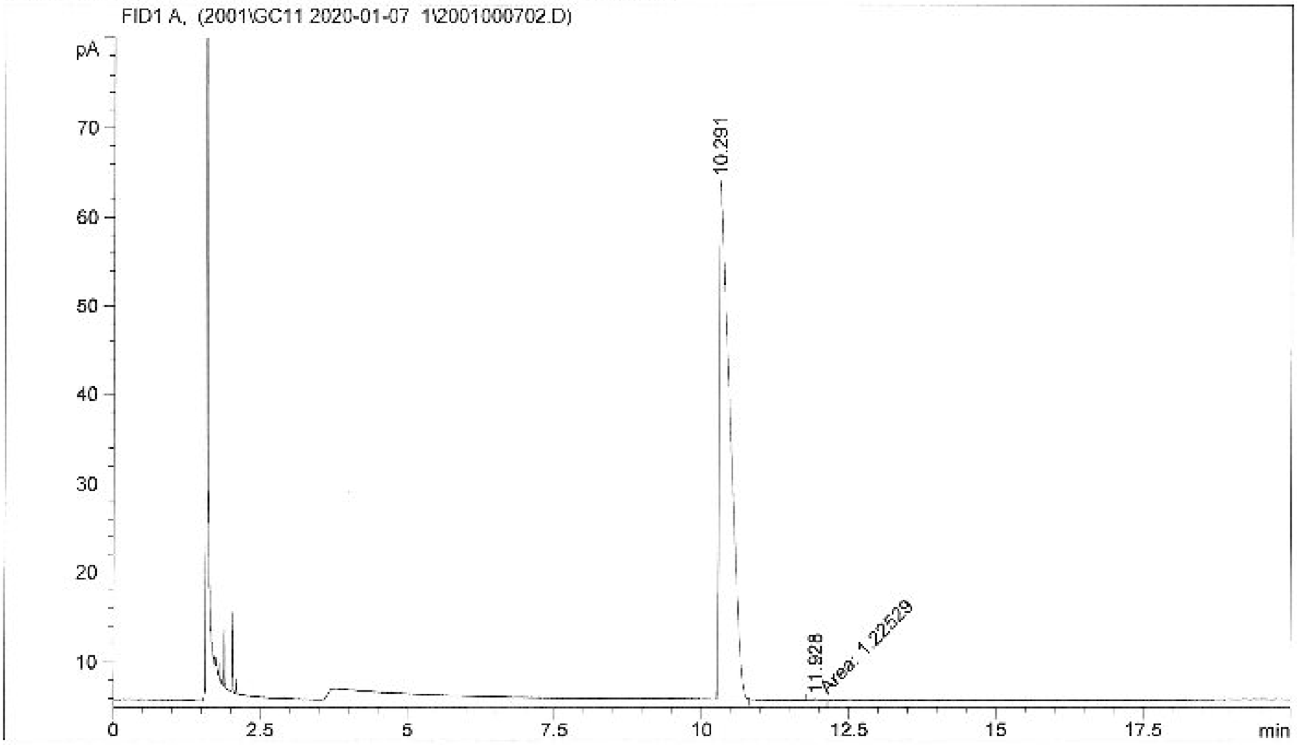

===
Area Percent Report
===

Sorted By : Signal
Multiplier : 1.0000
Dilution : 1.0000
Use Multiplier & Dilution Factor with ISTDs

Signal 1: FID1 A,

Peak #	RetTime [min]	Type	Width [min]	Area [pA*s]	Height [pA]	Area %
1	10.291	BB	0.1522	729.84650	58.09904	99.83240
2	11.928	MM	0.1095	1.22529	1.86554e-1	0.16760

Totals : 731.07178 58.28559

/ 99,82 %
/ 0,18 %

4-Phenyl-1,3-dioxalan-2-one (*rac*-2e) [6] [6] [6] [6] [6] [6] [6] [6] [6] [6] [6]

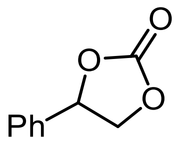

Additional Info : Peak(s) manually integrated

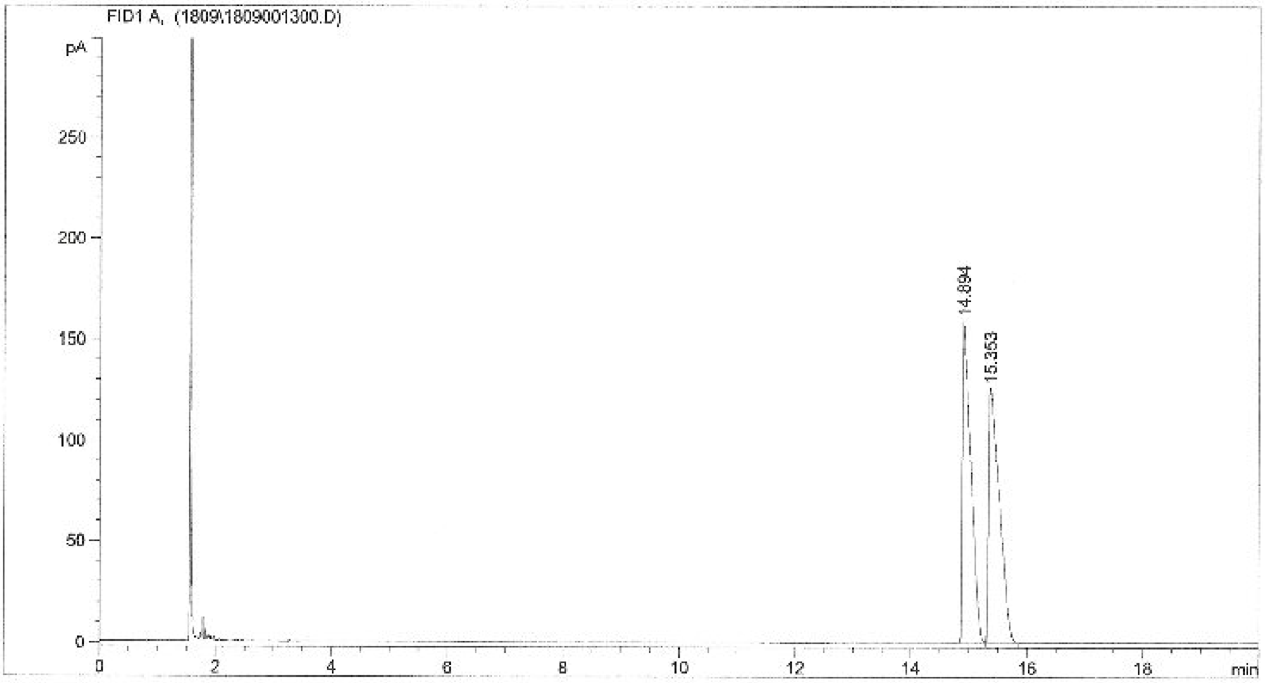

===

Area Percent Report

===

Sorted By : Signal
Multiplier : 1.0000
Dilution : 1.0000
Use Multiplier & Dilution Factor with ISTDs

Signal 1: FID1 A,

Peak #	RetTime [min]	Type	Width [min]	Area [pA*s]	Height [pA]	Area %
1	14.894	BV	0.1248	1589.96106	158.84991	49.93489
2	15.353	VB	0.1554	1594.10742	125.51729	50.06511

Totals : 3184.06848 284.36720

(*R*)-4-Phenyl-1,3-dioxalan-2-one (*R*-2e) [6] [6] [6] [6] [6] [6] [6] [6] [6] [6] [6]

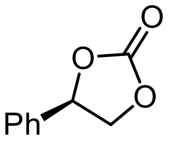

Additional Info : Peak(s) manually integrated

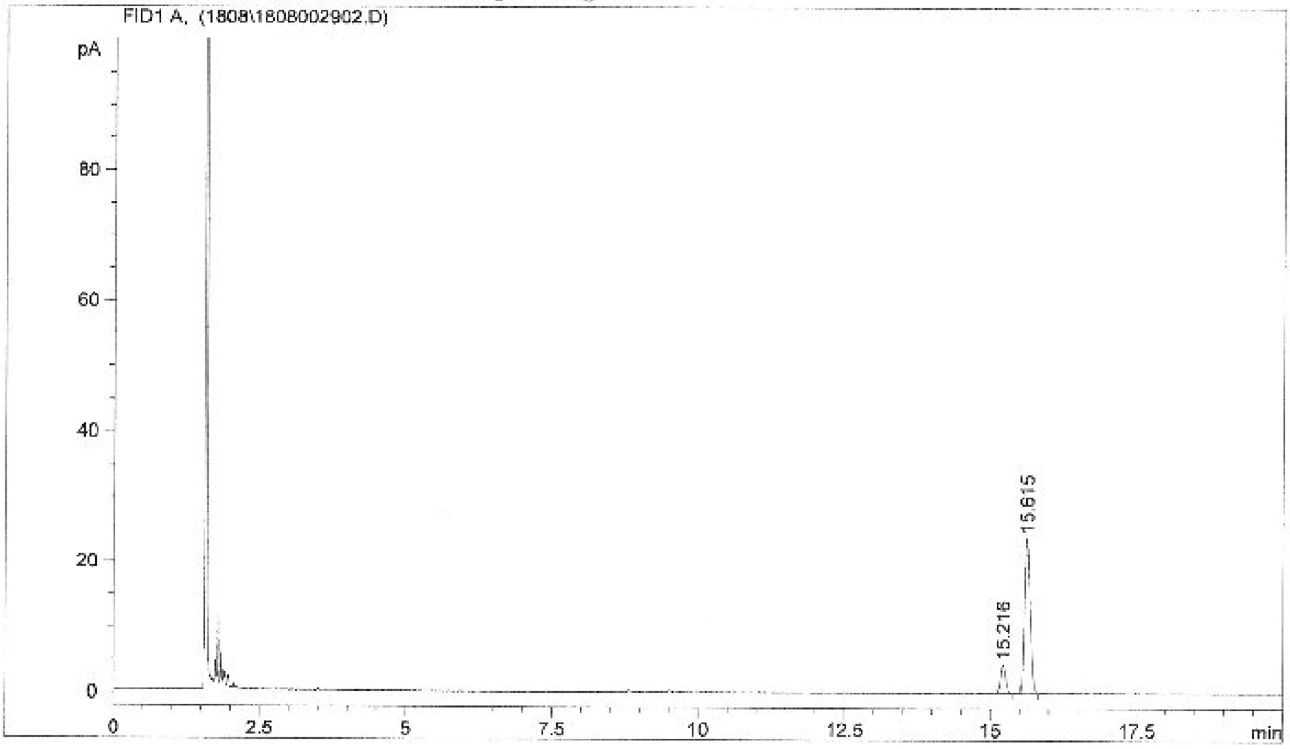

===
Area Percent Report
===

Sorted By : Signal
Multiplier : 1.0000
Dilution : 1.0000
Use Multiplier & Dilution Factor with ISTDs

Signal 1: FID1 A,

Peak #	RetTime [min]	Type	Width [min]	Area [pA*s]	Height [pA]	Area %
1	15.216	BB	0.0776	23.92527	4.15476	13.75155
2	15.615	BB	0.0759	150.05710	23.55858	86.24845

Totals : 173.98237 27.71334

/ 13.73 %
/ 86,27 %

1-(Oxiran-2-ylmethyl)-4-phenylpiperazine (*rac*-1j)

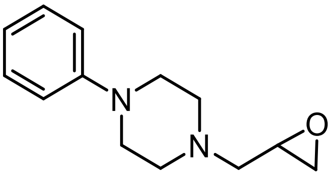

Additional Info : Peak(s) manually integrated

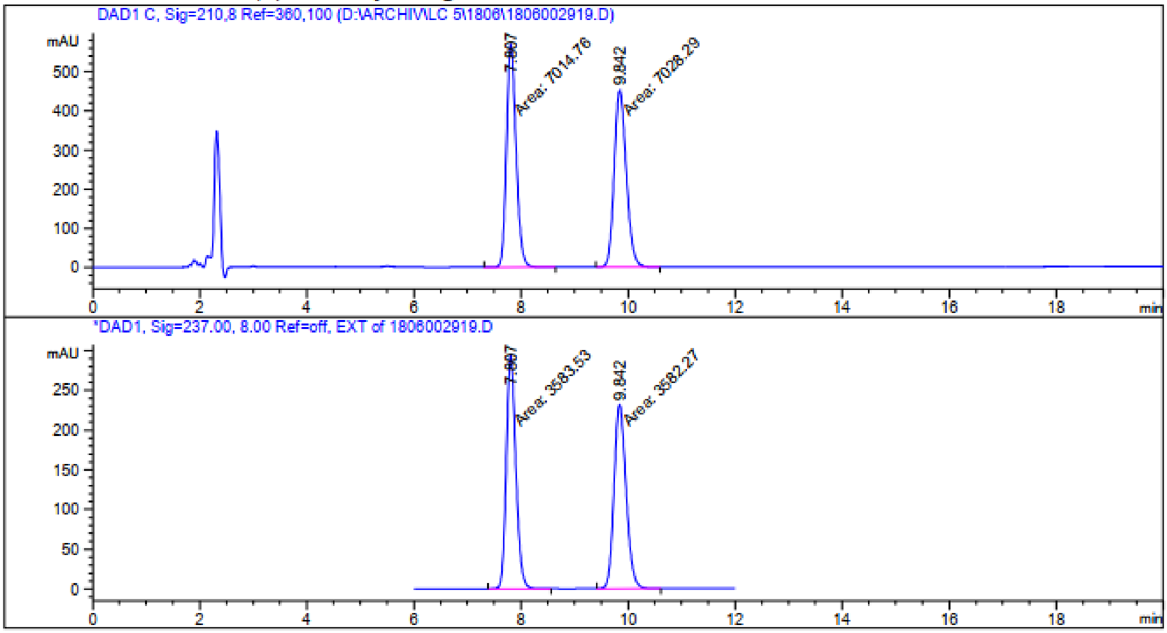

===
Area Percent Report
===

Sorted By : Signal
Multiplier : 1.0000
Dilution : 1.0000
Use Multiplier & Dilution Factor with ISTDs

Signal 1: DAD1 C, Sig=210,8 Ref=360,100

Peak #	RetTime [min]	Type	Width [min]	Area [mAU*s]	Height [mAU]	Area %
1	7.807	MM	0.2051	7014.76367	569.93549	49.9519
2	9.842	MM	0.2591	7028.28662	452.14575	50.0481

Totals : 1.40431e4 1022.08124

Signal 2: DAD1, Sig=237.00, 8.00 Ref=off, EXT
Signal has been modified after loading from rawdata file!

Peak #	RetTime [min]	Type	Width [min]	Area [mAU*s]	Height [mAU]	Area %
1	7.807	MM	0.2037	3583.53198	293.14435	50.0088
2	9.842	MM	0.2578	3582.27051	231.56297	49.9912

Totals : 7165.80249 524.70732

(*R*)-(Oxiran-2-ylmethyl)-4-phenylpiperazine (*R*-1j)

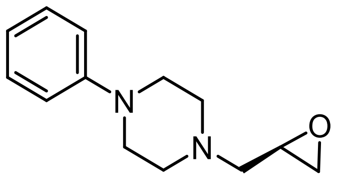

Additional Info : Peak(s) manually integrated

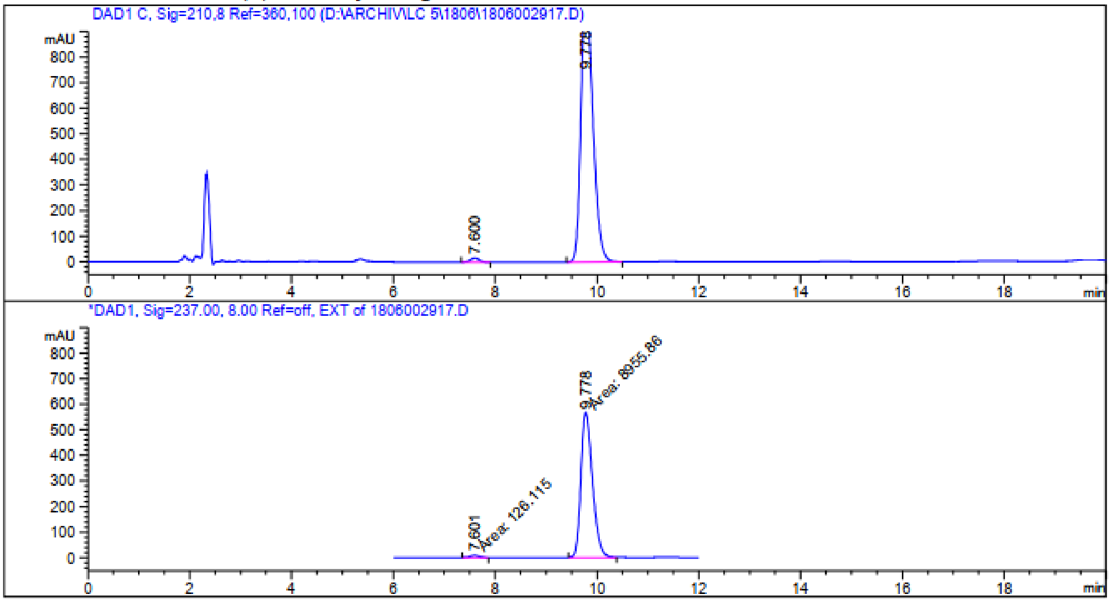

===

Area Percent Report

===

Sorted By : Signal
Multiplier : 1.0000
Dilution : 1.0000
Use Multiplier & Dilution Factor with ISTDs

Signal 1: DAD1 C, Sig=210,8 Ref=360,100

Peak #	RetTime [min]	Type	Width [min]	Area [mAU*s]	Height [mAU]	Area %
1	7.600	BB	0.1827	200.61403	16.87339	1.1563
2	9.778	BB	0.2472	1.71486e4	1068.51172	98.8437
Totals :				1.73492e4	1085.38511	

Signal 2: DAD1, Sig=237.00, 8.00 Ref=off, EXT
Signal has been modified after loading from rawdata file!

Peak #	RetTime [min]	Type	Width [min]	Area [mAU*s]	Height [mAU]	Area %
1	7.601	MM	0.2254	126.11471	9.32563	1.3886
2	9.778	MM	0.2628	8955.85742	567.98328	98.6114
Totals :				9081.97213	577.30890	

(*S*)-(Oxiran-2-ylmethyl)-4-phenylpiperazine (*S*-1j)

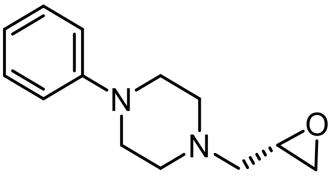

Additional Info : Peak(s) manually integrated

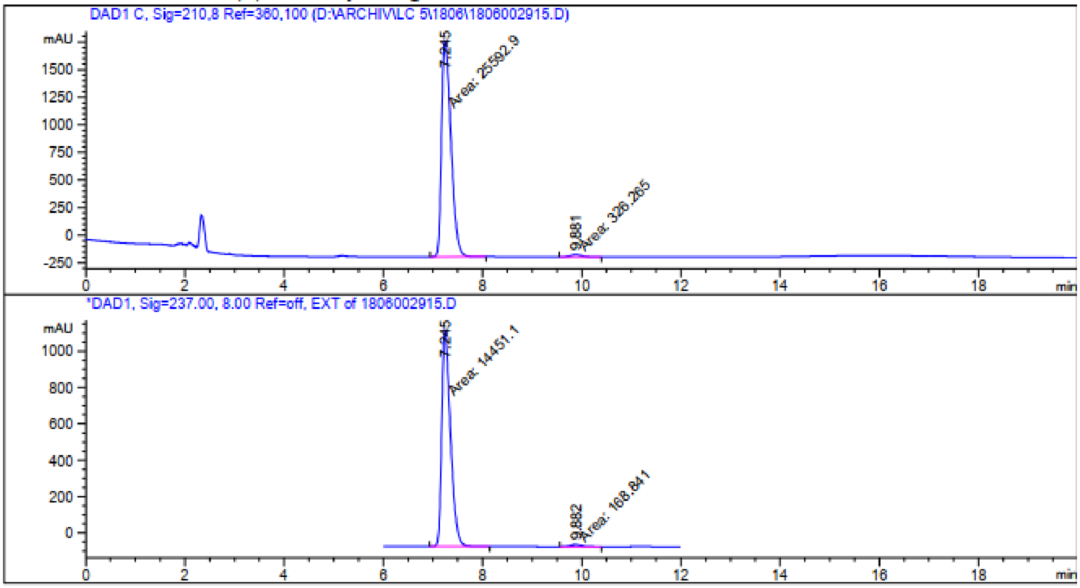

===

Area Percent Report

===

Sorted By : Signal
Multiplier : 1.0000
Dilution : 1.0000
Use Multiplier & Dilution Factor with ISTDs

Signal 1: DAD1 C, Sig=210,8 Ref=360,100

Peak #	RetTime [min]	Type	Width [min]	Area [mAU*s]	Height [mAU]	Area %
1	7.245	MM	0.2191	2.55929e4	1946.43115	98.7412
2	9.881	MM	0.2593	326.26523	20.96795	1.2588

Totals : 2.59192e4 1967.39910

Signal 2: DAD1, Sig=237.00, 8.00 Ref=off, EXT
Signal has been modified after loading from rawdata file!

Peak #	RetTime [min]	Type	Width [min]	Area [mAU*s]	Height [mAU]	Area %
1	7.245	MM	0.2027	1.44511e4	1188.15552	98.8451
2	9.882	MM	0.2610	168.84132	10.78093	1.1549

Totals : 1.46200e4 1198.93645

4-((4-Phenylpiperazin-1-yl)methyl)-1,3-dioxolan-2-one (*rac*-2j)

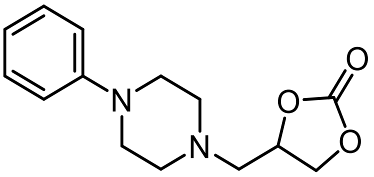

Additional Info : Peak(s) manually integrated

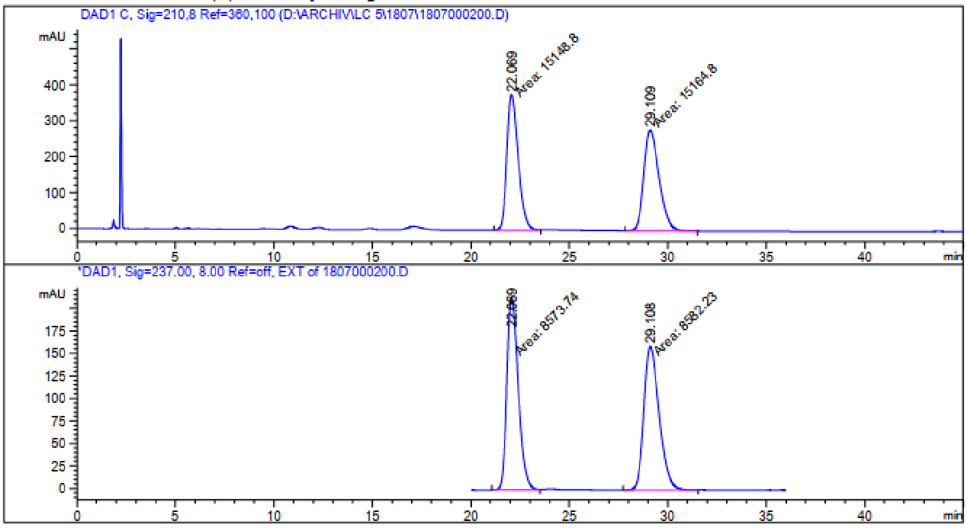

===
Area Percent Report
===

Sorted By : Signal
Multiplier : 1.0000
Dilution : 1.0000
Use Multiplier & Dilution Factor with ISTDs

Signal 1: DAD1 C, Sig=210,8 Ref=360,100

Peak #	RetTime [min]	Type	Width [min]	Area [mAU*s]	Height [mAU]	Area %
1	22.069	MF	0.6703	1.51488e4	376.66644	49.9736
2	29.109	MM	0.8993	1.51648e4	281.06229	50.0264

Totals : 3.03135e4 657.72873

Signal 2: DAD1, Sig=237.00, 8.00 Ref=off, EXT
Signal has been modified after loading from rawdata file!

Peak #	RetTime [min]	Type	Width [min]	Area [mAU*s]	Height [mAU]	Area %
1	22.069	MF	0.6673	8573.74023	214.15358	49.9753
2	29.108	MM	0.8978	8582.22656	159.32518	50.0247

Totals : 1.71560e4 373.47876

(*R*)-4-((4-phenylpiperazin-1-yl)methyl)-1,3-dioxolan-2-one (*R*-2j)

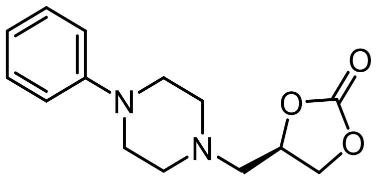

Signal 2: DAD1, Sig=237.00, 8.00 Ref=off, EXT
Signal has been modified after loading from rawdata file!

Peak #	RetTime [min]	Type	Width [min]	Area [mAU*s]	Height [mAU]	Area %
1	21.909	MF	0.6379	3222.30762	84.18455	38.0361
2	28.813	MM	0.8592	5249.39307	101.82453	61.9639

Totals : 8471.70068 186.00908

(*S*)-4-((4-Phenylpiperazin-1-yl)methyl)-1,3-dioxolan-2-one (*S*-2j)

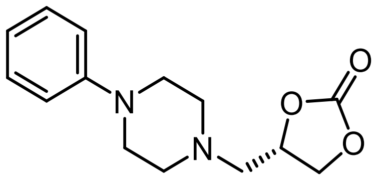

Additional Info : Peak(s) manually integrated

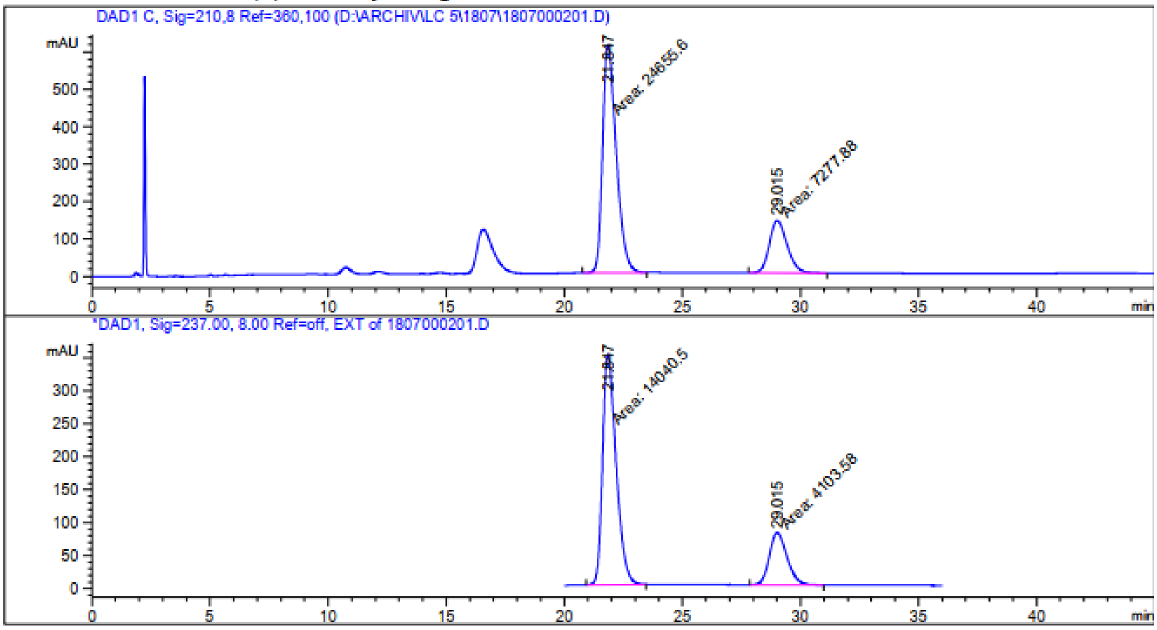


```
=====================================================================
                         Area Percent Report
=====================================================================

Sorted By             :      Signal
Multiplier            :      1.0000
Dilution              :      1.0000
Use Multiplier & Dilution Factor with ISTDs


Signal 1: DAD1 C, Sig=210,8 Ref=360,100

Peak RetTime Type  Width     Area       Height      Area
  #   [min]        [min]   [mAU*s]      [mAU]        %
----|-------|----|-------|----------|----------|--------|
   1  21.847 MF    0.6764 2.46556e4  607.55664  77.2093
   2  29.015 MM    0.8664 7277.88086  140.00128  22.7907

Totals :                  3.19335e4   747.55792


Signal 2: DAD1, Sig=237.00, 8.00 Ref=off, EXT
 Signal has been modified after loading from rawdata file!

Peak RetTime Type  Width     Area       Height      Area
  #   [min]        [min]   [mAU*s]      [mAU]        %
----|-------|----|-------|----------|----------|--------|
   1  21.847 MF    0.6700 1.40405e4  349.25845  77.3834
   2  29.015 MM    0.8656 4103.57568   79.01329  22.6166

Totals :                  1.81441e4   428.27174
```

3-(4-Phenylpiperazin-1-yl)propane-1,2-diol (*rac*-10)

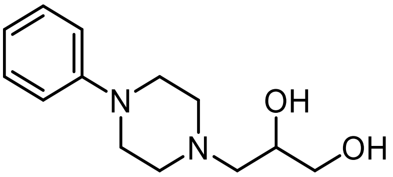


```
Additional Info : Peak(s) manually integrated
```

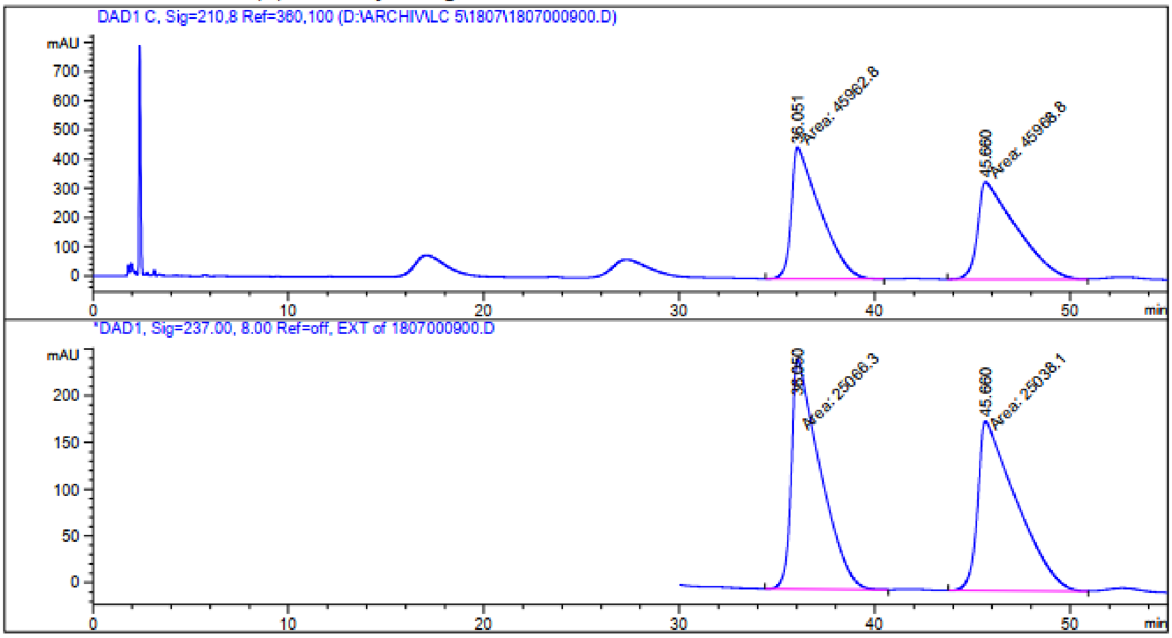


```
=====================================================================
                         Area Percent Report
=====================================================================

Sorted By             :      Signal
Multiplier            :      1.0000
Dilution              :      1.0000
Use Multiplier & Dilution Factor with ISTDs


Signal 1: DAD1 C, Sig=210,8 Ref=360,100

Peak RetTime Type  Width     Area      Height     Area
  #   [min]        [min]   [mAU*s]     [mAU]       %
----|-------|----|-------|----------|----------|--------|
   1  36.051 MM     1.7044 4.59628e4  449.45868  49.9967
   2  45.660 MM     2.3035 4.59688e4  332.60849  50.0033

Totals :                   9.19316e4  782.06717


Signal 2: DAD1, Sig=237.00, 8.00 Ref=off, EXT
 Signal has been modified after loading from rawdata file!

Peak RetTime Type  Width     Area      Height     Area
  #   [min]        [min]   [mAU*s]     [mAU]       %
----|-------|----|-------|----------|----------|--------|
   1  36.050 MM     1.6976 2.50663e4  246.09827  50.0281
   2  45.660 MM     2.3010 2.50381e4  181.36011  49.9719

Totals :                   5.01044e4  427.45837
```

(*R*)-3-(4-Phenylpiperazin-1-yl)propane-1,2-diol (*R*-10)

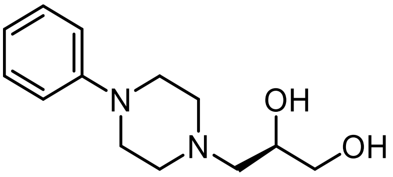

Additional Info : Peak(s) manually integrated

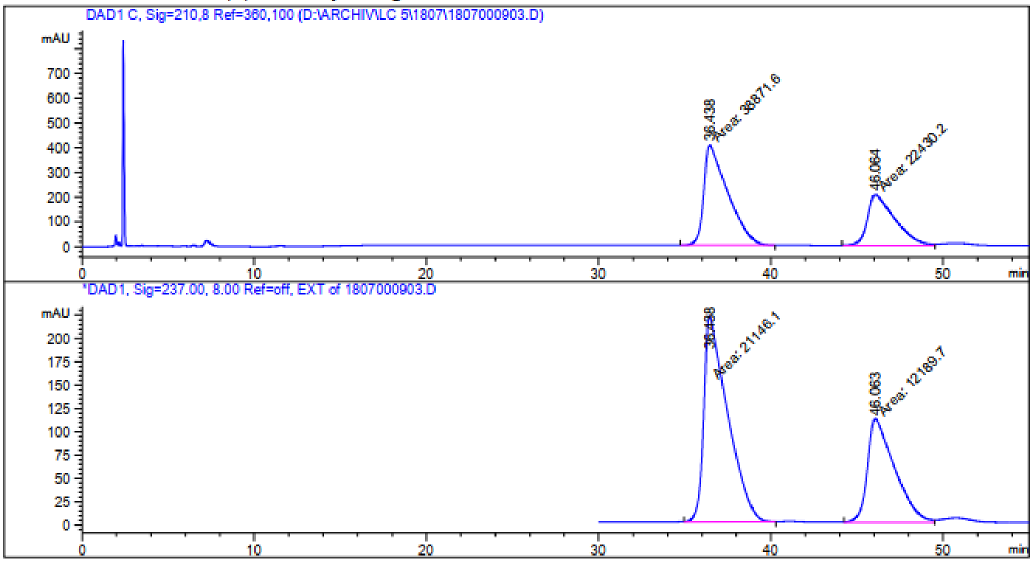


```
=====================================================================
                         Area Percent Report
=====================================================================

Sorted By             :      Signal
Multiplier            :      1.0000
Dilution              :      1.0000
Use Multiplier & Dilution Factor with ISTDs


Signal 1: DAD1 C, Sig=210,8 Ref=360,100

Peak RetTime Type  Width     Area      Height     Area
  #   [min]        [min]   [mAU*s]     [mAU]        %
----|-------|----|-------|----------|----------|--------|
   1  36.438 MM    1.6058 3.88716e4  403.44141  63.4102
   2  46.064 MF    1.8285 2.24302e4  204.45122  36.5898

Totals :                  6.13018e4  607.89262



Signal 2: DAD1, Sig=237.00, 8.00 Ref=off, EXT
 Signal has been modified after loading from rawdata file!

Peak RetTime Type  Width     Area      Height     Area
  #   [min]        [min]   [mAU*s]     [mAU]        %
----|-------|----|-------|----------|----------|--------|
   1  36.438 MM    1.5984 2.11461e4  220.49289  63.4336
   2  46.063 MF    1.8281 1.21897e4  111.13101  36.5664

Totals :                  3.33358e4  331.62390
```

(*S*)-3-(4-Phenylpiperazin-1-yl)propane-1,2-diol (*S*-10)

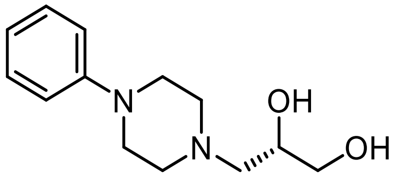

Additional Info : Peak(s) manually integrated

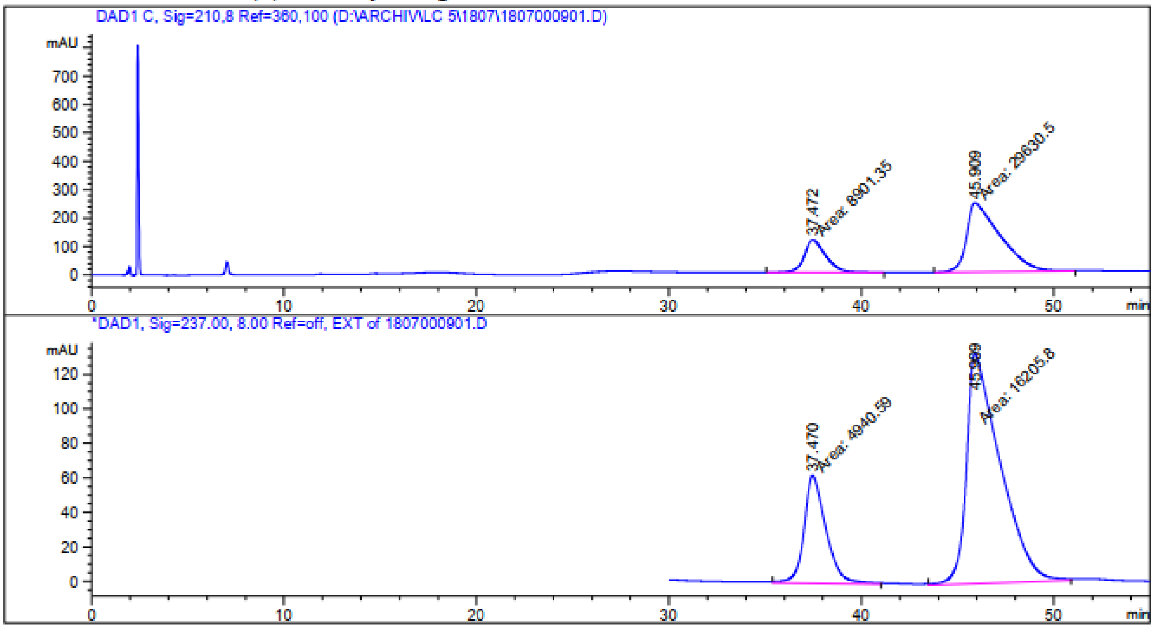


```
=====================================================================
                         Area Percent Report
=====================================================================

Sorted By             :      Signal
Multiplier            :      1.0000
Dilution              :      1.0000
Use Multiplier & Dilution Factor with ISTDs


Signal 1: DAD1 C, Sig=210,8 Ref=360,100

Peak RetTime Type  Width     Area      Height     Area
  #   [min]        [min]   [mAU*s]     [mAU]       %
----|-------|----|-------|----------|----------|--------|
   1  37.472 MM    1.2919 8901.34766  114.83495  23.1013
   2  45.909 MM    2.0214 2.96305e4   244.30763  76.8987

Totals :                  3.85318e4   359.14259
```

```
Signal 2: DAD1, Sig=237.00, 8.00 Ref=off, EXT
 Signal has been modified after loading from rawdata file!

Peak RetTime Type  Width     Area      Height     Area
  #   [min]        [min]   [mAU*s]     [mAU]       %
----|-------|----|-------|----------|----------|--------|
   1  37.470 MM    1.3186 4940.58594   62.44572  23.3637
   2  45.909 MM    2.0289 1.62058e4  133.12415  76.6363

Totals :                 2.11464e4   195.56987
```

14 References

[1] T. Werner, H. Büttner, *ChemSusChem* **2014**, *7*, 3268–3271.

[2] D. Wei-Li, J. Bi, L. Sheng-Lian, L. Xu-Biao, T. Xin-Man, A. Chak-Tong, *Appl. Catal. A* **2014**, *470*, 183–188.

[3] Y. Toda, Y. Komiyama, A. Kikuchi, H. Suga, *ACS Catal.* **2016**, *6*, 6906–6910.

[4] P. B. Kapadnis, E. Hall, M. Ramstedt, W. R. J. D. Galloway, M. Welch, D. R. Spring, *Chem. Commun.* **2009**, 538–540.

[5] H. Büttner, J. Steinbauer, C. Wulf, M. Dindaroglu, H. G. Schmalz, T. Werner, *ChemSusChem* **2017**, *10*, 1076–1079.

[6] J. Steinbauer, A. Spannenberg, T. Werner, *Green Chem.* **2017**, *19*, 3769–3779.

[7] Y. Hu, J. Steinbauer, V. Stefanow, A. Spannenberg, T. Werner, *ACS Sustainable Chem. Eng.* **2019**, *7*, 13257–13269.

[8] Y. Hu, S. Peglow, L. Longwitz, M. Frank, J. D. Epping, V. Brüser, T. Werner, *ChemSusChem* **2020**, *13*, 1825–1833.

[9] N. Tenhumberg, H. Büttner, B. Schäffner, D. Kruse, M. Blumenstein, T. Werner, *Green Chem.* **2016**, *18*, 3775–3788.

[10] G. Chłoń-Rzepa, A. Zagórska, A. Bucki, M. Kołaczkowski, M. Pawłowski, G. Satała, A. J. Bojarski, A. Partyka, A. Wesołowska, E. Pękala, *Arch. Pharm. Chem. Life Sci.* **2015**, *348*, 242–253.

[11] V. Laserna, G. Fiorani, C. J. Whiteoak, E. Martin, E. Escudero-Adán, A. W. Kleij, *Angew. Chem. Int. Ed.* **2014**, *53*, 10416–10419.

[12] G. Socrates, *Infrared and Raman Characteristic Group Frequencies*, 3rd ed., John Wiley & Sons, Chichester, **1994**.

[13] J. Steinbauer, C. Kubis, R. Ludwig, T. Werner, *ACS Sustainable Chem. Eng.* **2018**, *6*, 10778–10788.

[14] a) C. Kubis, D. Selent, M. Sawall, R. Ludwig, K. Neymeyr, W. Baumann, R. Franke, A. Börner, *Chem. Eur. J.* **2012**, *18*, 8780-8794; b) E. Hairer, G. Wanner, *Solving Ordinary Differential Equations 2, 2nd Ed.*, Springer, Berlin, **2002**; c) J. E. Dennis, D. M. Gay, R. E. Welsch, *ACS Trans. Math. Software* **1981**, *7*, 369–383.

[15] P. D. Mantor, O. Ablb, K. Y. Song, R. Kobayashi, *J. Chem. Eng. Data* **1982**, *27*, 243–245.

[16] M. J. Frisch, G. W. Trucks, H. B. Schlegel, G. E. Scuseria, M. A. Robb, J. R. Cheeseman, G. Scalmani, V. Barone, G. A. Petersson, H. Nakatsuji, X. Li, M. Caricato, A. Marenich, J. Bloino, B. G. Janesko, R. Gomperts, B. Mennucci, H. P. Hratchian, J. V. Ortiz, A. F. Izmaylov, J. L. Sonnenberg, D. Williams-Young, F. L. F. Ding, J. G. F. Egidi, A. P. B. Peng, T. Henderson, D. Ranasinghe, V. G. Zakrzewski, N. R. J. Gao, W. L. G. Zheng, M. E. M. Hada, K. Toyota, R. Fukuda, J. Hasegawa, M. Ishida, T. Nakajima, Y. Honda, O. Kitao, H. Nakai, T. Vreven, K. Throssell, J. A. M. Jr., J. E. Peralta, F. Ogliaro, M. Bearpark, J. J. Heyd, E. Brothers, K. N. Kudin, V. N. Staroverov, T. Keith, R. Kobayashi, J. Normand, K. Raghavachari, A. Rendell, J. C. Burant, S. S. Iyengar, J. Tomasi, M. Cossi, J. M. Millam, M. Klene, C. Adamo, R. Cammi, J. W. Ochterski, R. L. Martin, K. Morokuma, O. Farkas, J. B. Foresman, D. J. Fox, *Gaussian Inc.: Wallingford, CT, USA* **2009**.

[17] a) A. D. Becke, *J. Chem. Phys.* **1993**, *98*, 5648-5652; b) P. J. Stephens, F. J. Devlin, C. F. Chabalowski, M. J. Frisch, *The Journal of Physical Chemistry* **1994**, *98*, 11623–11627.

[18] F. Weigend, R. Ahlrichs, *Phys. Chem. Chem. Phys.* **2005**, *7*, 3297–3305.

[19] V. Trudel, A. K. Yudin, *Chem* **2019**, *5*, 2291–2293.

[20] A. V. Marenich, C. J. Cramer, D. G. Truhlar, *J. Phys. Chem. B* **2009**, *113*, 6378–6396.

[21] C. Legault, *Université de Sherbrooke* **2009**.
